# A Blood–Brain Barrier‐Permeable Guanylhydrazone Analogue of the ASIC3 Activator GMQ Inhibits Human Glioblastoma Stem Cell Growth In Vitro

**DOI:** 10.1002/cmdc.70438

**Published:** 2026-09-23

**Authors:** Leonardo Maiorana, Nicoletta Collura, Andrea Gotti, Giulia De Leonardis, Greta Donati, Alice Maiocchi, Luciana Marinelli, Andrea Menegon, Pierfausto Seneci

**Affiliations:** ^1^ Chemistry Department Università degli Studi di Milano – La Statale Milan Italy; ^2^ Experimental Imaging Centre IRCCS San Raffaele Scientific Institute Milan Italy; ^3^ Department of Pharmacy Università degli Studi di Napoli Federico Secondo Naples Italy; ^4^ Olon SpA Segrate Milan Italy

**Keywords:** ASIC3 channels, BBB permeability, cancer stem cell neurospheres, glioblastoma multiforme, guanyl hydrazones

## Abstract

The acid‐sensing ion channel 3 (ASIC3) is a neuronal voltage‐insensitive Na^+^ channel located in the peripheral nervous system (PNS) and activated by extracellular H^+^, that is dysregulated in peripheral neuropathic pain. ASIC3 was found in stem cells of central nervous system (CNS)–located glioblastoma multiforme (GBM CSCs), and its chronic activation kills dysfunctional GBM CSCs without any effect on ASIC3‐lacking CNS tissues. We rationally designed and synthesized blood–brain barrier (BBB)–compliant analogues of GMQ, a known guanidyl quinazoline ASIC3 activator; we replaced its guanidine group with a guanyl hydrazone (GH) and carried out scaffold substitutions and other structural variations in 16 GMQ analogues to establish a structure–activity relationship (SAR). Our more potent GH analogue **1a** showed specific activity against GBM CSC neurospheres, coupled with a better safety profile on mammalian nontumor cells and a better brain‐to‐plasma ratio compared with GMQ. Such results provide valuable insights for further structural optimization of heteroaryl GHs as ASIC3 modulators.

## Graphical abstract

The acid‐sensing ion channel 3 (ASIC3), physiologically located in the peripheral nervous system, was found in stem cells of CNS‐located glioblastoma multiforme (GBM CSCs); its chronic activation in the CNS kills dysfunctional GBM CSCs with no effect on ASIC3‐lacking CNS tissues. We synthesized 16 analogues of GMQ, a quinazoline ASIC3 activator; we replaced its guanidine with a guanyl hydrazone (GH) and established a structure–activity relationship (SAR). Our best hit **1a** showed activity against GBM CSC neurospheres, a better safety profile on mammalian nontumor cells and a better brain‐to‐plasma ratio than GMQ.

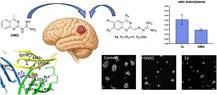

## 1 Introduction

Acid‐sensing ion channels (ASICs) [1] are voltage‐insensitive, proton‐gated channels mostly expressed in neurons, but found also in other human tissues. They assemble in homo‐ and heterotrimeric complexes, showing composition‐dependent properties [2]; the proton is their common activator, with pH sensitivity varying between 4.5 and 6.8 [3]. ASIC1 (1a,1b), ASIC2 (2a,2b), and ASIC3 (single isoform) were extensively characterized for their physiological and pathological roles [4], respectively in the central nervous system (CNS) for ASIC1,2 (stroke, multiple sclerosis, neurodegenerative diseases) and in the peripheral nervous system (PNS) for ASIC3 (pain). Peptide‐ and small‐molecule–based ASIC modulators with varying potencies and isoform selectivity were identified and characterized as putative treatments for neurological and psychiatric diseases [5].

ASICs also play a role in liver, cardiac, metabolic, and autoinflammatory nonneuronal pathologies [6]. Their acid‐dependent activation influences the proliferation, migration, and invasiveness of multiple tumor cell types, which express either a single or multiple ASIC isoforms in an acidic microenvironment [7]. ASIC1a [8] and ASIC3 [9] are expressed in brain tumors, particularly in glioblastoma (GBM) and in its cancer stem cells (GBM CSCs). In particular, the latter are considered responsible for GBM pharmacological resistance and tumor recurrence, thus representing a new attractive pharmacological target for GBM treatment [10].

**FIGURE 1:**
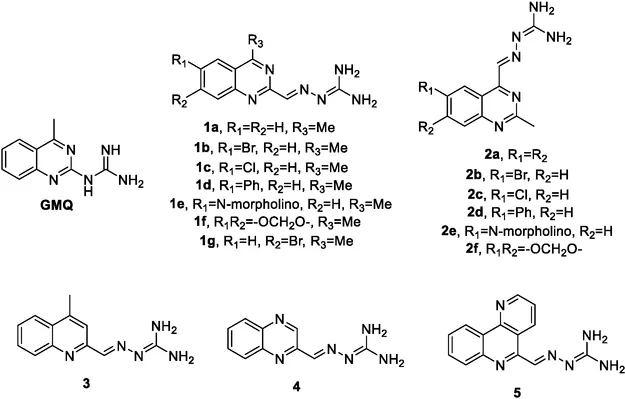
Chemical structure of GMQ and of GH‐based GMQ analogues **1a–5**.

Targeting CNS‐expressed ASIC1a to inhibit GBM is risky, due to the abundant ASIC1a‐dependent physiological neuronal processes that could be affected [3]. Conversely, the delivery of an ASIC3‐specific modulator in the brain to target GBM CSCs should not affect neuronal processes, due to the absence of ASIC3 in the CNS. In particular, we assessed the chronic activation of ASIC3 by a small molecule activator as an innovative anti‐GBM strategy, altering GBM CSC metabolism and cell cycle up to death [9]. Namely, 2‐guanidino‐4‐methylquinazoline (GMQ, Figure 1), a known ASIC3 activator [11], inhibited GBM CSC neurosphere growth more potently than the gold standard temozolomide and did not disturb control neurons [9], indicating its potential for the treatment of tumor pharmacological resistance and recurrence.

GMQ induced a nociceptive behavior in animal models of systemic pain [11], but was poorly permeable through the blood–brain barrier (BBB), preventing access to GMB CSCs at effective concentrations. Moderately basic aryl guanyl hydrazones (hereinafter GHs) have been used by us and others in BBB‐permeable drug leads [12]. We thus replaced the guanidine group in GMQ with a GH, as in GMQ‐like 2‐GH, 4‐Me quinazoline **1a** (Figure 1).

After assessing a versatile synthetic route, we synthesized an array of 16 heteroaryl GH analogues of GMQ, shown in Figure 1. In details, our efforts resulted in the synthesis of GMQ‐like (unsubstituted, 6‐, 6,7‐, 7‐substituted 2‐GH, 4‐Me quinazolines **1a–g**) and isomeric GMQ analogues (unsubstituted, 6‐, 6,7‐substituted 2‐Me, 4‐GH quinazolines **2a–f**) bearing a quinazoline scaffold. Initial scaffold‐hopping efforts [13] replaced the quinazoline scaffold with a quinoline (2‐GH, 4‐Me quinoline **3**), a quinoxaline (2‐GH quinoxaline **4**), and a tricyclic ring (5‐GH benzo[*h*][1,6] naphthyridine ring **5**). The GH array was evaluated in an in vitro screening on GBM CSC‐derived neurospheres, and GMQ‐like **1a** was identified as a promising bioavailable, safe hit.

## 2 Results and Discussion

### 2.1 Synthesis

**FIGURE 2:**
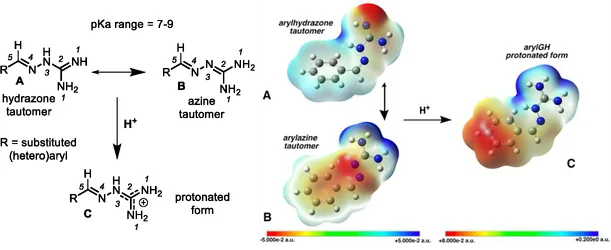
Tautomeric, pH‐dependent equilibria (left) and MEP maps (right) for (hetero)aryl GH forms (A,B) (neutral) and (C) (protonated). Color bars represent the electrostatic potential. The regions characterized by more negative electrostatic potential (red color) indicate areas with higher electron density, while regions characterized by more positive electrostatic potential (blue) indicate areas with lower electron density (Figure adapted from [12]).

Heteroaryl GHs typically show p*K*_a_ values ranging between 6.5 and 9 [14], adopting either a 1,4 nonconjugated/hydrazone or a 2,4‐conjugated/azine tautomeric form (respectively **A** and **B**, Figure 2, top left); the latter has a 4–6 kcal/mol stabilization preference. A protonated form **C** is observed upon acidification (bottom left). **A**, **B**, and **C** are characterized by different molecular electrostatic potential (MEP [15]) maps (Figure 2, right) and coexist at physiological pH; for example, antihypertensive α_2_‐adrenergic receptor agonist GH guanabenz shows a ≈16% neutral form abundance at pH 7.37 [16].

#### 2.1.1 GMQ‐Like 2‐GH, 4‐Me Quinazoline **1a**

**SCHEME 1:**
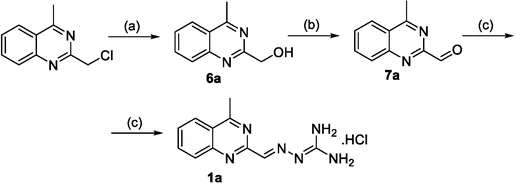
Synthesis of GMQ‐like 2‐GH, 4‐Me quinazoline **1a**. Reagents and conditions: (a) 2 M NaOH (20 eq.), 1,4‐dioxane, 80 °C, 6 h, 31%; (b) MnO_2_ (6 eq.), dry ACN, N_2_, 80 °C, 2 h, 41%; and (c) AG.HCl, EtOH, 80 °C, 7 h, 40% after reverse‐phase chromatography.

After an unsuccessful attempt centered around Se‐driven Riley oxidation [17], described in detail later, we assessed and carried out the synthesis of target GMQ‐like **1a** following the synthetic route depicted in Scheme 1.

A basic nucleophilic substitution (step a, Scheme 1) was carried out in highly diluted conditions to prevent polycondensation, obtaining alcohol **6a** in moderate yield. A short‐timed protocol entailing MnO_2_ in refluxing acetonitrile (ACN) yielded the sensitive aldehyde target **7a** in moderate yield (step b). Finally, standard condensation with aminoguanidine hydrochloride (hereinafter AG.HCl) in refluxing EtOH [12] yielded, after reverse‐phase chromatography, target 2‐GH, 4‐Me quinazoline **1a** in overall poor, unoptimized yields.

#### 2.1.2 Substituted GMQ‐Like 2‐GH, 4‐Me Quinazolines **1b–g**

**SCHEME 2:**
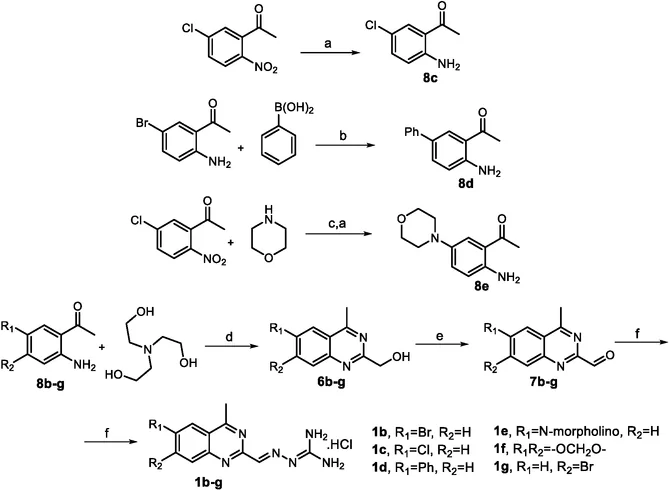
Synthesis of phenyl‐substituted 2‐GH, 4‐Me quinazolines **1b–1g**. Reagents and conditions: (a) Fe powder, aq. NH_4_Cl, EtOH, 70 °C, 2 h, 97% (**8c**), 86% (**8e**); (b) Pd(PPh_3_)_4_, 1:1 1,4‐dioxane/water, 100 °C, 3 h, 51%; (c) K_2_CO_3_, DMSO, N_2_, 110 °C, 5 h, 91%; (d) Eosin Y, NH_4_Cl, 1:1 ACN/water, blue LED (40 W), 30 °C, 22–72 h, 16% to 33%; (e) DMP, DCM, 0 °C to rt, 1 h, 35 to 71%; and (f) AG.HCl, EtOH, 80 °C, 7–14 h, 58% to quantitative, optional reverse‐phase chromatography.

In order to establish a better synthetic route toward substituted analogues of GMQ‐like **1a**, we used a metal‐free, visible‐light photoreaction between *o*‐aminoacetophenones, trialkylamines, and ammonium chloride, using eosin Y as a photocatalyst [18]. Its adaptation to our purposes and the synthesis of six substituted analogues of GMQ‐like **1a** are reported in Scheme 2.

We chose six *o*‐aminoacetophenones bearing different substituents. Three were commercially available, while chloro‐substituted **8c**, phenyl‐substituted **8d,** and *N*‐morpholino‐substituted **8e** resulted respectively from the reduction of chloro‐substituted *o*‐nitro (step a, Scheme 2, excellent yield), a Suzuki coupling on bromo‐substituted *o*‐amino (step b, moderate yield), and a two‐step substitution–reduction protocol on chloro‐substituted *o*‐nitroacetophenone (steps c and a, Scheme 2, good overall yield).

*o*‐Aminoacetophenones were then reacted with triethanolamine as a functionalized carbon synthon and ammonium chloride as a nitrogen source (step d), in a visible light–induced photochemical reaction catalyzed by the triarylmethane dye Eosin Y [18]. A slightly modified standard protocol, which could be further optimized, led to poor to moderate yields of hydroxymethyl quinazolines **6b–6g**. Dess–Martin periodinane (DMP) resulted the most effective among oxidation reagents, yielding moderate to good yields of formyl quinazolines **7b–7g** (step e). Target GMQ‐like analogues **1b–g** were obtained in good to quantitative yields and excellent purity by standard AG.HCl condensation (step f, Scheme 2).

**FIGURE 3:**
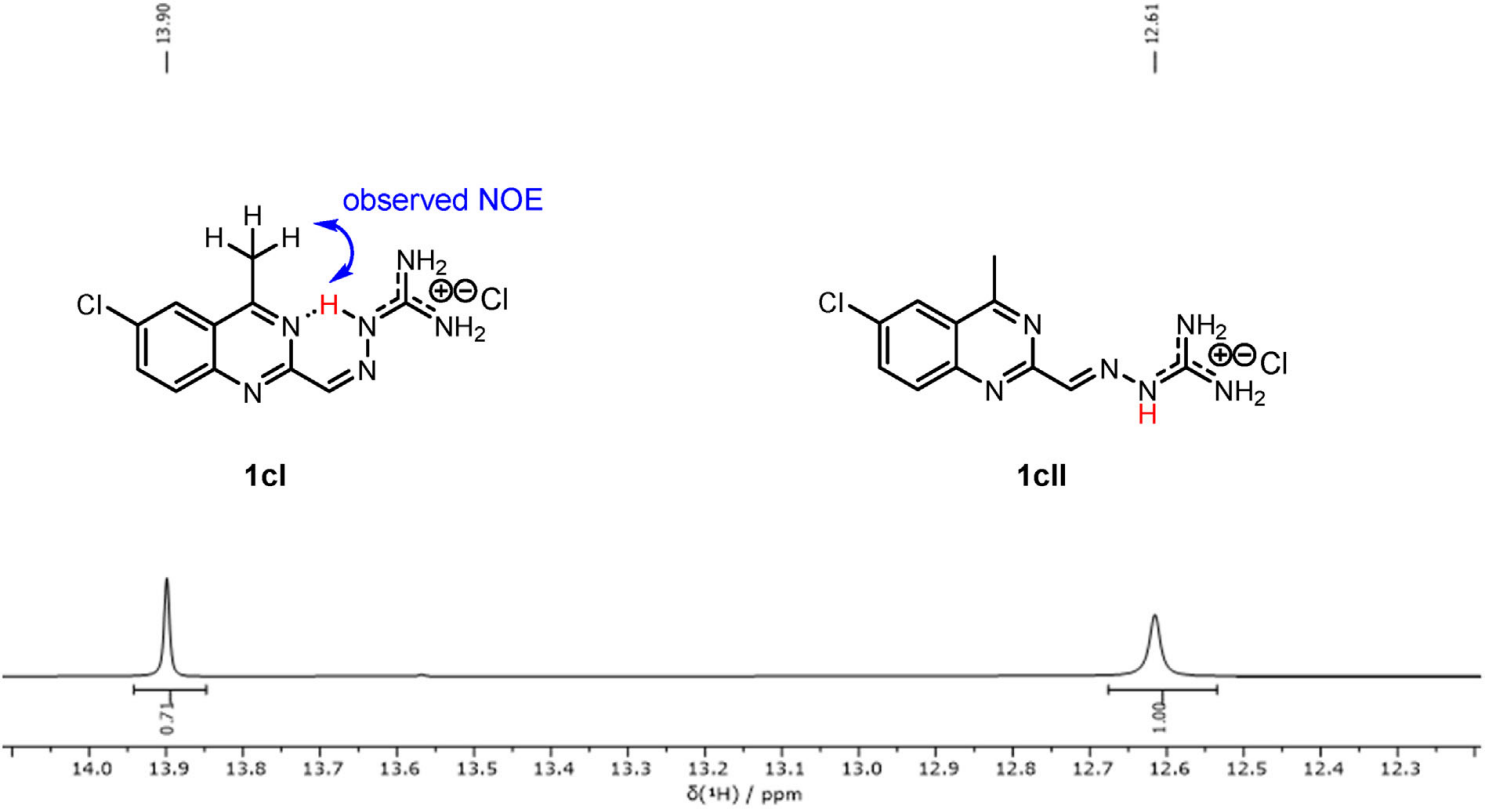
Tautomeric equilibrium for **1c**: proposed structures of **1cI** and **1cII** (top) and magnified downfield portion of its ^1^H‐NMR spectrum (bottom).

During their analytical characterization, we observed a quinazoline‐dependent change of the equilibrium between isomeric GH forms for GMQ‐like analogues **1b–g**. The ^1^H‐NMR signal of the N_3_ atom in the GHs was often split into the expected singlet at ≈12.6 ppm and a deshielded counterpart at ≈13.9 ppm—see for example the spectrum enlargement for GMQ‐like analogue **1c** in Figure 3 (the Nuclear Overhauser Effect SpectroscopY (NOESY) correlation is shown in Figure S1).

The deshielded signal is due to an intramolecular H‐bond established between the N_3_ atom in the GH, and the N_3_ atom in the quinazoline ring (“H‐bond,” Z structure **1cI**, Figure 3); such H‐bond forces the GH in a Z‐like/more compact form, compared to the E/linear form corresponding to the ≈12.6 ppm signal. The structural assignment for the isomeric “H‐bond” Z **1cI** and “linear” E **1cII** forms is confirmed by the observed NOE between the N_3_ atom in the GH and the 4‐Me group (Figure S1).

The **I**/**II** isoform ratio for our GH analogues **1a–5** is reported in Table 1. Please note that a NOE/H‐bond involving the N_1_ atom was not detected, possibly due to the inductive effect of the 4‐methyl group on the quinazoline N_3_, to enhance its basicity. Finally, a weaker H‐bond between the quinazoline N_3_ and the GH N_3_ was observed for isomeric GH analogues **2a–f**, that lack a vicinal methyl group to either N atom.

**TABLE 1:** Tautomeric equilibrium in GMQ‐like GH analogues **1a–5**: *E*/*Z*, **II**/**I** ratio.

GH analogue	*E*/*Z* ratio	GH analogue	*E*/*Z* ratio
**1a**	100/0	**2b**	88/12
**1b**	80/20	**2c**	95/5
**1c**	58/42	**2d**	92/8
**1d**	86/14	**2e**	87/13
**1e**	58/42	**2f**	87/13
**1f**	33/67	**3**	100/0
**1g**	78/22	**4**	100/0
**2a**	93/7	**5**	83/17

#### 2.1.3 Isomeric 2‐Me, 4‐GH Quinazolines **2a–f**

**SCHEME 3:**
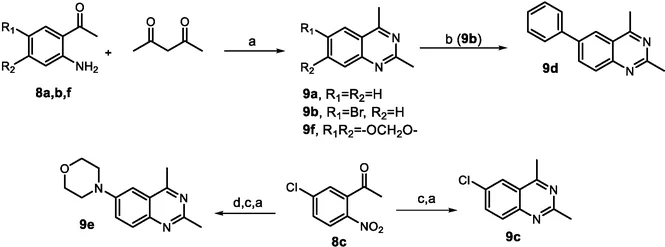
Synthesis of substituted 2,4‐diMe quinazolines **9a–f**. Reagents and conditions: (a) NH_4_Cl, neat, 100 °C, 7–10 h, 40% to 72%; (b) Pd(PPh_3_)_4_, Na_2_CO_3_, 2:2:1 toluene/EtOH/water, 110 °C, 6 h, 91%; (c) Fe powder, aq. NH_4_Cl, EtOH, 70 °C, 2 h, 86% to 97%; and (d) morpholine (2.5 eq.), dry DMF, N_2_, 110 °C, 5 h, 91%.

As mentioned, initially we targeted 2‐formyl, 4‐Me quinazolines through Se‐based Riley oxidation of 2,4‐dimethyl quinazolines, as reported for similar 4‐aryl substituted, 2‐formyl quinazolines [19], and substituted 2‐formyl, 4‐Me pyrimidines [20]. Thus, 2,4‐dimethyl quinazolins **9a–f** were prepared in one to three steps from either *o*‐amino‐ (**8a,b,f**) or *o*‐nitroacetophenones (**8c**), as shown in Scheme 3.

*o*‐Aminoacetophenone was reacted with acetylacetone as a carbon synthon and ammonium acetate as a nitrogen source (step a, Scheme 3). Heating in neat conditions yielded moderate yields of 2,4‐diMe quinazoline **9a**; better yields were observed from 6‐Br‐ (quinazoline **9b**) and 6,7‐dioxolanyl‐substituted (quinazoline **9f**) *o*‐aminoacetophenones. Quinazoline **9b** was submitted to Suzuki coupling with phenylboronic acid in standard conditions (step b), providing 6‐Ph quinazoline **9d** in excellent yields.

Commercial 2‐nitro, 5‐Cl acetophenone **8c** was reduced in almost quantitative yield with Fe/ammonium chloride (step c), and the resulting *o*‐aminoacetophenone was smoothly converted to 6‐Cl, 2,4‐diMe quinazoline **9c** (step a) in good yields. Precursor **8c** was also alkylated in excellent yield with *N*‐morpholine while heating (step d); the resulting *o*‐nitroacetophenone was submitted to the same nitro reduction—quinazoline formation procedure (steps c and a, Scheme 3) to yield 6‐*N*‐morpholino, 2,4‐diMe quinazoline **9e** in overall good yields.

**SCHEME 4:**
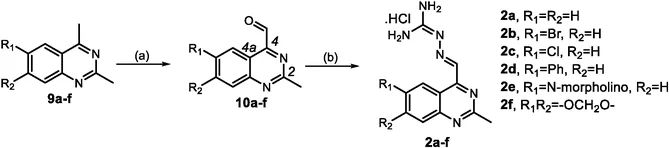
Synthesis of phenyl‐substituted 2‐Me, 4‐GH quinazolines **2a–2f**. Reagents and conditions: (a) SeO_2_, dry 1,4‐dioxane, N_2_, 100 °C, 30 min to 3 h, 33% to 74% and (b) AG.HCl, EtOH, 80 °C, 7 h, 23% to quantitative, optional reverse‐phase chromatography.

2,4‐diMe quinazolines **9a–f** were then submitted to Riley oxidation, expecting such protocol to regioselectively yield earlier described 2‐GH **1a** and its **1b–1f** analogues. The reaction outcome is shown in Scheme 4.

A standard Riley oxidation protocol converted 2,4‐diMe quinazolines **9a–f** into mono‐Me quinazoline aldehydes (step a, Scheme 4) in moderate to good yields. Surprisingly, the observed NMR long‐range correlation between the formyl group and the quinazoline C_4_ and C_4a_ carbons and between the protons of the residual methyl group and the quinazoline C_2_ carbon unequivocally determined a substituted 2‐Me, 4‐formyl quinazoline structure for intermediates **10a–f**. Finally, their condensation with AG.HCl in standard conditions (step b, Scheme 4) yielded isomeric 2‐Me, 4‐GH quinazolines **2a**–f in good to excellent yields.

#### 2.1.4 Scaffold Hopping: 2‐GH, 4‐Me Quinoline **3**, 2‐GH Quinoxaline **4** and 5‐GH Benzo[h][1,6] Naphthyridine **5**

**SCHEME 5:**
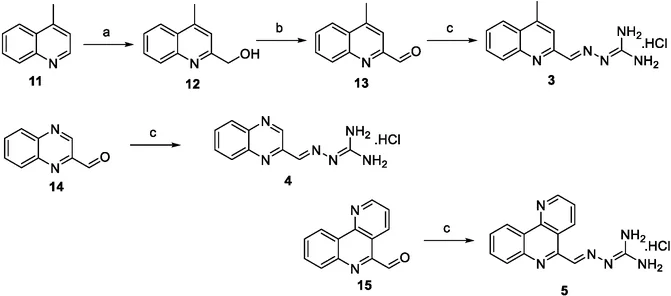
Synthesis of 2‐GH, 4‐Me quinoline **3** (top), 2‐GH quinoxaline **4** (middle), and 5‐GH benzo[h][1,6] naphthyridine **5** (bottom). Reagents and conditions: (a) Na_2_S_2_O_8_, *p*‐TosOH, 7:3 MeOH/water, 70 °C, 4 h, 88%; (b) DMP, DCM, 0 °C to rt, 1 h, 23%; and (c) AG.HCl, EtOH, 80 °C, 7 h, 80% to 93%.

Target 2‐GH, 4‐Me quinoline **3** was prepared from lepidine **11**, that was oxidatively functionalized in position 2 with sodium persulfate in excellent yield to 2‐hydroxymethyl quinoxaline **12** (step a, Scheme 5). The latter was partially oxidized with DMP (step b) to 2‐formyl quinoxaline **13** in poor, unoptimized yield, and eventually condensed with AG.HCl in standard conditions (step c, top) to yield target 2‐GH, 4‐Me quinoline **3** in excellent yield.

Both 2‐GH quinoxaline **4** and 5‐GH benzo[h][1,6] naphthyridine **5** were obtained in good yield and purity through a standard AG.HCl condensation on 2‐formyl quinoxaline **14** (step c, middle) and 5‐formyl benzo[h][1,6] naphthyridine **15** (step c, bottom, Scheme 5), respectively.

### 2.2 Biological Screening

#### 2.2.1 In Vitro Efficacy, CSC Neurospheres

**FIGURE 4:**
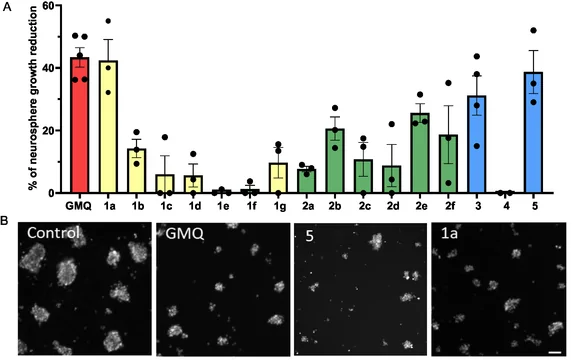
Panel (A) Activity screening of GMQ and heteroaryl GH analogues **1a–5** on MES‐0315 GBM CSC 3D neurospheres through an automated 3D assay that quantifies both the neurospheres number and surface area, to determine total tumor growth inhibition in a 3D culture system after treatment at 10 µM for 72 h. Dots represent the percentage of neurosphere growth inhibition in independent experiments, normalized to untreated control samples (100%). *n* = 3 for all samples except for GMQ *n* = 6 and sample 4 *n* = 2. Bars represent the standard error from the mean that represents the % of reduction of neurosphere growth (see Table 2). A statistical test by multiple comparison one‐way Anova using α 0.05, revealed no statistical difference between GMQ and GHs **1a**, **2e**, **3,** and **5** (GraphPad Prism). Panel (B) Images of neurospheres under control condition as well as after treatment with GMQ, GHs **5,** and **1a** at 10 µM. Bar length: 50 µm.

GMQ (10 µM) showed marked efficacy in controlling GBM CSCs growth on four GBM CSC subtypes in a 2D model of GBM CSCs, as previously reported [9]. Mesenchymal (MES) CSCs were also affected in a more predictive 3D neurosphere assay, where GMQ impacted on CSC proliferation and, thus, reduced both the number and the size of MES‐0315 neurospheres [9]. We tested MES‐0315 GBM CSCs with the 16‐membered array of heteroaryl GH analogues **1a–5** (single concentration, 10 µM) for 72 h on the 3D neurosphere assay, observing the activity panel depicted in Figure 4, panel A. The percentage inhibition on MES‐0315 GBM CSC 3D neurospheres after treatment with GHs **1a** **–5** is reported in Table 2.

**TABLE 2:** % inhibition on MES‐0315 GBM CSC 3D neurospheres for heteroaryl GHs **1a–5**.

GH analogue	% inhibition, 10 μM	GH analogue	% inhibition, 10 μM
**GMQ**	43.4	**2a**	7.6
**1a**	42.4	**2b**	20.6
**1b**	14.2	**2c**	10.8
**1c**	6	**2d**	8.8
**1d**	5.6	**2e**	25.5
**1e**	a	**2f**	18.7
**1f**	a	**3**	31.2
**1g**	9.7	**4**	a
—	—	**5**	38.7

a Not detectable, ≤2%.

While GMQ confirmed a strong effect on GBM neurospheres growth at 10 µM, we were pleased to observe comparable activity by GMQ‐like 2‐GH **1a**; its close analogues **1b–f** (yellow, Figure 4, panel A) showed a lower but often measurable inhibition of 3D neurospheres. Isomeric 4‐GH quinazolines **2a–f** (green) often showed activity, in particular for dioxolane‐substituted **2e**. Scaffold‐hopping analogue **5** showed slightly lower 3D neurosphere growth inhibition than GMQ and **1a**; an image of treated GBM CSC neurospheres treated at 10 µM with GMQ, **5** and **1a** is also provided in Figure 4, panel B.

#### 2.2.2 Cytotoxicity

**FIGURE 5:**
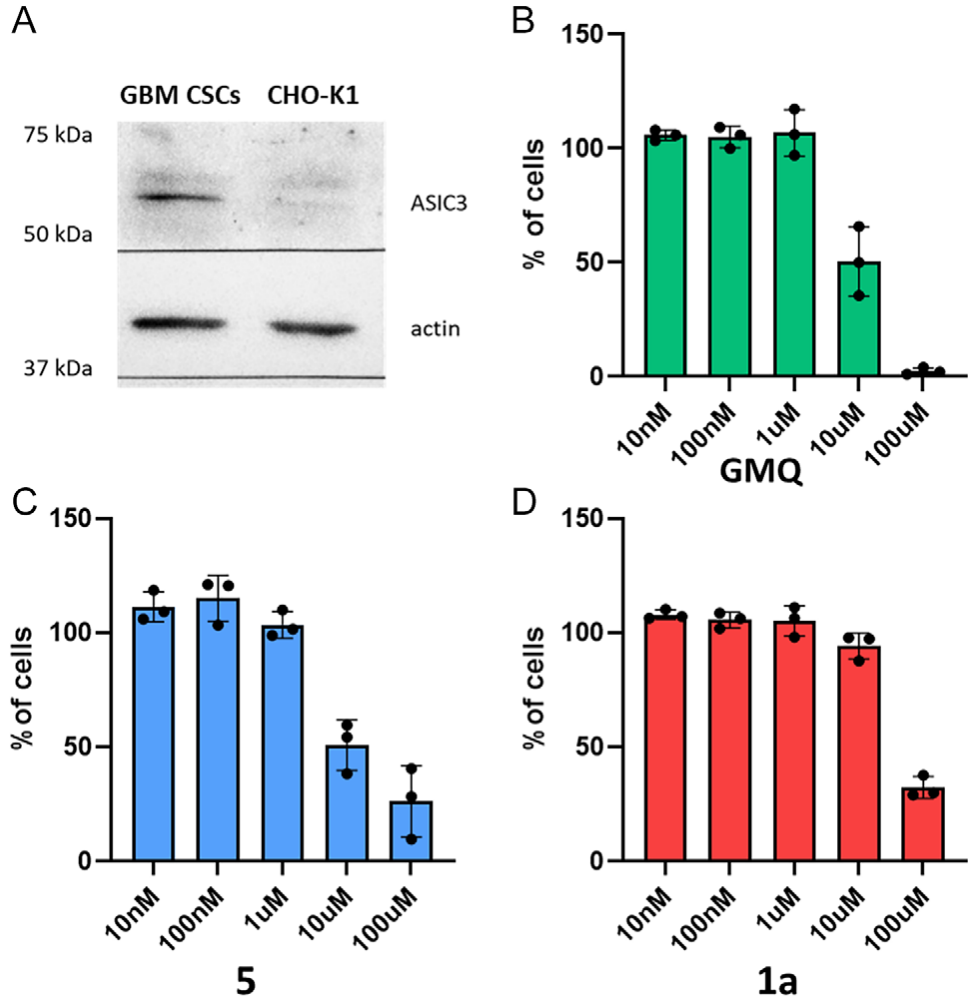
Chinese hamster ovary (CHO‐K1) cells that do not express ASIC3 (A) were treated with GMQ as a control (B), GHs **5** (C), and **1a** (D) for 72 h at different concentrations, to determine any toxic effect on this ASIC3‐lacking mammalian cells. Dots represent individual experiments (*n* = 3), and bars represent the standard deviation from the mean. Please note the expression of ASIC3 in human GBM CS shown in (A), in agreement with data reported in [9].

Both **1a** and **5** were submitted to a cytotoxicity assay in vitro, to determine their therapeutic window of efficacy. Increasing concentrations of both GHs were added to CHO‐K1 cells, known to express ion channels at low level and, in particular, to lack ASIC3 expression (Figure 5, panel A). The number of cells was measured after 72 h treatment at different concentrations of GMQ (panel B), **5** (panel C), and **1a** (panel D). While both GMQ and **5** showed partial toxicity at 10 μM, **1a** was safe at this concentration. As CHO‐K1 cells do not express ASIC3, the lack of any effect by **1a** at 10 μM supports its specific, ASIC3‐driven cytotoxicity on GBM CSCs. Considering its good specific activity and safety profile in vitro, GMQ‐like 2‐GH **1a** was selected as a promising hit for further characterization.

#### 2.2.3 In Vivo BBB Permeability

**FIGURE 6:**
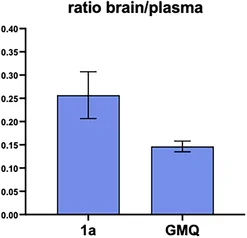
Plasma–brain ratios measured after i.v. injection of **1a** (left) and GMQ (right), both at 2 mg/kg, in mice sacrificed after 15 min. Bars represent the standard deviation from the mean (*n* = 3 mice per compound).

A pathway toward GBM treatment for any small molecule requires access and possibly accumulation in the brain; thus, we selected **1a** as a representative GMQ‐like GH analogue, it studied the BBB permeability compared to GMQ after i.v. injection in mice of both compounds. Namely, three mice per compound were dosed i.v. (2 mg/kg) and sacrificed 15 min after the injection; their average brain/plasma ratio was determined to be 14.5% for GMQ and 25.4% for **1a** (Figure 6).

While a single time point does not allow to determine any kinetic parameter, the higher brain/plasma ratio observed for GMQ‐like GH **1a** indicates a higher propensity for BBB permeation and a lower systemic distribution, to be quantitated in future studies.

### 2.3 In Silico ADMET Profiling of 1a

A computational ADMET prediction for GMQ‐like GH **1a**, carried out with the Deep‐PK tool [21], suggested an overall favorable profile for its protonated form. The compound is expected to display high intestinal absorption and good oral bioavailability, together with the ability to cross the BBB. In addition, it showed moderate plasma protein binding and a moderate volume of distribution, suggesting adequate systemic distribution. The predicted metabolic profile of **1a** indicated limited interactions with major CYP isoforms, with CYP1A2 being the only partially inhibited enzyme. The cardiac safety profile of **1a** was assessed using the CUPID platform [22], which predicts interactions with the cardiac ion channels hERG, Nav1.5, and Cav1.2. GMQ‐like GH **1a** was consistently classified as safe in all three models, suggesting a low liability for cardiac ion channel‐mediated toxicity; an applicability domain analysis indicated that these cardiac safety profile predictions fall within the reliable chemical space of the respective models, supporting their robustness. Finally, as to nephrotoxicity risks, we used the KidneyTox_v1 tool [23], where SHapley Additive eXplanations (SHAP) analysis indicated a moderate nephrotoxicity risk.

### 2.4 Modeling Studies

In the absence of an experimentally resolved structure for the human ASIC3 monomer, we took advantage of a multicomponent artificial intelligence (AI) system AlphaFold2, which uses machine learning to accurately predict a protein's 3D structure based solely on its primary amino acid sequence [24]. Using the published structure of *Gallus gallus* ASIC1 (PDB ID: 6AVE) as a template, we reconstructed the trimeric assembly of hASIC3. This structure was selected over other available entries (e.g., PDB IDs: 4NTW, 2QTS), as it represents the resting state (closed) of the channel, that is, the conformation known to bind GMQ.

**FIGURE 7:**
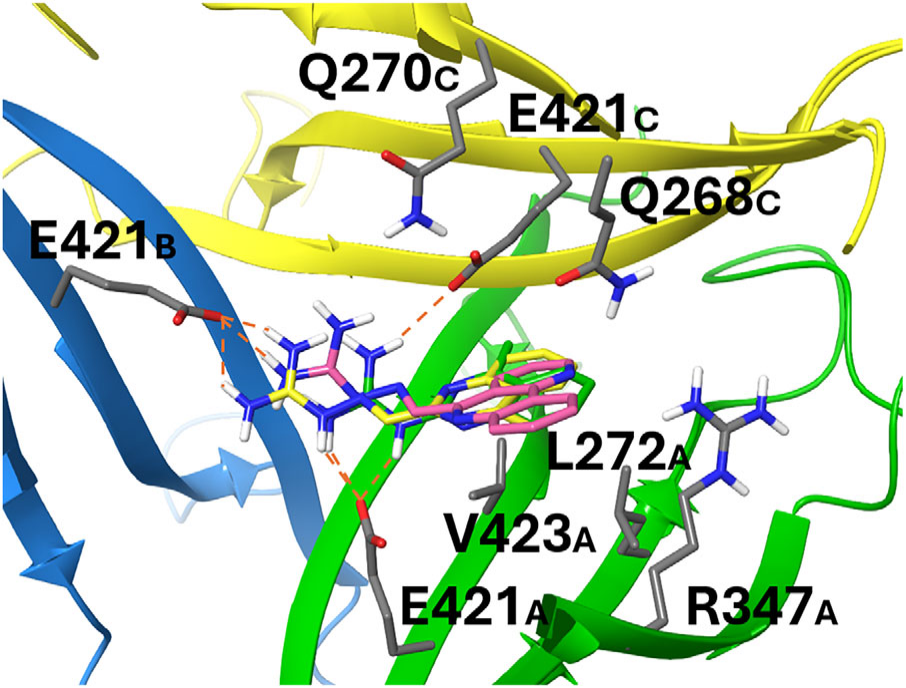
Molecular docking of GMQ, **1a**, and **5** (shown in green, yellow, and pink sticks representation, respectively) in the hASIC3 closed‐state 3D model. The AlphaFold2 model of human ASIC3 is represented as a color‐coded cartoon representation (green, (A); blue, (B); yellow, (C)). The main residues of the binding pocket are represented in sticks. Key interactions between GMQ, GHs **1a**, and **5** and the receptor are highlighted in orange dashed lines.

As for docking GMQ, **1a**, and **5** in the selected homotrimer (green, monomer A; blue, monomer B; and yellow, monomer C, Figure 7), the Glide software was used [25]. According to extensive single‐point mutagenesis experiments [26] and previous computational studies [27], GMQ was accommodated in a large cavity of the palm domain delimited by residues Leu76_(A)_, Leu272_(A)_, Arg 374_(A)_, Glu421_(A)_, Val423_(A)_, Glu421_(B)_, Leu76_(C)_, Glu78_(C)_, Gln268_(C)_, Gln270_(C)_, and Glu421_(C)_. Molecular docking of the three small molecules in our hASIC3 model revealed highly overlapping binding poses (Figure 7), consistent with their similar biological activities.

Although precise structure–activity relationships (SARs) cannot be established, given that we used a cell‐based assay in which ligand efficacy is influenced by factors other than receptor binding, we may draw some considerations for the most representative subset of GHs **1a–1g**. Namely, our docking results (Figure 7) suggest that an elongation of the polar chain from guanidine (GMQ) to guanylhydrazone (**1a**) should be tolerated; conversely, a substitution at the R_1_/6‐position should not be well tolerated, regardless of the substituent type, due to steric hindrance within the binding pocket. Both modeling observations are coherent with experimental data (see Table 2, GHs **1d–f**). The introduction of a chlorine or a bromine in the R_1_/6 or R_2_/7 position could reduce the hydrogen bond–donating ability of a guanidine (GMQ) or GH moiety (**1a**), possibly increasing the affinity for ASIC3; such theoretical assumption was verified for GMQ [11], but not for our GHs (compare **1a** with less potent, halogenated **1b**, **1c,** and **1g** in Table 2).

Overall, our results provide valuable SAR insights for designing a next generation of GMQ‐like ligands. In particular, they indicate that (i) replacing the guanidine group with a BBB‐permeable GH moiety is well tolerated; and (ii) tricyclic GH analogues bearing a fused ring at R_3_ (e.g., compound **5**) are well tolerated and may represent a new strategy to enhance their affinity for ASIC3. Moreover, replacing the quinazoline ring with a smaller heterocyclic scaffold to be further functionalized without active site clashes could represent a viable strategy for the design of a next generation of ASIC3 activators.

## 3 Conclusion

Putative candidates for GBM treatment, acting through innovative mechanisms, are actively sought against a tumor that shows poor pharmacological therapeutic opportunities and frequent recurrence, also through drug resistance mechanisms [28]. In addition to efficacy, a good safety profile, access to, and accumulation into the brain are key elements to determine the potential and usefulness of new drug candidates.

Here, we present a new class of heteroaryl GHs capable of significantly affecting the growth of GBM CSCs through ASIC3, thus reducing the growth of GBM CSC neurospheres. GBM CSCs are relevant drug targets for an efficient therapy, being capable to self‐renew, differentiate, and adapt to the tumor microenvironment. Several studies have demonstrated their resistance to conventional chemotherapy (e.g., temozolomide) and radiation, contributing to tumor growth and therapy resistance, thus representing important target cells for novel anticancer therapies [29].

Although the efficacy of the selected GMQ‐like hit **1a** was comparable to GMQ, the former showed lower toxicity in CHO‐K1 cells that do not express ASIC3. This indicates a specific ASIC3‐mediated activity in controlling GBM CSC growth, as well as a safer pharmacological profile probably due to fewer off‐target effects. In fact, the guanidine–GH shift provided a significant advantage for in vivo BBB penetration. Noteworthy, the presented SAR—particularly the activity observed for structurally distinct analogues, such as the tricyclic 5‐GH **5**—suggests that the quinazoline ring is not essential for maintaining ASIC3‐stimulating activity and could be replaced with alternative scaffolds, thus offering new, precious opportunities for derivatization.

Further structural optimization of these molecules may therefore represent a promising avenue toward the pharmacological treatment of GBM through ASIC3 modulation using BBB‐compliant, small heteroaryl GHs; we are progressing along this research avenue, and in due time, will present additional results.

## 4 Experimental Section

### 4.1 Synthesis

#### 4.1.1 General

Oven‐dried glassware was used to carry out chemical reactions, and dry solvents under a nitrogen atmosphere were employed. Purification of intermediates and final products was carried out by flash chromatography, using high‐purity grade silica gel as a stationary phase; alternatively, purification was performed by a BIOTAGE system using Biotage Sfar Duo cartridges (4, 10, or 25 g) for direct‐phase chromatography or Biotage Sfar Duo C_18_ cartridges (6, 12, or 30 g) for reverse‐phase chromatography. Reaction monitoring by thin layer chromatography (TLC) entailed precoated 60F_254_ plates, using UV light at 254 nm as a direct detection method or by charring either with a phosphomolybdic acid ethanolic solution, with a potassium permanganate, or a ninhydrin solution. ^1^H NMR and ^13^C NMR spectra were recorded in CDCl_3_, CD_3_OD, or DMSO‐*d*_6_, depending on compounds’ solubility. Chemical shifts (*δ*) for proton and carbon signals are quoted relatively to the solvent's peak and expressed in parts per million (ppm). Ultra‐high performance liquid chromatography/mass spectrometry analysis (UPLC/MS) was performed with a tunable ultraviolet (TUV) detector, a single quadrupole (SQD) mass spectrometer, and RP_18_ columns (2.1 × 100 mm, id = 1.7 μm).

#### 4.1.2 General Procedure A: Synthesis of Phenyl‐Substituted 2‐Hydroxymethyl‐4‐Methylquinazolines **6b–g** From Substituted o‐Aminoacetophenones **8b–g**

A substituted *o*‐aminoacetophenone (1 eq.) was dissolved in 1:1 ACN/H_2_O (0.15 M). Triethanolamine (5 eq.), NH_4_Cl (3 eq.), and eosin Y (0.1 eq.) were sequentially added, and the reaction mixture was stirred under 40W blue LED Kessil lamp irradiation (467 or 456 nm) for 22–72 h, monitored by TLC. A needle was inserted into the reaction tube cap to ensure access to air for the reaction mixture; an external fan was used to ensure that the reaction temperature did not exceed 35–40 °C. After reaction completion, the mixture was diluted with H_2_O and extracted four times with EtOAc. The combined organic phases were dried over Na_2_SO_4_, and the solvent was removed under reduced pressure. The crude was purified via direct‐phase flash column chromatography, affording a substituted 2‐hydroxymethyl‐4‐methylquinazoline either as a pure compound, or as an inseparable mixture with the 2‐unsubstituted byproduct as a minor component.

#### 4.1.3 General Procedure B: Synthesis of 2‐Aldehydo‐4‐Methylquinazolines **7b–g**, **13** by Oxidation of Phenyl‐Substituted 2‐Hydroxymethyl‐4‐Methylquinazolines **6b–g**, **12** With DMP

Solid DMP (1.3 eq.) was added to a stirred solution of alcohol (1 eq.) in dry DCM (0.1 M) under nitrogen atmosphere at 0 °C; after 15 min, the reaction was warmed to rt and further stirred for 1–2 h, monitored by TLC. After reaction completion, a sat. solution of Na_2_S_2_O_3_ was added to the mixture, followed by extracting four times with DCM. The combined organic phases were washed with a sat. solution of NaHCO_3_, dried over anhydrous Na_2_SO_4_, and concentrated under reduced pressure. The crude was purified by flash chromatography to obtain a pure aldehyde intermediate.

#### 4.1.4 General Procedure C: Synthesis of Quinazoline GHs **1a–g, 2a–f, 3–5** From Quinazoline Carboxaldehydes **7a–g, 10a–f, 13–15**

Aminoguanidine hydrochloride (AG^.^HCl, 0.90–0.96 eq.) was added to a vigorously stirred solution of aldehyde (1 eq.) in EtOH. The reaction mixture was stirred at reflux for 7–8 h and at room temperature overnight, monitored by TLC. After reaction completion, the reaction mixture was concentrated under reduced pressure to a solid residue. The residue was triturated three times in DCM, Et_2_O, or (*i‐*Pr)_2_O, then filtered and, if necessary, submitted to a reverse‐phase chromatographic separation to obtain a pure quinazoline GH.

#### 4.1.5 General Procedure D: Synthesis of Phenyl‐Substituted 2,4‐Dimethylquinazolines **9a–f** From Substituted o‐Aminoacetophenones **8a–f**

A substituted *o*‐aminoacetophenone (1.0 eq.) was introduced in a flask with two necks under nitrogen atmosphere. Then, NH_4_OAc (3.0 eq.) and acetylacetone (1.2 eq.) were added, and the resulting mixture was stirred at 100 °C for 7–10 h, monitored by TLC. After reaction completion, the mixture was quenched with water and extracted three times with EtOAc. The combined organic phases were washed with brine, dried over Na_2_SO_4_, and the solvent was removed under reduced pressure. The crude was purified via direct‐phase flash column chromatography, to obtain a pure quinazoline intermediate.

#### 4.1.6 General Procedure E: Synthesis of 4‐Formyl‐2‐Methylquinazolines **10a–f** by Riley Oxidation of 2,4‐Dimethylquinazolines **9a–f** With SeO_2_

A substituted 2,4‐dimethylquinazoline (1.0 eq.) was dissolved in dry 1,4‐dioxane (0.2 M) under nitrogen atmosphere. Solid SeO_2_ (either 1.0 or 2.0 eq.) was added, and the suspension was stirred at 80 °C for 30 min–3 h, monitored by TLC. The reaction mixture was cooled to rt and filtered over a Celite pad, washing with either EtOAc or DCM. The resulting crude was purified by direct‐phase flash column chromatography, affording either the pure 4‐formyl‐2‐methyl quinazoline intermediate, or an inseparable mixture with its 2‐regioisomer as a minor component.

### 4.2 2‐((4‐Methylquinazolin‐2‐yl)methylene)hydrazine‐1‐Carboximidamide Hydrochloride 1a

#### 4.2.1 Synthesis of (4‐Methylquinazolin‐2‐yl)methanol **6a**

2 M NaOH (16.4 mL, 33.0 mmol, 21 eq.) was added dropwise to a stirred solution of 2‐(chloromethyl)‐4‐methylquinazoline (300 mg, 1.6 mmol, 1 eq.) in 1,4‐dioxane (26 mL). The reaction mixture was stirred at 80 °C for 6 h, monitored by TLC (eluent mixture: 1:1 *n*‐Hex/EtOAc). After reaction completion, the mixture was cooled to rt and extracted with EtOAc (3 × 30 mL). The collected organic phase was dried over Na_2_SO_4_, and the solvent was removed under reduced pressure. The crude was purified via direct‐phase flash column chromatography (eluent mixture: 6:4 DCM/EtOAc), obtaining pure (4‐methylquinazolin‐2‐yl)methanol **6a** as a yellow solid (80 mg, 0.50 mmol, 31% yield). Analytical characterization: ^1^H‐NMR (400 MHz, CDCl_3_) *δ* 8.11 (dd, *J* = 8.2, 1.1 Hz, 1H, H5), 8.01 (d, *J* = 8.4 Hz, 1H, H8), 7.90 (ddd, *J* = 8.4, 6.9, 1.1 Hz, 1H, H7), 7.63 (ddd, *J* = 8.2, 6.9, 1.2 Hz, 1H, H6), 4.94 (s, 2H, H9), 4.04 (s, 1H, OH), 2.97 (s, 3H, H10). Our spectroscopic data are consistent with literature data [30].

#### 4.2.2 Synthesis of 4‐Methylquinazoline‐2‐Carbaldehyde **7a**

(4‐Methylquinazolin‐2‐yl)methanol **6a** (460 mg, 0.92 mmol, 1 eq.) was dissolved in dry ACN (9.2 mL) under nitrogen atmosphere. Solid MnO_2_ (480 mg, 5.52 mmol, 6 eq.) was added, and the resulting suspension was stirred at 80 °C for 2 h, monitored by TLC (eluent mixture: 1:1 DCM/EtOAc). After cooling to rt, the reaction mixture was filtered over a Celite pad, washing with EtOAc (125 mL), and then the solvent was removed under reduced pressure. The crude was purified via direct‐phase flash column chromatography (eluent mixture: 8:2 DCM/EtOAc), obtaining pure 4‐methylquinazoline‐2‐carbaldehyde **7a** as a yellow solid (65 mg, 0.38 mmol, 41% yield). Analytical characterization: ^1^H‐NMR (400 MHz, CDCl_3_) *δ* 10.23 (s, 1H, H9), 8.24 (ddd, *J* = 8.4, 1.3, 0.7 Hz, 1H, H5), 8.21 (ddd, *J* = 8.4, 1.4, 0.7 Hz, 1H, H8), 8.02 (ddd, *J* = 8.4, 7.0, 1.4 Hz, 1H, H6), 7.82 (ddd, *J* = 8.4, 6.9, 1.2 Hz, 1H, H7), 3.09 (s, 3H, H10). ^13^C‐NMR (101 MHz, CDCl_3_) *δ* 192.3, 170.3, 155.3, 149.7, 134.7, 130.4, 130.3, 125.4, 125.0, 22.1.

#### 4.2.3 Synthesis of 2‐((4‐Methylquinazolin‐2‐yl)methylene)hydrazine‐1‐Carboximidamide Hydrochloride **1a**

The reaction was carried out in 7 h according to general procedure C, using 4‐methylquinazoline‐2‐carbaldehyde **7a** (18.0 mg, 0.104 mmol, 1.0 eq.), AG·HCl (10.4 mg, 0.094 mmol, 0.9 eq.), and EtOH (1.0 mL). Trituration was done in (i‐Pr)_2_O (2 × 2.5 mL). After purification, pure GMQ‐like target 2‐((4‐methylquinazolin‐2‐yl)methylene)hydrazine‐1‐carboximidamide hydrochloride **1a** was obtained as a yellow solid (11 mg, 0.042 mmol, 40% yield), in a pure E/II tautomeric form. Analytical characterization: MS (ESI^+^): *m*/*z* [M + H^+^] 229.03. Calculated MS for C_11_H_13_N_6_: 229.12. ^1^H‐NMR (400 MHz, DMSO‐*d*_6_) *δ* 12.31 (s, 1H, H11), 8.36 (s, 1H, H9), 8.34 (d, *J* = 8.3 Hz, 2H, H5), 8.08–8.02 (m, 2H, H6, H8), 7.85 (brs, 4H, H12), 7.80 (dt, *J* = 8.2, 3.8 Hz, 1H, H7), 2.98 (s, 3H, H10). ^13^C‐NMR (101 MHz, DMSO‐*d*_6_) *δ* 169.3, 169.3, 155.9, 155.6, 149.1, 146.6, 134.7, 128.7, 128.4, 126.0, 123.0, 21.7, 21.6, 21.6.

### 4.3 2‐((6‐Chloro‐4‐Methylquinazolin‐2‐yl)methylene)hydrazine‐1‐Carboximidamide Hydrochloride 1c

#### 4.3.1 Synthesis of (6‐Chloro‐4‐Methylquinazolin‐2‐yl)methanol **6c**

The reaction was carried out in 22 h according to general procedure A, using 1‐(2‐amino‐5‐chlorophenyl)‐ethan‐1‐one **8c** (190 mg, 1.12 mmol, 1 eq.), triethanolamine (835 mg, 5.6 mmol, 5 eq.), eosin Y (73 mg, 0.11 mmol, 0.1 eq.), NH_4_Cl (189 mg, 3.36 mmol, 3 eq.), and 1:1 ACN/H_2_O (7.4 mL). After purification, pure (6‐chloro‐4‐methylquinazolin‐2‐yl)methanol **6c** was obtained as a white solid (77 mg, 0.37 mmol, 33% yield). Analytical characterization: ^1^H NMR (400 MHz, CDCl_3_) *δ* 8.08 (d, *J* = 2.3 Hz, 1H, H5), 7.96 (d, *J* = 8.9 Hz, 1H, H8), 7.83 (dd, *J* = 8.9, 2.3 Hz, 1H, H7), 4.93 (s, 2H, H9), 3.91 (s, 1H, —OH), 2.94 (s, 3H, H10).

#### 4.3.2 Synthesis of 6‐Chloro‐4‐Methylquinazoline‐2‐Carbaldehyde **7c**

The reaction was carried out in 1 h according to general procedure B, using (6‐chloro‐4‐methylquinazolin‐2‐yl)methanol **6c** (70 mg, 0.34 mmol, 1.0 eq.), DMP (185 mg, 0.44 mmol, 1.3 eq.), and dry DCM (3.3 mL). After purification, pure 6‐chloro‐methylquinazoline carbaldehyde **7c** was obtained as a pale yellow solid (49 mg, 0.24 mmol, 71% yield). Analytical characterization: ^1^H NMR (400 MHz, CDCl_3_) *δ* 10.16 (s, 1H, H9), 8.17–8.10 (m, 2H, H5, H8), 7.90 (dd, *J* = 9.0, 2.4 Hz, 1H, H7), 3.01 (s, 3H, H10).

#### 4.3.3 Synthesis of 2‐((6‐Chloro‐4‐Methylquinazolin‐2‐yl)methylene)hydrazine‐1‐Carboximidamide Hydrochloride **1c**

The reaction was carried out in 7 h according to general procedure C, using 6‐chloro‐methylquinazoline carbaldehyde **7c** (49 mg, 0.24 mmol, 1 eq.), AG^.^HCl (25 mg, 0.23 mmol, 0.96 eq.), and EtOH (2.4 mL). Trituration was done in (*i‐*Pr)_2_O (3 × 3 mL). After purification, pure target 2‐((6‐chloromethylquinazolinyl)methylene)hydrazine‐1‐carboximidamide hydrochloride **1c** was obtained as a white solid (49 mg, 0.16 mmol, 72% yield), in a 58/42 *E*/*Z*, II/I tautomeric form ratio. Analytical characterization: MS (ESI^+^): *m*/*z* [M + H^+^] 263.13. Calculated MS for C_11_H_12_ClN_6_: 263.08. ^1^H NMR (400 MHz, DMSO‐*d*_6_) *δ* 13.90 (s, 1H, H11′), 12.61 (s, 1H, H11), 8.73–8.36 (brs, 4H, H12′), 8.57 (d, *J* = 9.0 Hz, H8′), 8.44 (d, *J* = 2.3 Hz, 1H, H5′), 8.40 (s, 1H, H5), 8.34 (s, 1H, H9), 8.15 (dd, *J* = 9.0, 2.3 Hz, 1H, H7′), 8.04 (m, 2H, H7, H8), 8.01 (brs, 4H, H13), 7.73 (s, 1H, H9′), 3.02 (s, 3H, H10′), 2.96 (s, 3H, H10). ^13^C NMR (101 MHz, DMSO‐*d*_6_) *δ* 170.14, 169.22, 156.11, 155.79, 155.60, 154.69, 147.66, 146.34, 145.83, 138.28, 135.61, 135.28, 133.92, 132.89, 131.04, 130.59, 125.19, 125.07, 123.78, 123.29, 21.80, 21.64.

### 4.4 4‐((2‐Methylquinazolinyl)methylene)hydrazine‐1‐Carboximidamide Hydrochloride 2e

#### 4.4.1 Synthesis of 6‐(2,4‐Dimethylquinazolinyl)morpholine **9e**

The reaction was carried out in 7 h according to general procedure D, using NH_4_OAc (116 mg, 1.50 mmol, 3.0 eq.), 1‐(2‐amino‐5‐morpholinophenyl)ethan‐1‐one **8e** (110 mg, 0.50 mmol, 1.0 eq.), and acetylacetone (60 mg, 0.60 mmol, 1.2 eq.). Pure 6‐(2,4‐dimethylquinazolin‐6‐yl)morpholine **9e** was obtained as a brown solid (77 mg, 0.32 mmol, 60% yield). Analytical characterization: ^1^H‐NMR (400 MHz, CDCl_3_) *δ* 7.82 (d, *J* = 9.2 Hz, 1H, H8), 7.58 (dd, *J* = 9.2, 2.7 Hz, 1H, H7), 7.14 (d, *J* = 2.7 Hz, 1H, H5), 3.94–3.87 (m, 4H, H11, H12), 3.30–3.23 (m, 4H, H13, H14), 2.84 (s, 3H, H10), 2.79 (s, 3H, H9). ^13^C‐NMR (101 MHz, CDCl_3_) *δ* 166.3, 161.2, 149.5, 145.5, 129.1, 126.0, 123.1, 106.0, 66.9, 49.5, 26.2, 21.9.

#### 4.4.2 Synthesis of 6‐Morpholino‐2‐Methylquinazoline‐4‐Carbaldehyde **10e**

The reaction was carried out in 30 min according to general procedure B, using 4‐(2,4‐dimethylquinazolin‐6‐yl)morpholine **9e** (50 mg, 0.20 mmol, 1 eq.), SeO_2_ (23 mg, 0.20 mmol, 1.0 eq.), and 1,4‐dioxane (1.0 mL). Washing after Celite pad filtration was done using EtOAc (125 mL). After purification, pure methyl‐6‐morpholinoquinazoline 4‐carbaldehyde **10e** was obtained as a yellow solid (38 mg, 0.15 mmol, 74% yield). Analytical characterization: ^1^H‐NMR (400 MHz, CDCl_3_) *δ* 10.28 (s, 1H, H10), 8.31 (d, *J* = 2.8 Hz, 1H, H5), 7.92 (d, *J* = 9.5 Hz, 1H, H8), 7.70 (dd, *J* = 9.5, 2.8 Hz, 1H, H7), 3.95–3.89 (m, 4H, H11, H12), 3.41–3.34 (m, 4H, H13, H14), 2.94 (s, 3H, H9). ^13^C‐NMR (101 MHz, CDCl_3_) *δ* 196.6, 161.8, 153.2, 151.7, 149.6, 129.3, 126.7, 121.1, 105.1, 67.2, 67.1, 49.4, 49.0, 26.2.

#### 4.4.3 Synthesis of 4‐((2‐Methyl‐6‐Morpholinoquinazolinyl)methylene)hydrazine‐1‐Carboximidamide Hydrochloride **2e**

The reaction was carried out in 7 h according to general procedure C, using methyl‐6‐morpholinoquinazoline 4‐carbaldehyde **10e** (29 mg, 0.11 mmol, 1 eq.), AG^.^HCl (11 mg, 0.10 mmol, 0.90 eq.) and EtOH (1.1 mL). Trituration was done in Et_2_O (2 × 2.5 mL) and DCM (2.5 mL). After purification, pure 4‐((2‐methyl‐6‐morpholinoquinazolinyl)methylene)hydrazine‐1‐carboximidamide hydrochloride **2e** was recovered as a yellow solid (16 mg, 0.05 mmol, 41% yield), in a 87/13 *E*/*Z*, II/I tautomeric form ratio. Analytical characterization: MS (ESI^+^): *m*/*z* [M + H^+^] 314.12. Calculated MS for C_15_H_20_N_7_O: 314.17. ^1^H NMR (400 MHz, DMSO‐*d*_6_) *δ* (ppm) 12.32 (s, 1H, H10), 8.66 (s, 1H, H1′), 8.49 (s, 1H, H1), 8.33 (brs, 4H, H11′), 8.08 (d, *J* = 2.6 Hz, 1H, H2), 7.96 (dd, *J* = 9.4, 2.4 Hz, 1H, H2′), 7.88 (dd, *J* = 9.3, 2.6 Hz, 1H, H3), 7.81 (d, *J* = 9.3 Hz, 1H, H4), 7.75 (brs, 4H, H11), 4.07 (q, *J* = 4.9 Hz, 4H, H7′, H8′), 3.41 (t, *J* = 4.9 Hz, 4H, H6′, H9′), 3.77 (dd, *J* = 6.0, 3.4 Hz, 4H, H7, H8), 3.35–3.29 (m, 4H, H6, H9), 2.88 (s, 3H, H5′), 2.70 (s, 3H, H5). ^13^C NMR (101 MHz, DMSO‐*d*_6_) *δ* 160.0, 156.1, 155.7, 155.6, 150.0, 147.4, 147.3, 146.9, 128.4, 126.4, 120.7, 105.9, 66.0, 47.9, 25.4.

### 4.5 2‐(Benzo[h][1,6] Naphthyridin‐5‐ylmethylene)hydrazine‐1‐Carboximidamide Hydrochloride 5

#### 4.5.1 *Synthesis of* 2*‐*(*Benzo*[*h*][*1*,*6*]*naphthyridin‐5‐ylmethylene*)*hydrazine‐1‐Carboximidamide Hydrochloride* ***5***

The reaction was carried out in 7 h according to general procedure C, using commercial benzo[h]3[1,6]naphthyridine‐5‐carbaldehyde **15** (100 mg, 0.480 mmol, 1.0 eq.), AG.HCl (52 mg, 0.466 mmol, 0.97 eq.), and EtOH (4.80 mL). Five drops of 1 M HCl were added to the mixture prior to heating. Trituration was done in Et_2_O (2 × 5 mL). Pure target 2‐(benzo[h[1,6]naphthyridin‐5‐ylmethylene)hydrazine‐1‐carboximidamide hydrochloride **5** was obtained as a green solid (112 mg, 0.372 mmol, 80% yield) in a 87/13 *E*/*Z*, II/I tautomeric form ratio, without further purification. Analytical characterization: MS (ESI^+^): *m*/*z* [M + H^+^] 265.24. Calculated MS for C_14_H_12_N_6_: 264.29. ^1^H NMR (600 MHz, DMSO‐*d*_6_) *δ* 14.86 (s, 1H, H11′), 12.86 (s, 1H, H11), 9.64 (dd, *J* = 8.4, 1.6 Hz, 1H, H3), 9.32–9.27 (m, 3H, H5, H3′, H5′), 9.09 (dd, *J* = 8.2, 1.5 Hz, 2H, H7, H7′), 8.91–8.76 (bs, 4H, H12′), 8.80 (d, *J* = 8.1 Hz, 1H, H10′), 8.74 (s, 1H, H1), 8.67 (s, 1H, H1′), 8.29 (d, *J* = 8.1 Hz, 1H, H10), 8.11 (bs, 4H, H12), 7.99 (ddd, *J* = 8.3, 6.9, 1.5 Hz, 1H, H8′), 7.96 (ddd, *J* = 8.3, 6.9, 1.5 Hz, 1H, H8), 7.94–7.90 (m, 2H, H9′, H4′), 7.90–7.86 (m, 2H, H9, H4). ^13^C‐NMR (101 MHz, DMSO‐*d*_6_) *δ* 155.5, 153.9, 150.8, 148.3, 147.8, 144.1, 136.9, 131.1, 129.1, 128.7, 124.7, 124.1, 123.5, 118.7. Only the signals for the major form are reported.

### 4.6 Biological Screening

#### 4.6.1 GBM CSC Culture

GBM specimens were collected from patients with histologic diagnosis of primary GBM (WHO Grade 4 glioma) after informed consent was obtained from all subjects and/or their legal guardian(s). The experimentation has been performed following the ethical standards of the Declaration of Helsinki and in accordance with the protocol 01‐CSC07 approved by the Institutional Ethical Review Board of San Raffaele Scientific Institute to R.G. MES GBM CSCs were established at the Neural Stem Cell Biology Unit, San Raffaele Scientific Institute, Milan, Italy, and previously validated [31, 32]. The cells were cultured under the conditions of the NeuroSphere Assay (NSA) [33]. Briefly, GBM CSCs were cultured at 37 °C in Neurocult medium (NeuroCult NS‐A Proliferation Kit, human) (Cat: 5751; Voden, Monza, Italy) supplemented with hEGF (20 ng/µL; Cat: SRP3027‐500UG; Sigma‐Aldrich, Burlington, VT, USA), hFGF (10 ng/µL; Cat: PHG0026; Life Technologies Italia, Monza, Italy), and heparin (0.0002%; Cat: 7908; Voden, Monza, Italy). Neurospheres were routinely subcultured by mechanical dissociation and re‐plated under the same in vitro conditions. All the cultures were routinely tested for mycoplasma contamination.

#### 4.6.2 CHO‐K1 Culture

CHO‐K1 cells [34] (RRID: CVCL_0214, AXXAM S.p.A., Bresso (MI), Italy) were cultured in a 1:1 DMEM‐F12 Mix with Ultraglutamine I (Lonza Group Ltd., Basel, Switzerland) supplemented with 10% fetal bovine serum (FBS; EuroClone S.p.A., Pero, MI, Italy), 13 mM HEPES buffer (Lonza Group Ltd.), 0.375% sodium bicarbonate solution (Lonza Group Ltd.), 1 mM sodium pyruvate solution (Lonza Group Ltd.), and 1% Pen/Strep solution (Lonza Group Ltd.) at 37 °C in a humidified atmosphere with 5% CO_2_.

#### 4.6.3 Pharmacological Treatment

Cells were treated with either GMQ hydrochloride (Cat: SML0874‐50MG; Sigma–Aldrich, Burlington, VT, USA) or with heteroaryl GHs **1a–5**, for 72 h. The treatments were applied 24 h after cell plating and repeated after 36 h. GMQ was dissolved in DMSO at 25 mM and then diluted to 10 μM in the final medium (0.3% as the highest final concentration); heteroaryl GHs were dissolved in DMSO at 10 mM and then diluted to 10 μM in the final medium.

#### 4.6.4 GBM CSC Survival Assay

Single GBM CSCs were seeded at 5 × 10^3^ per well in black 96‐well plates with a transparent flat bottom (Cat GR655090, Euroclone, Milan, Italy). Cells were then treated twice over a 72h period with GMQ or with heteroaryl GHs **1a–5**. At the end of the experiment, 100 µL of medium was removed, and then the neurospheres were incubated with 1:5.000 Hoechst diluted in medium for 15 min. Then, the neurospheres were imaged with a Thermo Scientific ArrayScan XTI High Content Analysis (HCA) Reader at 5× magnification; four fields per well and five wells per condition were used for single experiments. The analysis was performed by Fiji‐ImageJ 2.15.1 software (https://imagej.net/software/fiji). First a threshold was applied, and then, only neurospheres larger than 500 µm^2^ were considered. The total neurosphere area/field value was calculated by multiplying the total neurosphere number/field by the average neurosphere area/field.

#### 4.6.5 CHO‐K1 Survival Assay

Cells were seeded at 7 × 10^3^ per well in black 96‐well plates with a transparent flat bottom (Cat: GR655090; Euroclone, Milan, Italy). Cells were then incubated for 15 min with DRAQ5 10 mM (Cat: 62251; Thermo Fisher Scientific, Waltham, MA, USA) diluted in DMEM F‐12. The total number of cell nuclei was measured with a Thermo Scientific ArrayScan XTI High Content Analysis (HCA) Reader (Thermo Fisher Scientific, Waltham, MA, USA). Using 20× magnification, 16 fields per well in each plate were acquired, and three wells per condition were used. For the analysis, HCS Studio software was used (Thermo Fisher Scientific, Waltham, MA, USA). All the DRAQ5‐positive nuclei in each field were quantified with the software. Total cell nuclei were quantified and normalized to the mean value of the controls.

#### 4.6.6 Immunoblotting

Immunoblotting has been performed as in [9]. Briefly, cells were mechanically detached and centrifuged at 8000 rpm for 10 min, then incubated for 10 min on ice in 50 µL of RIPA buffer [Tris–HCl (25 mM), NaCl (150 mM), EDTA (2 mM), SDS (0.1%), sodium deoxycholate (1%), and Triton‐100X (1%)]. The lysates were centrifuged for 15 min at 13,000 rpm at 4 °C. The proteins were quantified using a BCA assay kit (Thermo Fisher Scientific, Waltham, MA, USA). The blotted membranes were blocked with 5% BSA in TBS‐Tween for 45 min at room temperature. Subsequently, the membranes were probed overnight at 4 °C with primary antibodies against ASIC3 (Cat: ASC‐018; Alomone Labs, Jerusalem, Israel) and anti Actin (A5441 Sigma Aldrich, Merck KGaA, Darmstadt, Germany). Afterward, the membranes were incubated with HRP‐linked secondary antibodies diluted in TBS‐Tween with 1:5000 5% BSA for 1 h at room temperature [antirabbit (Cat: 7074S) and antimouse (Cat: 7076S); Cell Signaling, Danvers, MA, USA]. After incubation with the secondary antibodies, the membranes were treated with ECL Prime Western Blotting Detection reagent (Cat: GEHRPN2232; Euroclone, Milan, Italy). Blot images were acquired using a ChemiDoc MP imaging system (Bio‐Rad, Hercules, CA, USA). Different portions of the blot were cut at the appropriate molecular weight region, in order to perform multiple staining, and the actin counterstain was used for quantification on the same blot. The intensities of the protein bands were analyzed via the free software Fiji ImageJ.

#### 4.6.7 Brain Studies in Mice—Calibration

Brain tissues were homogenized in 20 mM ammonium formate (1 g/5 mL). Calibration curves and QC samples were prepared in mouse blank plasma or blank brain homogenate by adding 2.5 µL of each stock solution to 22.5 µL matrix. Samples (25 µL) and calibrants were added with 150 µL of cold WS_IS_ solution containing 0.1 µM Verapamil in MeOH. Samples were shaken for 15 min on an orbital shaker at room temperature, placed at 4 °C for 10 min and centrifuged for 15 min at 4600 *g* at 5 °C. The liquid phase from each sample were placed in a 96‐well plate for analysis.

#### 4.6.8 Brain Studies in Mice—In Vivo Treatments

No adverse effects were noted during in vivo treatments (2 mg/kg, i.v.). Mice were sacrificed after 15 min, then the IV bolus and compounds in plasma and brain were quantified. Concentrations in the brain were low, and their quantification could be influenced by blood leftovers in the brain.

### 4.7 Modeling Studies

#### 4.7.1 In Silico Modeling of hASIC3 Three‐Dimensional Model and Ligands Preparation

The trimeric assembly of hASIC3 was reconstructed by an in silico procedure, due to the absence of its experimentally resolved 3D structure. The multicomponent AI system, AlphaFold2, which uses machine learning to accurately predict a protein's 3D structure based solely on its primary amino acid sequence [24], was used to achieve the 3D model of the hASIC3 monomer. To build the trimeric assembly, the experimentally resolved 3D structure of *G. gallus* ASIC1 (PDB ID: 6AVE) [35] was used as a template. In detail, the generated hASIC3 monomeric model was superimposed on each of the three monomers present in the trimeric structure of the chosen *G. gallus* ASIC1. The achieved trimeric assembly was then prepared using the graphical interface of Schrödinger's molecular modeling platform, Maestro v. 14.4.138 [36]. In detail, the module Protein Preparation Workflow [37, 38] included in Maestro was used to add hydrogens, create disulfide bonds and cap the N‐ and C‐terminal residues with the acetyl (ACE) and *N*‐methyl amide (NME) groups, respectively. To properly describe the protonation state of the protein residues and the hydrogen‐bonding networks correctly at neutral pH, their p*K*_a_s were evaluated with the Propka program [39] included in Maestro. Finally, a relaxation procedure was performed by running a restrained minimization only on the initially added hydrogen atoms according to the OPLS4 force field [40]. GMQ, **1a,** and **5** were prepared through the LigPrep [41] module of Maestro, employing the OPLS4 force field. Epik [42, 43] was used to evaluate their p*K*_a_ in order to properly describe the protonation state at neutral pH.

#### 4.7.2 Molecular Docking Simulations

Molecular docking simulations were performed by using the Glide SP module [26, 44, 45] included in Maestro. The receptor grid was generated using the Receptor Grid Generation module and was centered in a spatial region that has been shown to represent a binding region for GMQ [27]. In detail, it was centered on two key residues of this region, that is, Glu421_A_ and Glu421_C_ and a receptor grid of 16 Å × 16 Å × 16 Å was generated. Docking simulations with Glide were performed using default parameters. Sampling of nitrogen atom inversions (when not belonging to cycles) and of different ring conformations were allowed, while nonplanar amide conformations were penalized. The receptor was kept fixed, the ligands were treated as flexible, and no other constraints were added. The azine tautomer was used in all computational studies for GHs **1a** and **5**, in accordance with several computational (quantum mechanical) and experimental studies demonstrating a 4–6 kcal/mol stabilization of the azine relative to the hydrazone tautomer [46, 47]. Regarding the protonation state, p*K*_a_ values were predicted using MolGpKa [48], PROPKA [49] and Rowan [50]. The three methods consistently predicted p*K*_a_ values in the ranges of 7.8–8.2 for GH **1a** and 7.6–8.0 for GH **5**. These results indicate that the protonated species is expected to predominate at physiological pH; accordingly, the protonated form was used in all docking calculations.

## Conflicts of Interest

The authors declare no conflicts of interest.

## Supporting information

Synthetic protocols for 2‐GH, 4‐Me quinazolines **1b, 1d–g**, 4‐GH, 2‐Me quinazolines **2a–d, 2f**, quinoline GH **3,** and quinoxaline GH **4**. Analytical characterization (^1^H‐ and ^13^CNMR, HPLC‐MS) for intermediates (^1^H‐ and ^13^C‐NMR) and for target heteroaryl GHs **1a–5** (^1^H‐ and ^13^C‐NMR, LC‐MS). Supporting Scheme 1: Graphichal overview of the synthetic schemes to target heteroaryl GHs **1a–5**. Supporting Figure 1: NOESY spectrum of **1c**. The authors have cited additional references within the Supporting Information [51, 52, 53, 54].

## Data Availability

The data that support the findings of this study are available from the corresponding author upon reasonable request.
