# Supplementary material for: A Blood–Brain Barrier‐Permeable Guanylhydrazone Analogue of the ASIC3 Activator GMQ Inhibits Human Glioblastoma Stem Cell Growth In Vitro
Source: ChemMedChem. 2026 Sep 23;21(18):e70438. doi: 10.1002/cmdc.70438 (PMC13600949; doi:10.1002/cmdc.70438)
Supplement: Supplementary file 1 [file CMDC-21-e70438-s001.pdf]

A BBB-permeable Guanylhydrazone Analogue of the ASIC3 activator GMQ Inhibits Human Glioblastoma Stem Cell Growth In Vitro

Leonardo Maiorana,[1,§] Nicoletta Collura,[2,§] Andrea Gotti,[1] Giulia De Leonardis,[2] Greta Donati,[3] Alice Maiocchi,[1,£] Luciana Marinelli,[3,*] Andrea Menegon,[2,*] Pierfausto Seneci[1,*]

[1] Chemistry Department, Università degli Studi di Milano, Via Golgi 19, 20133 Milan, Italy.

[2] Experimental Imaging Centre, San Raffaele Scientific Institute, Via Olgettina 58, 20132 Milan, Italy.

[3] Department of Pharmacy, Università degli Studi di Napoli Federico II, via D. Montesano 49, 80131, Napoli, Italy.

[£] Present address: Olon SpA, Via B. Cellini 20, 20090 Segrate (MI), Italy.

[§] These authors have equally contributed to the present work.

* Corresponding authors.

SUPPLEMENTARY INFORMATION

Synthetic chemistry 2

Analytical chemistry 87

SYNTHETIC CHEMISTRY

GENERAL PROCEDURES

Synthesis and characterization

Oven-dried glassware was used to carry out chemical reactions, and dry solvents under a nitrogen atmosphere were employed. Solvents were purchased from Sigma Aldrich and used as such. Chemical reagents were purchased from Sigma Aldrich, Fluorochem, BLD, TCI. Purification of intermediates and final products was carried out by flash chromatography using high purity grade silica gel (Merck Grade, pore size 60 Å, 230–400 mesh particle size, Sigma- Aldrich, Milan, Italy) as a stationary phase. Alternatively, purification was performed by a BIOTAGE® system using Biotage Sfar Duo cartridges (4, 10 or 25 g) for direct phase chromatography or Biotage Sfar Duo C_{18} cartridges (6, 12 or 30 g) for reverse phase chromatography. Reaction monitoring by thin layer chromatography (TLC) entailed Merck- precoated $60F_{254}$ plates, using UV light at 254 nm as a direct detection method, or by charring either with a phosphomolybdic acid ethanolic solution, with a potassium permanganate or a ninhydrin solution. ^{1}H NMR and ^{13}C NMR spectra were recorded in $CDCl_3$, CD_3OD, or DMSO-*d*$_6$, depending on compounds' solubility, on Bruker DRX-400 instruments. Chemical shifts (δ) for proton and carbon signals are quoted relatively to the solvent's peak and expressed in parts per million (ppm). Ultra-high performance liquid chromatography/mass spectrometry analysis (UPLC/MS) was performed using an Acquity® UPLC/MS System equipped with a tunable ultraviolet (TUV) detector, a single quadrupole (SQD) mass spectrometer and ACQUITY UPLC BEH SHIELD RP_{18} columns (2.1 ×100 mm, id =1.7 μm).

Purity statement

Reaction intermediates were characterized by ^{1}H-NMR and MS. Final synthetic targets were submitted to a more complete characterization, including ^{13}C-, COSY, HSQC and HMBC NMR, and LC-MS; such spectra, provided in the Supplementary Information, generally indicate a good purity. In particular, GMQ-like 2-GH quinazolines (**1a**-**g**), isomeric GMQ-like 4-GH quinazolines (**2a**-**f**) and scaffold hopping-derived 2-GH heterocycles from scaffold hopping (**3**-5**)** confirmed by LC-MS a ≥ 90% purity suitable for biology profiling.

The **general procedures A-E** shown in Scheme S1 and described in the main text were employed for the synthesis of GMQ-like 2-GH quinazolines **1a**–**g**, isomeric GMQ-like 4-GH quinazolines **2a**–**f** and GMQ-like 2-GHs **3-5** from scaffold hopping.

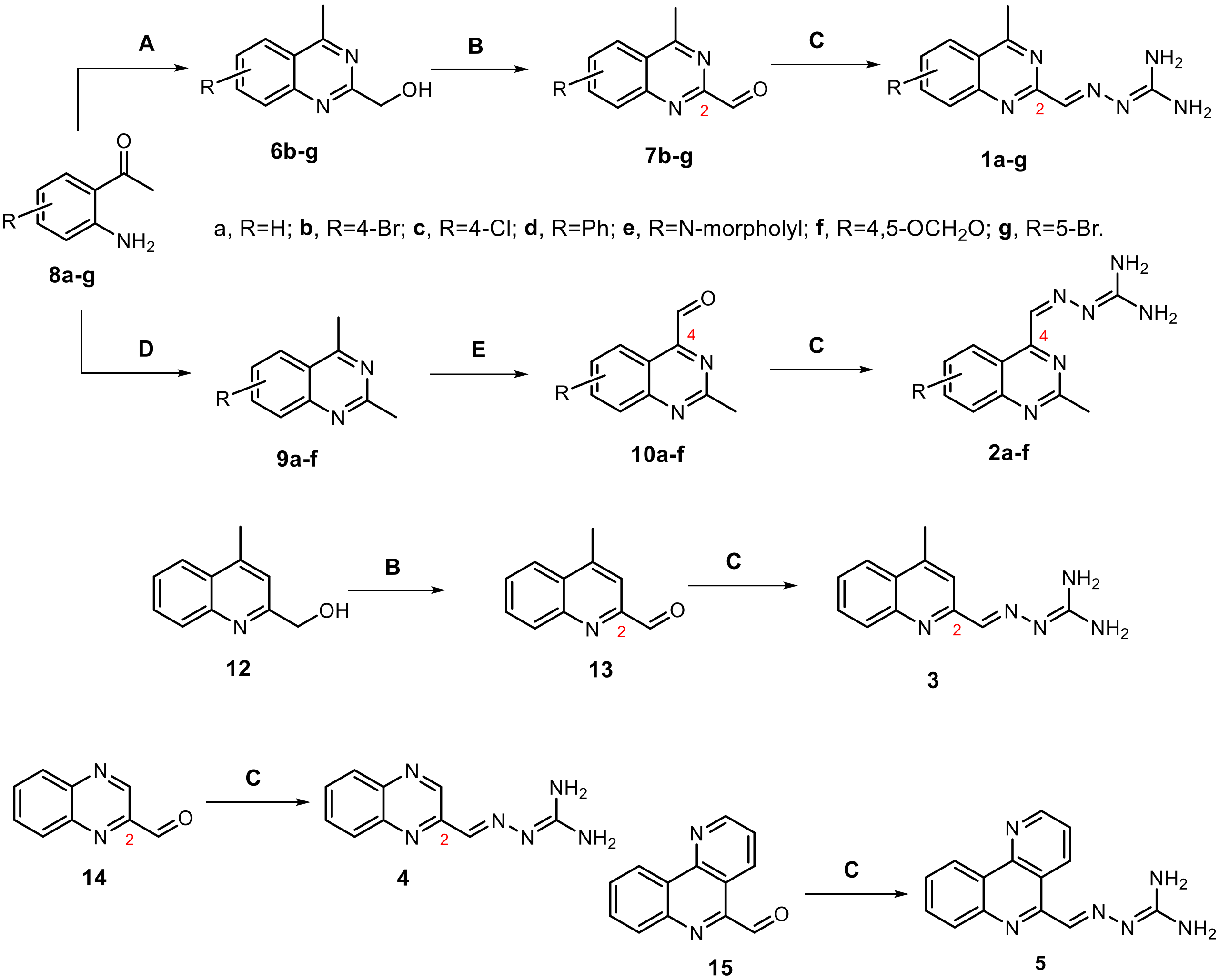

Scheme S1 –General procedures A-E employed for the synthesis of GH-containing analogues of GMQ.

SYNTHESIS

Synthesis of substituted *o*-aminoacetophenones 8c, 8d and 8e

Substituted o-aminoacetophenones **8a, 8b, 8f** and **8g** were purchased from BLD. **8c** and **8e** were synthetized in our laboratories starting from commercial 1-(5-chloro-2-nitrophenyl)-ethan-1-one in a one- or two-step procedure, respectively. Substituted o-aminoacetophenone **8d** was directly obtained from **8b** via Suzuki coupling.

Synthesis of 1-(2-amino-5-chlorophenyl)ethan-1-one 8c

Solid Fe (1.2 g, 20 mmol, 10 eq) and a sat. aqueous NH_4Cl solution (2.6 mL) were added to a stirred suspension of 1-(5-chloro-2-nitrophenyl)-ethan-1-one (0.40 g, 2 mmol, 1 eq) in EtOH (10 mL). The mixture was stirred at reflux for 3 hours, monitoring by TLC (eluent mixture: 8:2 n-Hex/EtOAc), then it was cooled to rt and filtered over a Celite pad, washing with EtOAc (125 mL). The solvent was evaporated under reduced pressure and the crude was purified *via* direct phase flash column chromatography (eluent mixture: 8:2 *n*-Hex/EtOAc), to obtain pure 1-(2-amino-5-chlorophenyl)ethan-1-one **8c** as a yellow solid (329 mg, 1.94 mmol, 97% yield).

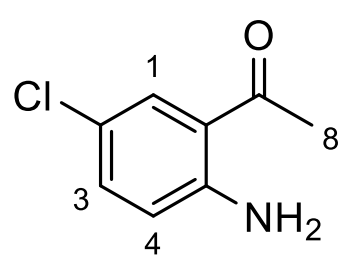

^{1}H-NMR (400 MHz, $CDCl_3$) δ 7.66 (d, *J* = 2.4 Hz, 1H, H1), 7.20 (dd, *J* = 8.8, 2.5 Hz, 1H, H3), 6.60 (d, *J* = 8.8 Hz, 1H, H4), 2.56 (s, 3H, H8). Spectroscopic data are consistent with the literature [51].

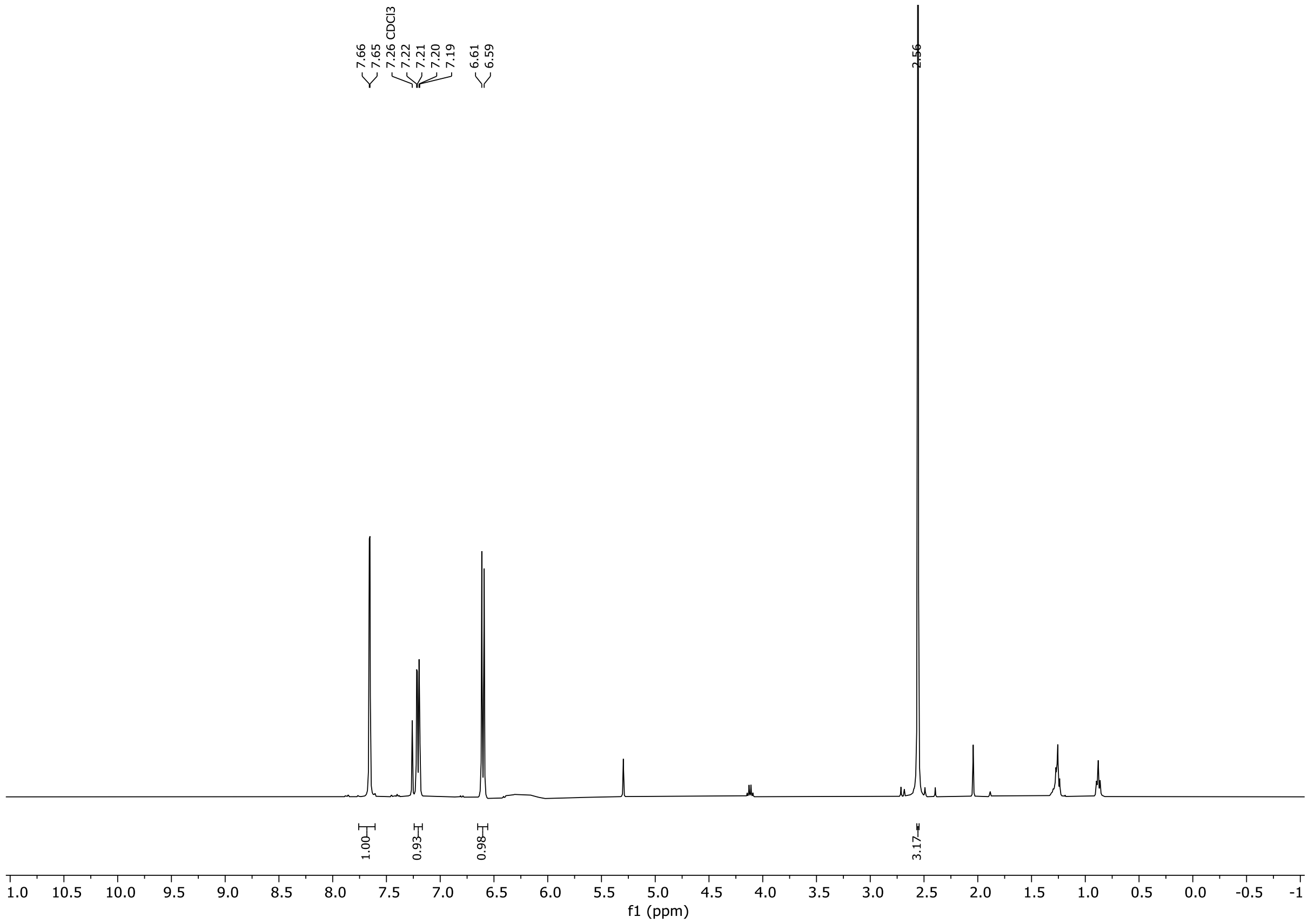

Synthesis of 1-(4-amino-[1,1'-biphenyl]-3-yl)ethan-1-one 8d

1-(2-Amino-5-bromophenyl)-ethan-1-one **8b** (400 mg, 1.87 mmol, 1 eq.), phenylboronic acid (251 mg, 2.06 mmol, 1.1 eq.), oven-dried K_2CO_3 (775 mg, 5.61 mmol, 3 eq.) and $Pd(PPh_3)_4$ (108 mg, 0.09 mmol, 0.05 eq.) were sequentially added to a round bottom flask under nitrogen atmosphere. 1:1 1,4-Dioxane/H_2O (7.4 mL) was added, and the resulting mixture was stirred at 100 °C for 3 hours, monitoring by TLC (eluent mixture: 95:5 *n*-Hex/EtOAc). The reaction mixture was poured into EtOAc (15 mL) and the layers were separated. The organic phase was washed with 1M HCl (15 mL) and brine (15 mL), dried over Na_2SO_4 and concentrated under reduced pressure. The crude was purified *via* BIOTAGE® direct phase flash column chromatography (eluent mixture from 3% to 30% EtOAc in *n*-Hex), obtaining pure 1-(4-amino-[1,1'-biphenyl]-3-yl)ethan-1-one **8d** as a yellow solid (136 mg, 0.61 mmol, 33% yield).

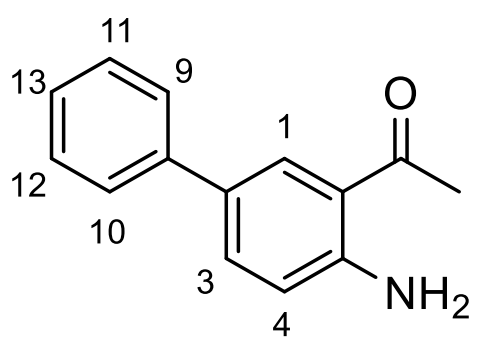

^1H NMR (400 MHz, $CDCl_3$) δ 7.96 (s, 1H, H1), 7.60 – 7.54 (m, 3H, H3, H9, H10), 7.46 (t, *J* = 7.5 Hz, 2H, H11, H12), 7.35 (t, *J* = 7.5 Hz, 1H, H13), 7.29 – 7.24 (t, 1H), 6.79 (d, *J* = 8.4 Hz, 1H, H4), 5.94 (s, 2H, $-NH_2$), 2.68 (s, 3H, H8). Spectroscopic data are consistent with the literature [52].

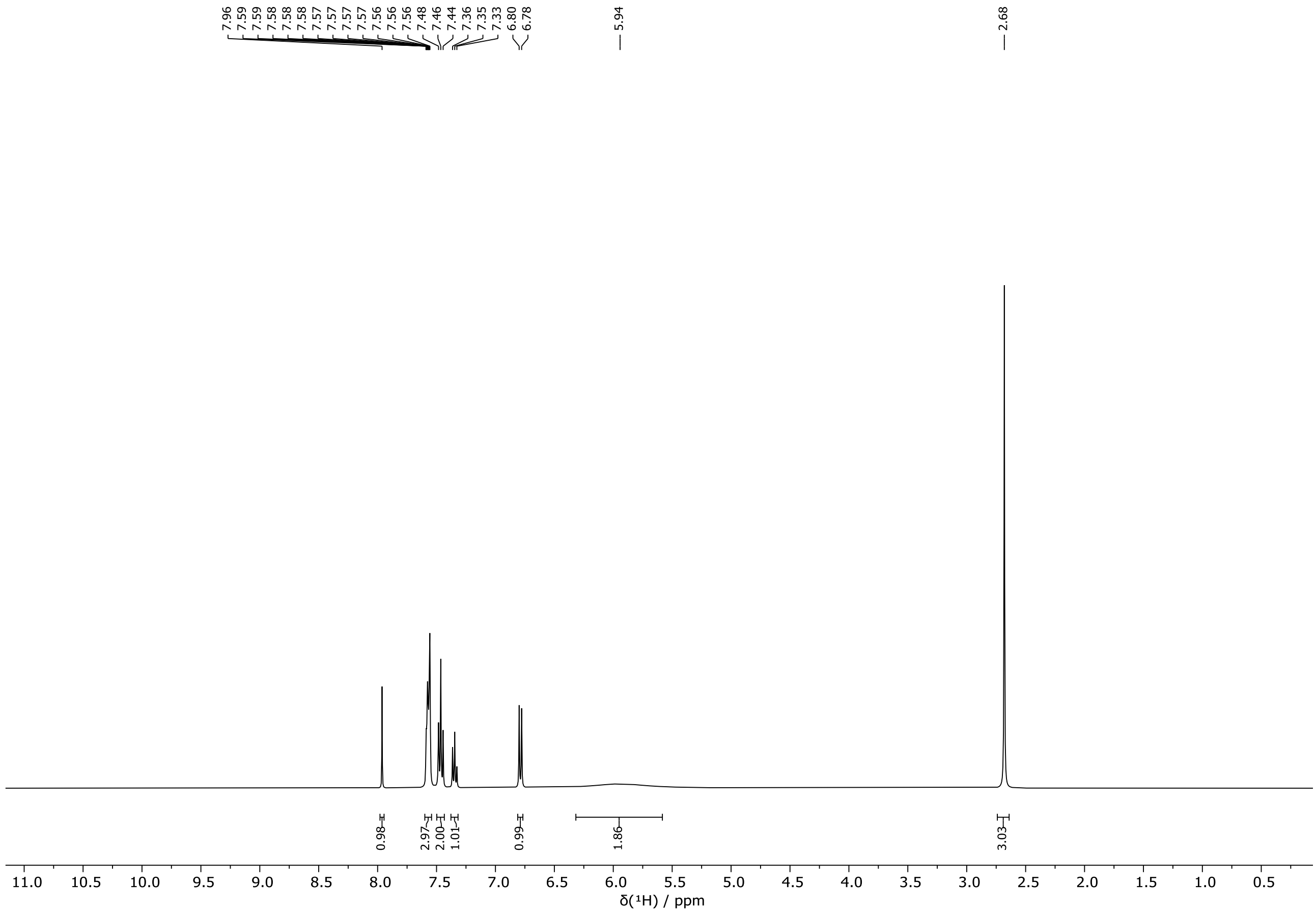

Synthesis of 1-(5-morpholino-2-nitrophenyl)ethan-1-one

1-(5-Chloro-2-nitrophenyl)-ethan-1-one (150 mg, 0.75 mmol, 1.0 eq) was dissolved in dry DMF (1.5 mL) under nitrogen atmosphere. Morpholine (163 mg, 1.90 mmol, 2.5 eq) was added and the solution was stirred at 110 °C for 5 hours, monitoring by TLC (eluent mixture: 3:7 DCM/EtOAc). Then, the mixture was cooled to rt and ice water (2 mL) was added, leading to the precipitation of an orange solid, that was filtered on Hirsch funnel and washed with ice water (10 mL). The obtained crude 1-(5-morpholino-2-nitrophenyl)ethan-1-one was pure enough to be used without any further purification (160 mg, 0.64 mmol, 91% yield).

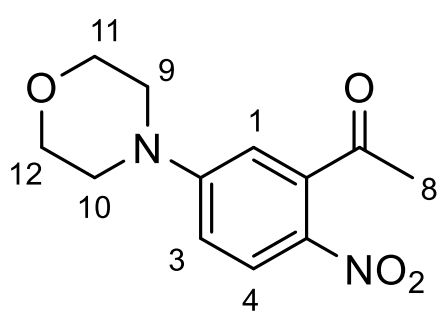

^{1}H-NMR (400 MHz, $CDCl_3$) δ 8.09 (d, *J* = 9.3 Hz, 1H, H4), 6.83 (dd, *J* = 9.3, 2.8 Hz, 1H, H3), 6.58 (d, *J* = 2.8 Hz, 1H, H1), 3.87 – 3.83 (m, 4H, H11, H12), 3.39 (t, *J* = 5.0 Hz, 4H, H9, H10), 2.50 (s, 3H, H8). Spectroscopic data are consistent with the literature [53].

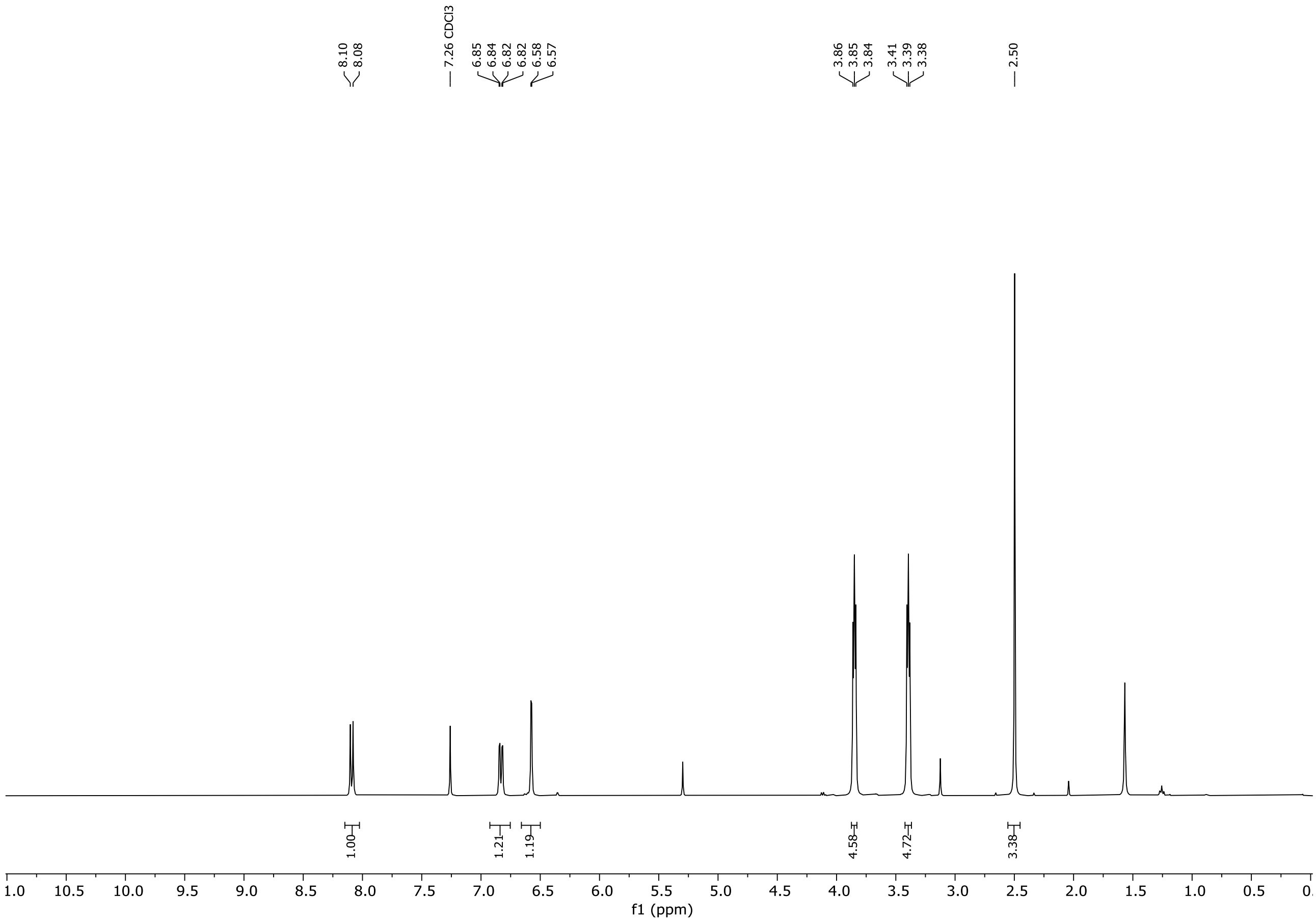

Synthesis of 1-(2-amino-5-morpholinophenyl)ethan-1-one 8e

Solid Fe (313 mg, 5.60 mmol, 10 eq) and a sat. aqueous NH_4Cl solution (0.73 mL) were added to a suspension of 1-(5-chloro-2-nitrophenyl)-ethan-1-one (140 mg, 0.56 mmol, 1 eq) in EtOH (2.8 mL). The mixture was stirred at reflux for 2 hours until the disappearance of the starting material (TLC monitoring: 3:7 DCM/EtOAc), then it was cooled to rt and filtered over a Celite pad, washing with EtOAc (125 mL). The solvent was removed under reduced pressure and the crude was purified *via* direct phase flash column chromatography (eluent mixture: 1:9 *n*-Hex/EtOAc), to obtain pure 1-(2-amino-5-morpholinophenyl)ethan-1-one **8e** as an orange solid (120 mg, 0.54 mmol, 86% yield).

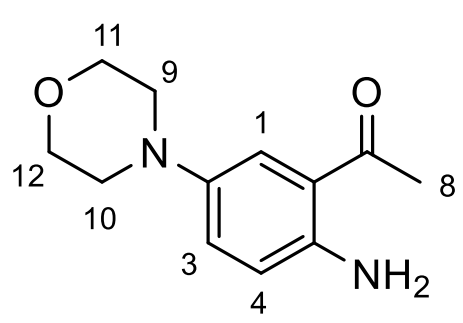

^{1}H-NMR (400 MHz, $CDCl_3$) δ 7.24 (s, 1H, H1), 7.06 (dd, *J* = 8.9, 2.7 Hz, 1H, H3), 6.65 (d, *J* = 8.9 Hz, 1H, H4), 6.03 (s, 2H, NH_2), 3.88 (t, *J* = 4.7 Hz, 4H, H11, H12), 3.02 (t, *J* = 4.7 Hz, 4H, H9, H10), 2.57 (s, 3H, H8). Spectroscopic data are consistent with the literature [54].

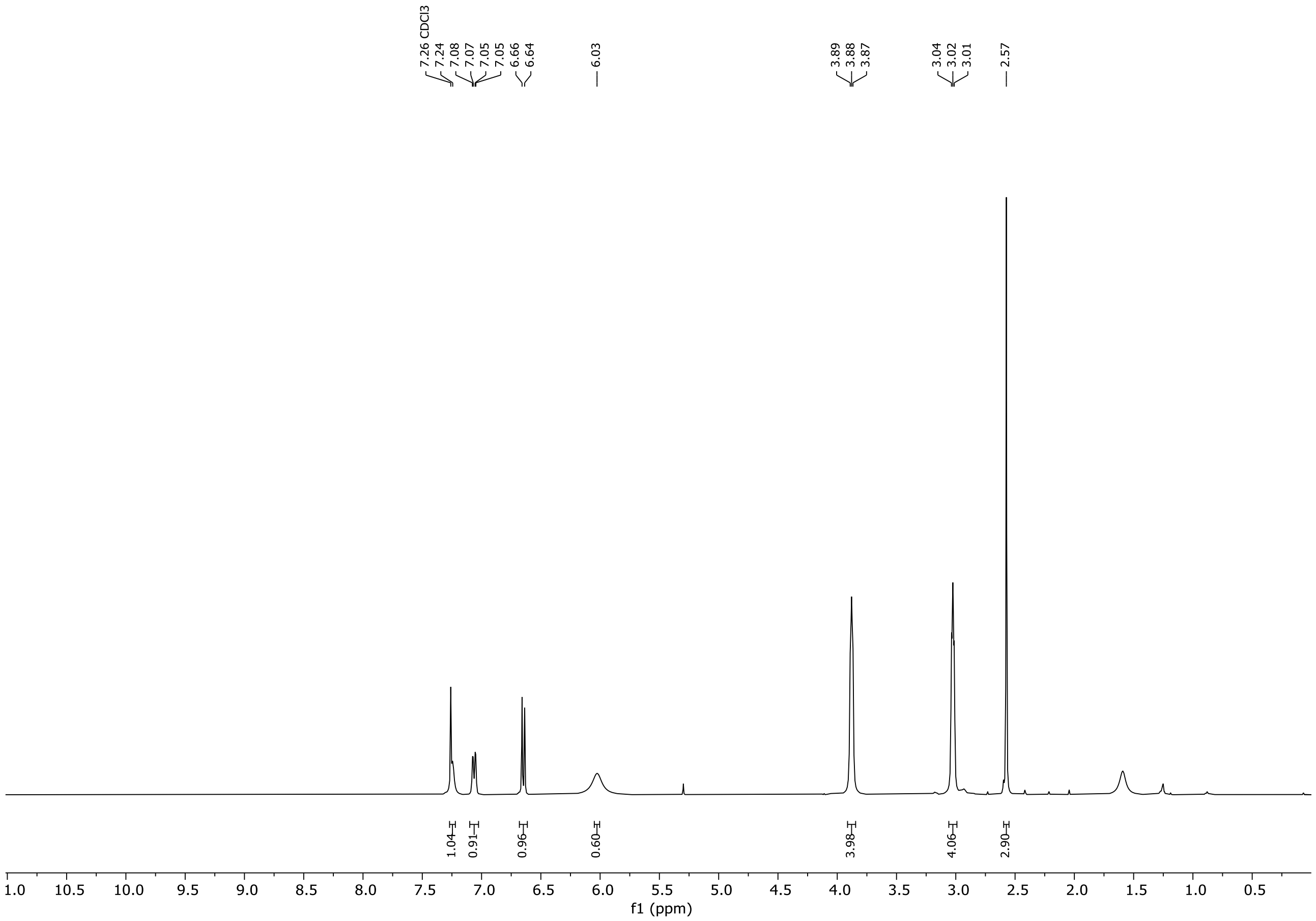

Synthesis of GMQ-like 2-GH quinazolines 1a-g

Synthesis of 2-((4-methylquinazolin-2-yl)methylene)hydrazine-1-carboximidamide hydrochloride 1a

The synthesis of 2-((4-methylquinazolin-2-yl)methylene)hydrazine-1-carboximidamide hydrochloride **1a** is described in the main text. The analytical characterization of intermediate (4-methylquinazolin-2-yl)methanol **6a**, 4-methylquinazoline-2-carbaldehyde **7a** and 2-((4-methylquinazolin-2-yl)methylene)hydrazine-1-carboximidamide hydrochloride **1a** is reported below.

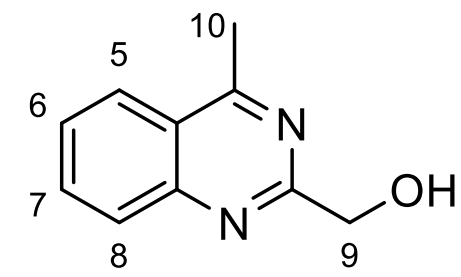

6a

^{1}H-NMR (400 MHz, $CDCl_3$) δ 8.11 (dd, *J* = 8.2, 1.1 Hz, 1H, H5), 8.01 (d, *J* = 8.4 Hz, 1H, H8), 7.90 (ddd, *J* = 8.4, 6.9, 1.1 Hz, 1H, H7), 7.63 (ddd, *J* = 8.2, 6.9, 1.2 Hz, 1H, H6), 4.94 (s, 2H, H9), 4.04 (s, 1H, OH), 2.97 (s, 3H, H10). Spectroscopic data are consistent with the literature [30].

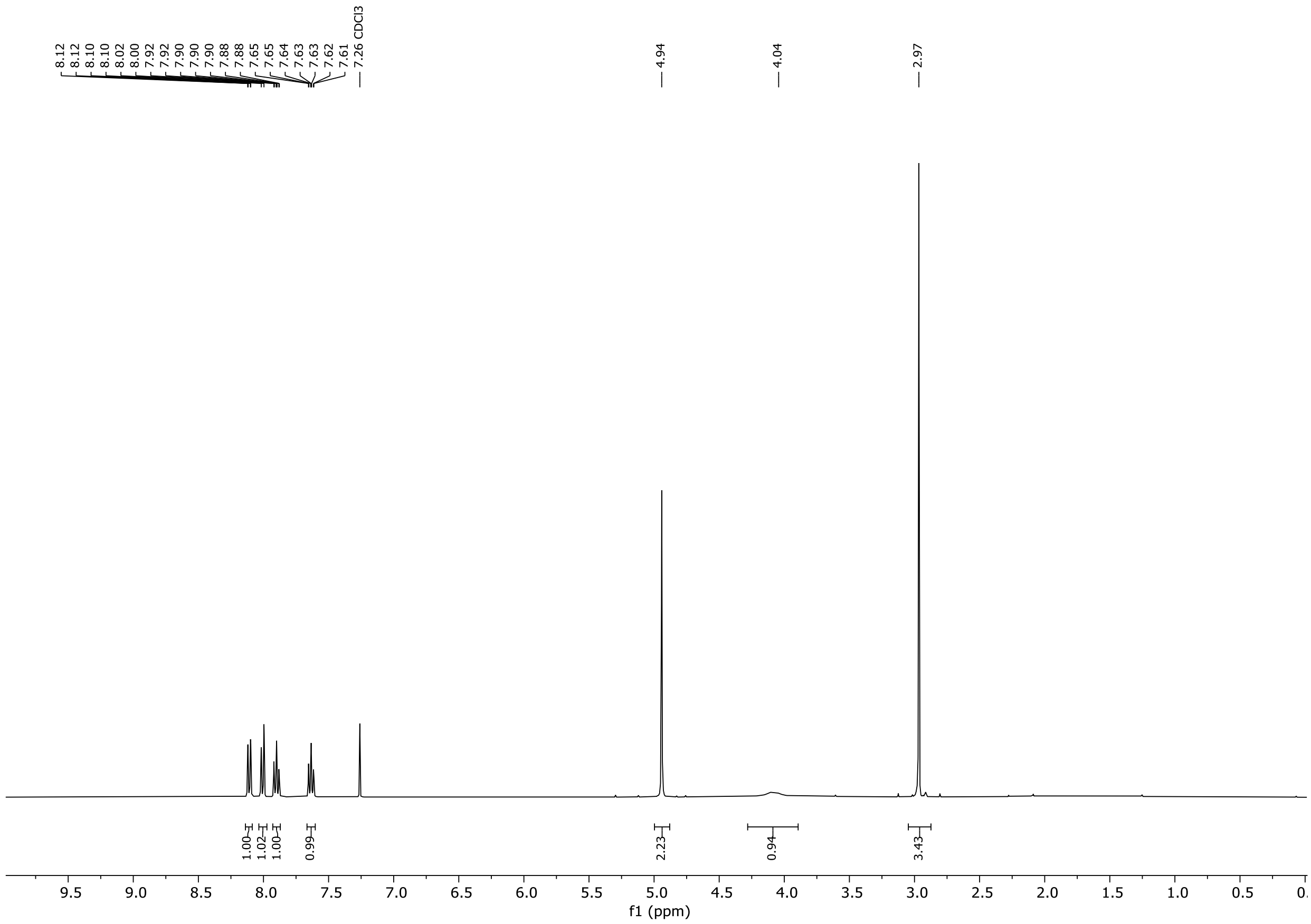

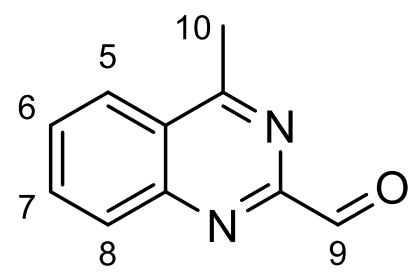

7a

^{1}H-NMR (400 MHz, $CDCl_3$) δ 10.23 (s, 1H, H9), 8.24 (ddd, *J* = 8.4, 1.3, 0.7 Hz, 1H, H5), 8.21 (ddd, *J* = 8.4, 1.4, 0.7 Hz, 1H, H8), 8.02 (ddd, *J* = 8.4, 7.0, 1.4 Hz, 1H, H6), 7.82 (ddd, *J* = 8.4, 6.9, 1.2 Hz, 1H, H7), 3.09 (s, 3H, H10).

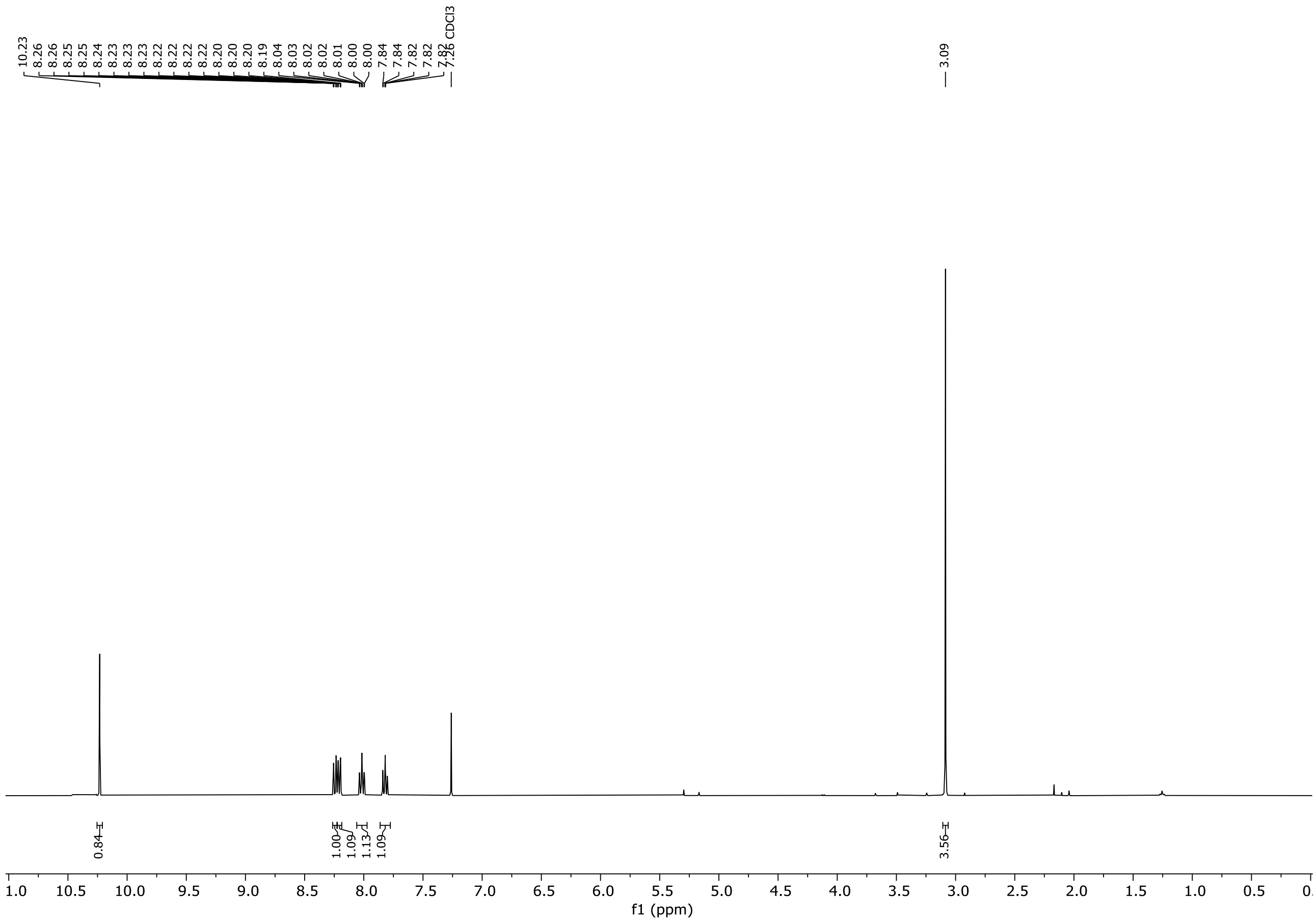

^{13}C-NMR (101 MHz, $CDCl_3$) δ 192.3, 170.3, 155.3, 149.7, 134.7, 130.4, 130.3, 125.4, 125.0, 22.1.

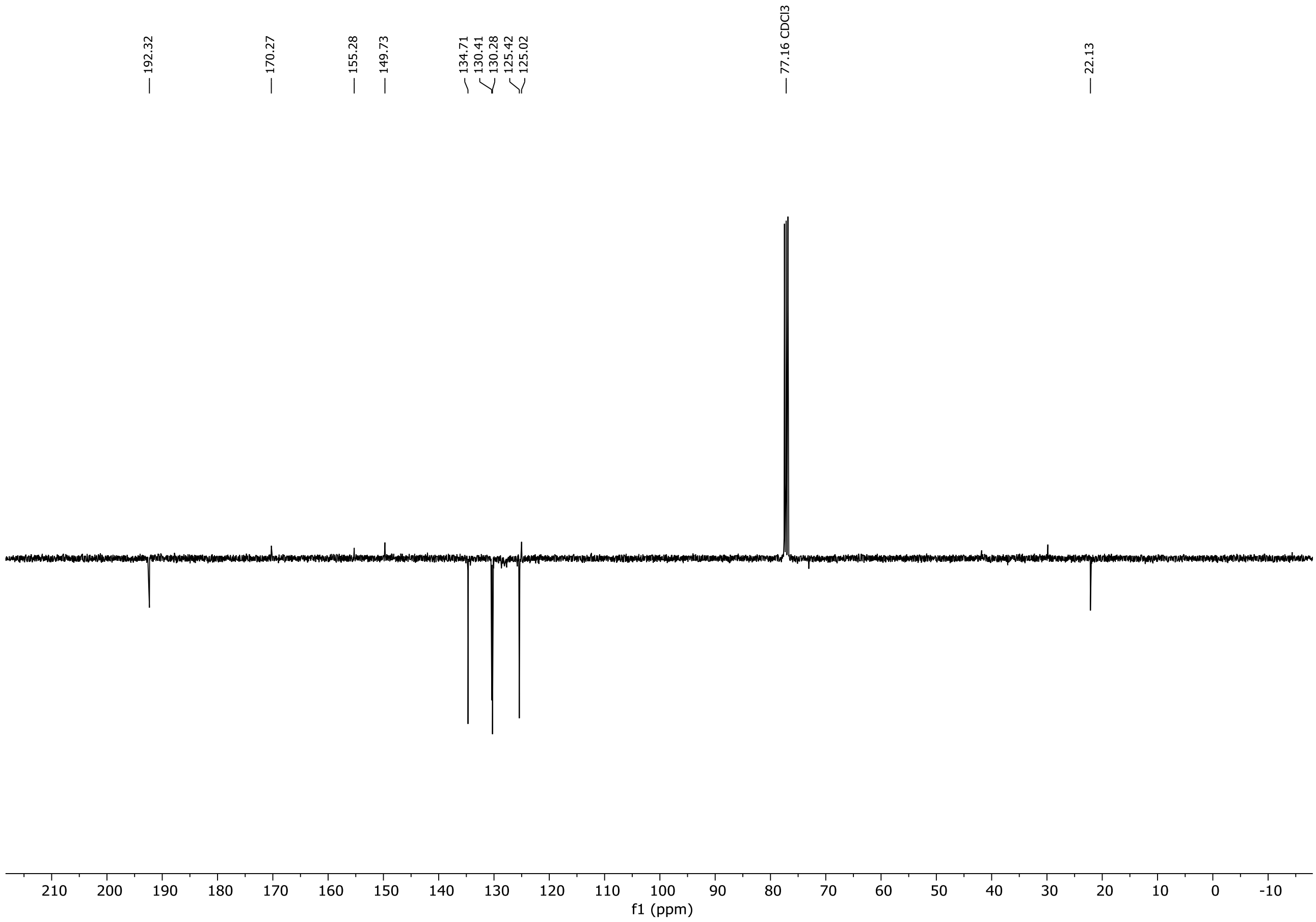

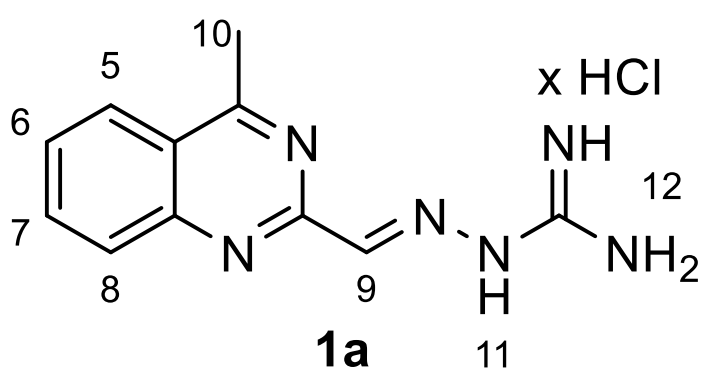

1a

^{1}H-NMR (400 MHz, DMSO-d_6) δ 12.31 (s, 1H, H11), 8.36 (s, 1H, H9), 8.34 (d, J = 8.3 Hz, 2H, H5), 8.08 – 8.02 (m, 2H, H6, H8), 7.85 (brs, 4H, H12), 7.80 (dt, J = 8.2, 3.8 Hz, 1H, H7), 2.98 (s, 3H, H10).

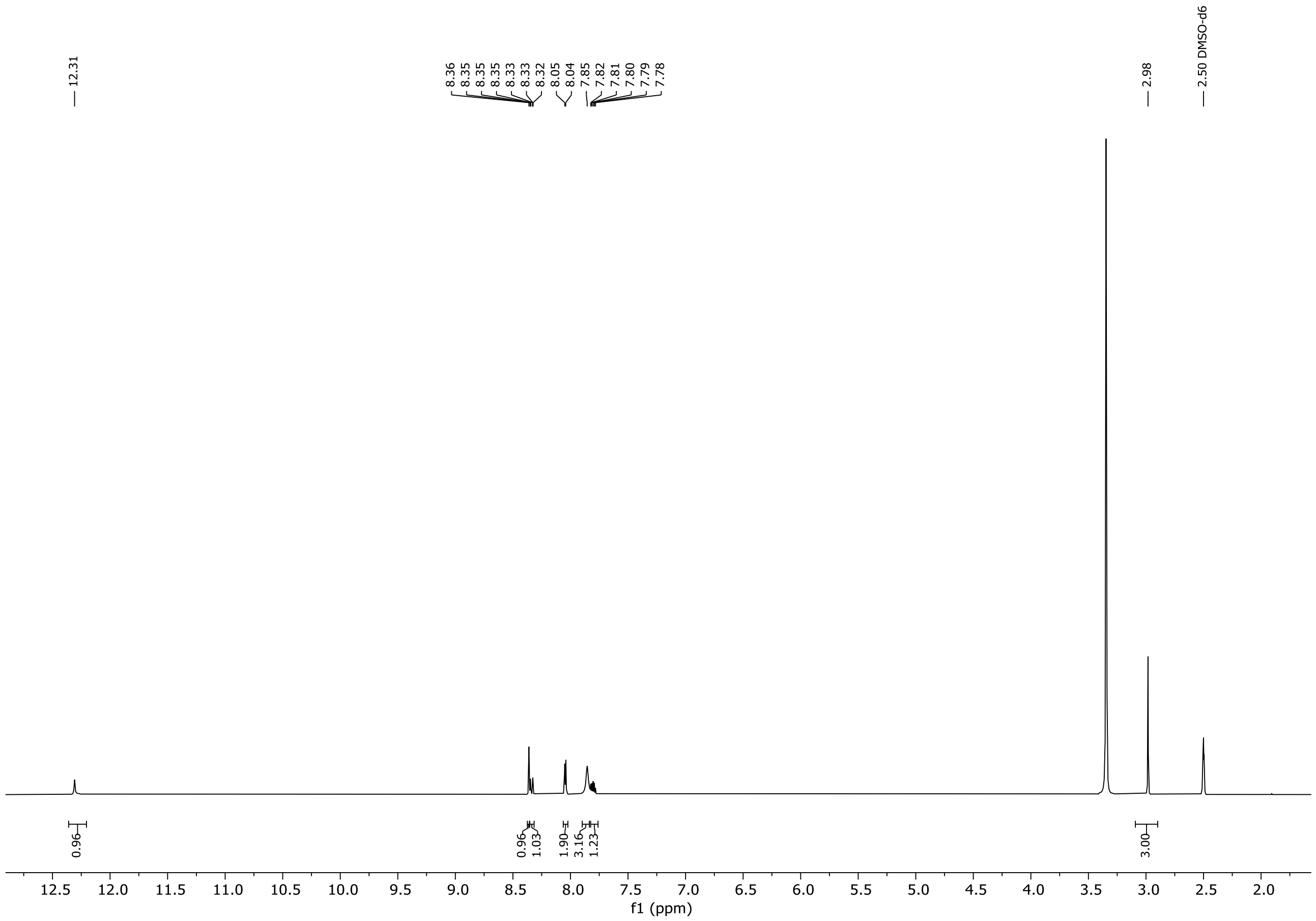

^{13}C-NMR (101 MHz, DMSO-d_6) δ 169.3, 169.3, 155.9, 155.6, 149.1, 146.6, 134.7, 128.7, 128.4, 126.0, 123.0, 21.7, 21.6, 21.6.

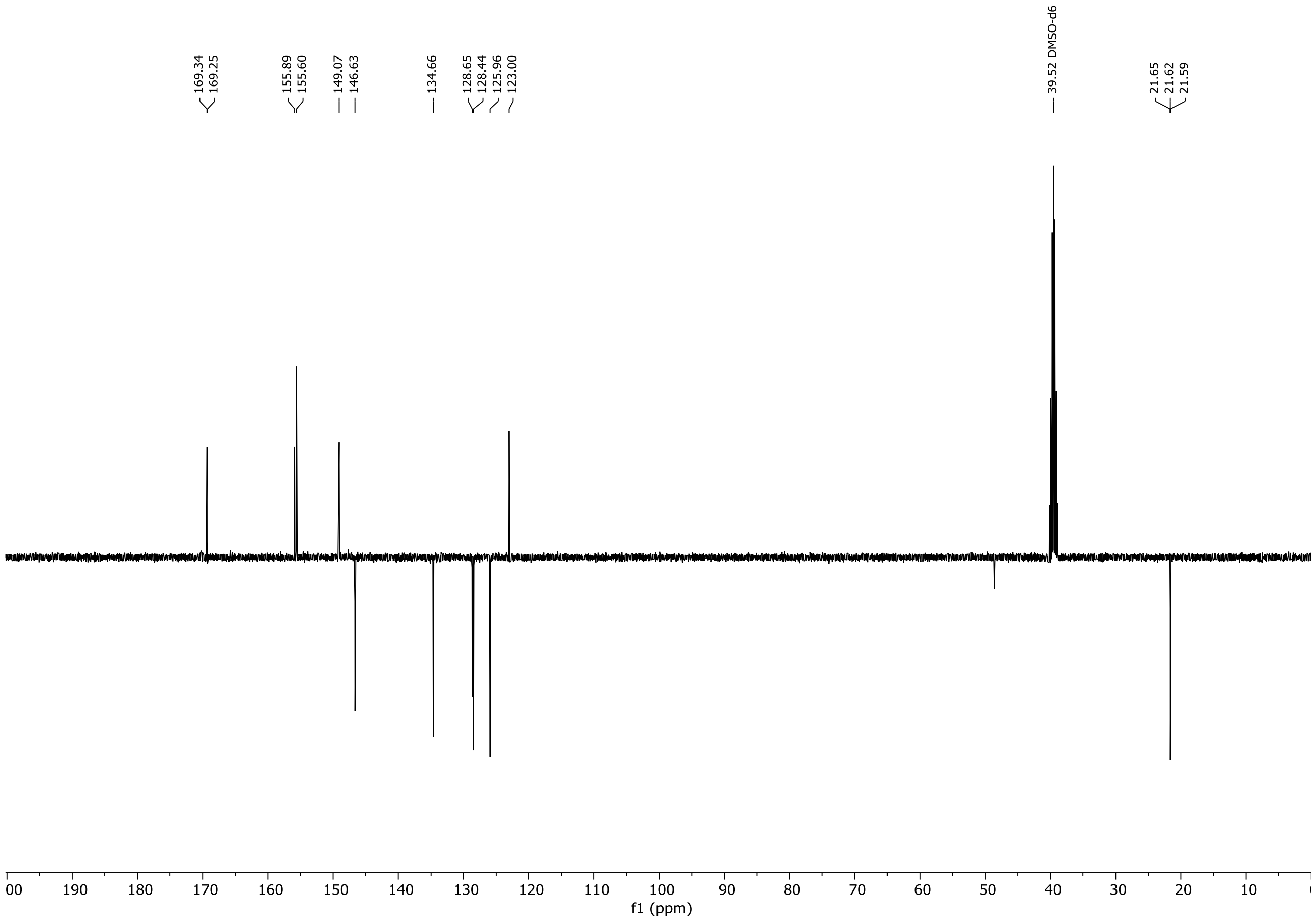

MS (ESI$^+$): m/z [M + H$^+$] 229.03. Calculated MS for $C_{11}H_{13}N_6$: 229.12.

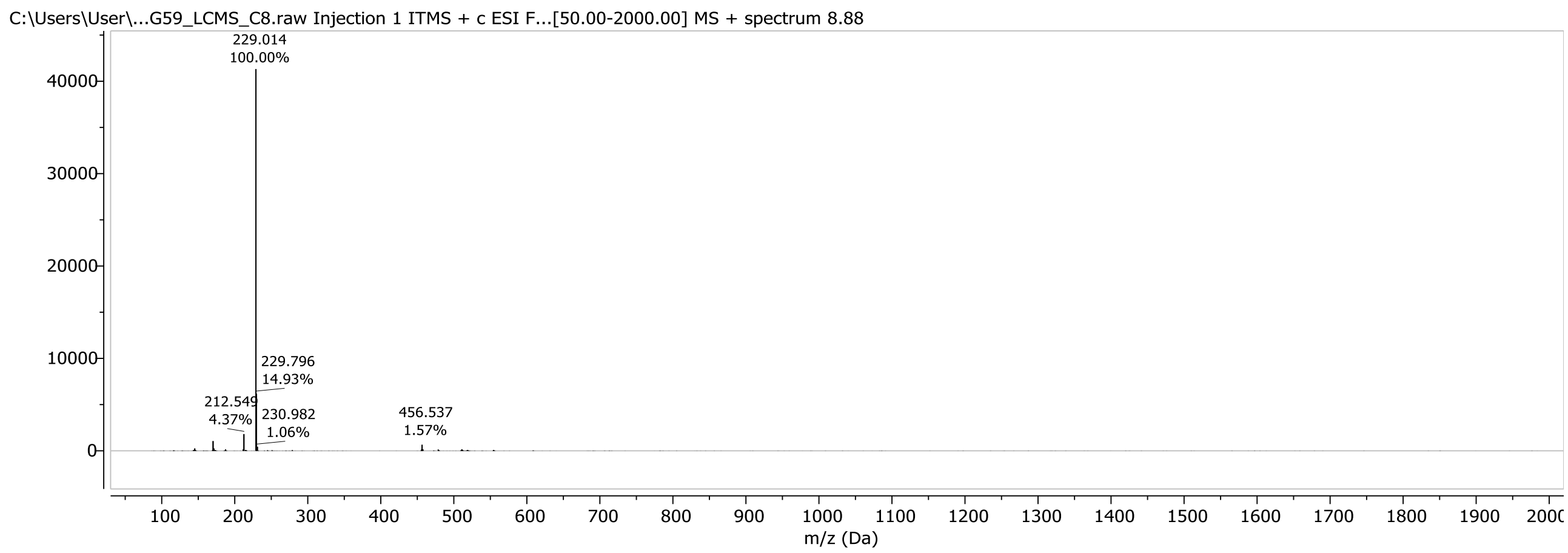

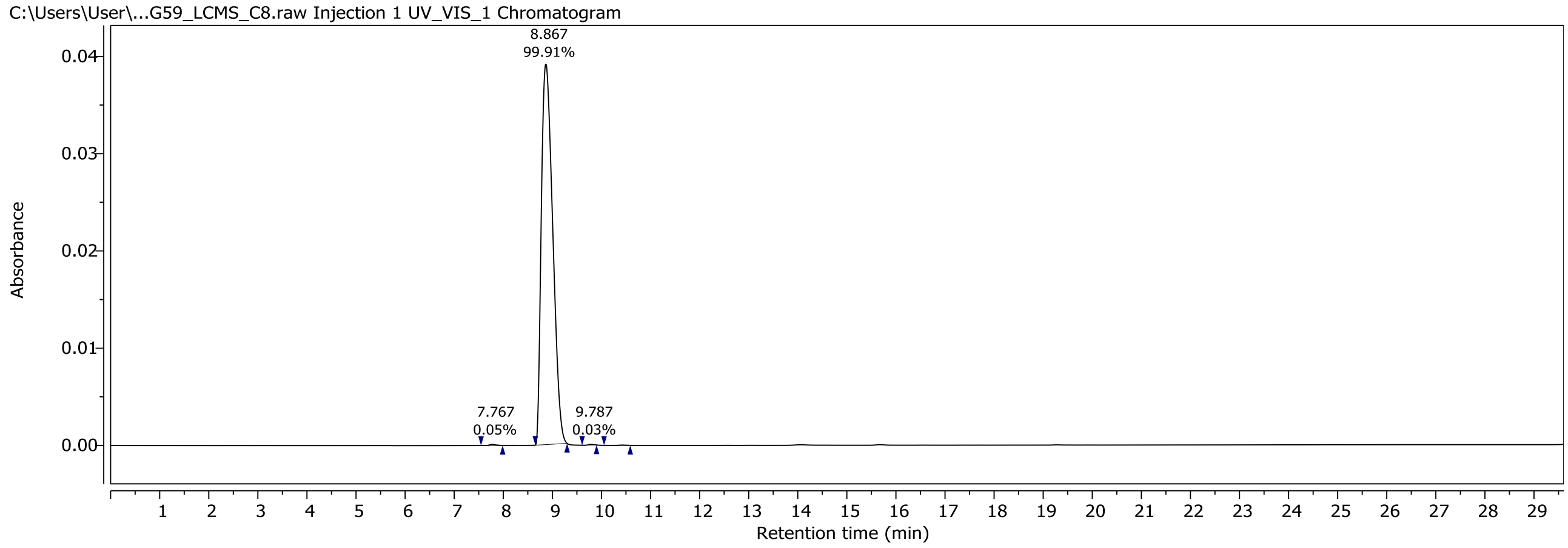

Synthesis of 2-((6-bromo-4-methylquinazolinyl)methylene)hydrazine-1-carboximidamide hydrochloride 1b

Synthesis of 6-bromo-4-methylquinazolin-2-yl)methanol 6b

The reaction was performed according to **general procedure A**, using 1-(2-amino-5-bromophenyl)-ethan-1-one **8b** (107 mg, 0.5 mmol, 1 eq.), triethanolamine (373 mg, 2.5 mmol, 5 eq), eosin Y (32 mg, 0.05 mmol, 0.1 eq.), NH_4Cl (80 mg, 1.5 mmol, 3 eq.) and 1:1 CH_3CN/H_2O (3.3 mL). After purification, pure (6-bromo-4-methylquinazolin-2-yl)methanol **6b** was obtained as a white solid (34 mg, 0.13 mmol, 26% yield).

Reaction time: 24 hours.

TLC monitoring: eluent mixture 7:3 *n*-Hex/EtOAc.

BIOTAGE® Direct phase flash column chromatography: eluent mixture from 6% to 60% EtOAc in *n*-Hex.

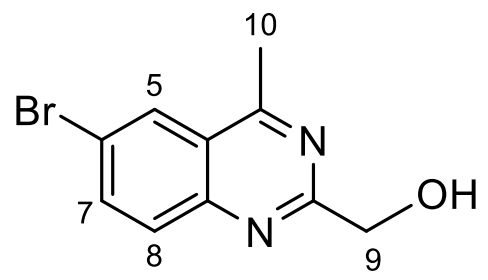

^{1}H NMR (400 MHz, $CDCl_3$) δ 8.24 (d, J = 2.3 Hz, 1H, H5), 7.95 (dd, J = 8.8, 2.3 Hz, 1H, H7), 7.87 (d, J = 8.9 Hz, 1H, H8), 4.92 (s, 2H, H9), 3.68 (s, 1H, OH), 2.92 (s, 3H, H10).

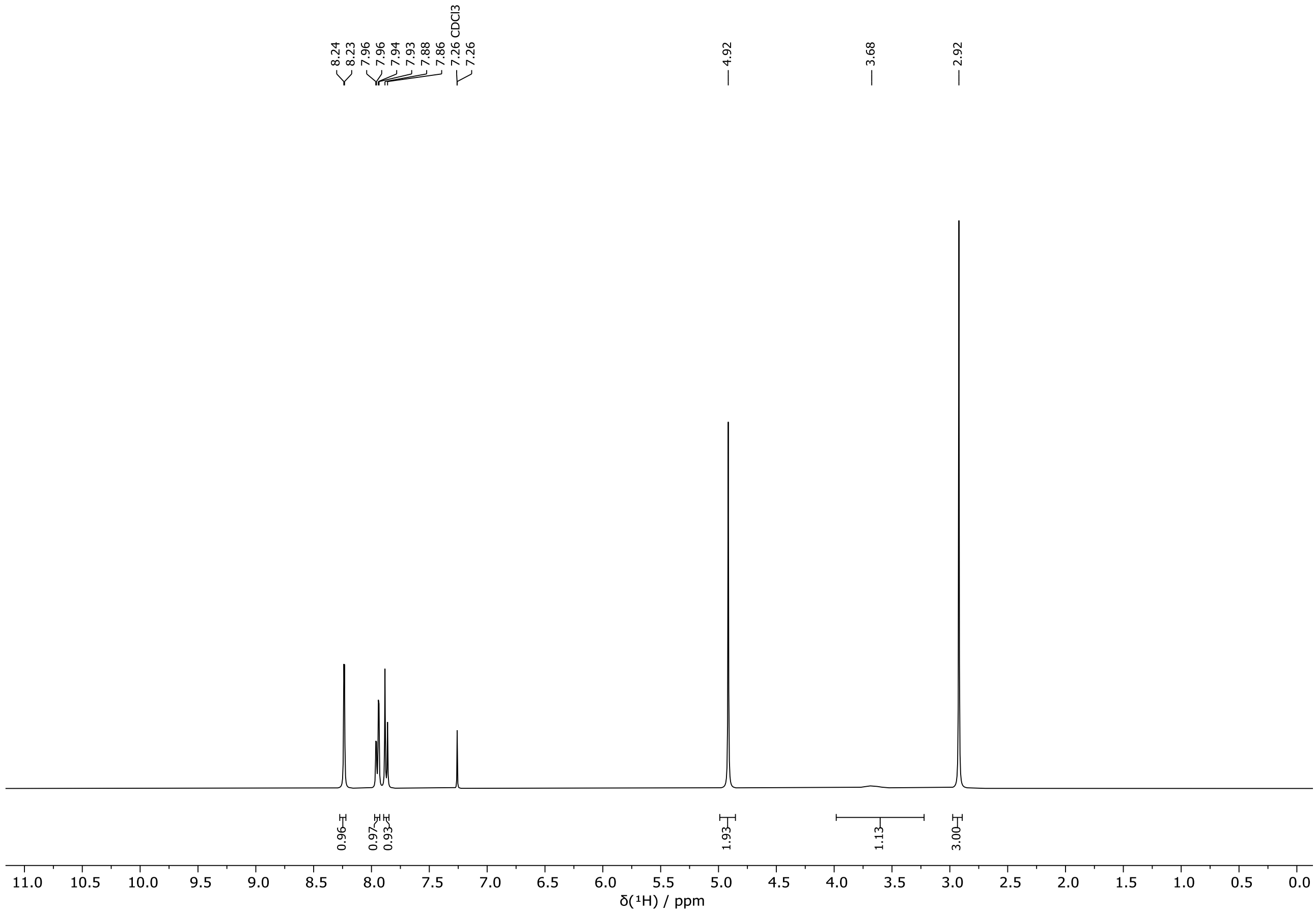

Synthesis of 6-bromo-methylquinazoline 2-carbaldehyde 7b

The reaction was performed according to **general procedure B**, using (6-bromo-4-methylquinazolin-2-yl)methanol **6b** (49 mg, 0.19 mmol, 1.0 eq), DMP (107 mg, 0.25 mmol, 1.3 eq.) and dry DCM (2.0 mL). After purification, pure 6-bromo-methylquinazoline 2-carbaldehyde **7b** was obtained as a white solid (18 mg, 0.07 mmol, 36% yield).

Reaction time: 1 hour.

TLC monitoring: eluent mixture 4:6 *n*-Hex/EtOAc.

BIOTAGE® Direct phase flash column chromatography: eluent mixture from 15% to 100% EtOAc in *n*-Hex.

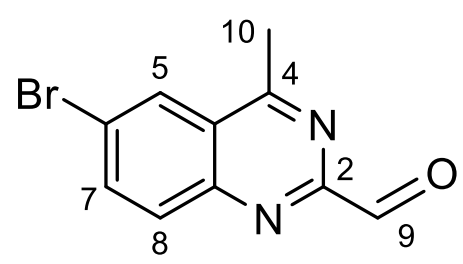

^{1}H NMR (400 MHz, $CDCl_3$) δ 10.20 (s, 1H, H9), 8.35 (s, 1H, H5), 8.13 – 8.04 (m, 2H, H7, H8), 3.05 (s, 3H, H10).

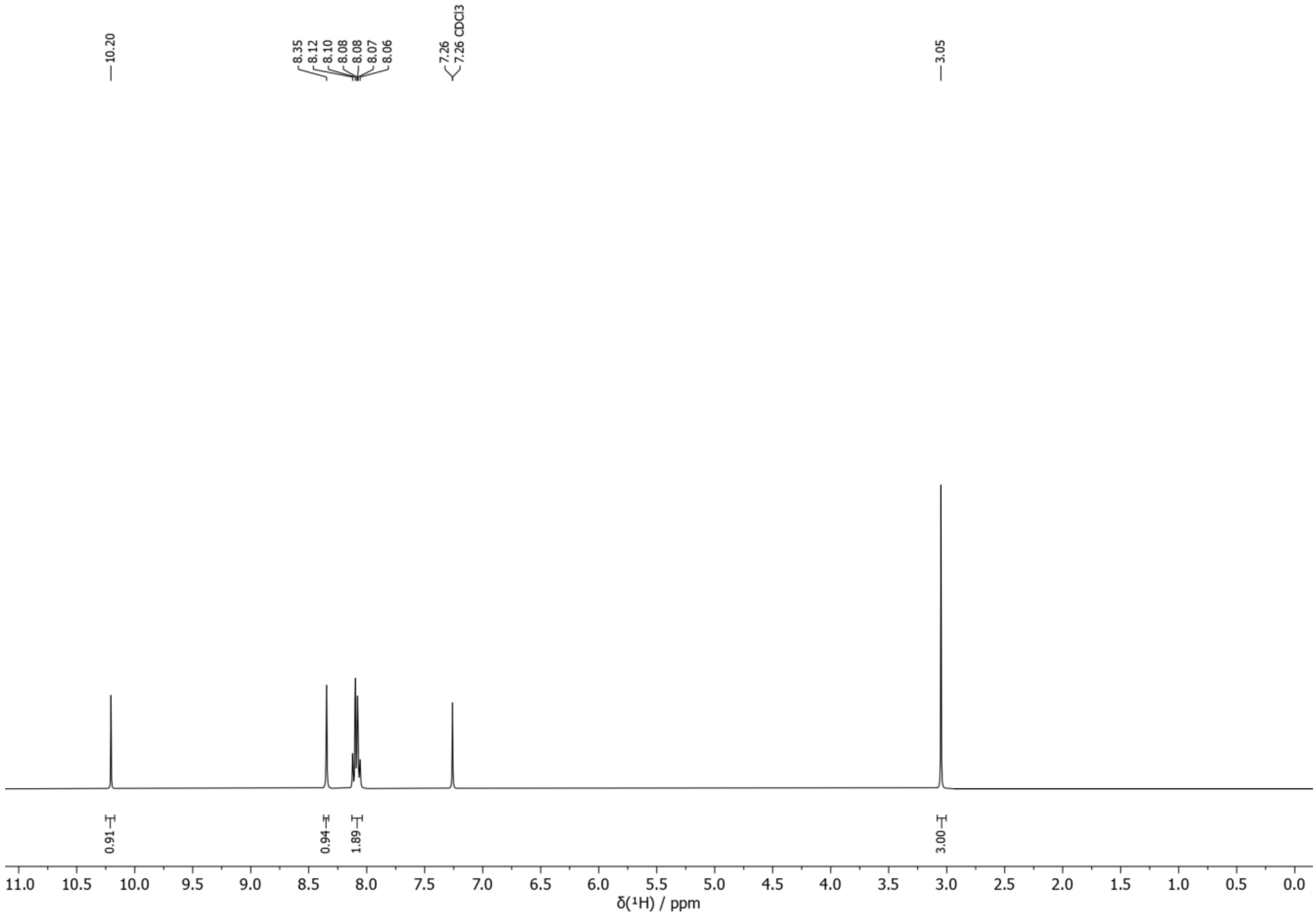

Synthesis of 2-((6-bromo-4-methylquinazolinyl)methylene)hydrazine-1-carboximidamide hydrochloride 1b

The reaction was performed according to **general procedure C**, using 6-bromo-methylquinazoline 2-carbaldehyde **7b** (17 mg, 0.068 mmol, 1 eq.), AG·HCl (7.1 mg, 0.064 mmol, 0.95 eq.) and EtOH (0.7 mL). Trituration was done in (*i*-Pr)$_2$O (3 x 3 mL). Pure target 2-((6-bromomethylquinazolinyl)methylene)hydrazine-1-carboximidamide hydrochloride **1b** was obtained as a white solid (22 mg, 0.064 mmol, quantitative yield) without further purification.

1:0.25 ratio E/Z (80%/20%).

Reaction time: 7 hours, then rt overnight.

TLC monitoring: eluent mixture 1:1 DCM/EtOAc.

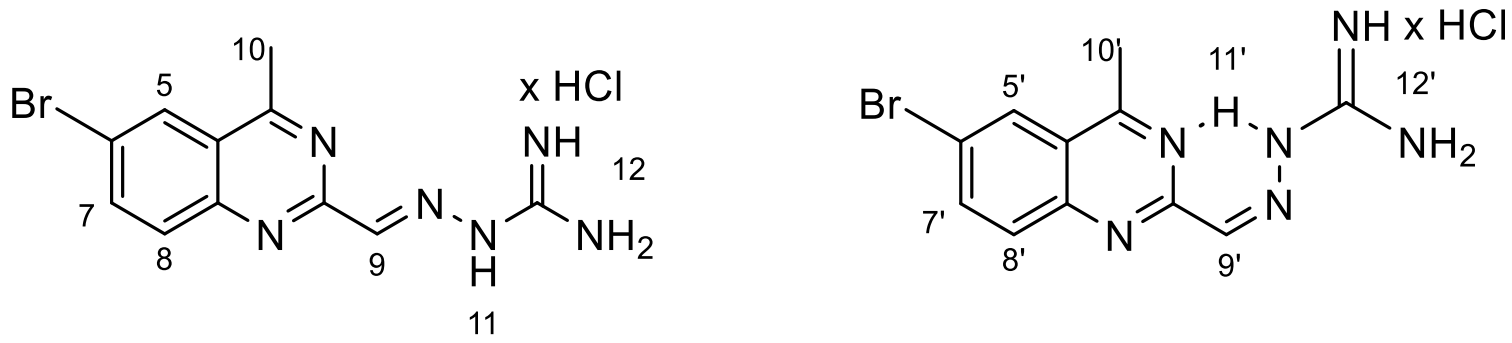

^{1}H NMR (400 MHz, DMSO-*d*$_6$) δ 13.90 (s, 1H, H11’), 12.72 (s, 1H, H11), 8.58-8.53 (m, 5H, H12’, H8’), 8.54 (m, 2H, H5’, H5), 8.36 (s, 1H, H9), 8.25 (dd, *J* = 8.8, 2.2 Hz, 1H, H7’), 8.15 (dd, *J* = 8.8,

2.2 Hz, 1H, H7), 8.02 – 7.88 (m, 5H, H12, H8), 7.73 (s, 1H, H9’), 3.03 (s, 3H, H10’), 2.96 (s, 3H, H10).

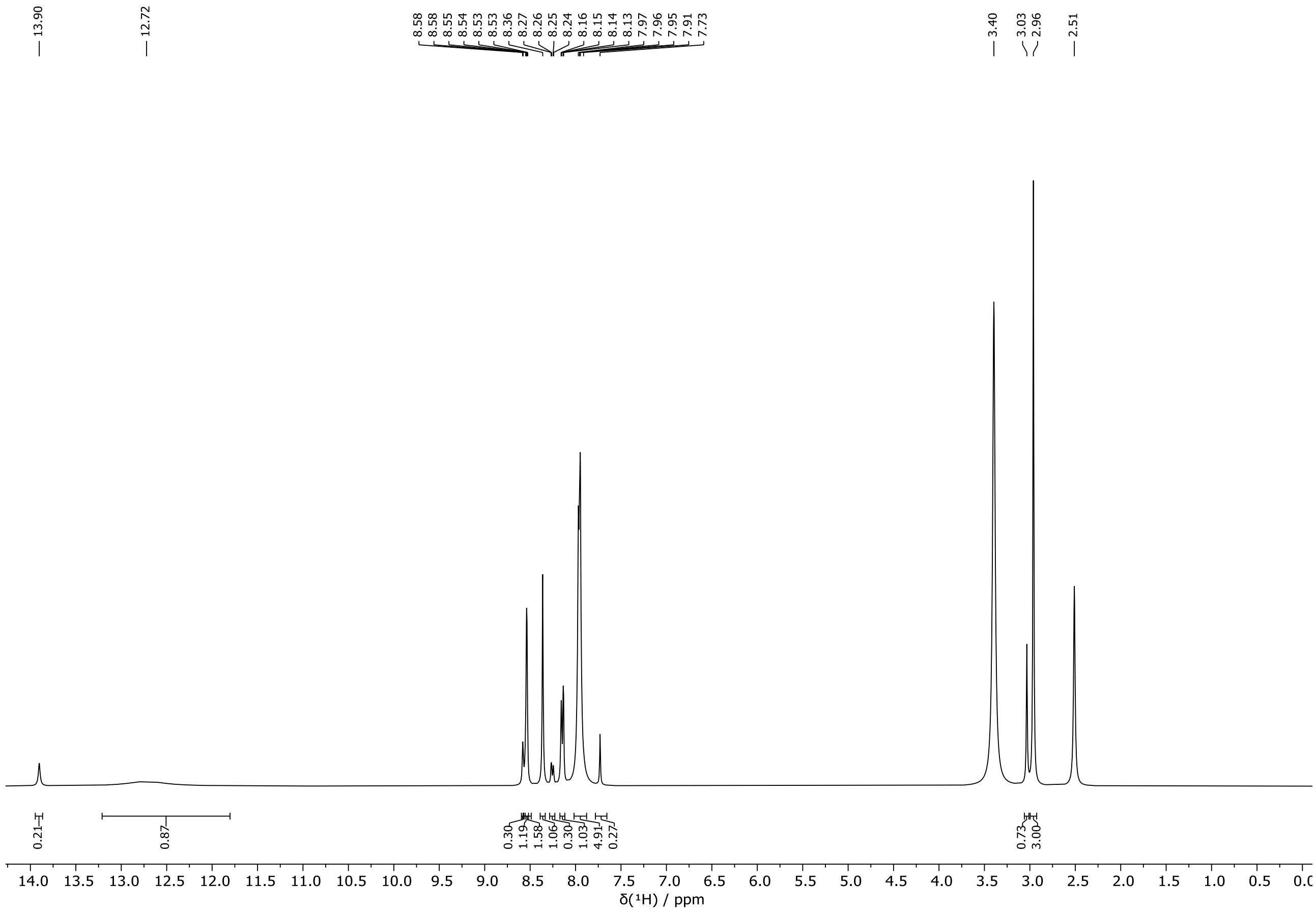

^{13}C NMR (101 MHz, $CDCl_3$) δ 170.46, 169.45, 156.66, 156.34, 156.17, 155.14, 148.35, 147.00, 146.51, 138.76, 138.62, 138.25, 131.53, 131.09, 128.80, 128.72, 124.66, 124.15, 123.14, 121.96, 40.67, 40.46, 40.25, 40.04, 39.83, 39.63, 39.42, 22.28, 22.17.

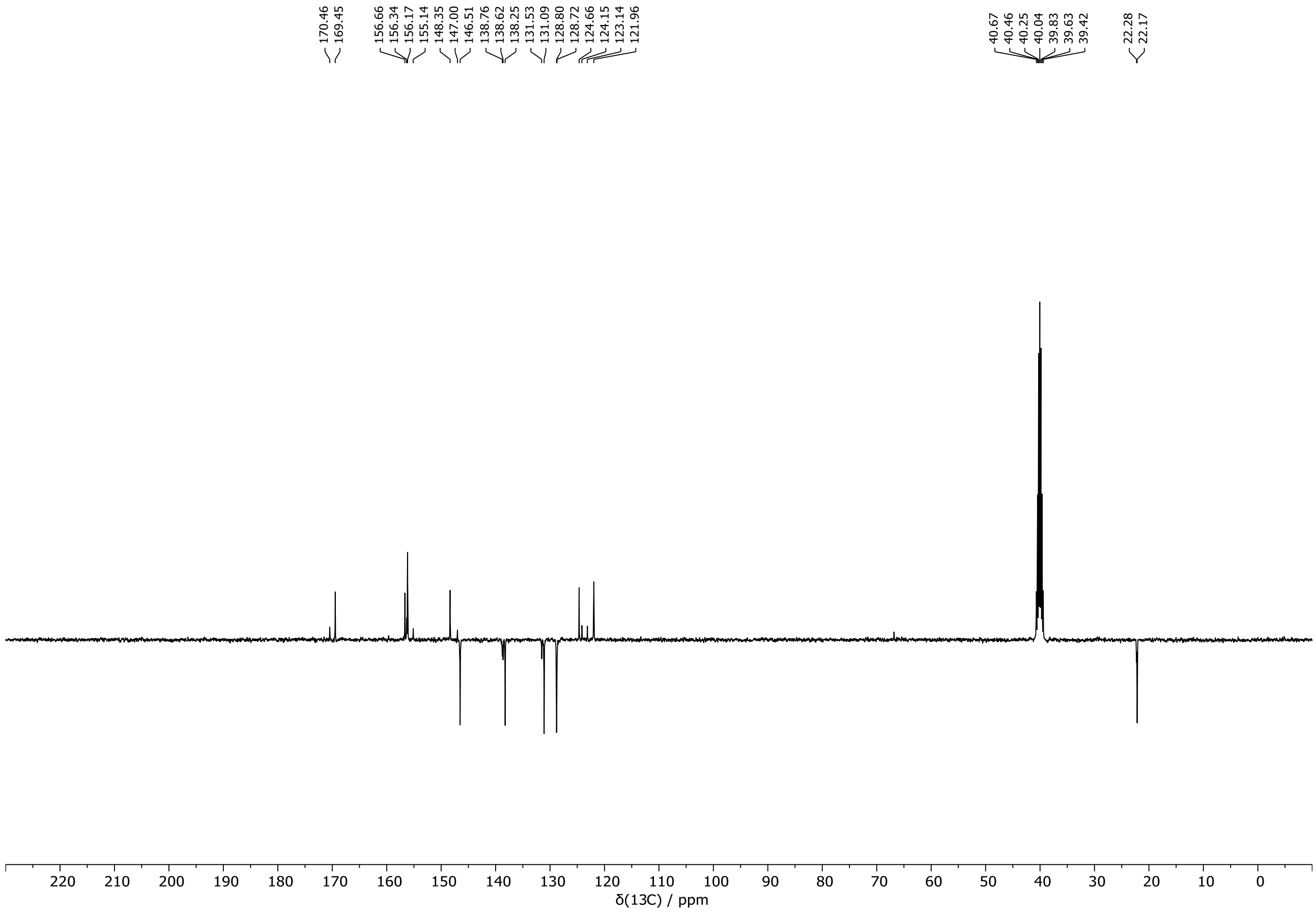

MS (ESI$^+$): m/z [M + H$^+$] 308.88. Calculated MS for $C_{15}H_{20}N_7O$: 308.16.

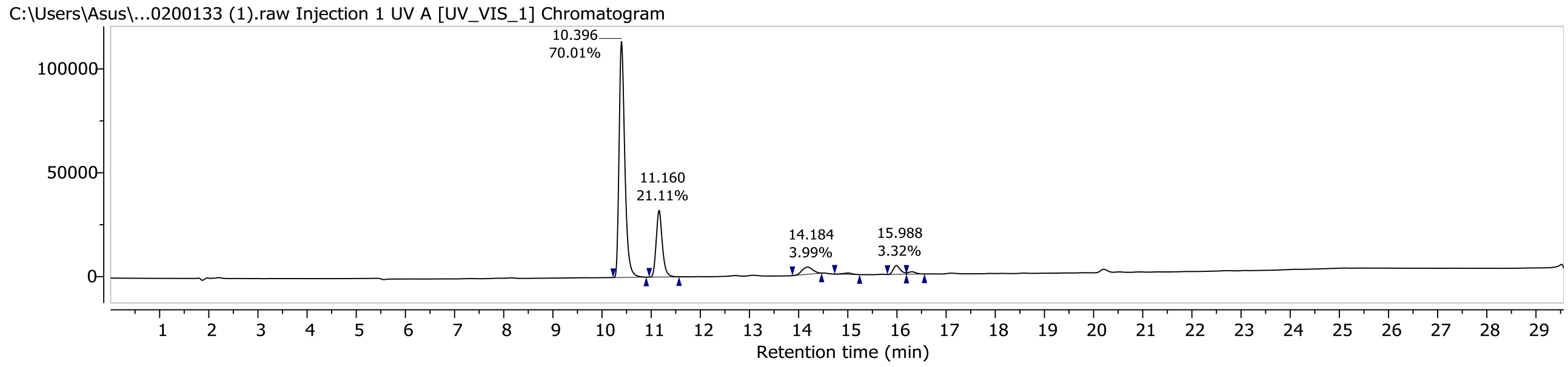

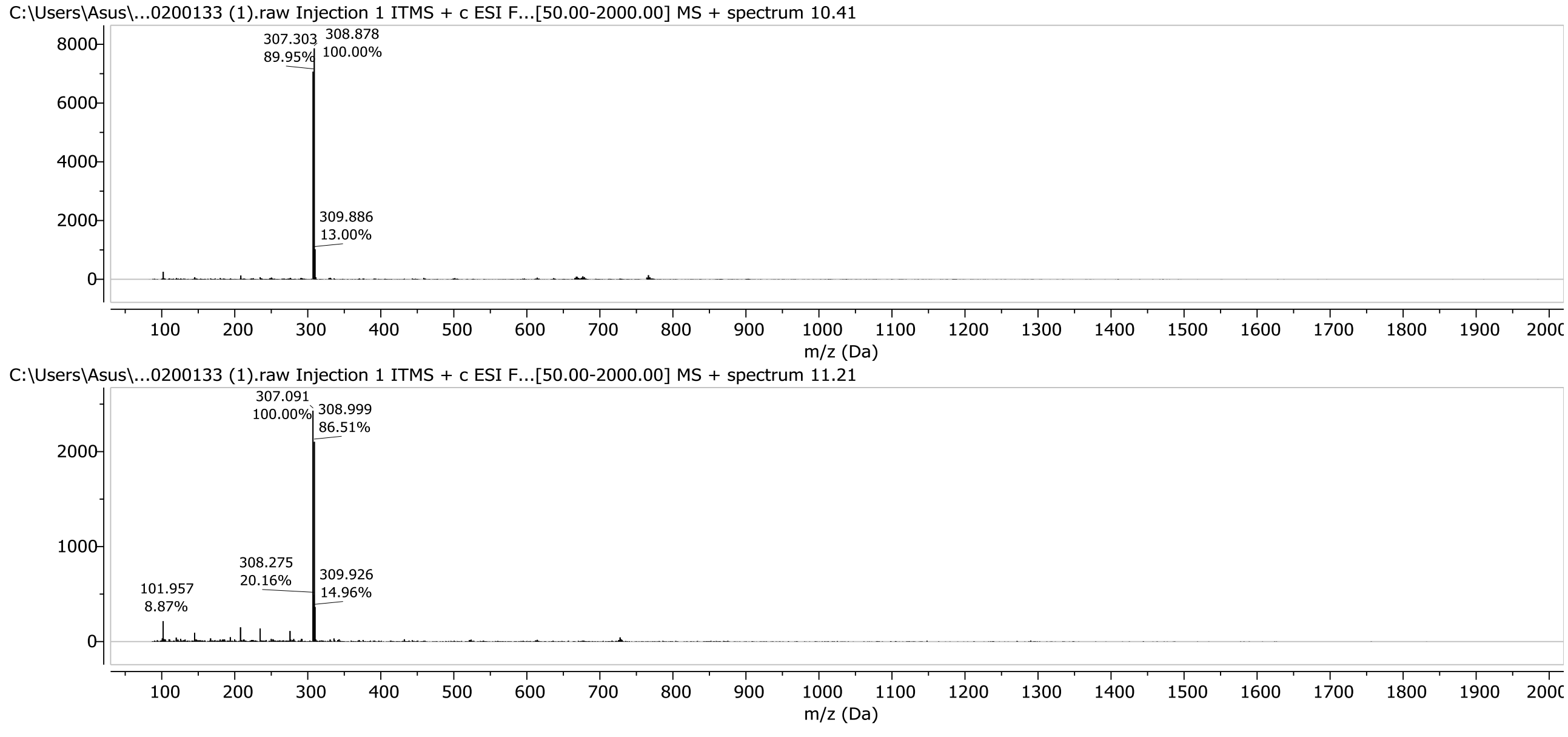

Synthesis of 2-((6-chloro-4-methylquinazolin-2-yl)methylene)hydrazine-1-carboximidamide hydrochloride 1c

The synthesis of 2-((6-chloro-4-methylquinazolin-2-yl)methylene)hydrazine-1-carboximidamide hydrochloride **1c** is described in the main text. The analytical characterization of intermediate (6-chloro-4-methylquinazolin-2-yl)methanol **6c**, 6-chloro-4-methylquinazoline-2-carbaldehyde **7c** and 2-((6-chloro-4-methylquinazolin-2-yl)methylene)hydrazine-1-carboximidamide hydrochloride **1c** is reported below.

^{1}H NMR (400 MHz, $CDCl_3$) δ 8.08 (d, *J* = 2.3 Hz, 1H, H5), 7.96 (d, *J* = 8.9 Hz, 1H, H8), 7.83 (dd, *J*

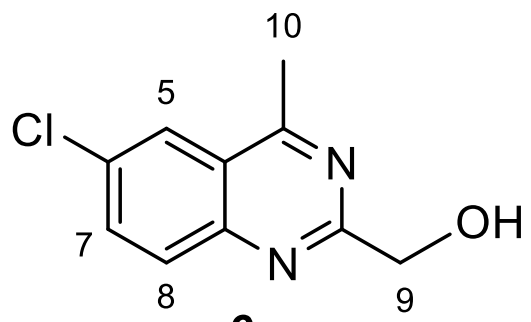

6c

= 8.9, 2.3 Hz, 1H, H7), 4.93 (s, 2H, H9), 3.91 (s, 1H, -OH), 2.94 (s, 3H, H10).

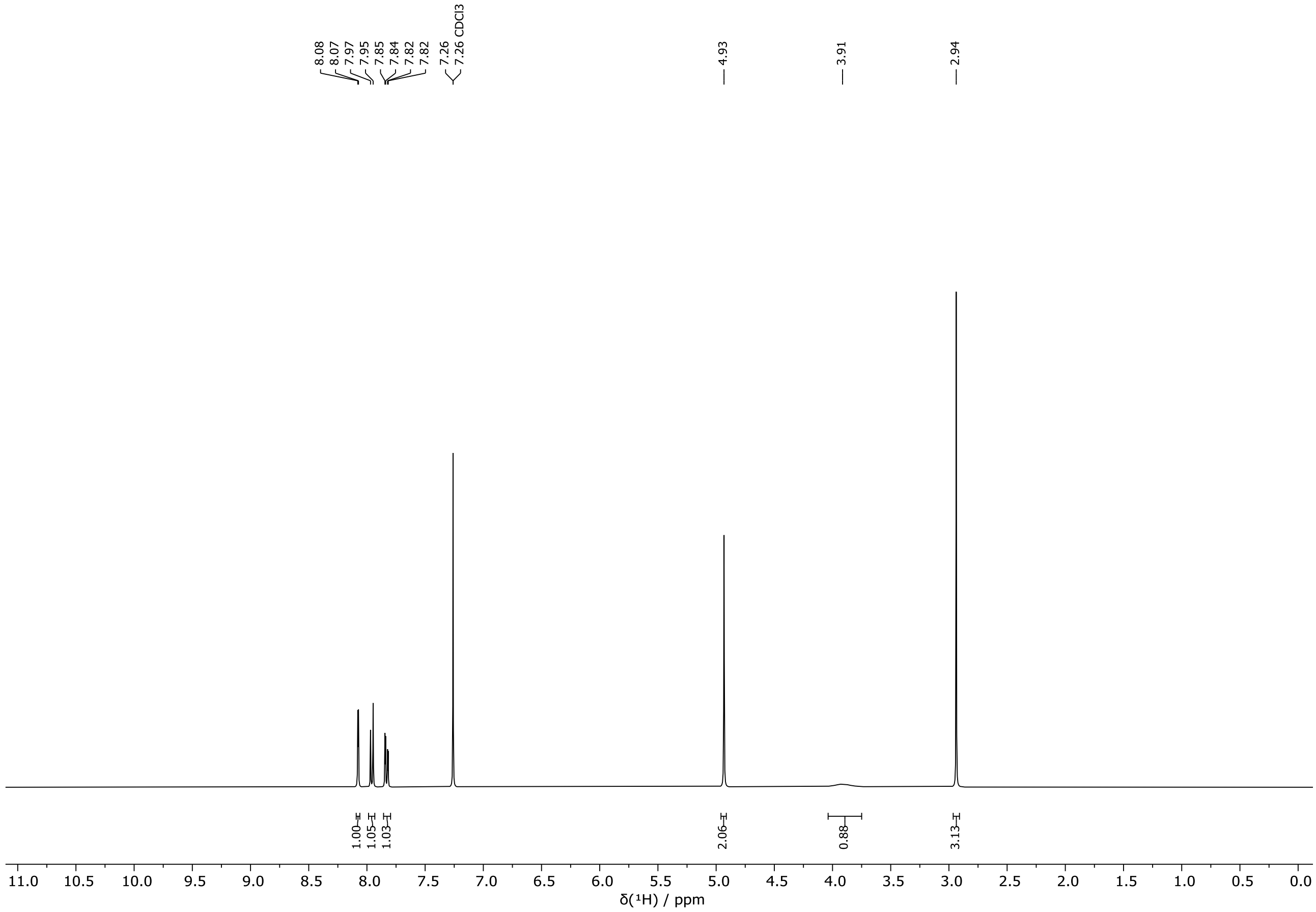

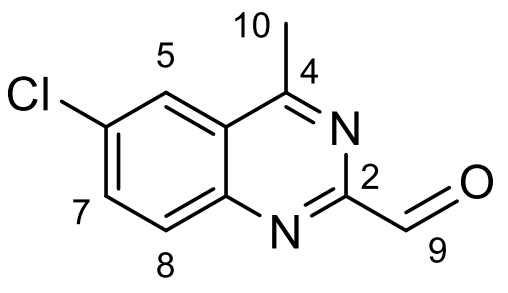

7c

^{1}H NMR (400 MHz, $CDCl_3$) δ 10.16 (s, 1H, H9), 8.17 – 8.10 (m, 2H, H5, H8), 7.90 (dd, J = 9.0, 2.4 Hz, 1H, H7), 3.01 (s, 3H, H10).

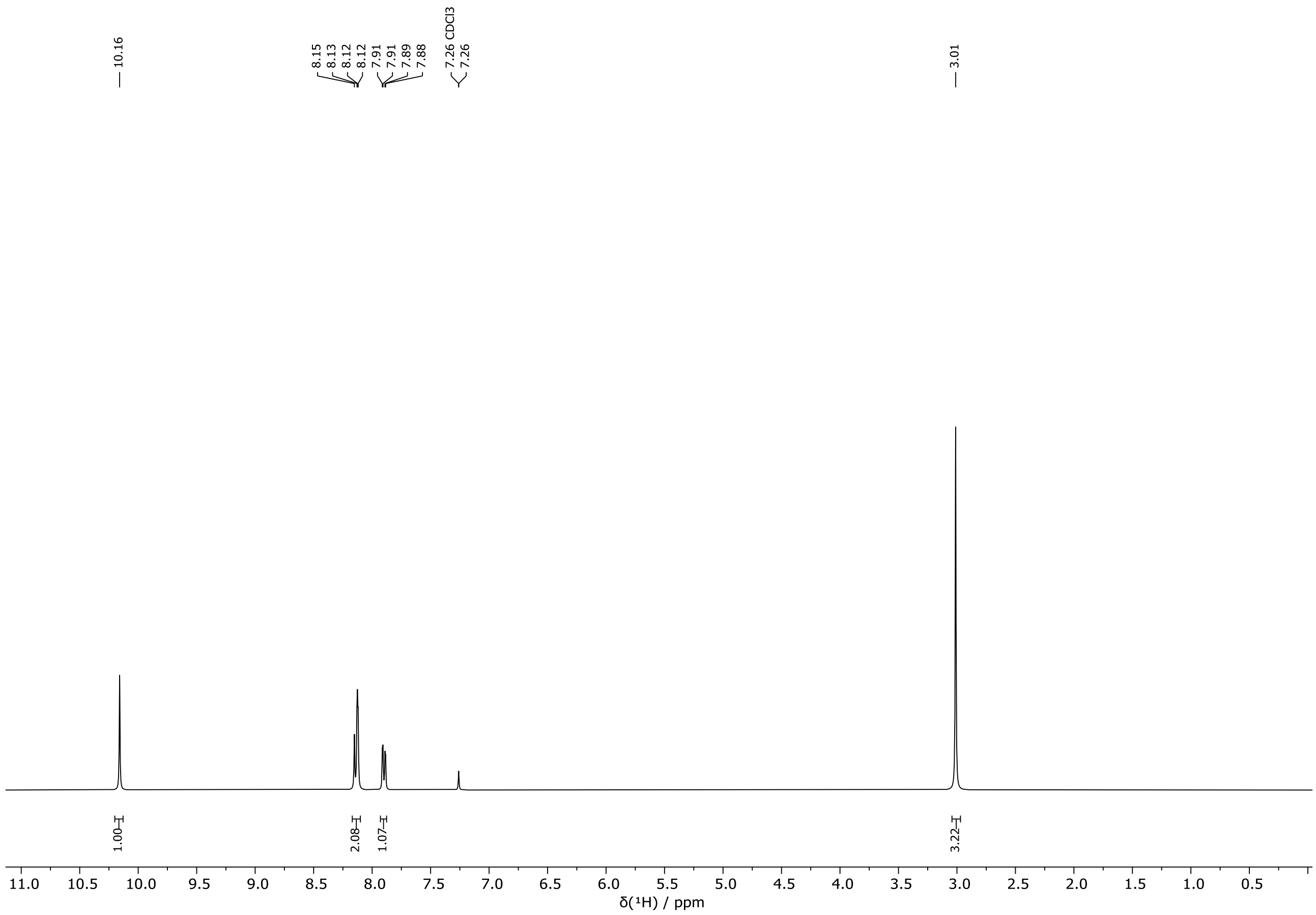

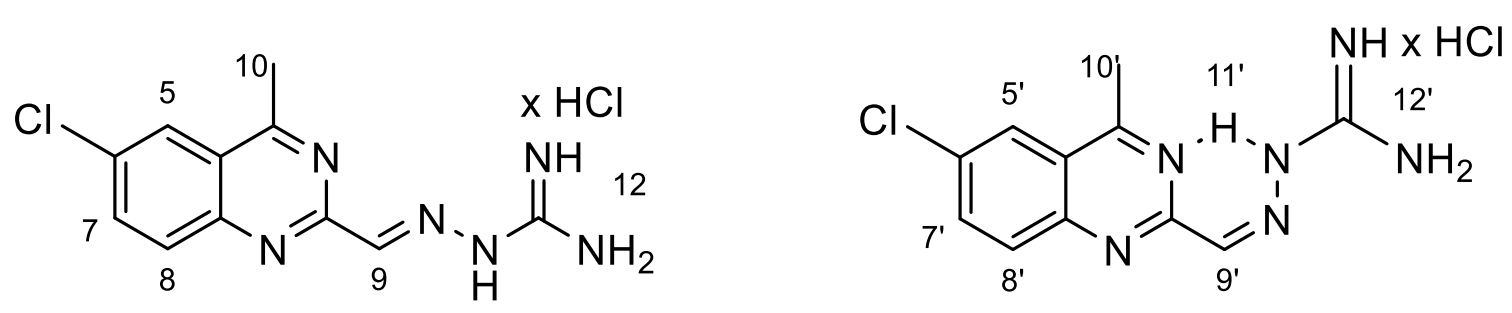

1cII **1cI**

^{1}H NMR (400 MHz, DMSO-d_6) δ 13.90 (s, 1H, H11'), 12.61 (s, 1H, H11), 8.73 – 8.36 (brs, 4H, H12'), 8.57 (d, J = 9.0 Hz, H8'), 8.44 (d, J = 2.3 Hz, 1H, H5'), 8.40 (s, 1H, H5), 8.34 (s, 1H, H9), 8.15 (dd, J = 9.0, 2.3 Hz, 1H, H7'), 8.04 (m, 2H, H7, H8), 8.01 (brs, 4H, H13), 7.73 (s, 1H, H9'), 3.02 (s, 3H, H10'), 2.96 (s, 3H, H10).

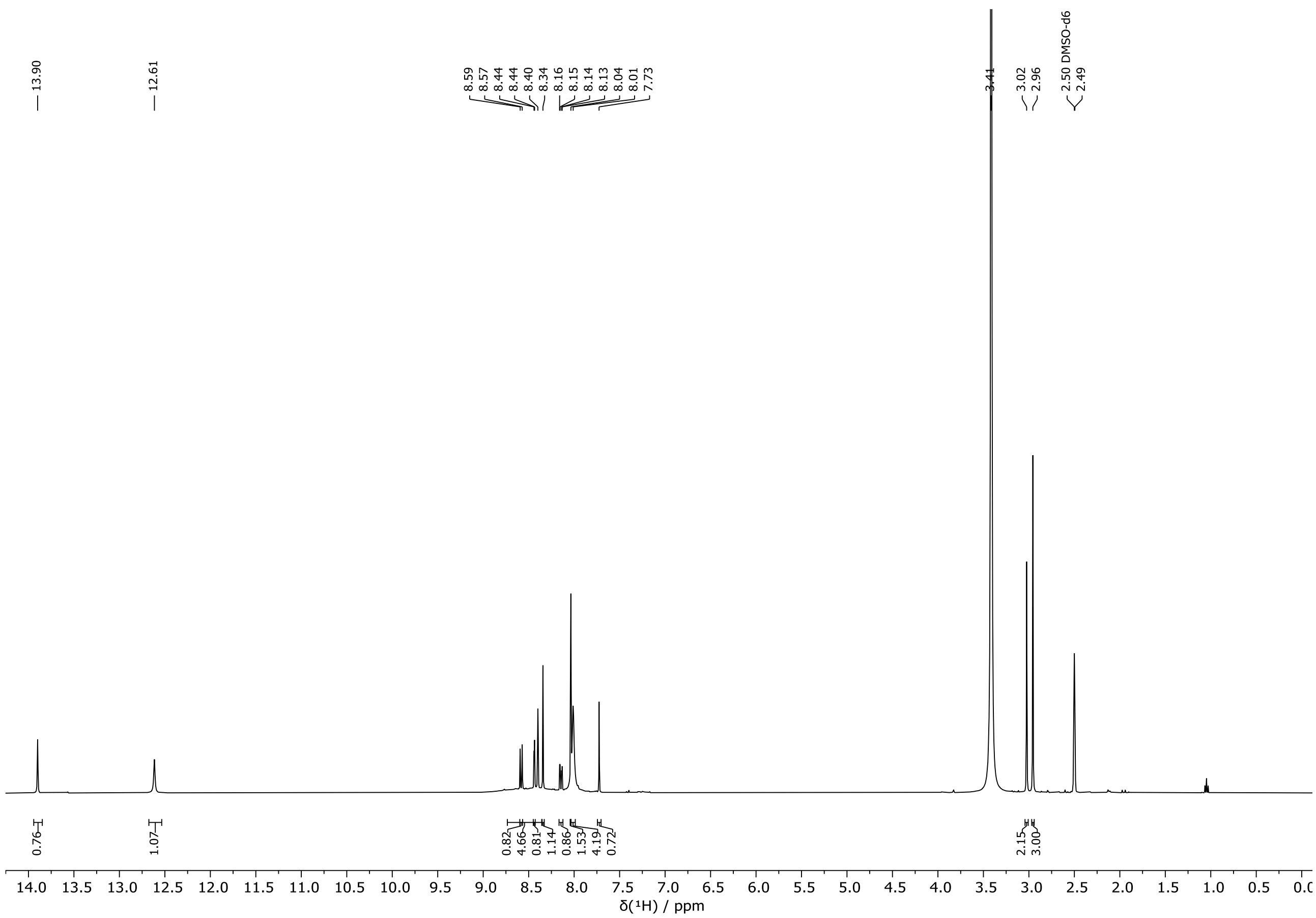

^{13}C NMR (101 MHz, DMSO-d_6) δ 170.14, 169.22, 156.11, 155.79, 155.60, 154.69, 147.66, 146.34, 145.83, 138.28, 135.61, 135.28, 133.92, 132.89, 131.04, 130.59, 125.19, 125.07, 123.78, 123.29, 21.80, 21.64.

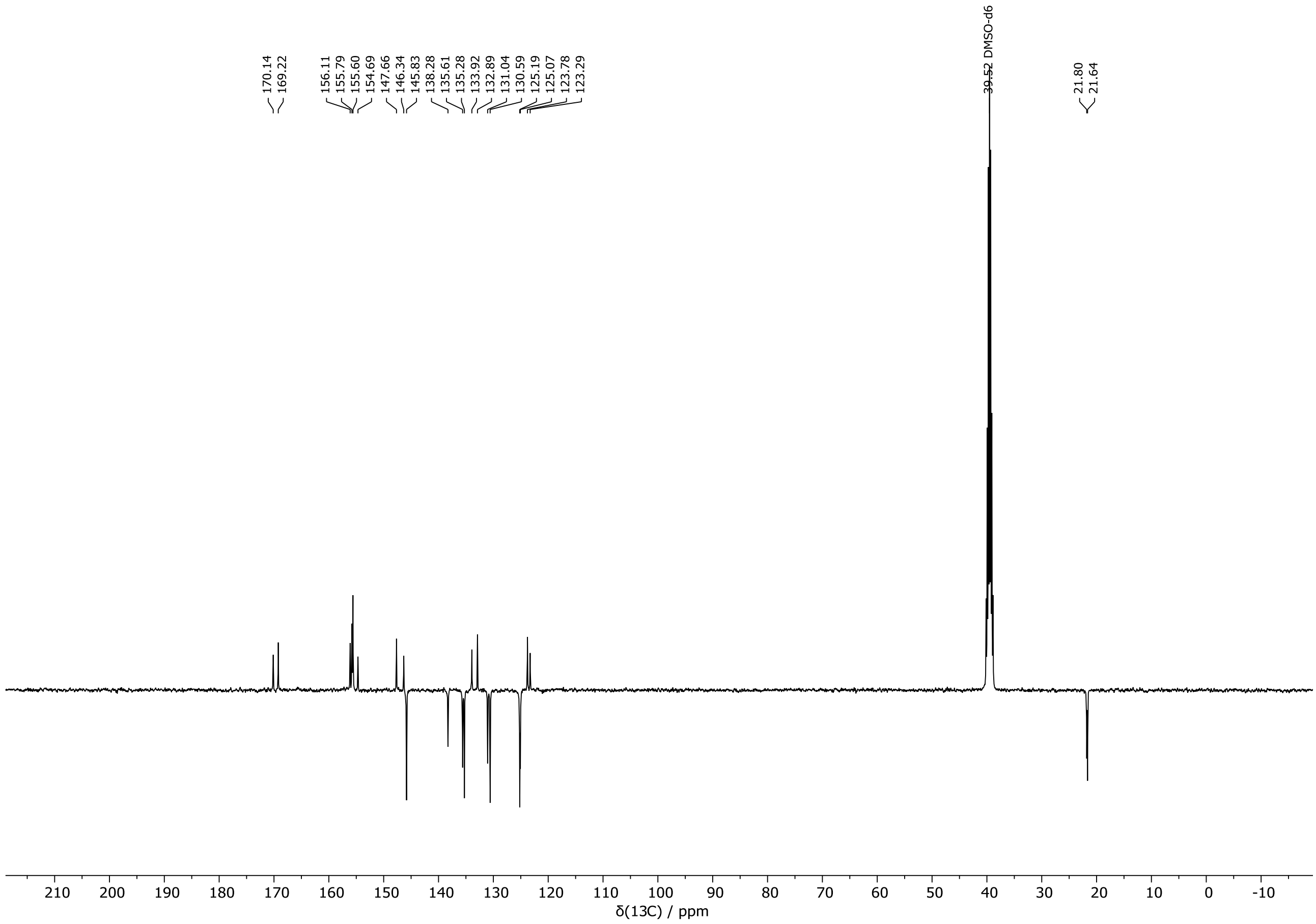

MS (ESI^+): m/z [$M + H^+$] 263.13. Calculated MS for $C_{11}H_{12}ClN_6$: 263.08.

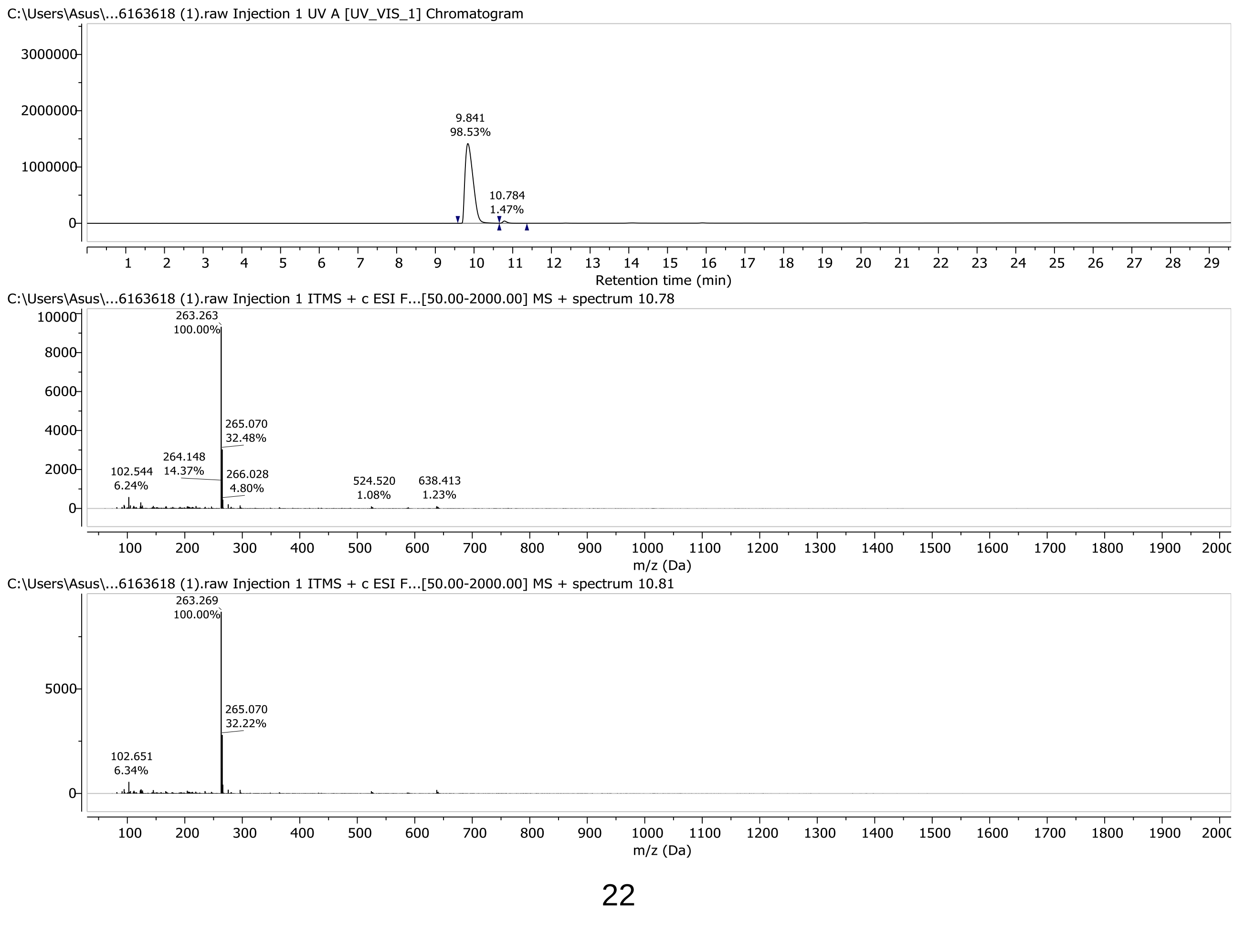

Synthesis of 2-((methyl-6-phenylquinazolinyl)methylene)hydrazine-1-carboxIMIDamide hydrochloride 1d

Synthesis of (4-methyl-6-phenylquinazolin-2-yl)methanol 6d

The reaction was performed according to **general procedure A**, using 1-(4-amino-[1,1'-biphenyl]-3-yl)ethan-1-one **8d** (136 mg, 0.62 mmol, 1 eq.), triethanolamine (459 mg, 3.0 mmol, 5 eq.), eosin Y (40 mg, 0.062 mmol, 0.1 eq.), NH_4Cl (99 mg, 1.85 mmol, 3 eq.) and 1:1 CH_3CN/H_2O (4.0 mL). Chromatographic purifications did not allow the separation of the 2-unsubstituted byproduct and the isolation of pure intermediate (4-methyl-6-phenylquinazolin-2-yl)methanol **6d**. The mixture (1:0.2 product:byproduct ratio), used as such in the next reaction step, appeared as a pale yellow solid (100 mg).

Reaction time: 24 hours.

TLC monitoring: eluent mixture 7:3 *n*-Hex/EtOAc.

BIOTAGE® Direct phase flash column chromatography: eluent mixture from 11% to 90% EtOAc in *n*-Hex.

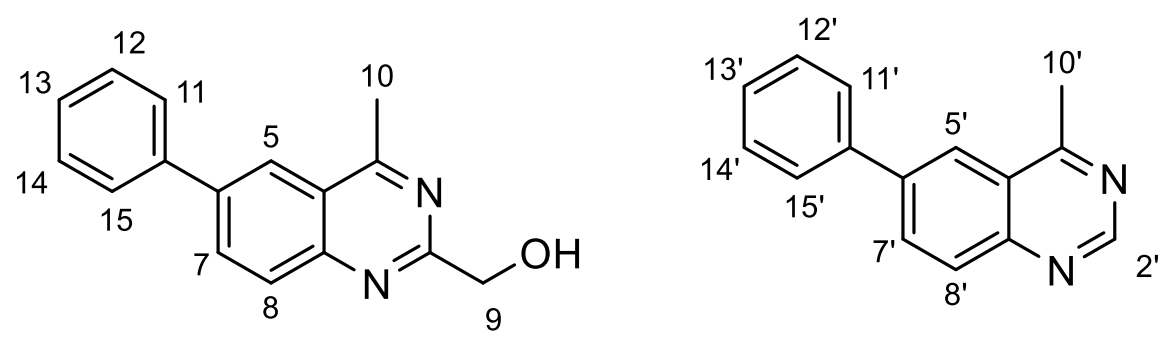

^{1}H NMR (400 MHz, $CDCl_3$) δ 9.18 (s, 1H, H2’), 8.24 (d, *J* = 2.2 Hz, 2H, H5, H5’), 8.15 (dd, *J* = 8.7, 2.2 Hz, 2H, H7, H7’), 8.07 (d, *J* = 8.7 Hz, 2H, H8, H8’), 7.70 (d, *J* = 7.1 Hz, 4H, H11, H11’, H15, H15’), 7.53 (t, *J* = 7.5 Hz, 4H, H12, H12’, H14, H14’), 7.45 (d, *J* = 7.1 Hz, 2H, H13, H13’), 4.96 (s, 2H, H9), 3.01 (s, 6H, H10, H10’).

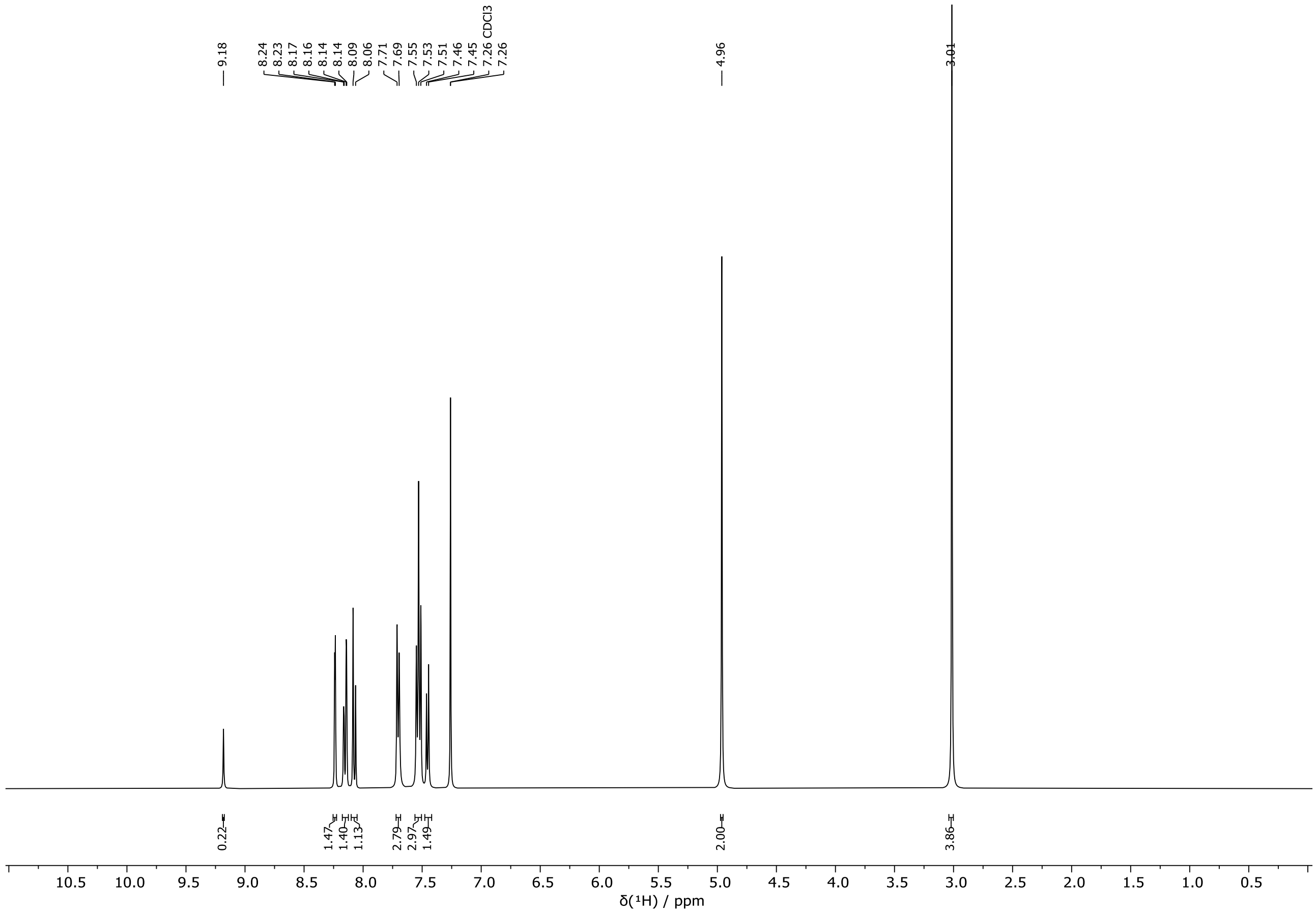

Synthesis of methyl-6-phenylquinazoline 2-carbaldehyde 7d

The reaction was performed according to **general procedure B**, using the impure (4-methyl-6-phenylquinazolin-2-yl)methanol **6d** (100 mg, 0.40 mmol, 1 eq.), DMP (220 mg, 0.52 mmol, 1.3 eq), and dry DCM (4.0 mL). The chromatographic purification did not allow the separation of the 2-unsubstituted byproduct and the isolation of the pure intermediate methyl-6-phenylquinazoline 2-carbaldehyde **7d**. The mixture (1:1 product:byproduct ratio), used as such in the next reaction step, appeared as a pale yellow, oily, solid (50 mg).

Reaction time: 1 hour.

TLC monitoring: eluent mixture 55:45 *n*-Hex/EtOAc.

BIOTAGE® Direct phase flash column chromatography: eluent mixture from 11% to 90% EtOAc in *n*-Hex.

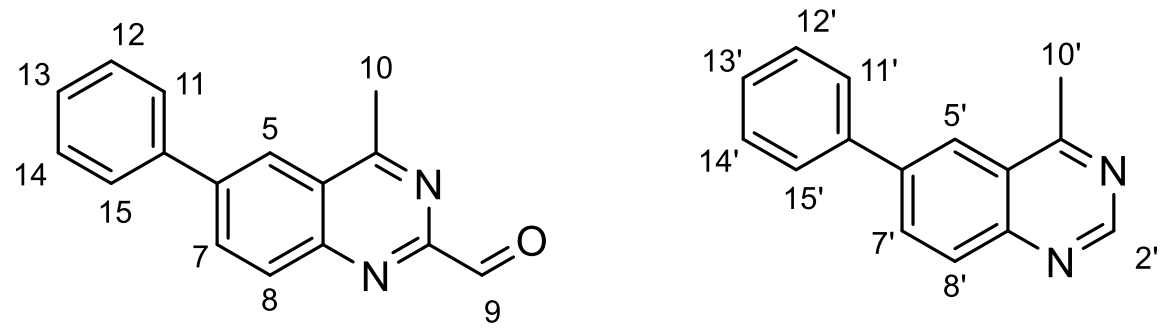

^{1}H NMR (400 MHz, $CDCl_3$) δ 10.20 (s, 1H, H9), 9.15 (s, 1H, H2'), 8.30 – 8.18 (m, 2H, H5, H5', H7, H7'), 8.16 – 8.04 (m, 2H, H8, H8'), 7.76 – 7.63 (m, 2H, H11, H11', H15, H15'), 7.57 – 7.43 (m, H12, H12', H14, H14'), 3.09 (s, 1H, H10), 2.99 (s, 1H, H10').

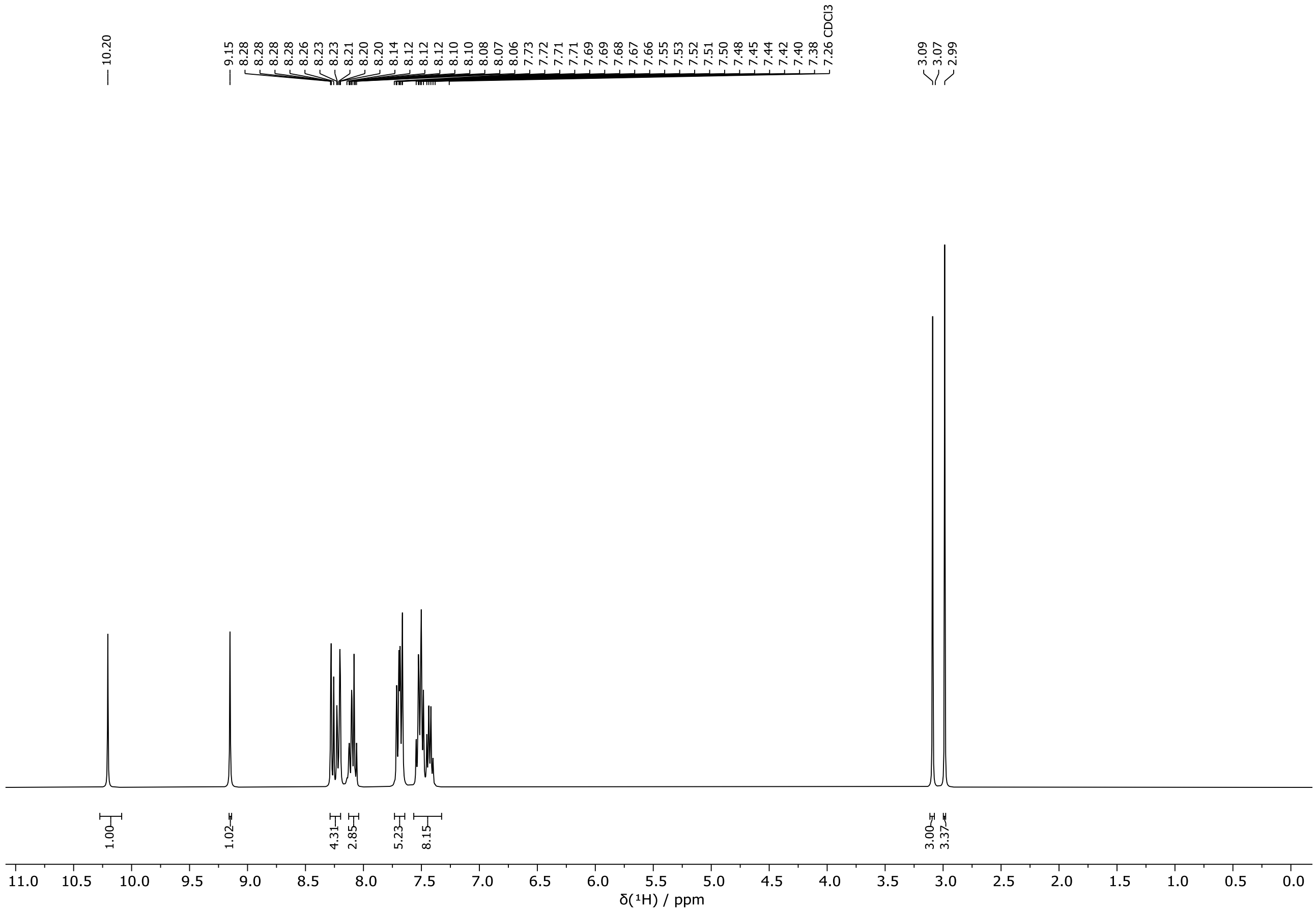

Synthesis of 2-((methyl-6-phenylquinazolinyl)methylene)hydrazine-1-carboximidamide hydrochloride 1d

The reaction was performed according to **general procedure C**, using the impure 6-phenyl-methylquinazoline carbaldehyde **7d** (50 mg, 0.20 mmol, 1 eq.), AG·HCl (8.4 mg, 0.076 mmol, 0.38 eq.) and EtOH (2.0 mL). Trituration was done in (*i*-Pr)$_2$O (2 x 2.5 mL). After purification, pure target 2-((methyl-6-phenylquinazolinyl)methylene)hydrazine-1-carboximidamide hydrochloride **1d** was obtained as a yellow solid (12 mg, 0.035 mmol, 6% yield over three steps).

1:0.16 E/Z ratio (86%/14%).

Reaction time: 7 hours, then rt overnight.

TLC monitoring: eluent mixture 1:1 DCM/EtOAc.

BIOTAGE® Reverse phase flash column chromatography: eluent mixture from 0% to 100% CH_3CN in H_2O.

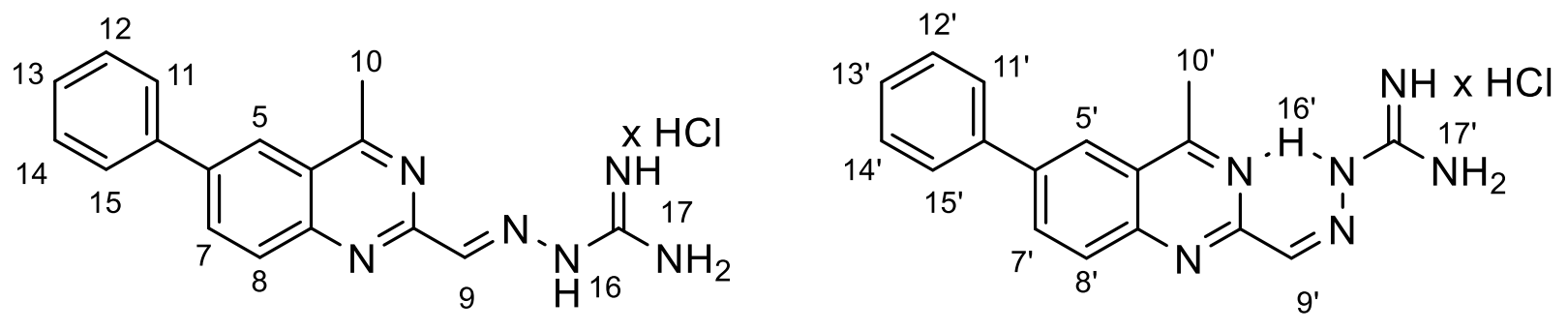

^{1}H NMR (400 MHz, DMSO-d_6) δ 14.10 (s, 1H, H16'), 12.57 (s, 1H, H16), 8.59 (d, *J* = 9.0 Hz, 1H, H8'), 8.50 (d, *J* = 2.1 Hz, 1H, H5), 8.46 (d, *J* = 2.2 Hz, 1H, H5'), 8.45-8.35 (brs, 4H, H17'), 8.38 (s, 1H, H9), 8.36 (dd, *J* = 8.8, 2.1 Hz, 1H, H7), 8.33 (d, *J* = 9.5 Hz, 2H, H11', H15'), 8.17 (dd, *J* = 9.1, 2.4 Hz, 1H, H7'), 8.11 (d, *J* = 8.8 Hz, 1H, H8), 7.95 (brs, 4H, H17), 7.92 (d, *J* = 7.3 Hz, 2H, H11, H15), 7.66 (s, 1H, H9'), 7.56 (t, *J* = 7.5 Hz, 4H, H12, H12', H14, H14'), 7.47 (t, *J* = 7.3 Hz, 2H, H13, H13'), 3.06 (s, 3H, H10), 2.95 (s, 3H, H10').

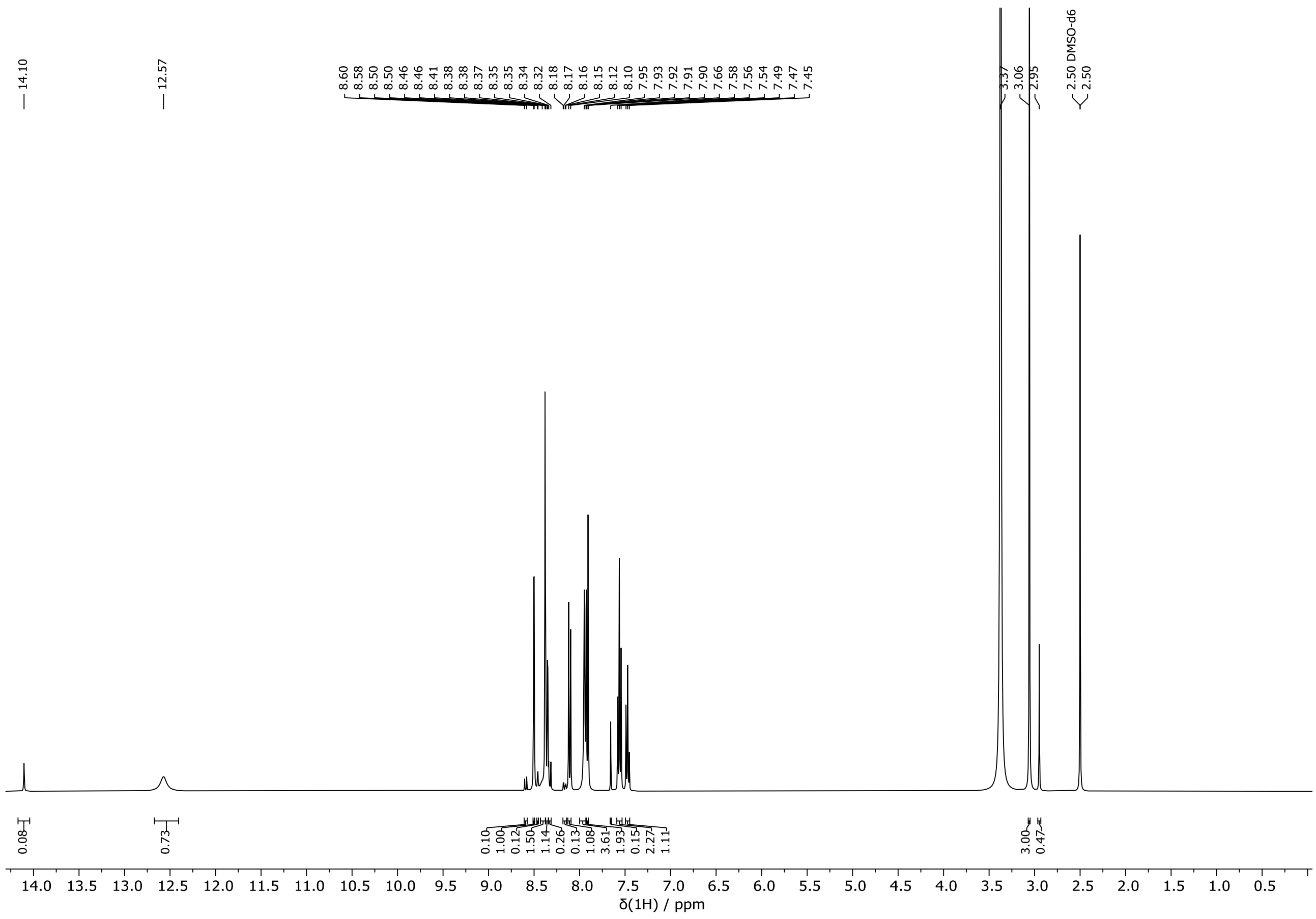

^{13}C NMR (101 MHz, DMSO-d_6) δ 169.52, 166.95, 155.53, 155.45, 155.34, 150.73, 148.20, 146.24, 141.80, 139.89, 138.51, 133.51, 129.42, 128.95, 128.90, 128.86, 127.78, 127.21, 125.37, 123.73, 123.14, 123.00, 65.67, 47.25, 39.93, 39.73, 39.52, 39.31, 39.10, 38.89, 38.68, 30.46, 21.52.

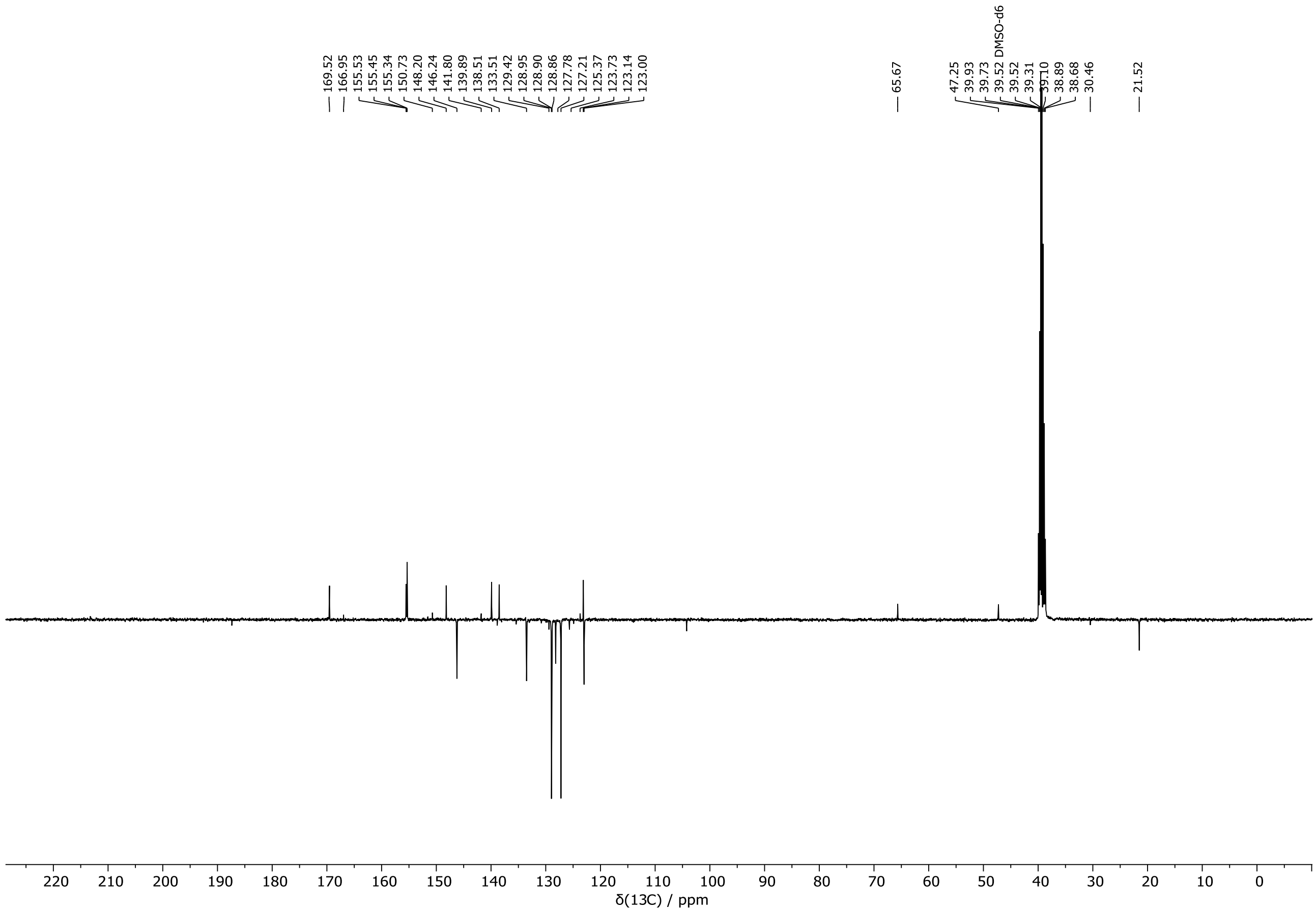

MS (ESI$^+$): m/z [M + H$^+$] 305.19. Calculated MS for $C_{17}H_{17}N_6$: 305.15.

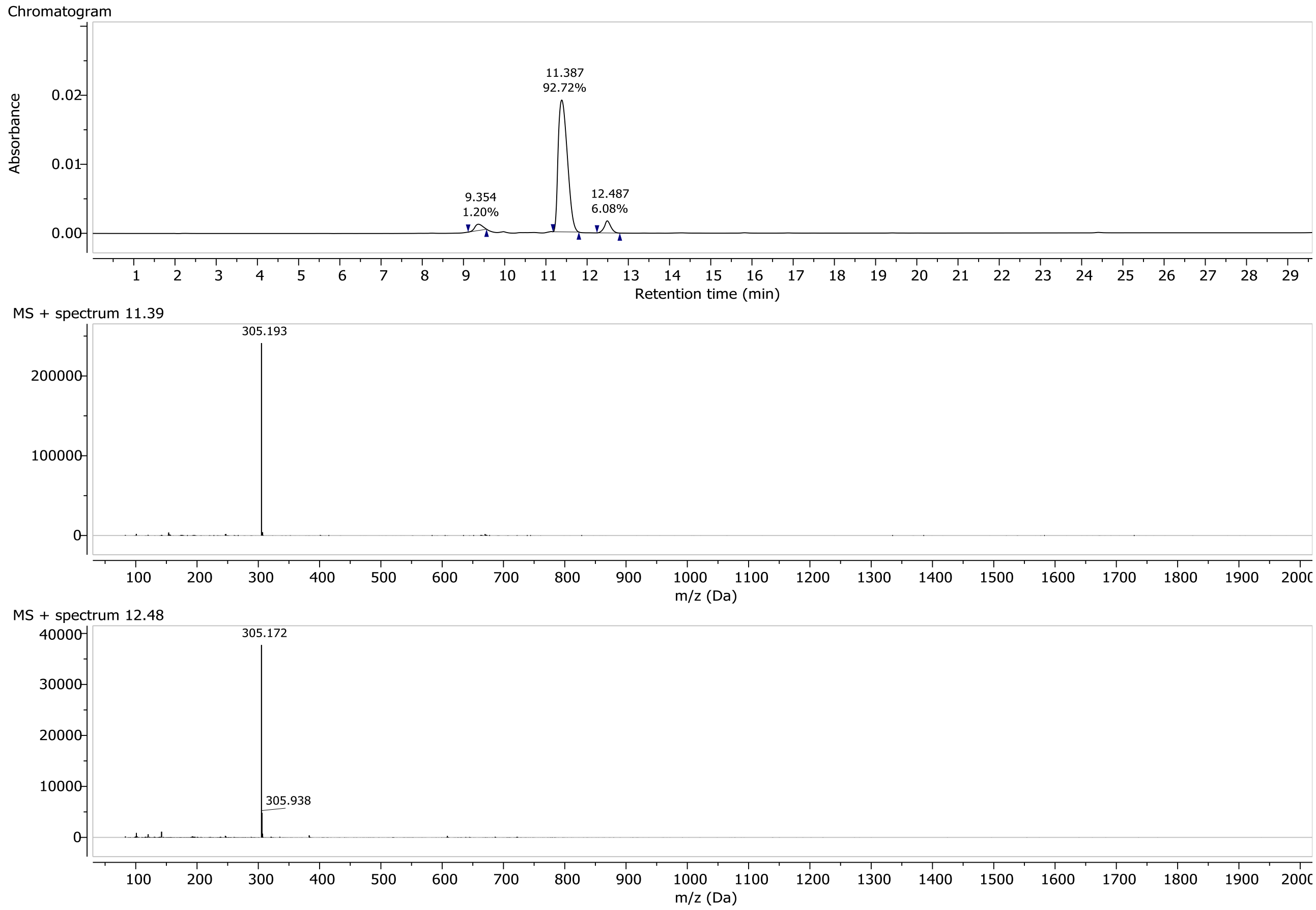

Synthesis of 2-((methyl-6-morpholinoquinazolinyl)methylene)hydrazine-1-carboximidamide hydrochloride 1e

Synthesis of (4-methyl-6-morpholinoquinazolin-2-yl)methanol 6e

The reaction was performed according to **general procedure A**, using 1-(2-amino-5-morpholinophenyl)-ethan-1-one **8e** (189 mg, 0.9 mmol, 1 eq.), triethanolamine (640 mg, 4.3 mmol, 5 eq), eosin Y (56 mg, 0.09 mmol, 0.1 eq.), NH_4Cl (138 mg, 2.6 mmol, 3 eq.) and 1:1 CH_3CN/H_2O (5.8 mL). Chromatographic purifications did not allow the separation of the 2-unsubstituted byproduct, and the isolation of the pure desired intermediate (4-methyl-6-morpholinoquinazolin-2-yl)methanol **6e**. The mixture (1:1.2 product:byproduct ratio), used as such in the next reaction step, appeared as a dark amber, oily solid (96 mg).

Reaction time: 72 hours.

TLC monitoring: eluent mixture 3:7 DCM/EtOAc.

BIOTAGE® Direct phase flash column chromatography: eluent mixture from 17% to 100% EtOAc in DCM.

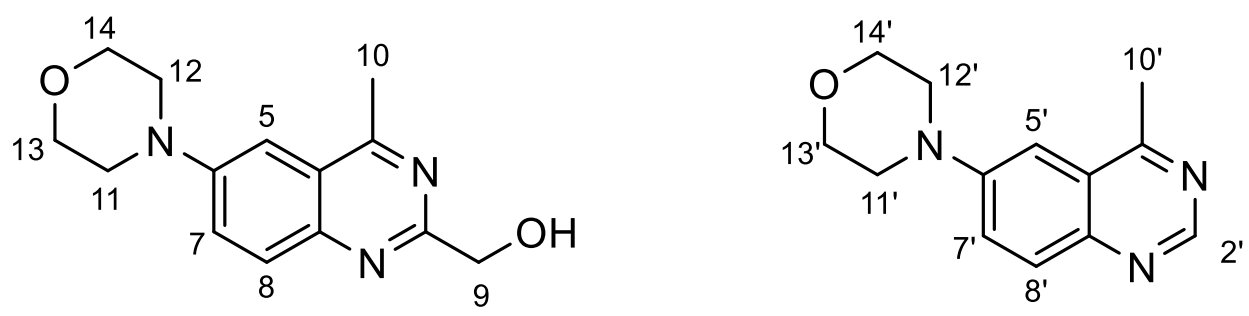

^{1}H NMR (400 MHz, $CDCl_3$) δ 8.98 (s, 1H, H2’), 7.88 (d, *J* = 9.2 Hz, 1H, H8’), 7.84 (d, *J* = 9.2 Hz, 1H, H8), 7.59 (dd, *J* = 9.3, 2.7 Hz, 2H, H7, H7’), 7.12 (dd, *J* = 6.2, 2.7 Hz, 2H, H5, H5’), 4.86 (s, 2H, H9), 3.93 – 3.86 (m, 8H, H11, H12, H11’, H12’), 3.32 – 3.22 (m, 8H, H13, H14, H13’, H14’), 2.84 (s, 6H, H10, H10’).

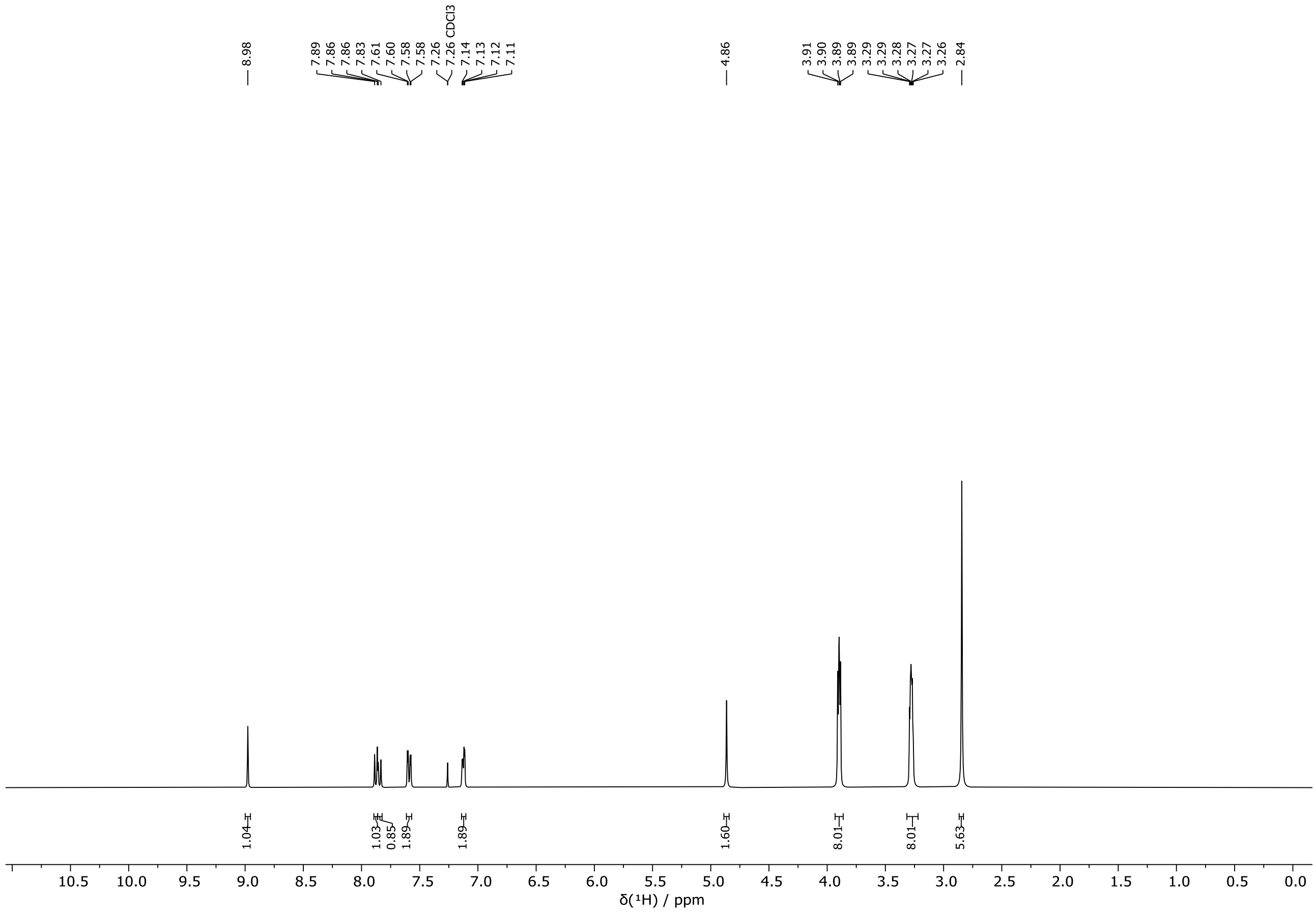

Synthesis of methyl-6-morpholinoquinazoline 2-carbaldehyde 7e

The reaction was performed according to **general procedure B**, using the impure (4-methyl-6-morpholinoquinazolin-2-yl)methanol **6e** (96 mg, 0.37 mmol, 1 eq.), DMP (203 mg, 0.48 mmol, 1.3 eq.) and dry DCM (3.7 mL). After purification, pure methyl-6-morpholinoquinazoline 2-carbaldehyde **7e** was obtained as an orange solid (37 mg, 0.143 mmol, 17% yield over two steps).

Reaction time: 2 hours.

TLC monitoring: eluent mixture 3:7 DCM/EtOAc.

BIOTAGE® Direct phase flash column chromatography: eluent mixture from 17% to 100% EtOAc in DCM.

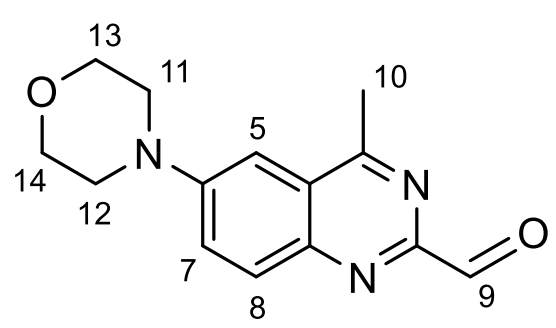

^{1}H NMR (400 MHz, $CDCl_3$) δ 10.14 (s, 1H, H9), 8.08 (d, *J* = 9.3 Hz, 1H, H8), 7.69 (dd, *J* = 9.7, 2.7 Hz, 1H, H7), 7.17 (d, *J* = 2.7 Hz, 1H, H5), 3.95 – 3.92 (m, 4H, H11, H12), 3.42 – 3.39 (m, 4H, H13, H14), 2.96 (s, 3H, H10).

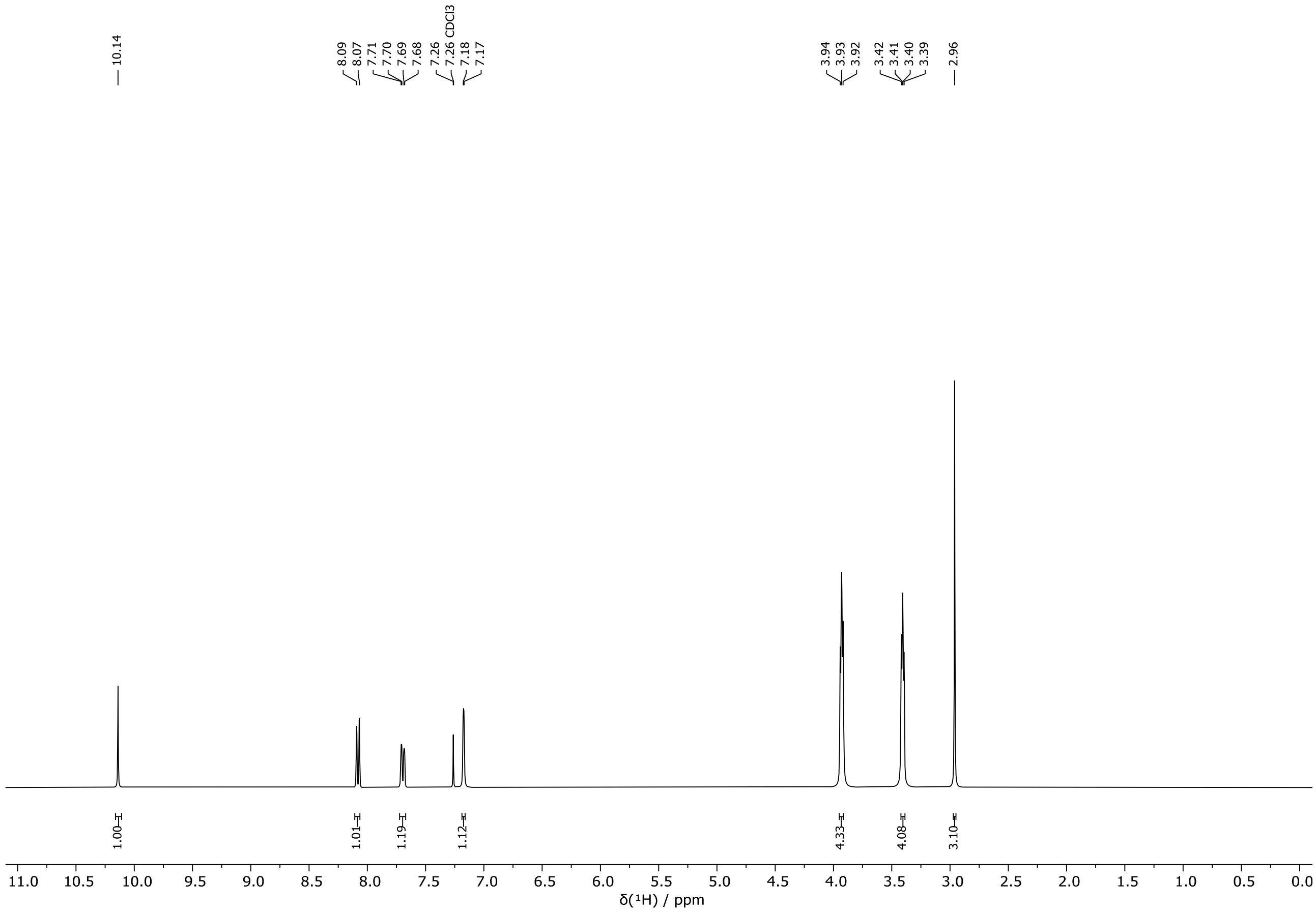

Synthesis of 2-((6-morpholino-4-methyl-quinazolinyl)methylene)hydrazine-1-carboximidamide hydrochloride 1e

The reaction was performed according to **general procedure C**, using 6-morpholino-methylquinazoline 2-carbaldehyde **7e** (18 mg, 0.068 mmol, 1 eq.), AG·HCl (6.8 mg, 0.061 mmol, 0.90 eq.) and EtOH (0.7 mL). Trituration was done in Et_2O (3 x 3 mL). After purification, pure target 2-((methyl-6-morpholinoquinazolinyl)methylene)hydrazine-1-carboximidamide hydrochloride **1e** was obtained as a red solid (18 mg, 0.051 mmol, 84% yield).

1:0.73 E/Z ratio (58%/42%).

Reaction time: 7 hours, then rt overnight.

TLC monitoring: eluent mixture 1:1 DCM/EtOAc.

BIOTAGE® Reverse phase flash column chromatography: eluent mixture from 0% to 100% CH_3CN in H_2O.

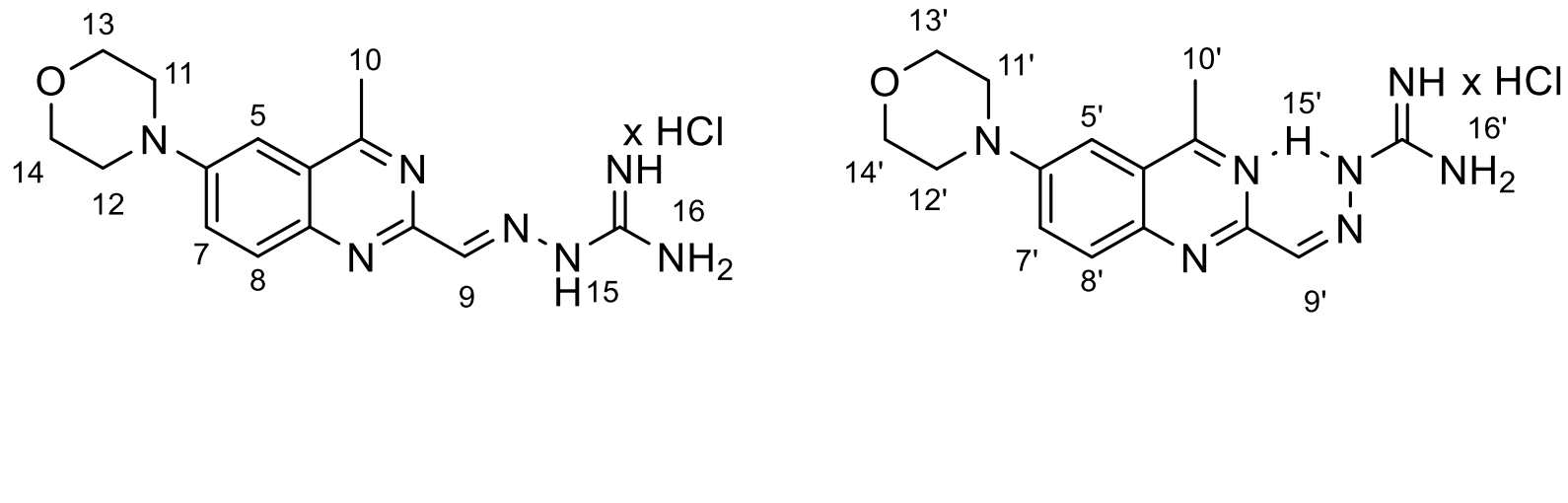

^{1}H NMR (400 MHz, DMSO-d_6) δ 14.10 (s, 1H, H15'), 12.55 (s, 1H, H15), 8.50 (brs, 4H, H16'), 8.30 (d, J = 9.4 Hz, 1H, H8'), 8.27 (s, 1H, H9), 8.02 (d, J = 9.4 Hz, 1H, H7'), 7.98 (m, 5H, H8, H16), 7.89 (m, 2H, H7, H7'), 7.65 (s, 1H, H9'), 7.31 (m, 2H, H5, H5'), 3.80 (m, 8H, H13, H14, H13', H14'), 3.44 – 3.36 (m, 8H, H11, H12, H11', H12'), 2.94 (s, 2H, H10), 2.90 (s, 3H, H10').

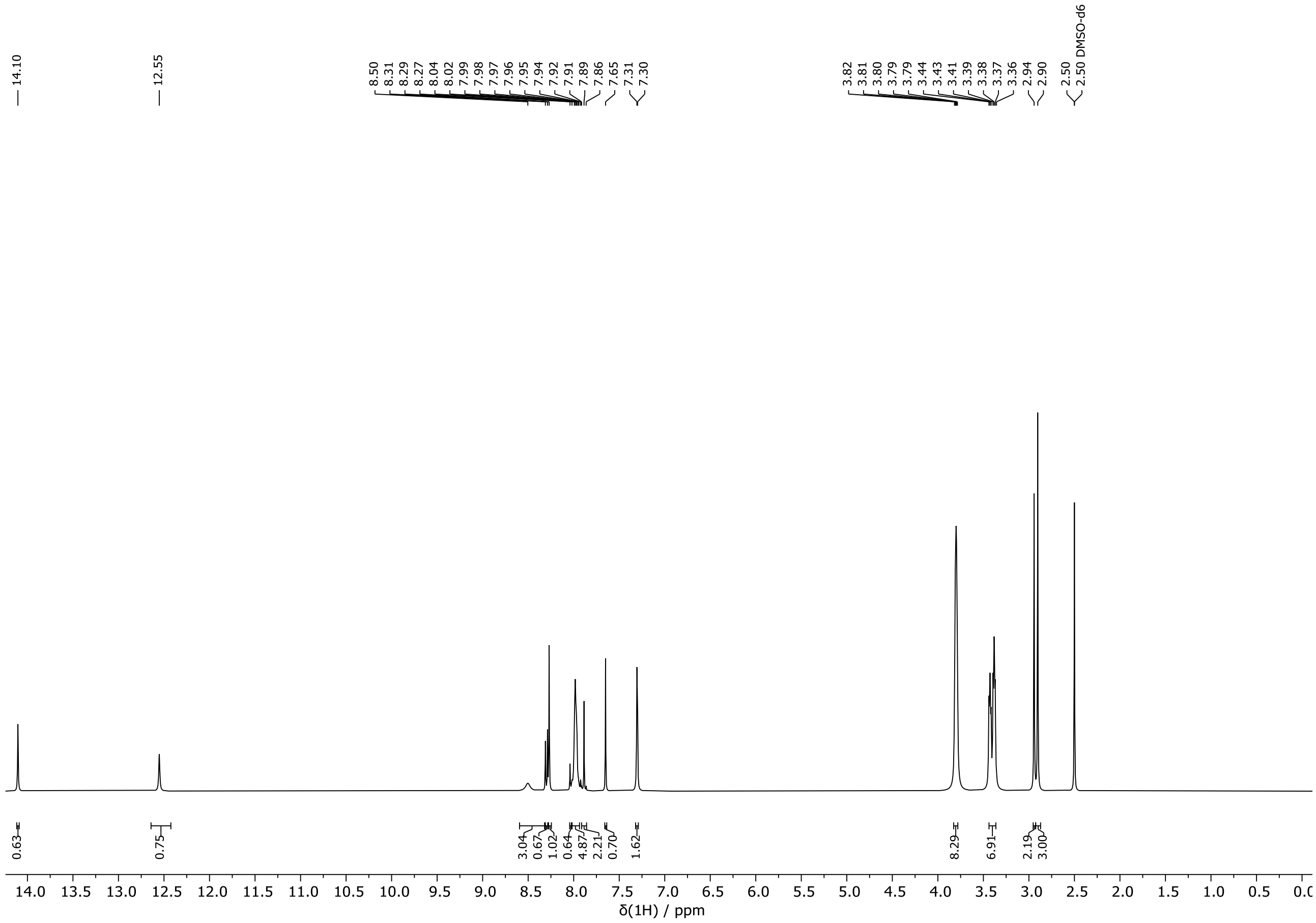

^{13}C NMR (101 MHz, D_2O) δ 167.62, 167.24, 156.14, 156.01, 152.94, 152.23, 151.40, 150.85, 146.77, 143.94, 142.46, 139.19, 124.71, 124.40, 105.51, 104.91, 66.38, 66.35, 48.14, 47.93, 45.91, 40.61, 40.40, 40.20, 39.99, 39.77, 39.57, 39.36, 22.15, 21.91.

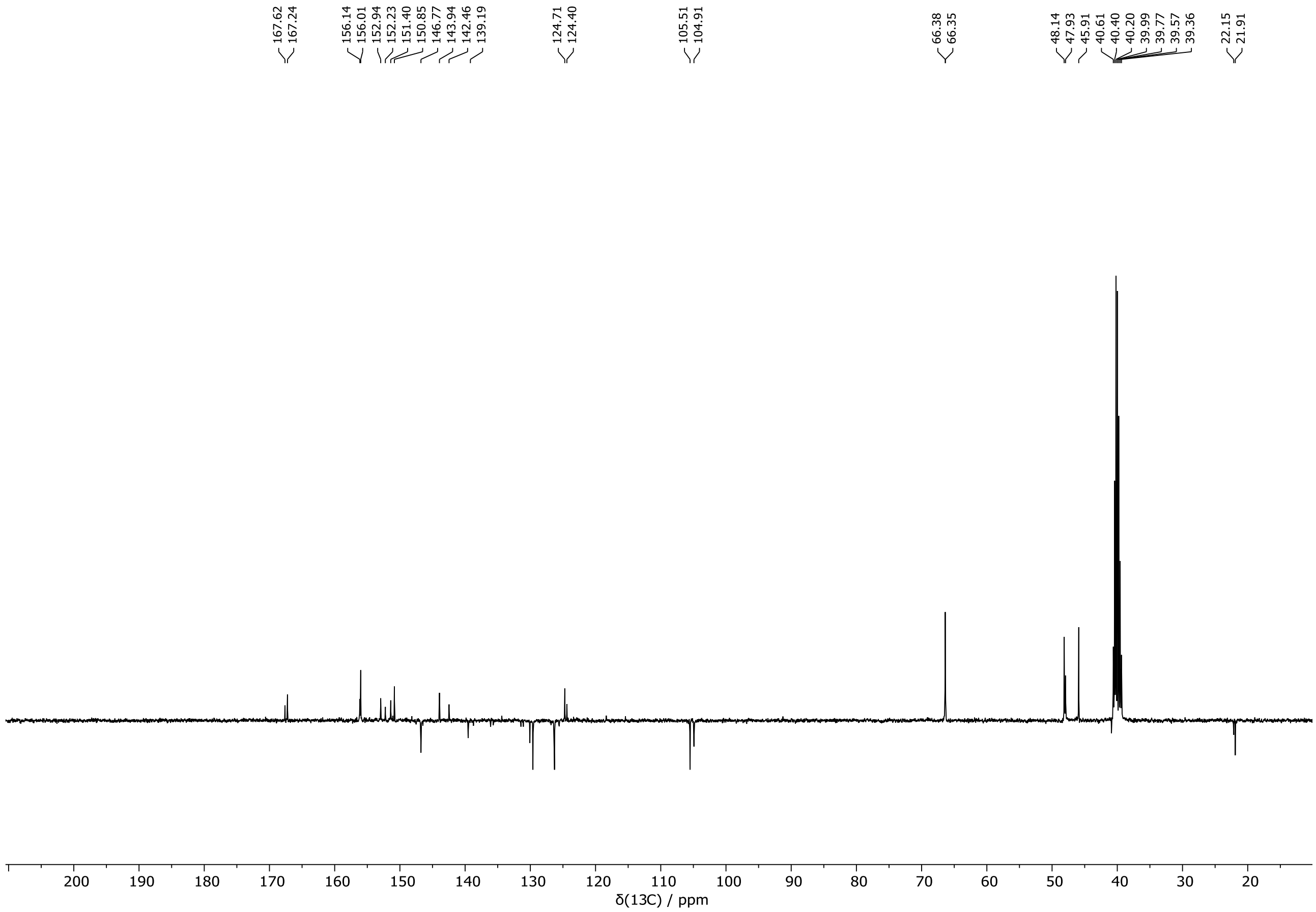

MS (ESI$^+$): m/z [M + H$^+$] 314.12. Calculated MS for $C_{15}H_{20}N_7O$: 314.17.

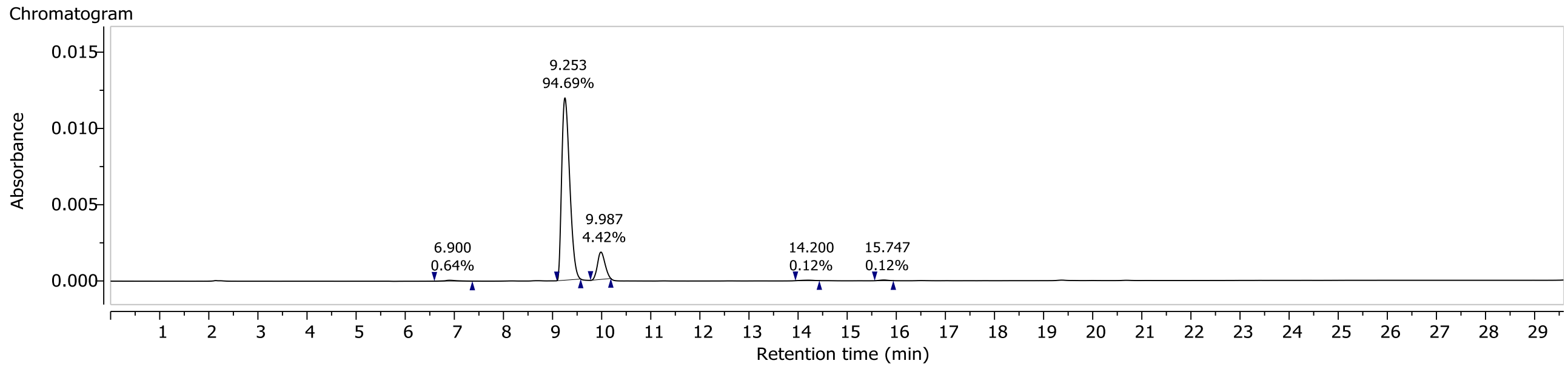

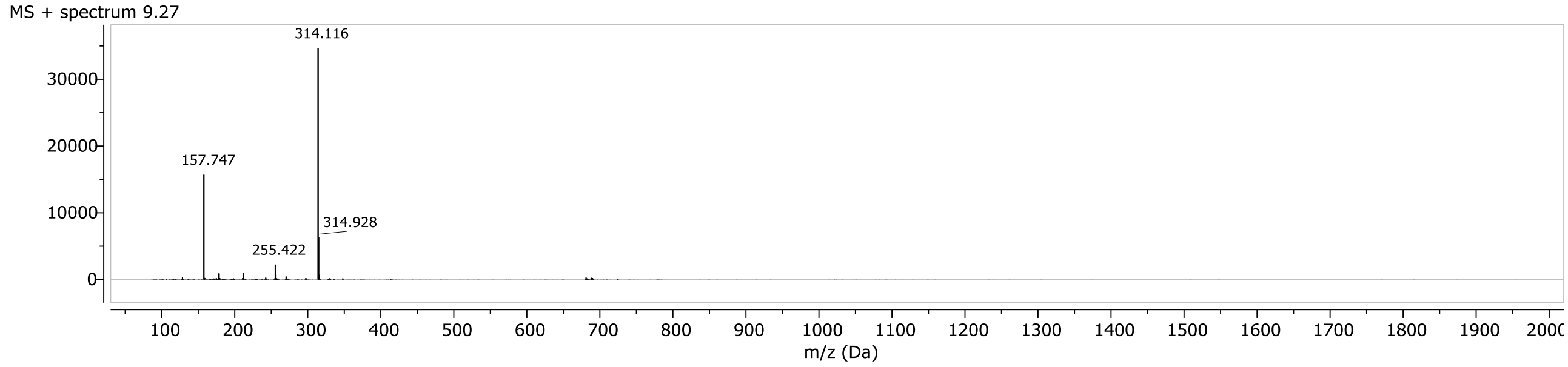

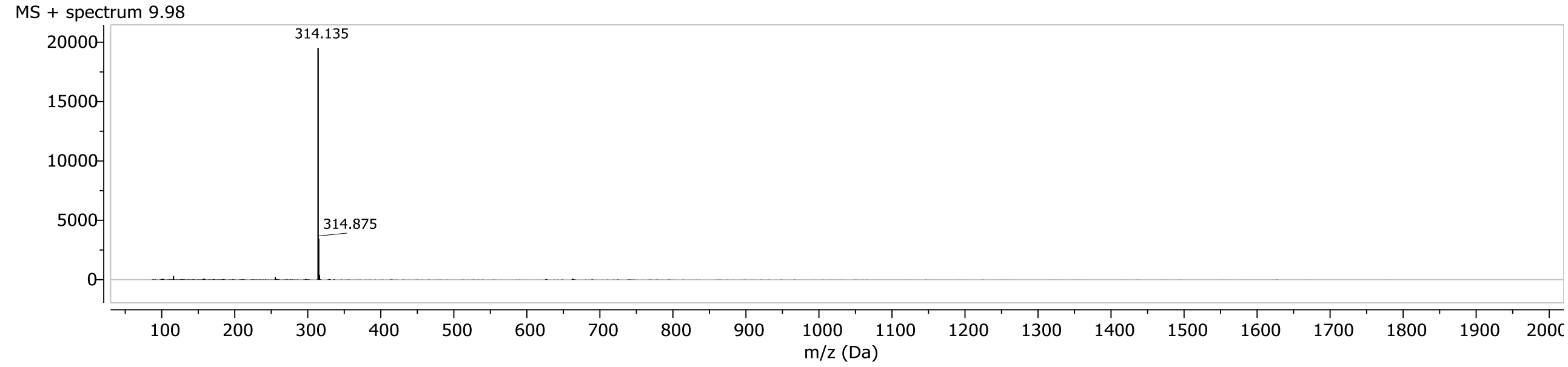

Synthesis of 2-((methyl-[1,3]dioxolo[4,5-g]quinazolinyl)methylene)hydrazine-1-carboximidamide hydrochloride 1f

Synthesis of (8-methyl-[1,3]dioxolo[4,5-g]quinazolin-6-yl)methanol 6f

The reaction was performed according to **general procedure A**, using 1-(6-aminobenzo[d][1,3]dioxol-5-yl)ethan-1-one **8f** (134 mg, 0.75 mmol, 1 eq.), triethanolamine (559 mg, 3.75 mmol, 5 eq.), eosin Y (49 mg, 0.075 mmol, 0.1 eq.), NH_4Cl (120 mg, 2.25 mmol, 3 eq.) and 1:1 CH_3CN/H_2O (5.0 mL). Chromatographic purifications did not allow the separation of the 2-unsubstituted byproduct, and the isolation of the desired product (8-methyl-[1,3]dioxolo[4,5-g]quinazolin-6-yl)methanol **6f** as a pure compound. The mixture (1:1 product:byproduct ratio), used as such in the next reaction step, appeared as a pale yellow solid (100 mg).

Reaction time: 70 hours.

TLC monitoring: eluent mixture 6:4 *n*-Hex/EtOAc.

BIOTAGE® Direct phase flash column chromatography: eluent mixture from 11% to 90% EtOAc in *n*-Hex.

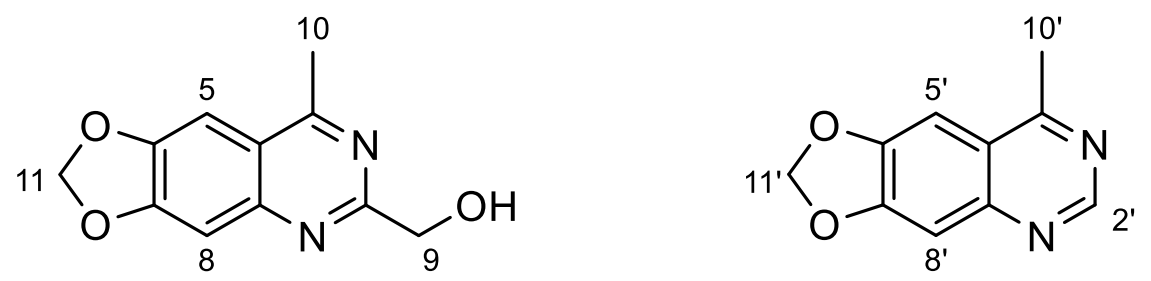

^{1}H NMR (400 MHz, $CDCl_3$) δ 8.98 (s, 1H, H2’), 7.29 (d, J = 2.8 Hz, 2H, H5, H5’), 7.26 (m, 2H+solvent peak, H8, H8’), 6.17 (s, 4H, H11, H11’), 4.86 (s, 2H, H9), 4.01 (s, 1H, -OH), 2.83 (s, 6H, H10, H10’).

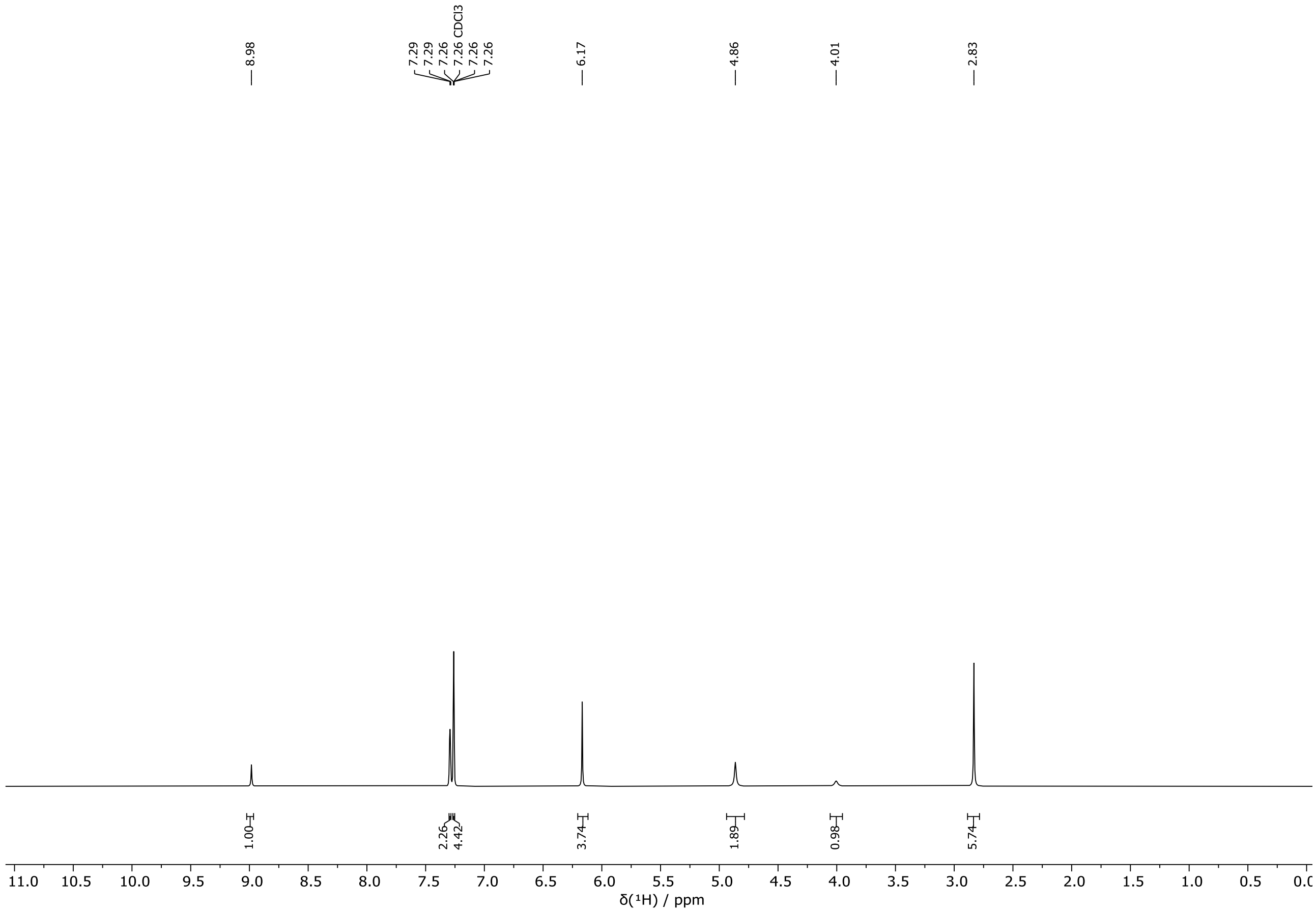

Synthesis of methyl-[1,3]dioxolo[4,5-g]quinazoline 2-carbaldehyde 7f

The reaction was performed according to **general procedure B**, using the impure (8-methyl-[1,3]dioxolo[4,5-g]quinazolin-6-yl)methanol **6f** (100 mg, 0.46 mmol, 1 eq), DMP (255 mg, 0.60 mmol, 1.3 eq.) and dry DCM (4.6 mL). After purification, pure methyl-[1,3]dioxolo[4,5-g]quinazoline 2-carbaldehyde **7f** was obtained as a white solid (39 mg, 0.18 mmol, 24% yield over two steps).

Reaction time: 2 hours.

TLC monitoring: eluent mixture 1:1 DCM/EtOAc.

BIOTAGE® Direct phase flash column chromatography: eluent mixture from 12% to 100% EtOAc in DCM.

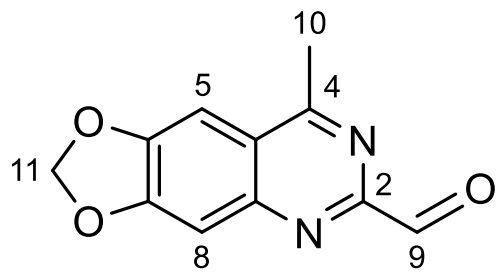

^{1}H NMR (400 MHz, $CDCl_3$) δ 10.15 (s, 1H, H9), 7.47 (s, 1H, H5), 7.37 (s, 1H, H8), 6.23 (s, 2H, H11), 2.93 (s, 3H, H10).

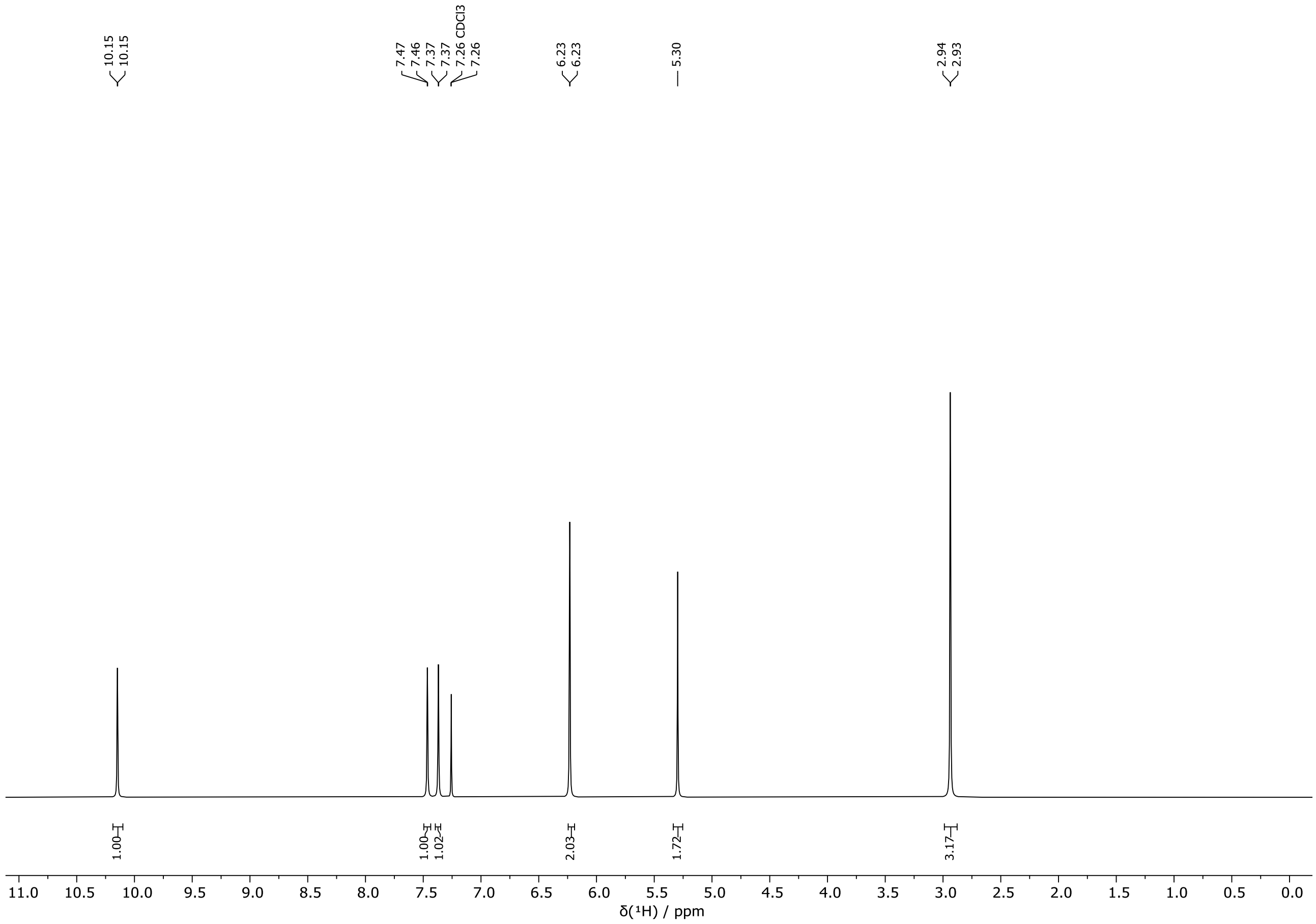

Synthesis of 2-((4-methyl-[1,3]dioxolo[4,5-g]quinazolinyl)methylene)hydrazine-1-carboximidamide hydrochloride 1f

The reaction was performed according to **general procedure C**, using methyl-[1,3]dioxolo[4,5-g]quinazoline carbaldehyde **7f** (34 mg, 0.16 mmol, 1 eq.), AG·HCl (16.6 mg, 0.15 mmol, 0.96 eq.) and EtOH (1.6 mL). Trituration was done in Et_2O (3 x 3 mL). After purification, pure target 2-((methyl-[1,3]dioxolo[4,5-g]quinazolinyl)methylene)hydrazine-1-carboximidamide hydrochloride **1f** was obtained as a pale yellow solid (27 mg, 0.087 mmol, 58% yield).

Reaction time: 7 hours, then rt overnight.

0.48:1 E/Z ratio (33%/67%).

TLC monitoring: eluent mixture 1:1 DCM/EtOAc.

BIOTAGE® Reverse phase flash column chromatography: eluent mixture from 0% to 100% CH_3CN in H_2O.

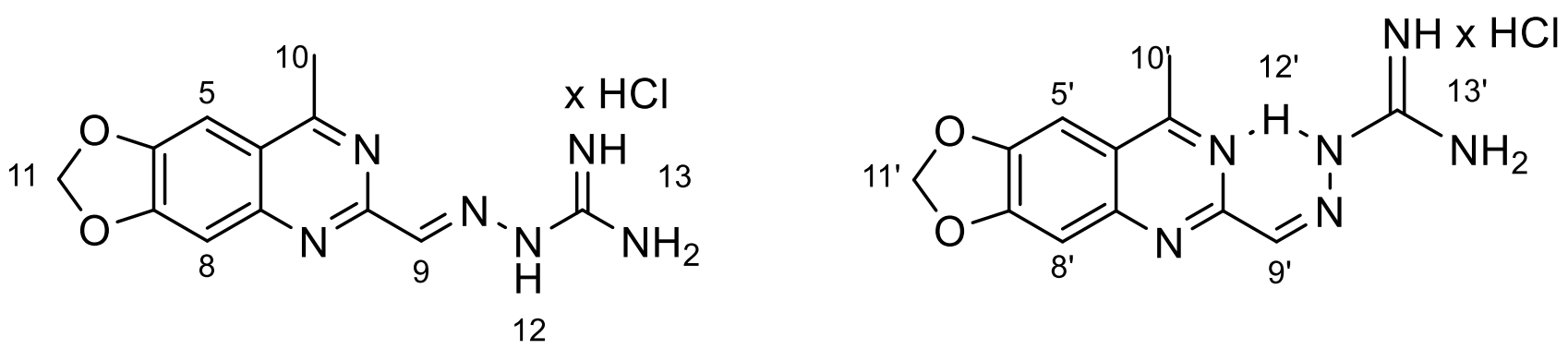

^{1}H NMR (400 MHz, DMSO-d_6) δ 14.06 (s, 1H, H12'), 12.14 (s, 1H, H12), 8.26-7.91 (brs, 8H, H13, H13'), 8.10 (s, 1H, H9), 7.60 (s, 1H, H9'), 7.58 (s, 1H, H8), 7.58 (s, 1H, H8'), 7.56 (s, 1H, H5), 7.28 (s, 1H, H5'), 6.29 (s, 2H, H11), 6.26 (s, 2H, H11'), 2.83 (s, 3H, H10), 2.78 (s, 3H, H10').

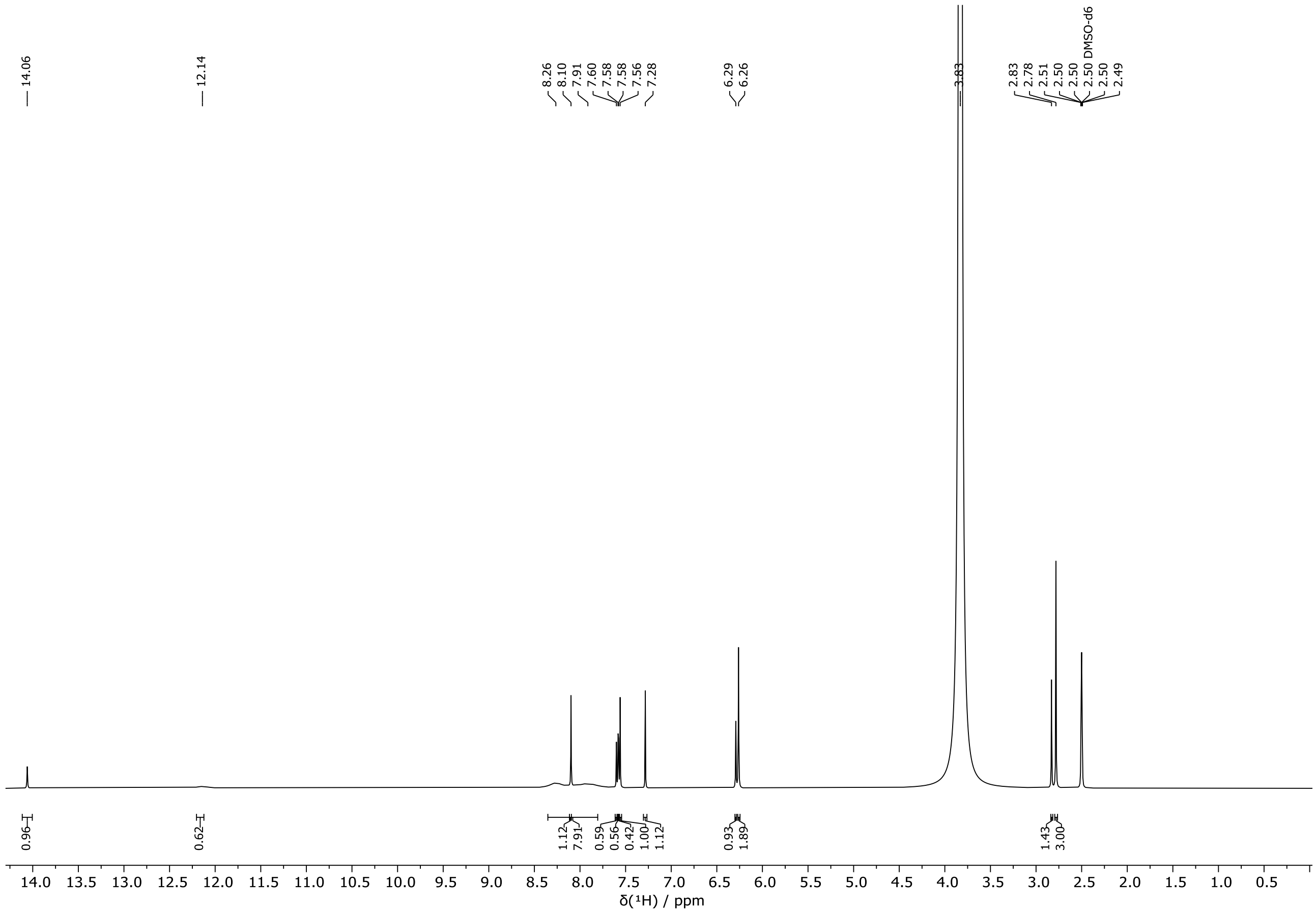

^{13}C NMR (101 MHz, DMSO-d_6) δ 167.06, 166.82, 156.11, 155.94, 155.06, 154.92, 154.67, 149.77, 148.90, 147.72, 145.51, 139.43, 120.76, 104.64, 104.37, 103.83, 103.62, 101.57, 101.39, 56.67, 22.12, 21.73.

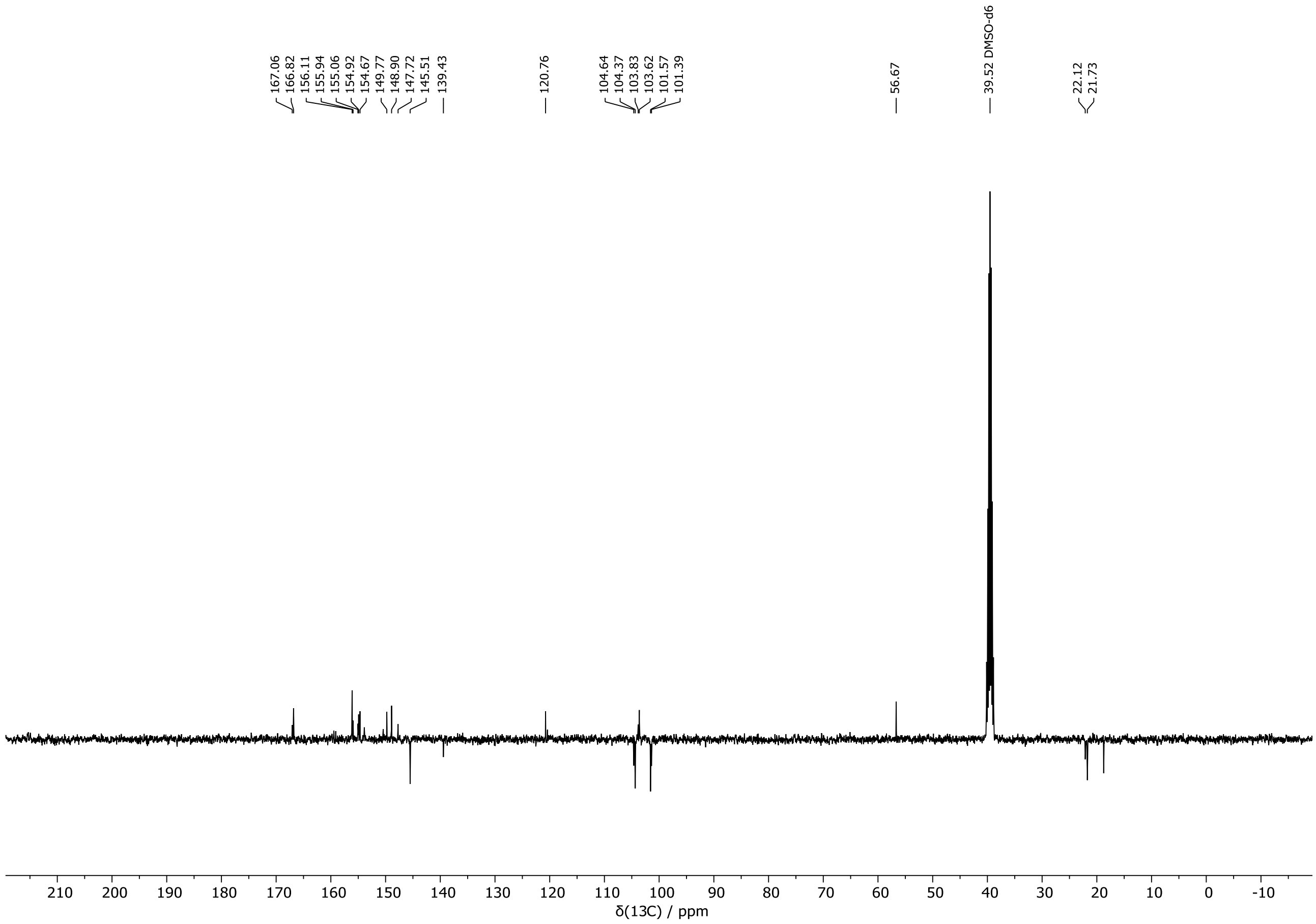

MS (ESI^+): m/z [M + H^+] 273.06. Calculated MS for $C_{12}H_{12}N_6O_2$: 273.27.

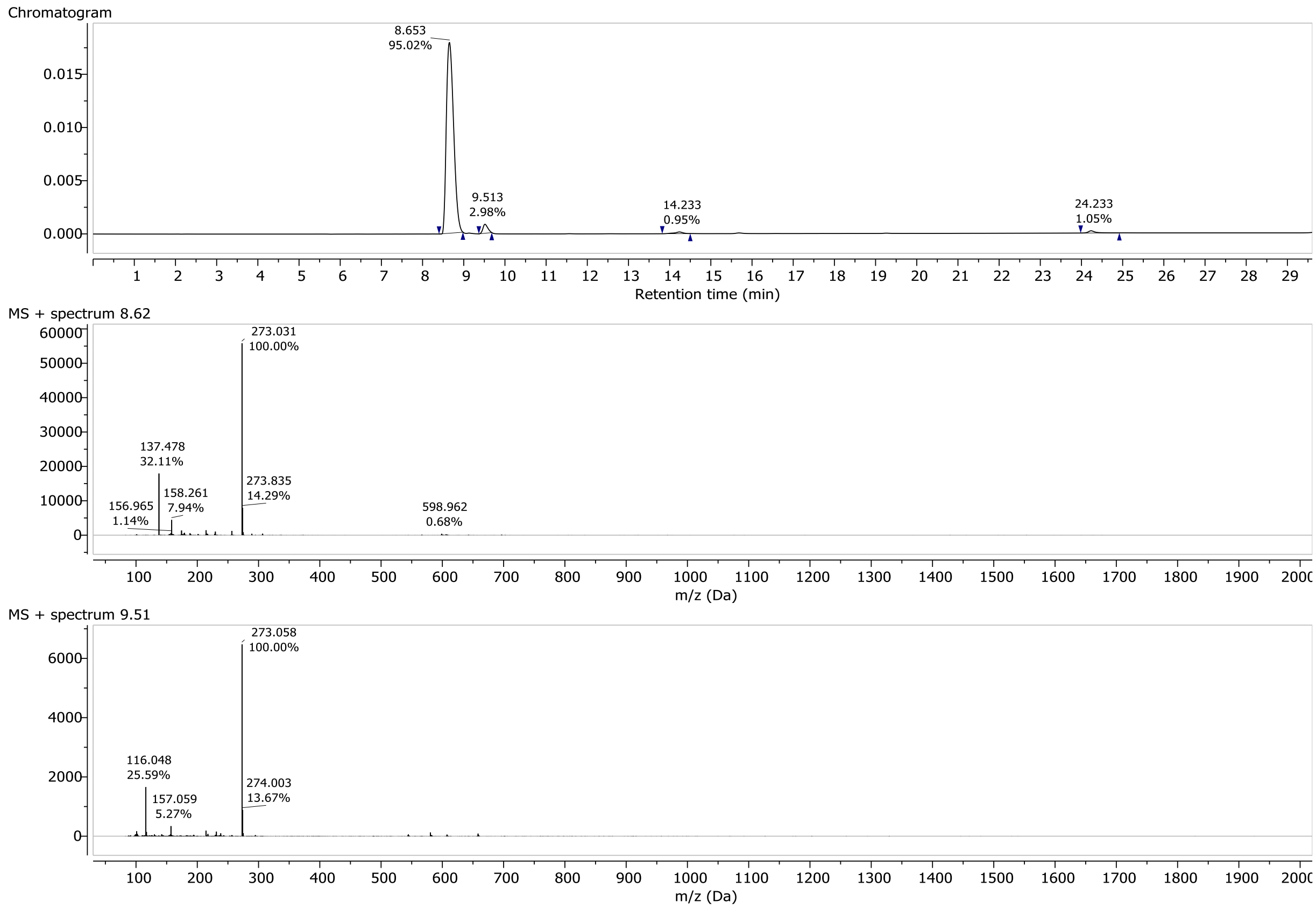

Synthesis of 2-((7-bromomethylquinazolinyl)methylene)hydrazine-1-carboximidamide hydrochloride 1g

Synthesis of (7-bromo-4-methylquinazolin-2-yl)methanol 6g

The reaction was performed according to **general procedure A**, using 1-(2-ammino-4-bromophenyl)-ethan-1-one **8g** (107 mg, 0.5 mmol, 1 eq), triethanolamine (373 mg, 2.5 mmol, 5 eq), eosin Y (32 mg, 0.05 mmol, 0.1 eq.), NH_4Cl (80 mg, 1.5 mmol, 3 eq.) and 1:1 CH_3CN/H_2O (3.3 mL). After purification, pure (7-bromo-4-methylquinazolin-2-yl)methanol **6g** was obtained as a white solid (31 mg, 0.12 mmol, 24% yield).

Reaction time: 24 hours.

TLC monitoring: eluent mixture 7:3 *n*-Hex/EtOAc.

BIOTAGE® Direct phase flash column chromatography: eluent mixture from 6% to 60% EtOAc in *n*-Hex.

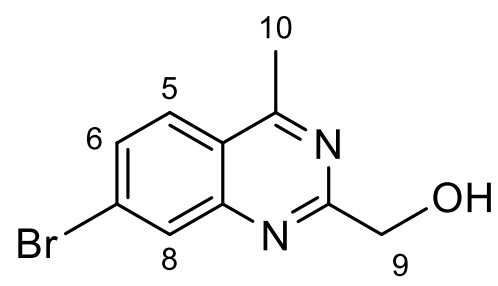

^{1}H NMR (400 MHz, $CDCl_3$) δ 8.17 (d, *J* = 1.9 Hz, 1H, H8), 7.95 (d, *J* = 8.9 Hz, 1H, H5), 7.71 (dd, *J* = 8.9, 1.9 Hz, 1H, H6), 4.94 (s, 2H, H9), 2.94 (s, 3H, H10).

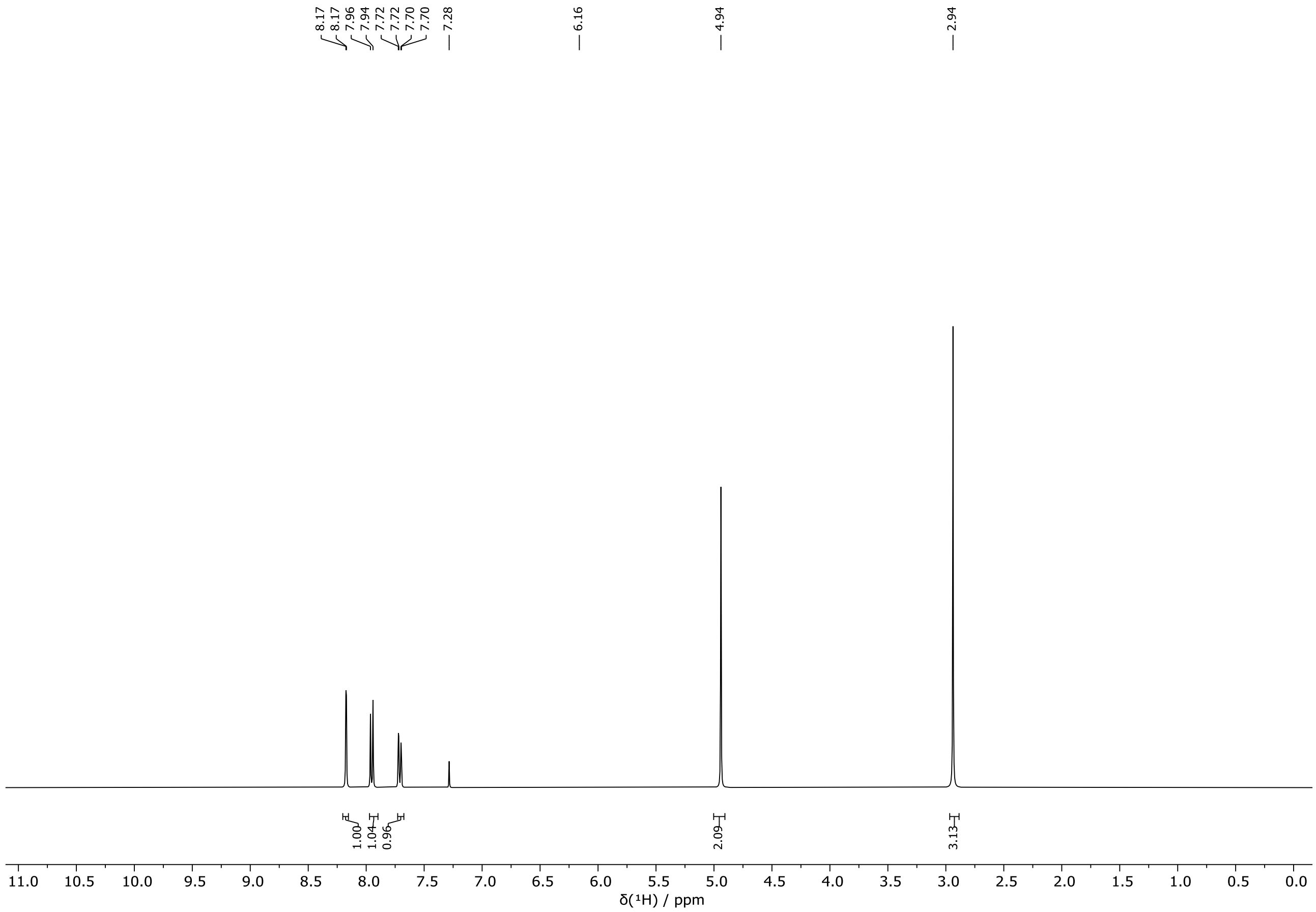

Synthesis of 7-bromo-methylquinazoline 2-carbaldehyde 7g

The reaction was performed according to **general procedure B**, using pure (7-bromo-4-methylquinazolin-2-yl)methanol **6g** (61 mg, 0.24 mmol, 1.0 eq), DMP (133 mg, 0.31 mmol, 1.3 eq.) and dry DCM (2.4 mL). After purification, pure 7-bromo-methylquinazoline 2-carbaldehyde **7g** was obtained as a white solid (29 mg, 0.12 mmol, 50% yield).

Reaction time: 1 hour.

TLC monitoring: eluent mixture 4:6 *n*-Hex/EtOAc.

BIOTAGE® Direct phase flash column chromatography: eluent mixture from 15% to 100% EtOAc in *n*-Hex.

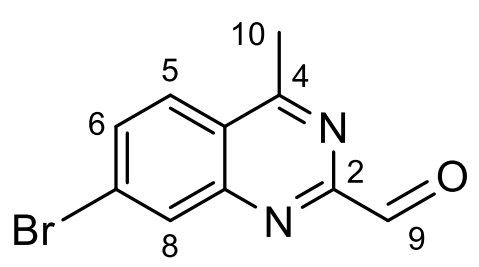

^{1}H NMR (400 MHz, $CDCl_3$) δ 10.19 (s, 1H, H9), 8.41 (s, 1H, H8), 8.05 (d, *J* = 8.8 Hz, 1H, H5), 7.88 (d, *J* = 8.9 Hz, 1H, H6), 3.05 (s, 3H, H10).

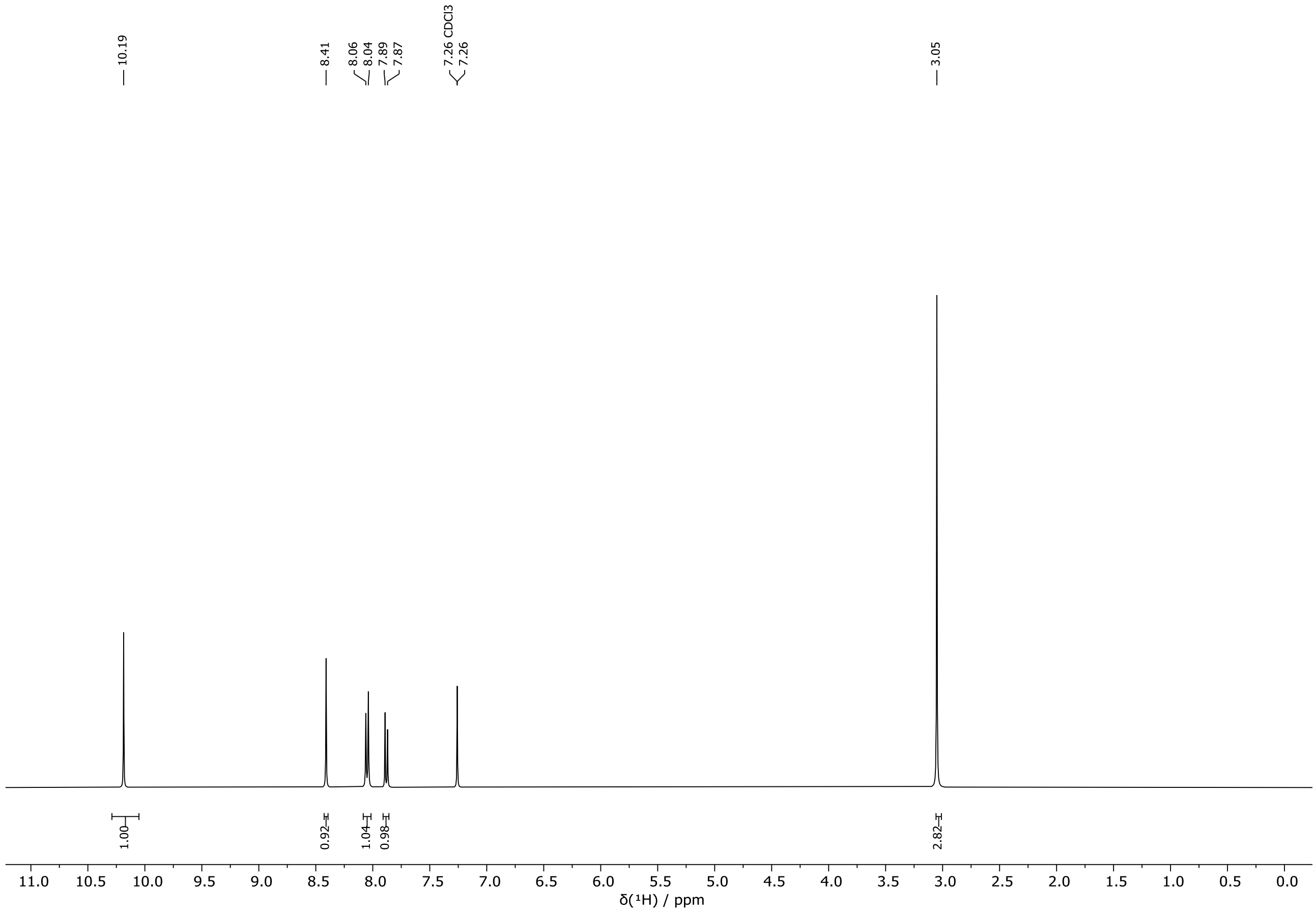

Synthesis of 2-((7-bromomethylquinazolinyl)methylene)hydrazine-1-carboximidamide hydrochloride 1g

The reaction was performed according to **general procedure C**, using 7-bromo-methylquinazoline 2-carbaldehyde **7g** (27 mg, 0.11 mmol, 1 eq.), AG·HCl (11.3 mg, 0.10 mmol, 0.95 eq.) and EtOH (1.1 mL). Trituration was done in (i-Pr)$_2$O (3 x 2.5 mL). Pure target 2-((6-bromomethylquinazolinyl)methylene)hydrazine-1-carboximidamide hydrochloride **1g** was obtained as a yellow solid (35 mg, 0.10 mmol, quantitative yield) without further purification.

1:0.28 E/Z ratio (78%/22%).

Reaction time: 7 hours, then rt overnight.

TLC monitoring: eluent mixture 1:1 DCM/EtOAc.

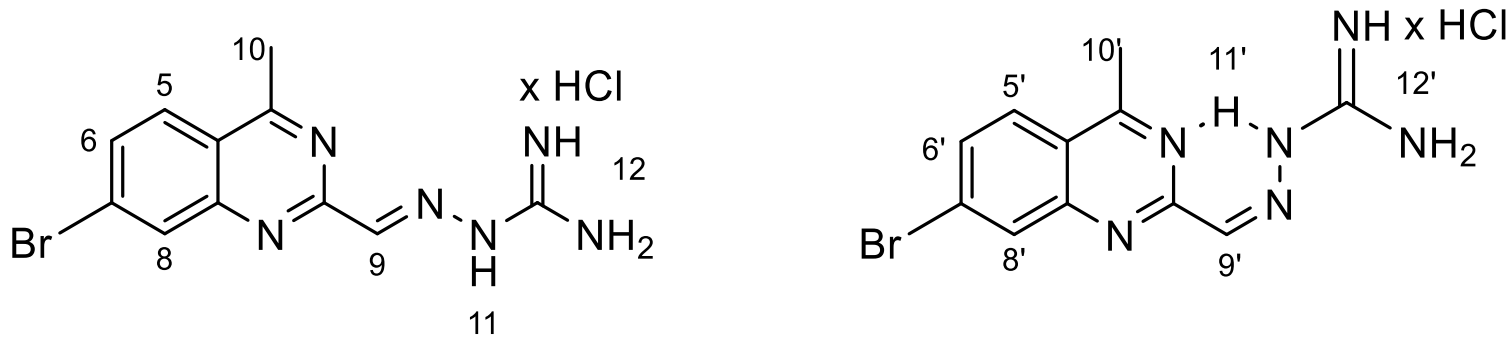

^{1}H NMR (400 MHz, DMSO-d_6) δ 14.00 (s, 1H, H11’), 12.55 (s, 1H), 8.87 (s, 1H, H8’), 8.57 (brs, 4H, H12’), 8.35 (s, 1H, H9’), 8.31 (d, J = 8.8 Hz, H5’), 8.28 (d, J = 8.8 Hz, 1H, H5), 8.25 (d, J = 2.3 Hz, 1H, H8), 8.03 (d, J = 8.8 Hz, H6’), 7.98-7.83 (m, 5H, H6, H12), 3.02 (s, 3H, H10’), 2.96 (s, 3H, H10).

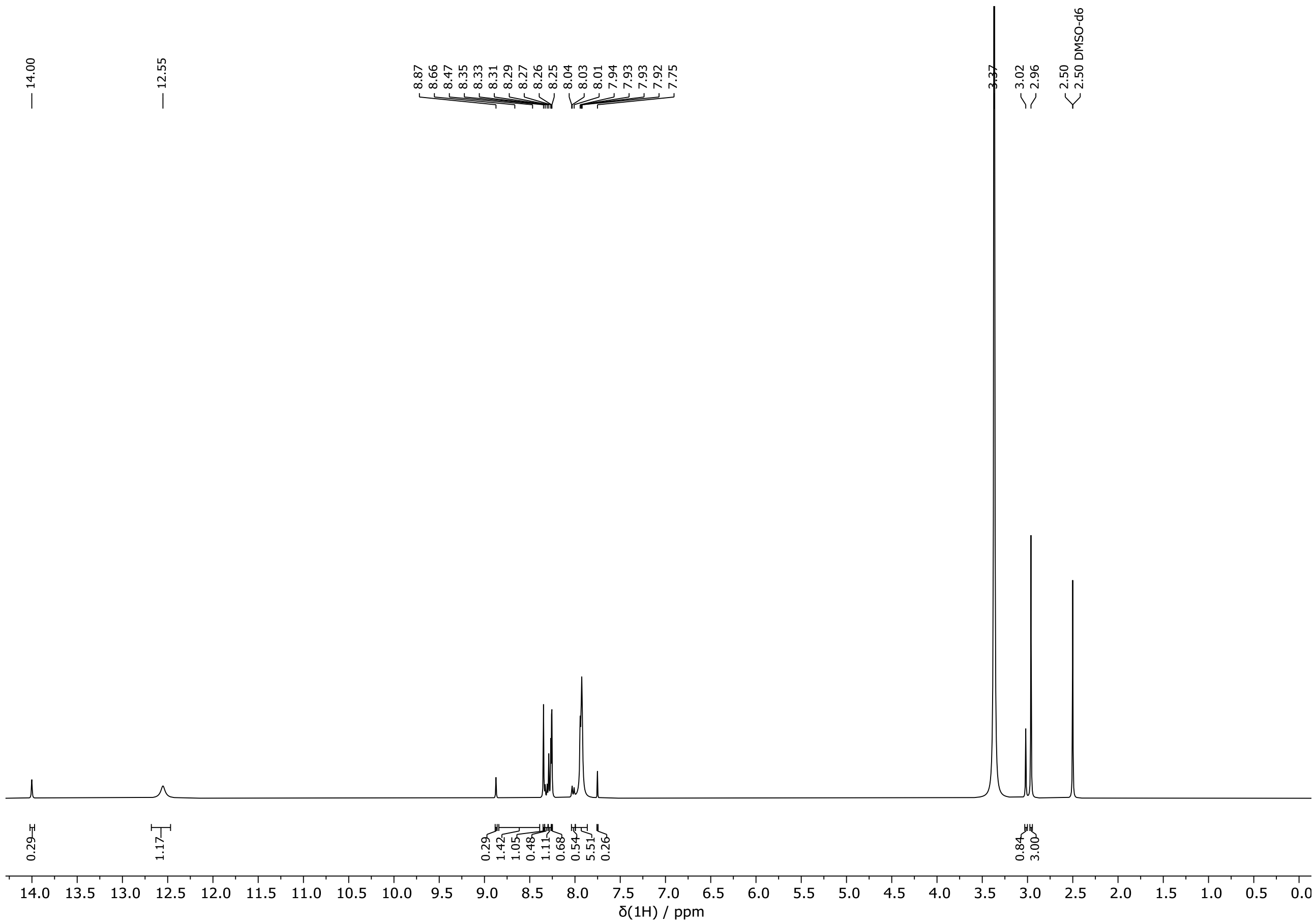

^{13}C NMR (101 MHz, DMSO-d_6) δ 171.04, 169.91, 156.78, 155.68, 155.48, 155.30, 149.94, 146.11, 138.15, 132.60, 131.72, 130.71, 130.34, 128.34, 128.12, 128.01, 121.90, 121.43, 21.72, 21.64.

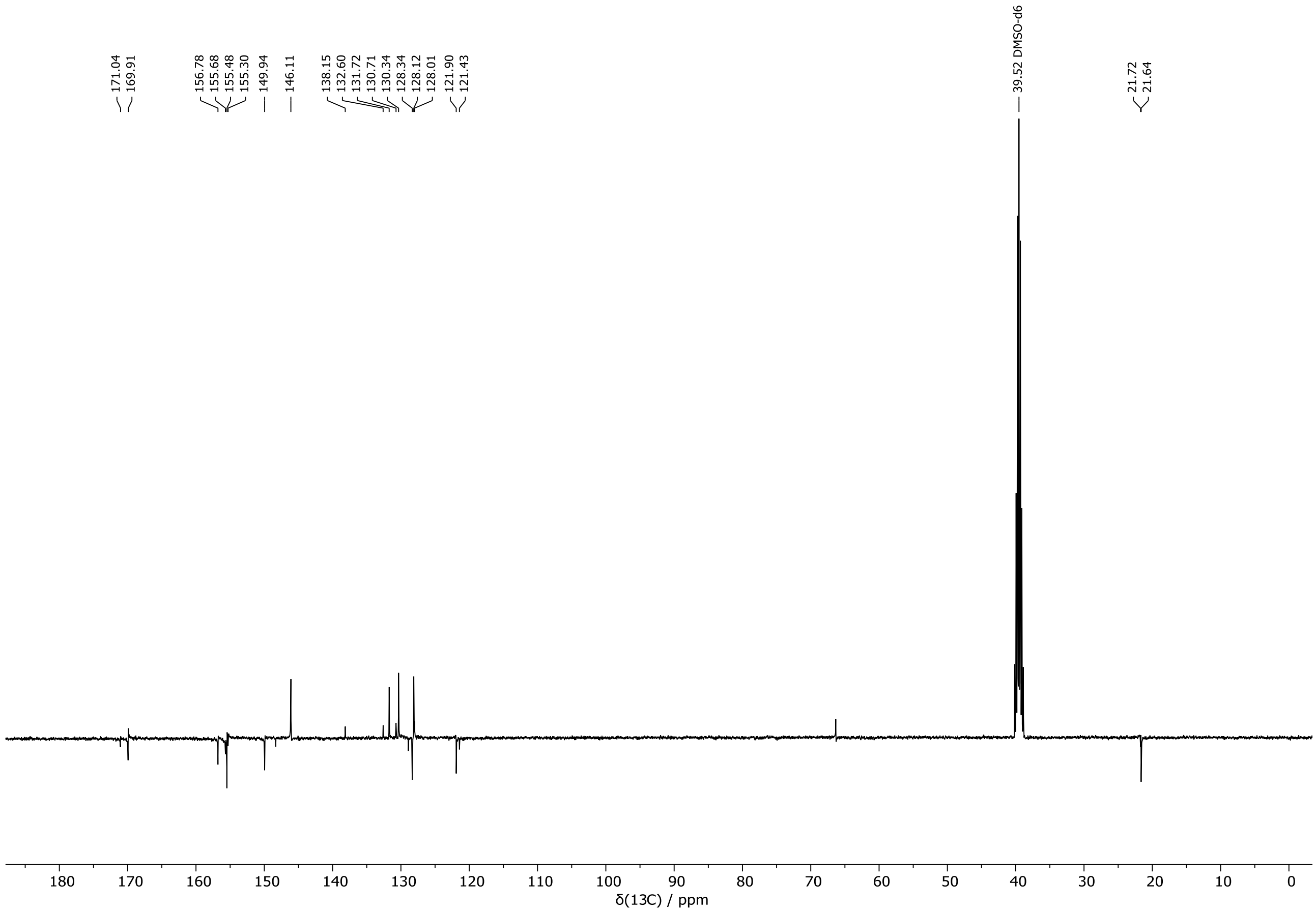

MS (ESI$^+$): m/z [M + H$^+$] 309.01. Calculated MS for $C_{15}H_{20}N_7O$: 308.16.

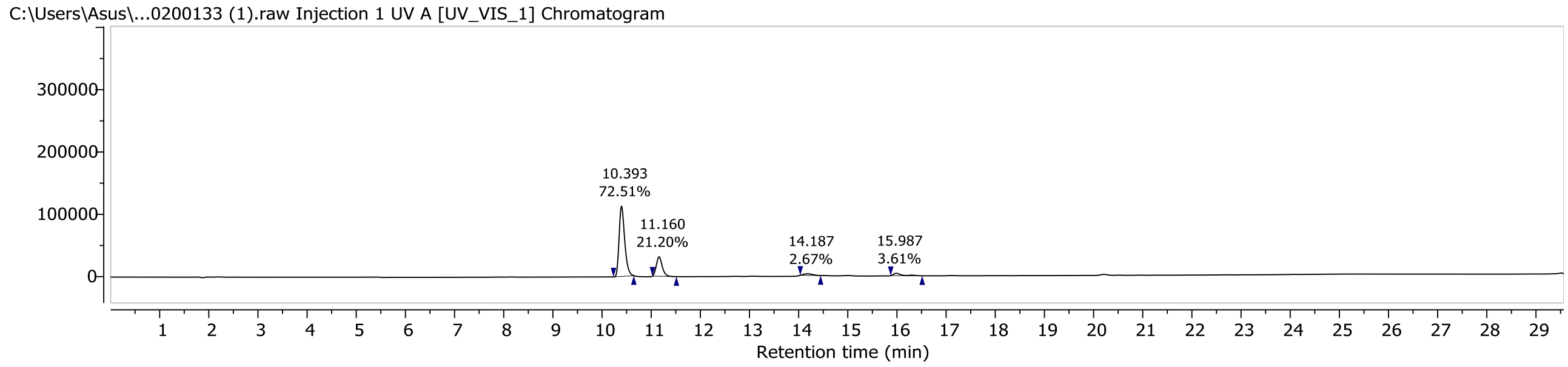

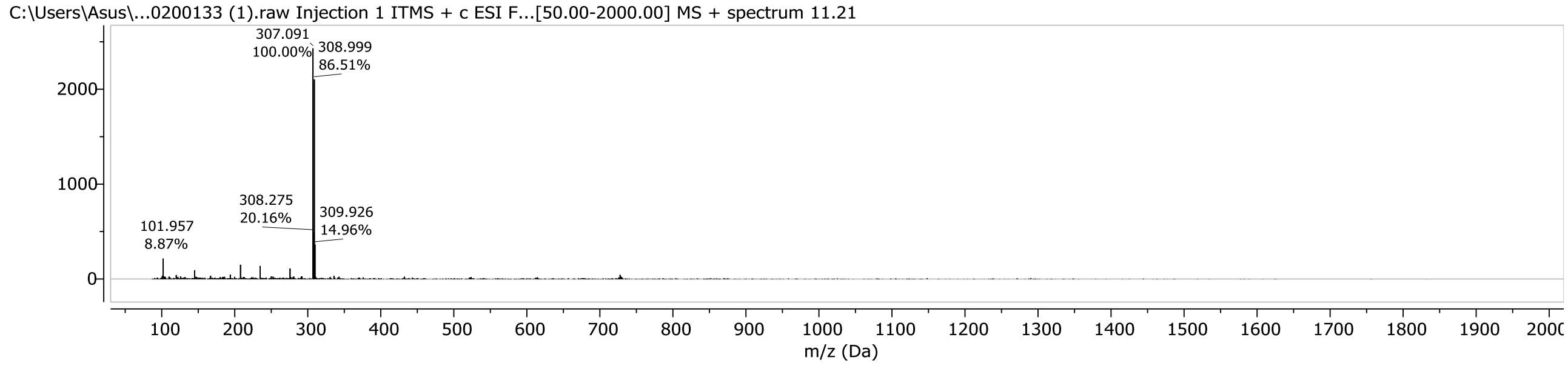

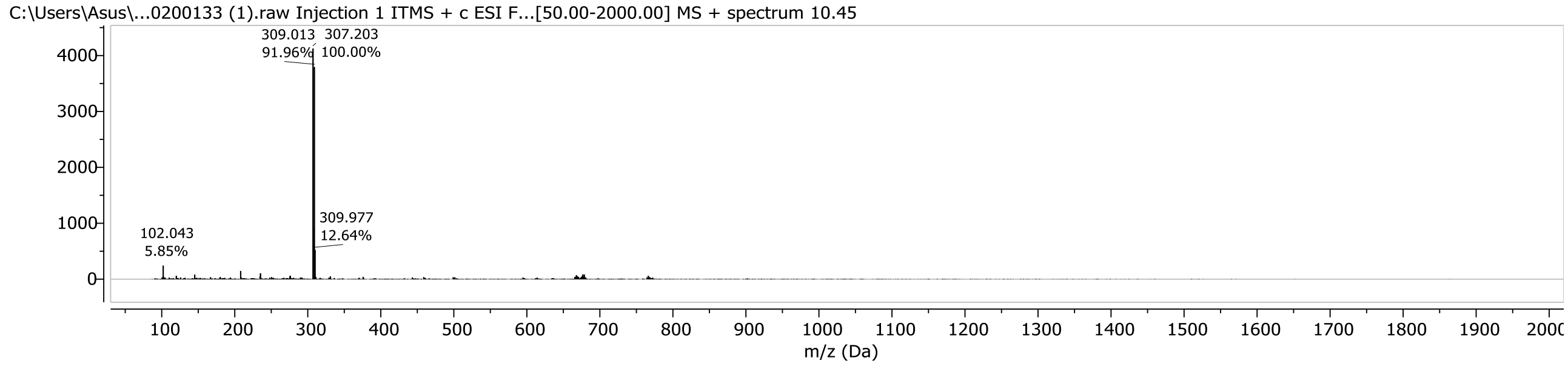

Synthesis of isomeric GMQ-like 4-GH quinazolines 2a-f

Synthesis of 4-((2-methylquinazolinyl)methylene)hydrazine-1-carboximidamide hydrochloride 2a

Synthesis of 2,4-dimethylquinazoline 9a

The reaction was performed according to **general procedure D**, using NH_4OAc (462 mg, 6.0 mmol, 3.0 eq), 1-(2-aminophenyl)ethan-1-one **8a** (0.260 mL, 2.0 mmol, 1.0 eq) and acetylacetone (0.245 mL, 2.4 mmol, 1.2 eq). Pure 2,4-dimethylquinazoline **9a** was obtained as a yellow oil (147 mg, 0.79 mmol, 40% yield).

Reaction time: 7 hours.

TLC monitoring: eluent mixture 1:1 DCM/EtOAc.

Direct phase flash column chromatography: eluent mixture from 9:1 to 2:8 DCM/EtOAc.

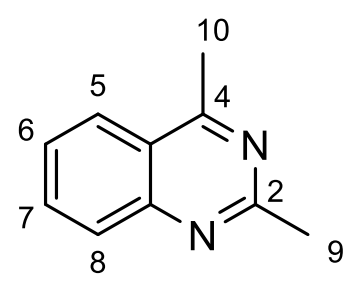

^{1}H-NMR (400 MHz, $CDCl_3$) δ 8.02 (d, *J* = 8.3 Hz, 1H, H5), 7.91 (d, *J* = 8.3 Hz, 1H, H8), 7.85 – 7.76 (m, 1H, H7), 7.53 (t, *J* = 7.6 Hz, 1H, H6), 2.90 (s, 3H, H9), 2.83 (s, 3H, H10).

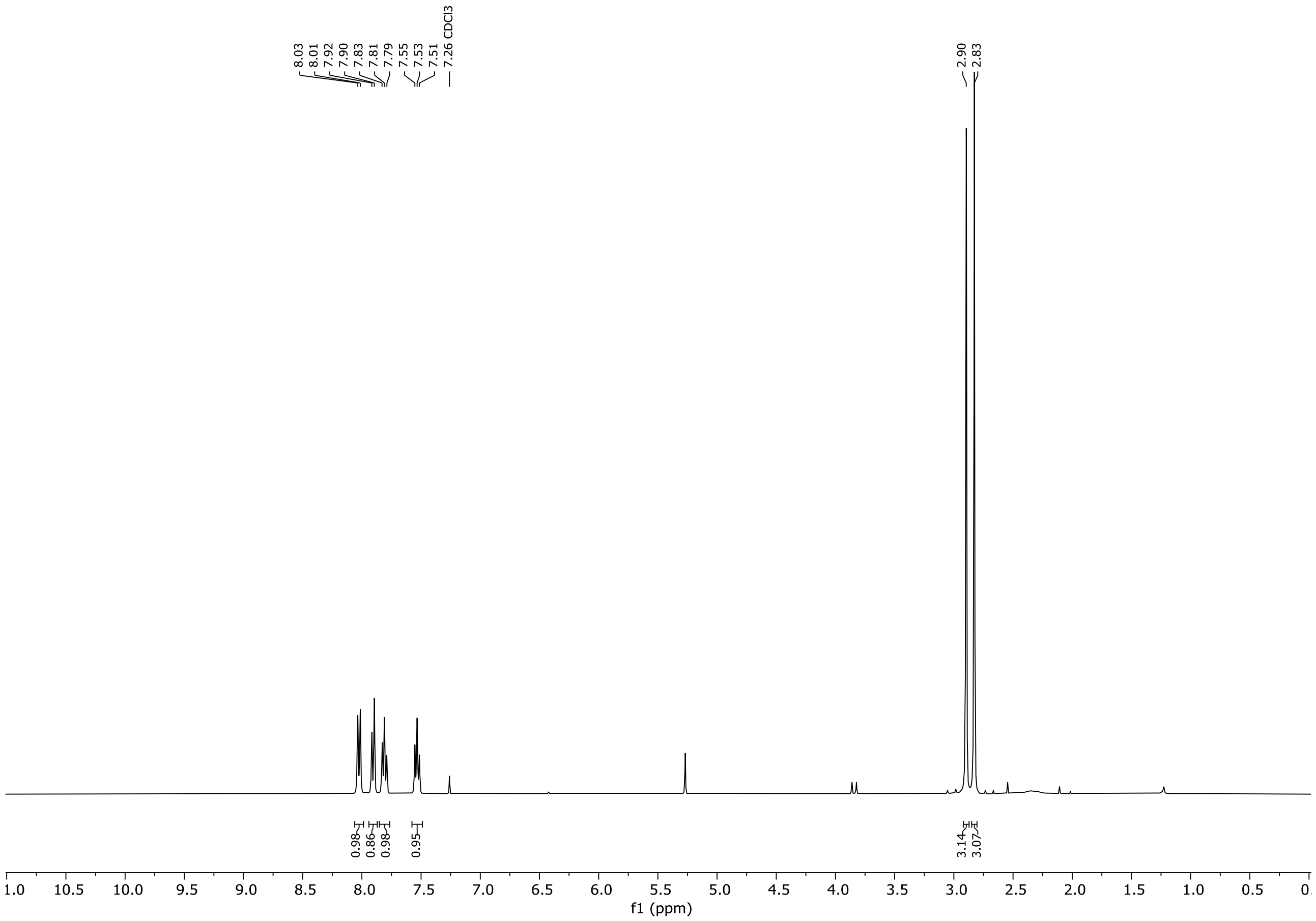

^{13}C-NMR (101 MHz, $CDCl_3$) δ 168.6, 163.9, 150.3, 134.1, 128.6, 127.1, 125.4, 122.6, 26.8, 22.1.

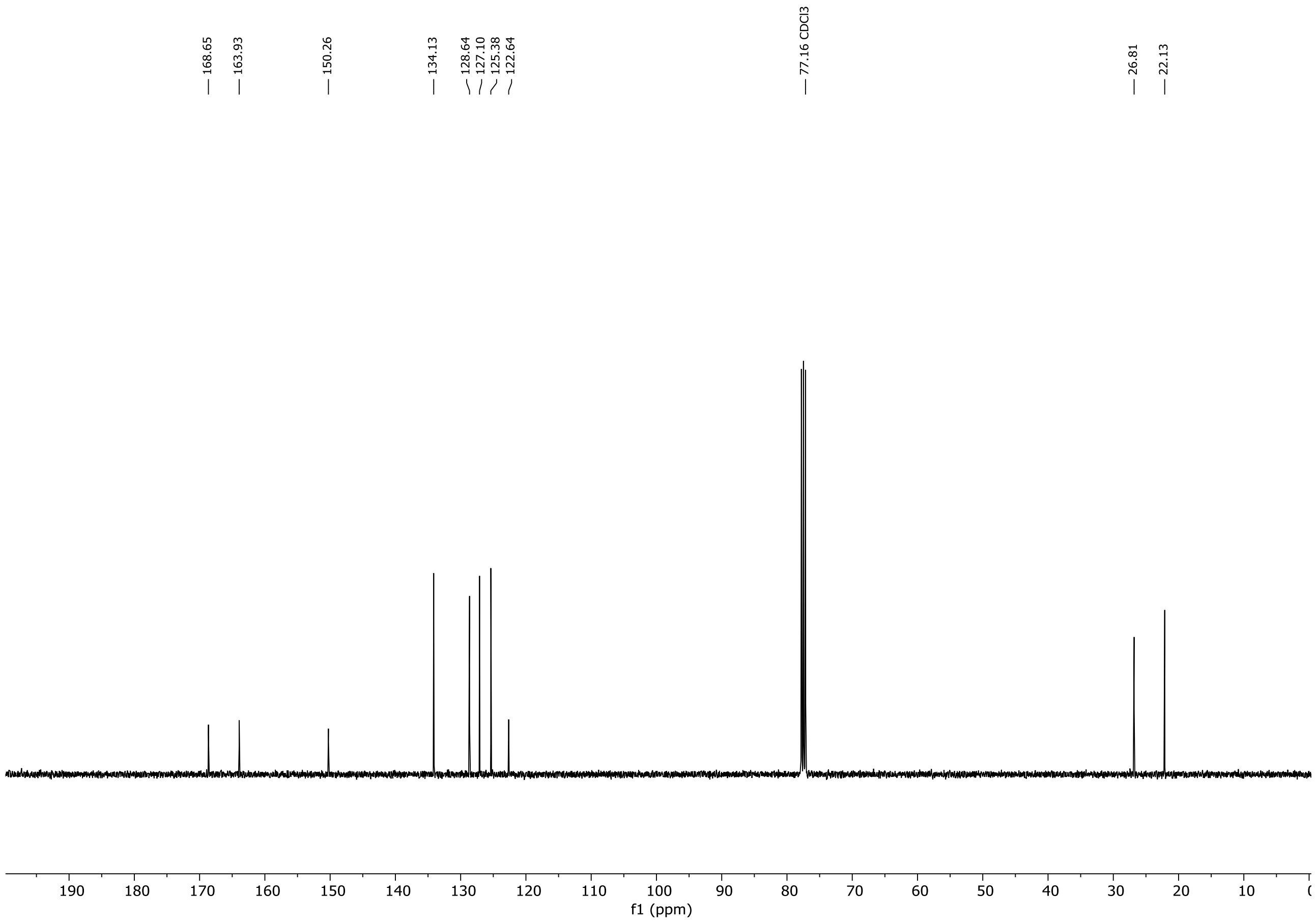

Synthesis of 2-methylquinazoline-4-carbaldehyde 10a

The reaction was performed according to **general procedure E**, using 2,4-dimethylquinazoline **9a** (0.14 mL, 0.948 mmol, 1.0 eq), SeO_2 (210 mg, 1.89 mmol, 2.0 eq.) and 1,4-dioxane (4.7 mL). Washing after Celite pad filtration was done using DCM (125 mL). After purification, pure 2-methylquinazoline-4-carbaldehyde **10a** was obtained as a yellow solid (74 mg, 0.43 mmol, 45% yield).

Reaction time: 3 hours.

TLC monitoring: eluent mixture 1:1 DCM/EtOAc.

Direct phase flash column chromatography: eluent mixture 8:2 DCM/EtOAc.

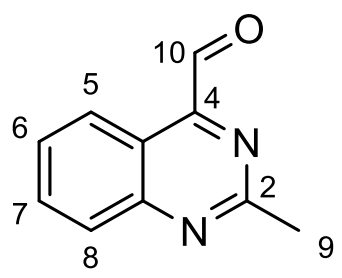

^{1}H-NMR (400 MHz, $CDCl_3$) δ 10.28 (s, 1H, H10), 9.09 – 8.93 (m, 1H, H5), 8.03 (d, *J* = 7.6 Hz, 1H, H8), 7.92 (ddd, *J* = 8.5, 6.9, 1.4 Hz, 1H, H7), 7.69 (ddd, *J* = 8.5, 6.9, 1.4 Hz, 1H, H6), 2.99 (s, 3H, H9).

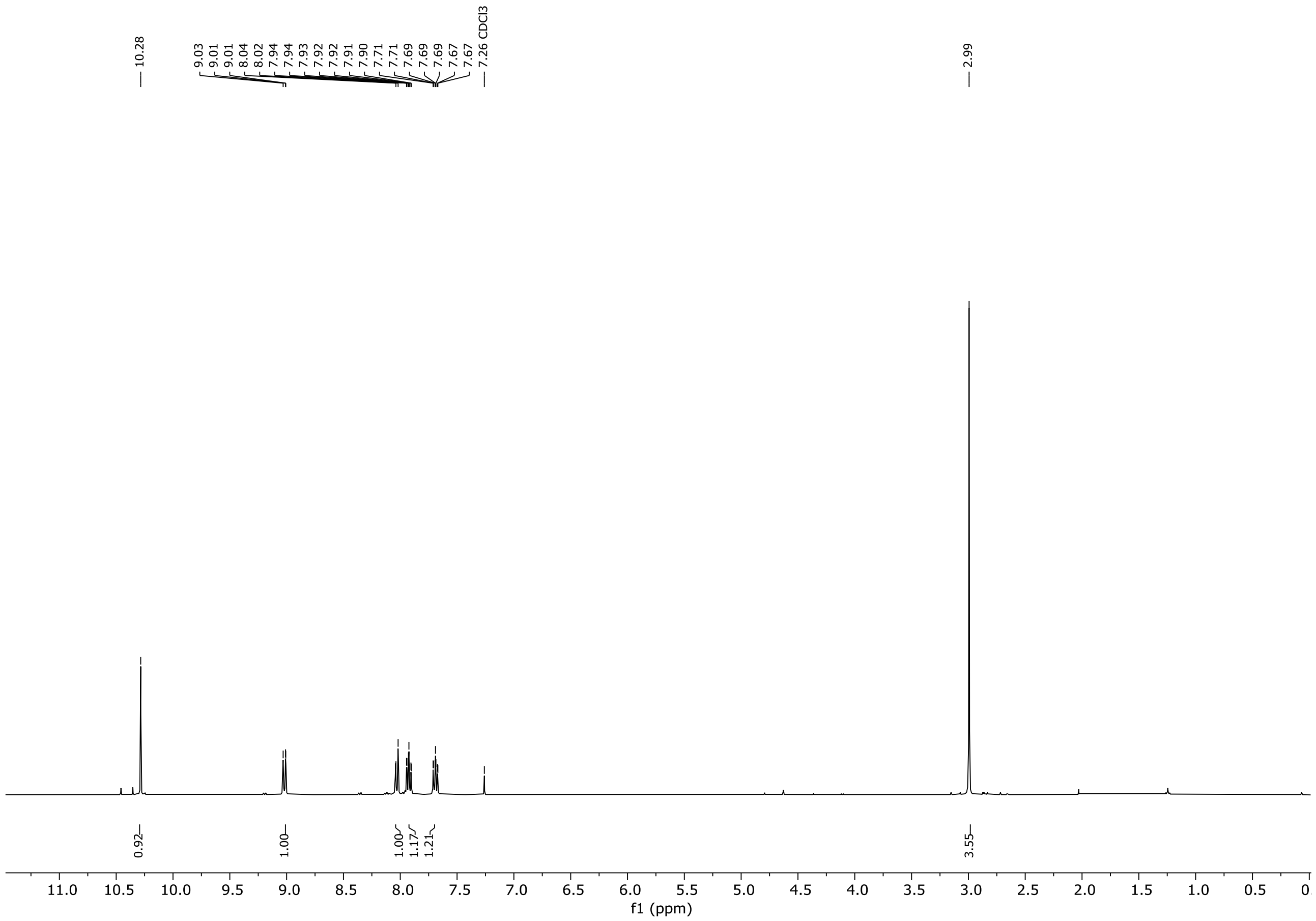

^{13}C-NMR (101 MHz, $CDCl_3$) δ 195.5, 164.5, 155.6, 152.9, 134.6, 129.3, 128.3, 125.5, 119.1, 26.2.

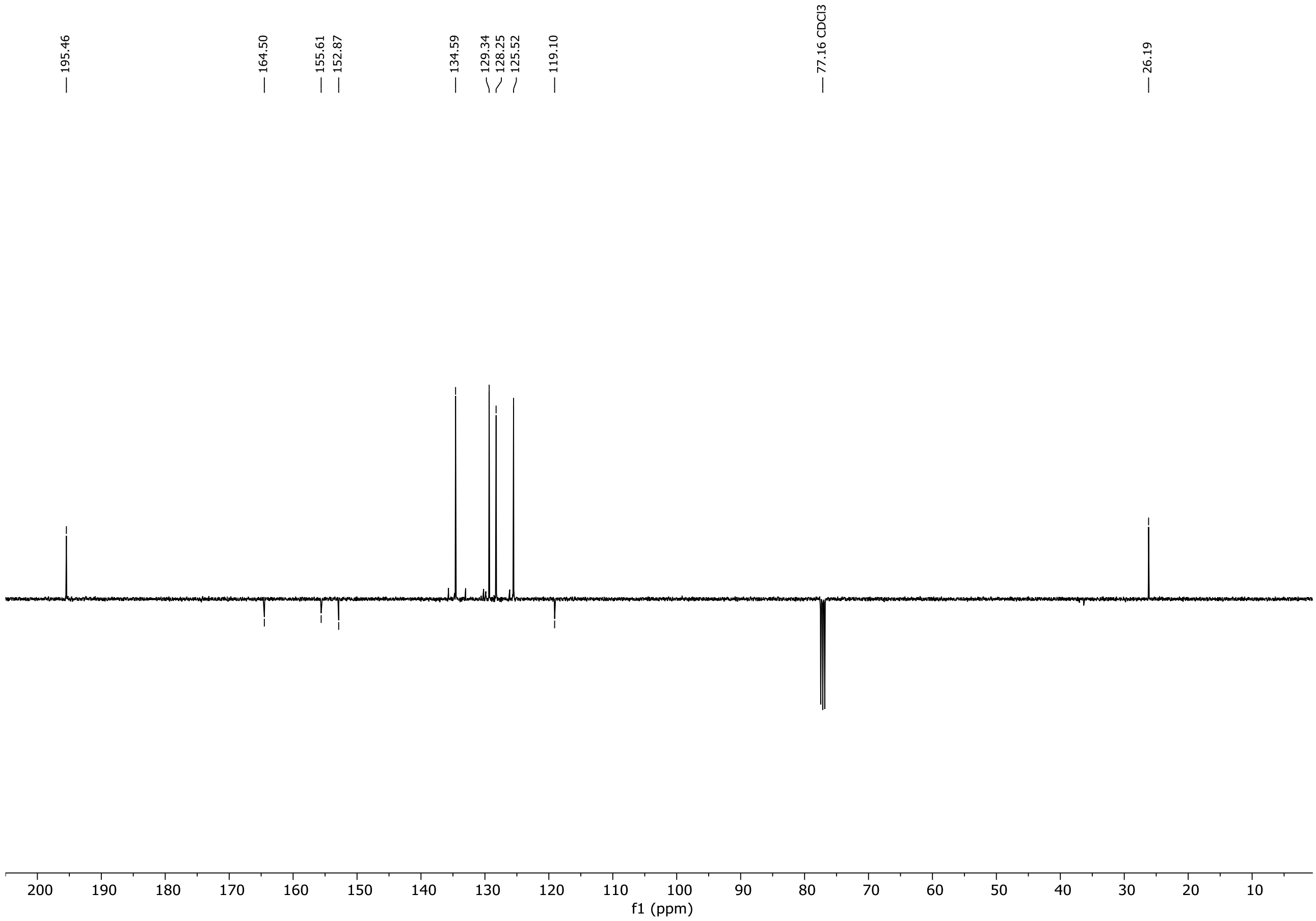

Synthesis of 4-((2-methylquinazolinyl)methylene)hydrazine-1-carboximidamide hydrochloride 2a

The reaction was performed according to **general procedure C**, using 2-methylquinazoline-4-carbaldehyde **10a** (25 mg, 0.145 mmol, 1.0 eq), AG.HCl (15 mg, 0.142 mmol, 0.98 eq) and EtOH (1.5 mL). Trituration was done in Et_2O (2 x 2.5 mL) and DCM (2.5 mL). After purification, pure target 2-((2-methylquinazolin-4-yl)methylene)hydrazine-1-carboximidamide hydrochloride **2a** was obtained as a yellow solid (24 mg, 0.091 mmol, 63% yield).

1:0.08 E/Z ratio (93%/7%).

Reaction time: 7 hours, then rt overnight.

TLC monitoring: eluent mixture 1:1 DCM/EtOAc.

BIOTAGE® Reverse phase flash column chromatography: eluent mixture from 0% to 100% CH_3CN in H_2O.

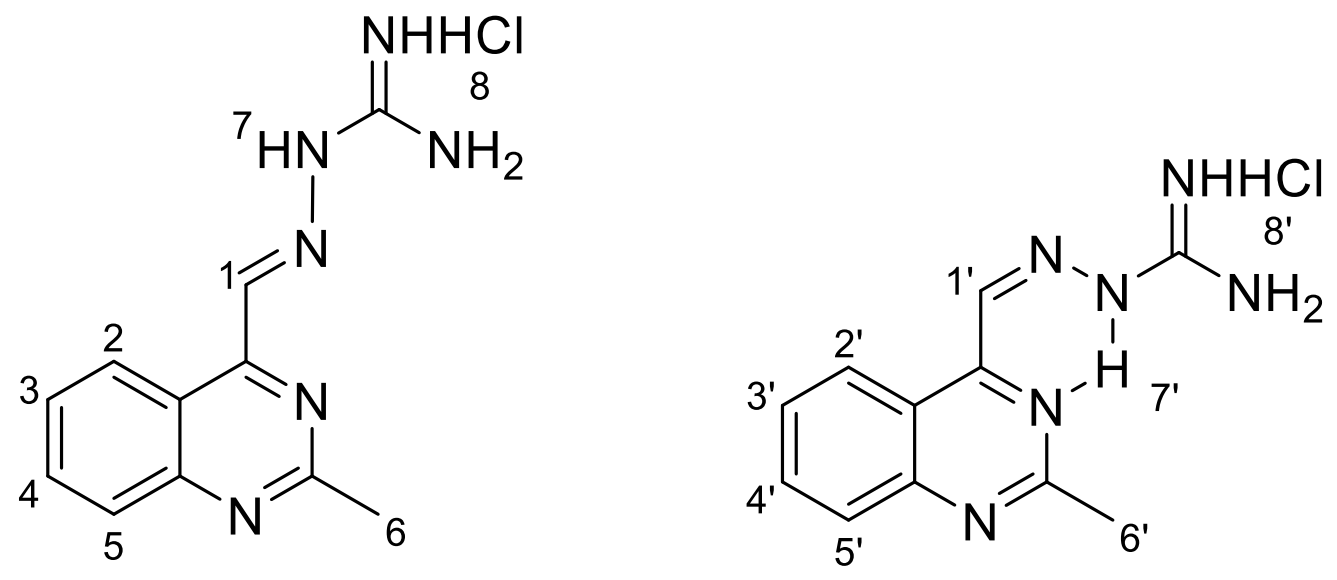

^{1}H NMR (400 MHz, DMSO-d_6) δ (ppm) 12.65 (s, 1H, H7), 8.97 (d, *J* = 8.4 Hz, 1H, H2), 8.74 (d, *J* = 8.5 Hz Hz, 1H, H2’), 8.66 (s, 1H, H1’), 8.55 (s, 1H, H1), 8.50 (bs, 4H, H8’), 7.96-8.04 (m, 8H, H3, H5, H8, H3’, H5’), 7.85 – 7.76 (m, 1H, H4’), 7.73 (ddd, *J* = 8.4, 6.5, 1.6 Hz, 1H, H4), 2.97 (s, 3H, H6’), 2.80 (s, 3H, H6).

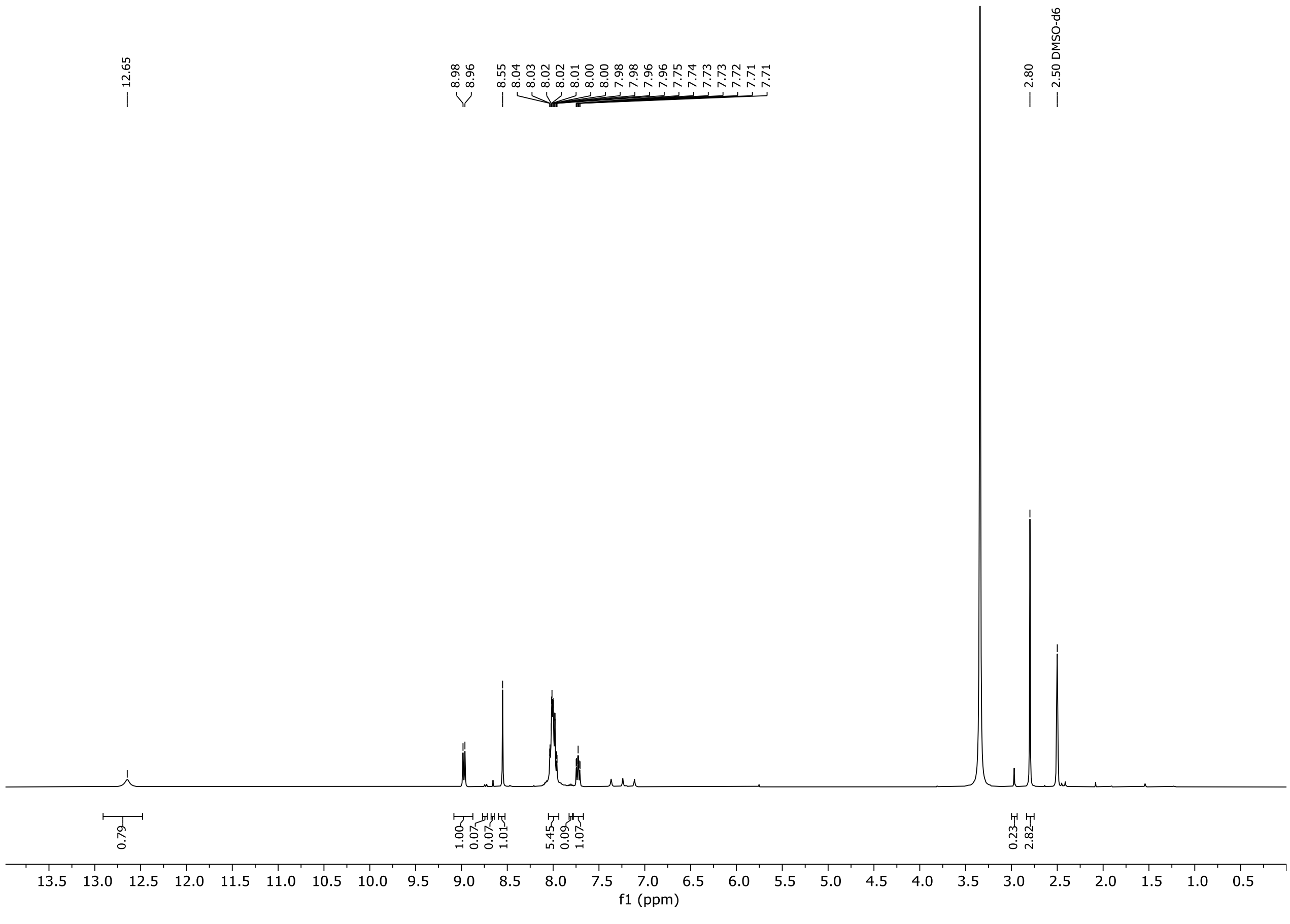

^{13}C-NMR (101 MHz, DMSO-d_6) δ 163.1, 158.0, 155.5, 151.2, 147.7, 134.3, 128.3, 127.7, 127.0, 119.4, 25.7. Only the signals for the major form are reported.

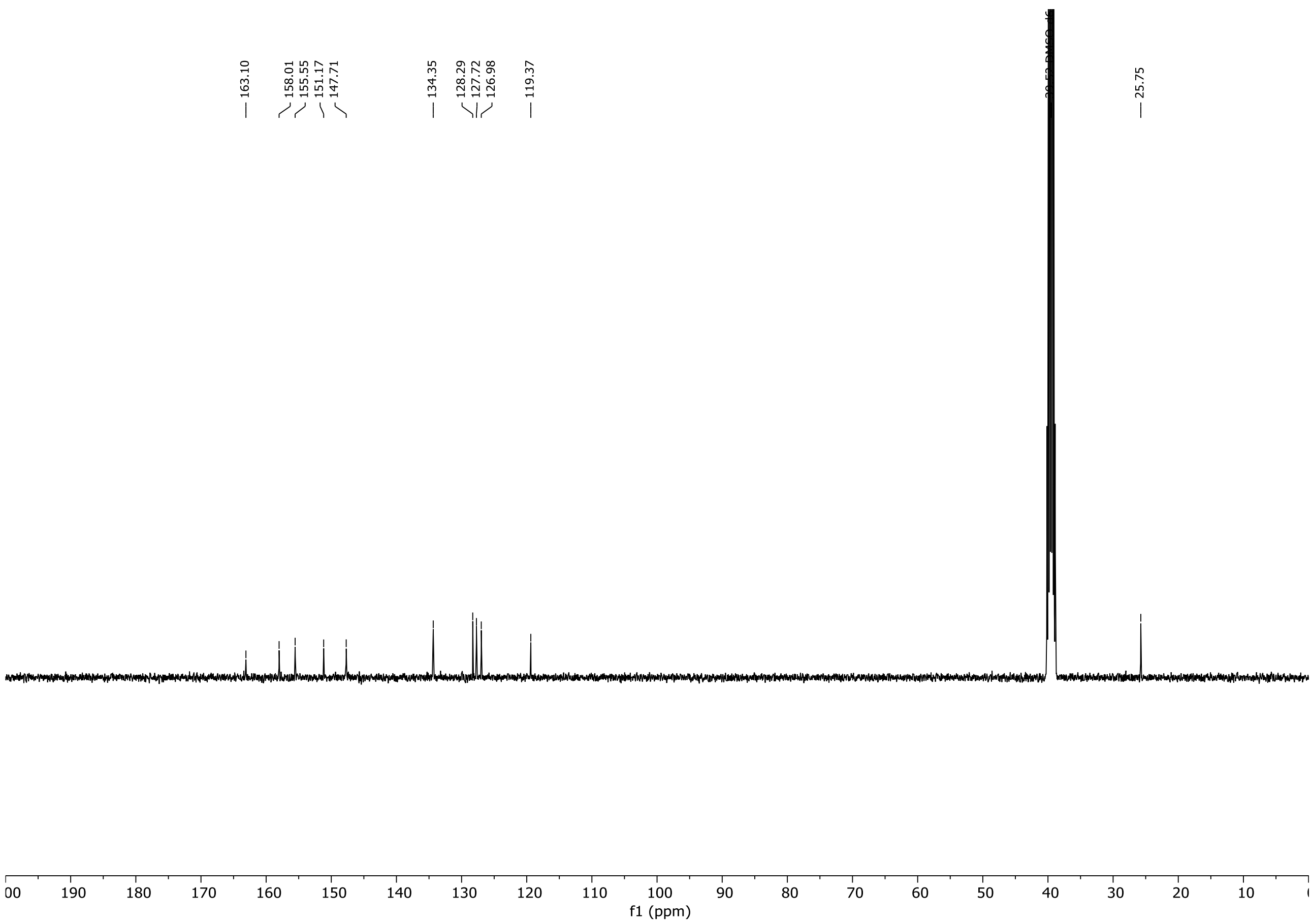

MS (ESI^+): m/z [$M + H^+$] 228.94. Calculated MS for $C_{11}H_{13}N_6$: 229.12.

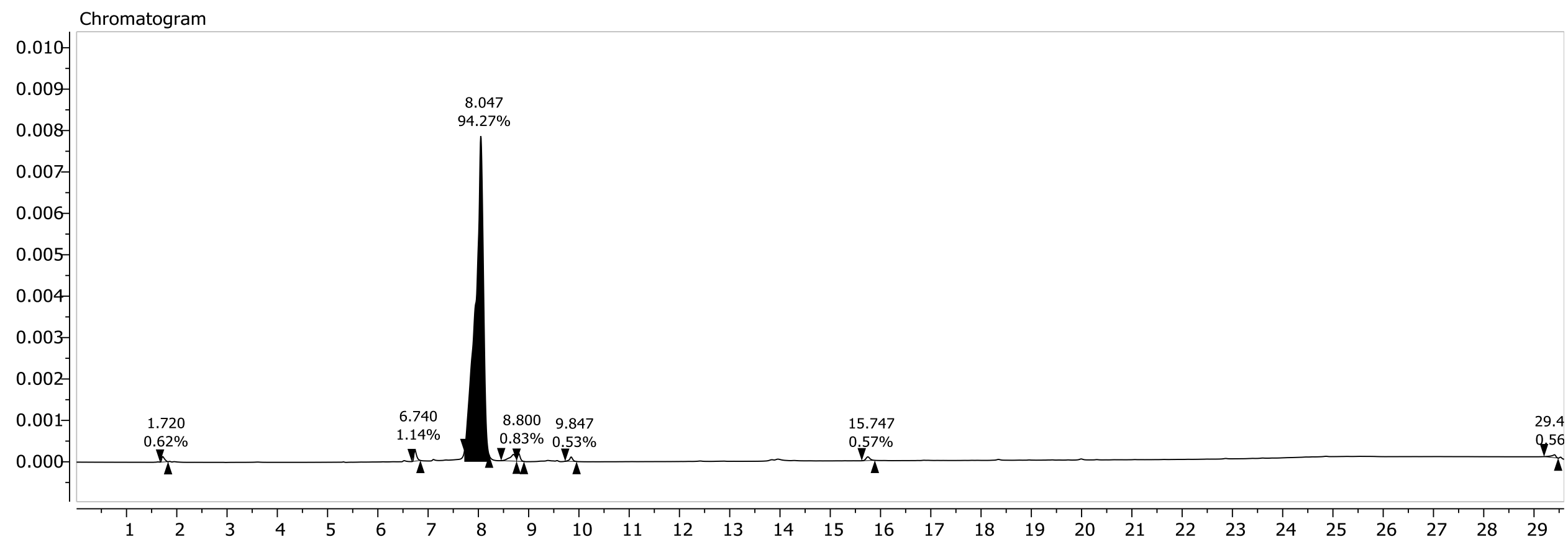

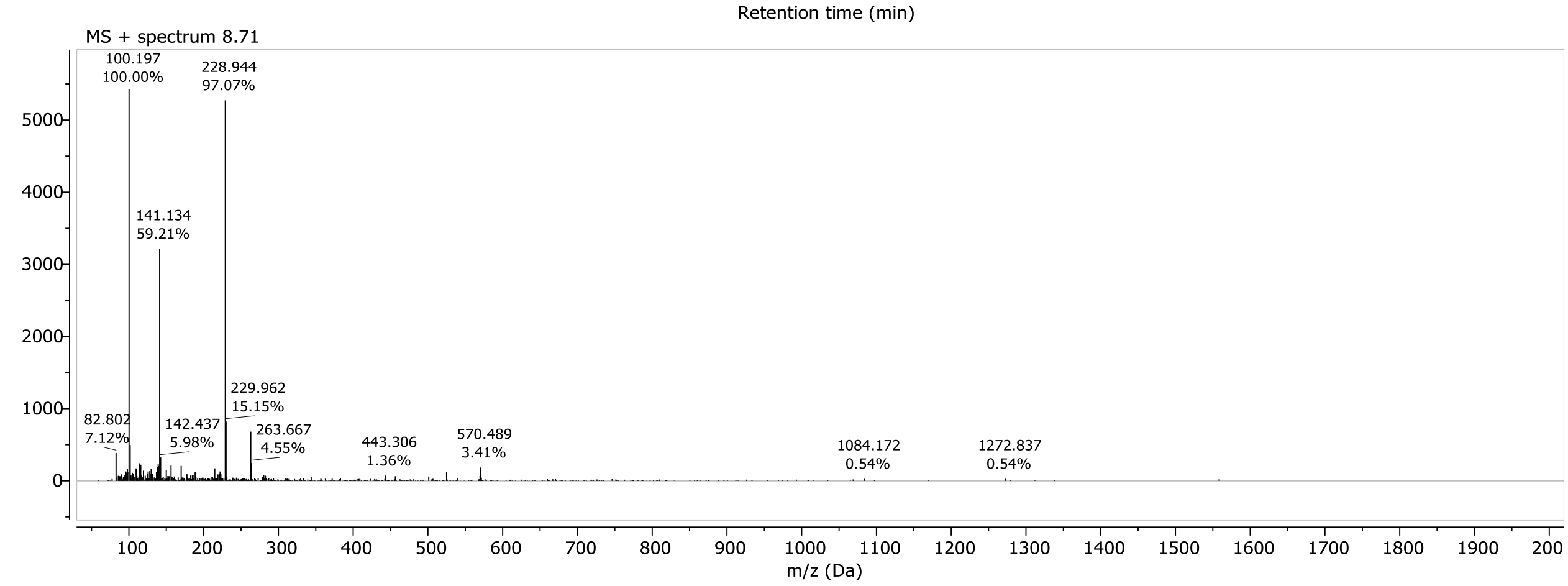

Synthesis of 4-((6-bromo-2--methylquinazolinyl)methylene)hydrazine-1-carboximidamide hydrochloride 2b

Synthesis of 6-bromo-2,4-dimethylquinazoline 9b

The reaction was performed according to **general procedure D**, using NH_4OAc (215 mg, 2.79 mmol, 3.0 eq), 1-(2-amino-5-bromophenyl)ethan-1-one **8b** (200 mg, 0.93 mmol, 1.0 eq) and acetylacetone (112 mg, 1.12 mmol, 1.2 eq). Pure 6-bromo-2,4-dimethylquinazoline **9b** was obtained as a brown solid (138 mg, 0.58 mmol, 63% yield).

Reaction time: 7 hours.

TLC monitoring: eluent mixture 1:1 DCM/EtOAc.

Direct phase flash column chromatography: eluent mixture from 6:4 to 1:1 *n*-Hex/EtOAc.

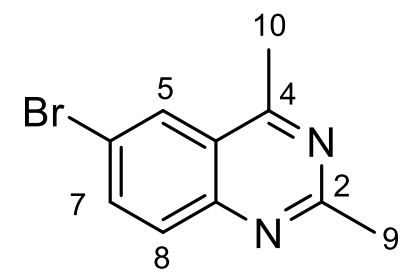

^{1}H-NMR (400 MHz, $CDCl_3$) δ 8.20 (d, *J* = 2.1 Hz, 1H, H5), 7.91 (dd, *J* = 9.0, 2.1 Hz, 1H, H7), 7.83 (d, *J* = 9.0 Hz, 1H, H8), 2.90 (s, 3H, H9), 2.84 (s, 3H, H10).

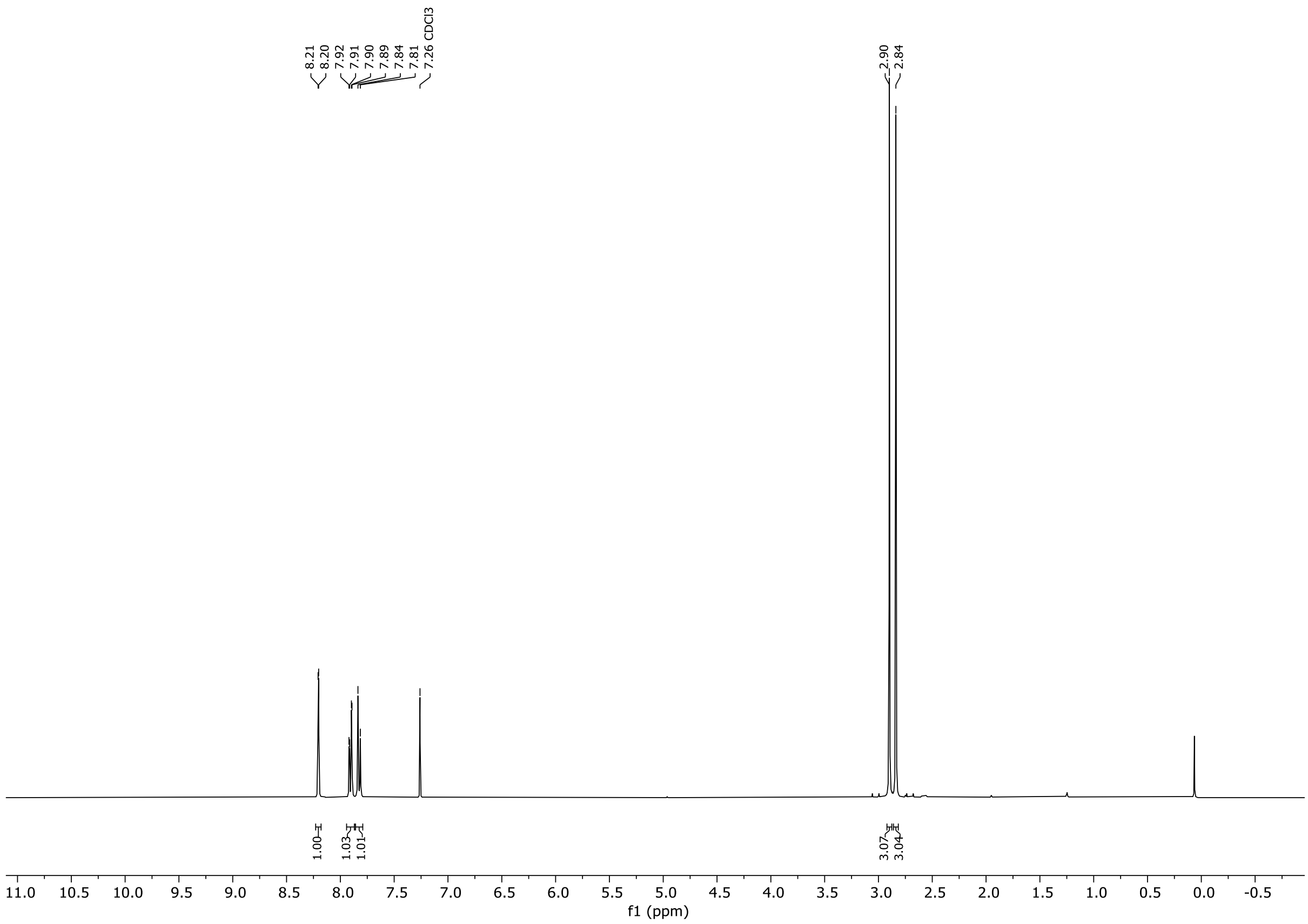

^{13}C-NMR (101 MHz, $CDCl_3$) δ 167.6, 164.0, 148.5, 137.4, 130.1, 127.5, 123.4, 120.4, 26.4, 21.8.

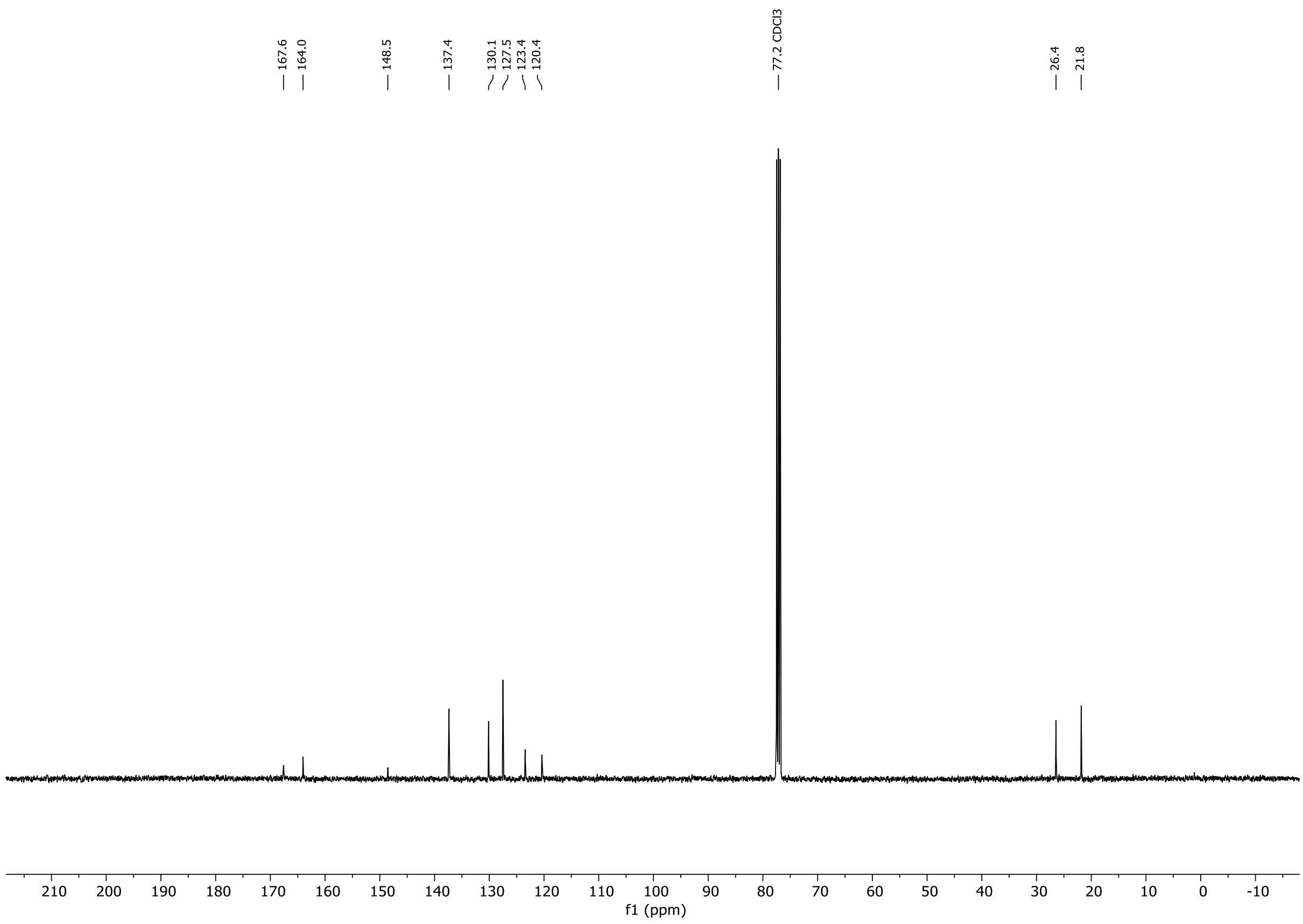

Synthesis of 6-bromo-methylquinazoline 4-carbaldehyde 10b

The reaction was performed according to **general procedure E**, using 6-bromo-2,4-dimethylquinazoline **9b** (128 mg, 0.54 mmol, 1.0 eq), SeO_2 (60 mg, 0.54 mmol, 1.0 eq.) and 1,4-dioxane (2.7 mL). Washing after Celite pad filtration was done using EtOAc (125 mL). After purification, pure 6-bromo-methylquinazoline 4-carbaldehyde **10b** was obtained as a yellow solid (53 mg, 0.21 mmol, 38% yield).

Reaction time: 30 min.

TLC-monitoring: eluent mixture 1:1 DCM/EtOAc.

Direct phase flash column chromatography: eluent mixture 85:15 *n*-Hex/EtOAc.

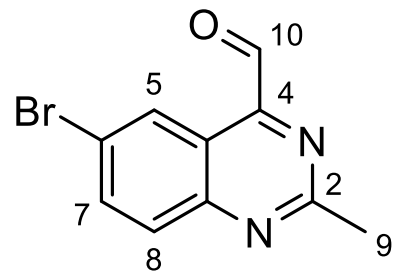

^{1}H-NMR (400 MHz, $CDCl_3$) δ 10.24 (s, 1H, H10), 9.23 (d, *J* = 2.2 Hz, 1H, H5), 7.99 (dd, *J* = 9.0, 2.2 Hz, 1H, H7), 7.89 (d, *J* = 9.0 Hz, 1H, H8), 2.98 (s, 3H, H9).

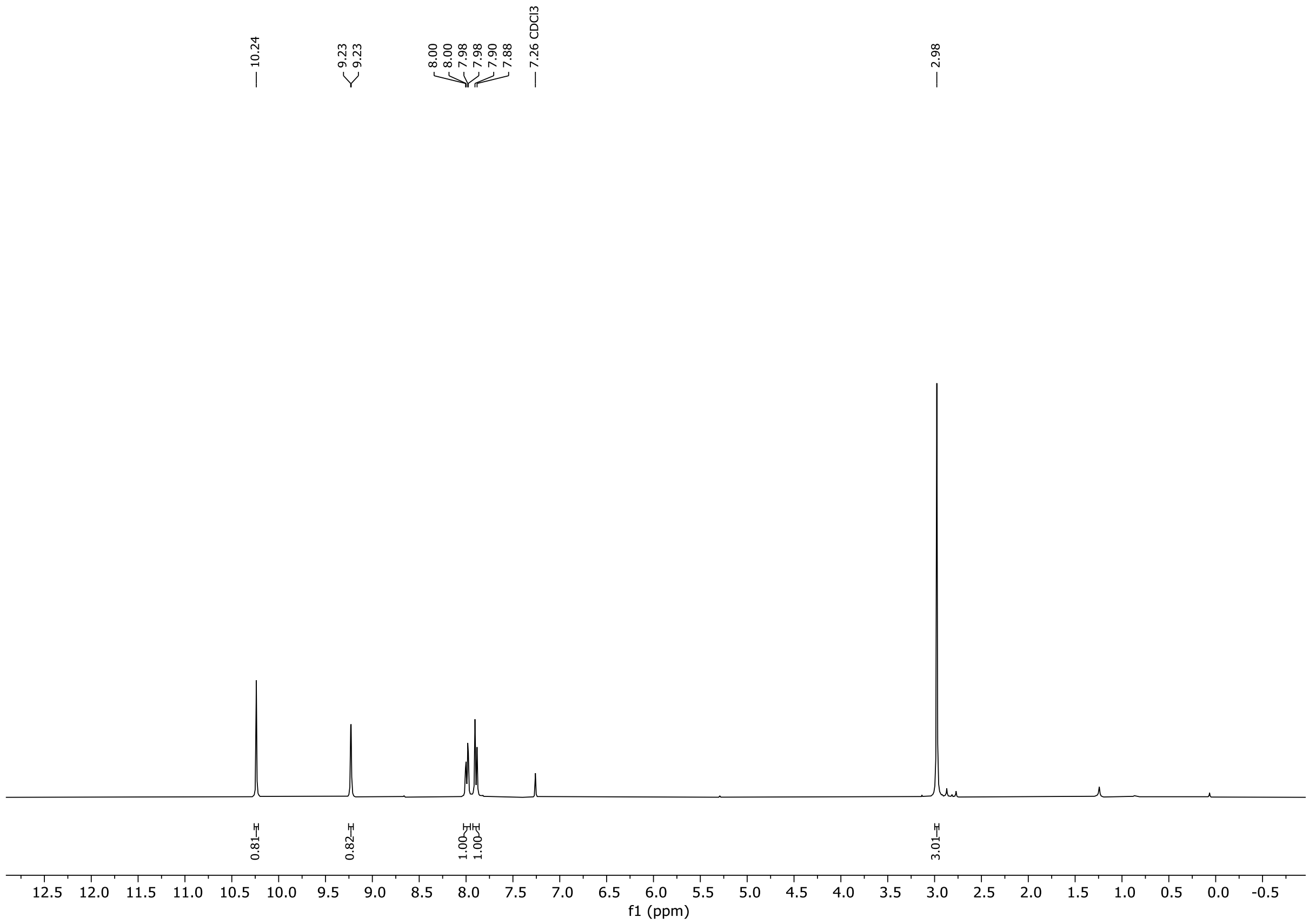

^{13}C-NMR (101 MHz, $CDCl_3$) δ 195.0, 164.9, 154.4, 151.5, 138.3, 129.9, 127.8, 123.8, 119.8, 26.2.

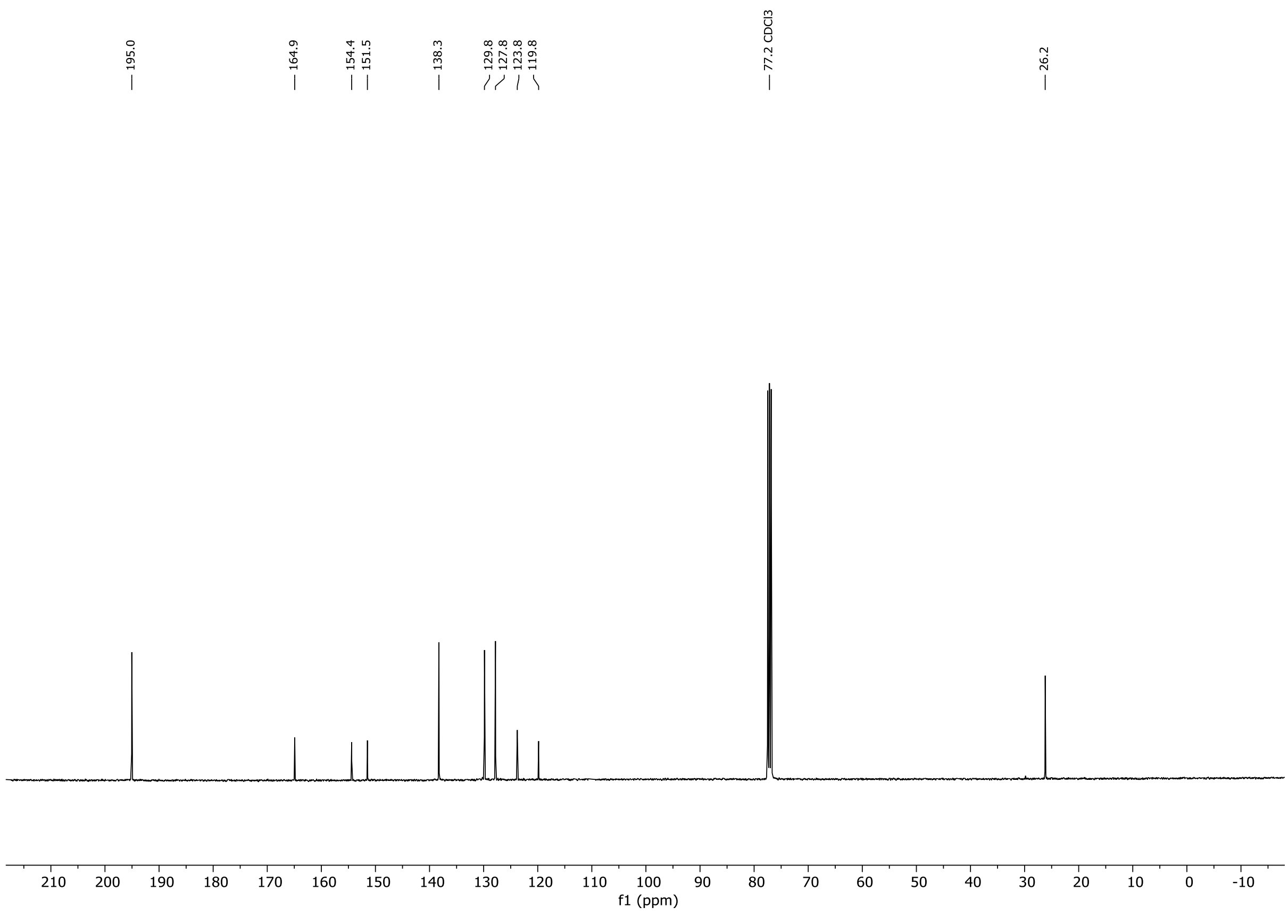

Synthesis of 4-((6-bromo-2-methylquinazolinyl)methylene)hydrazine-1-carboximidamide hydrochloride 2b

The reaction was performed according to **general procedure C**, using the 6-bromo-methylquinazoline 4-carbaldehyde **10b** (48 mg, 0.19 mmol, 1 eq.), AG·HCl (19 mg, 0.17 mmol, 0.90 eq.) and EtOH (2.0 mL). Trituration was done in (*i*-Pr)$_2$O (3 x 2.5 mL). After purification, pure 4-((6-bromo-2-methylquinazolinyl)methylene)hydrazine-1-carboximidamide hydrochloride **2b** was recovered as a yellow solid (24 mg, 0.07 mmol, 34% yield).

1:0.14 E/Z ratio (88%/12%).

Reaction time: 7 hours, then rt overnight.

TLC monitoring: eluent mixture 1:1 DCM/EtOAc.

BIOTAGE® Reverse phase flash column chromatography: eluent mixture from 0% to 100% CH_3CN in H_2O.

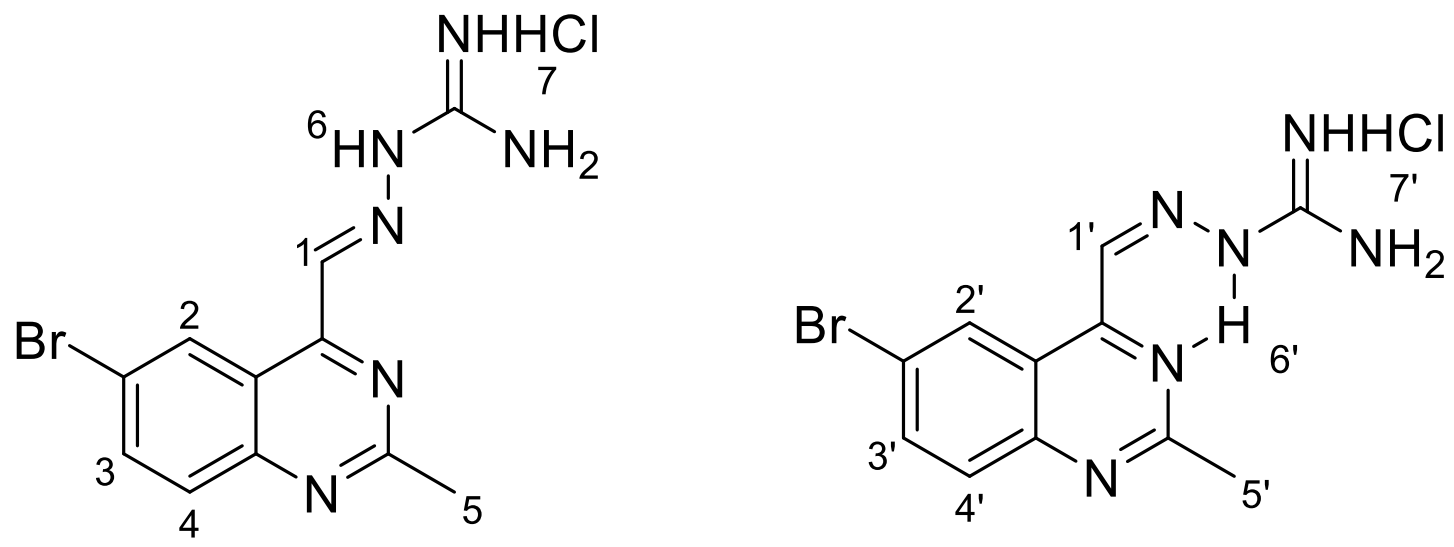

^{1}H NMR (400 MHz, DMSO-d_6) δ (ppm) 14.12 (s, 1H, H6’), 12.62 (s, 1H, H6), 9.04 (d, *J* = 2.1 Hz, 1H, H2’), 9.01 (d, *J* = 2.2 Hz, 1H, H2), 8.67 (s, 1H, H1’), 8.59-8.52 (brs, 4H, H7’), 8.55 (s, 1H, H1), 8.14 (dd, *J* = 9.0, 2.2 Hz, 1H, H3), 8.20 (dd, *J* = 9.0, 2.1 Hz, 1H, H3’), 8.08 (s, 4H, H7), 7.92 (d, *J* = 9.0 Hz, 1H, H4), 7.35 (d, *J* = 9.0 Hz, 1H, H4’), 3.00 (s, 3H, H5’), 2.79 (s, 3H, H5)

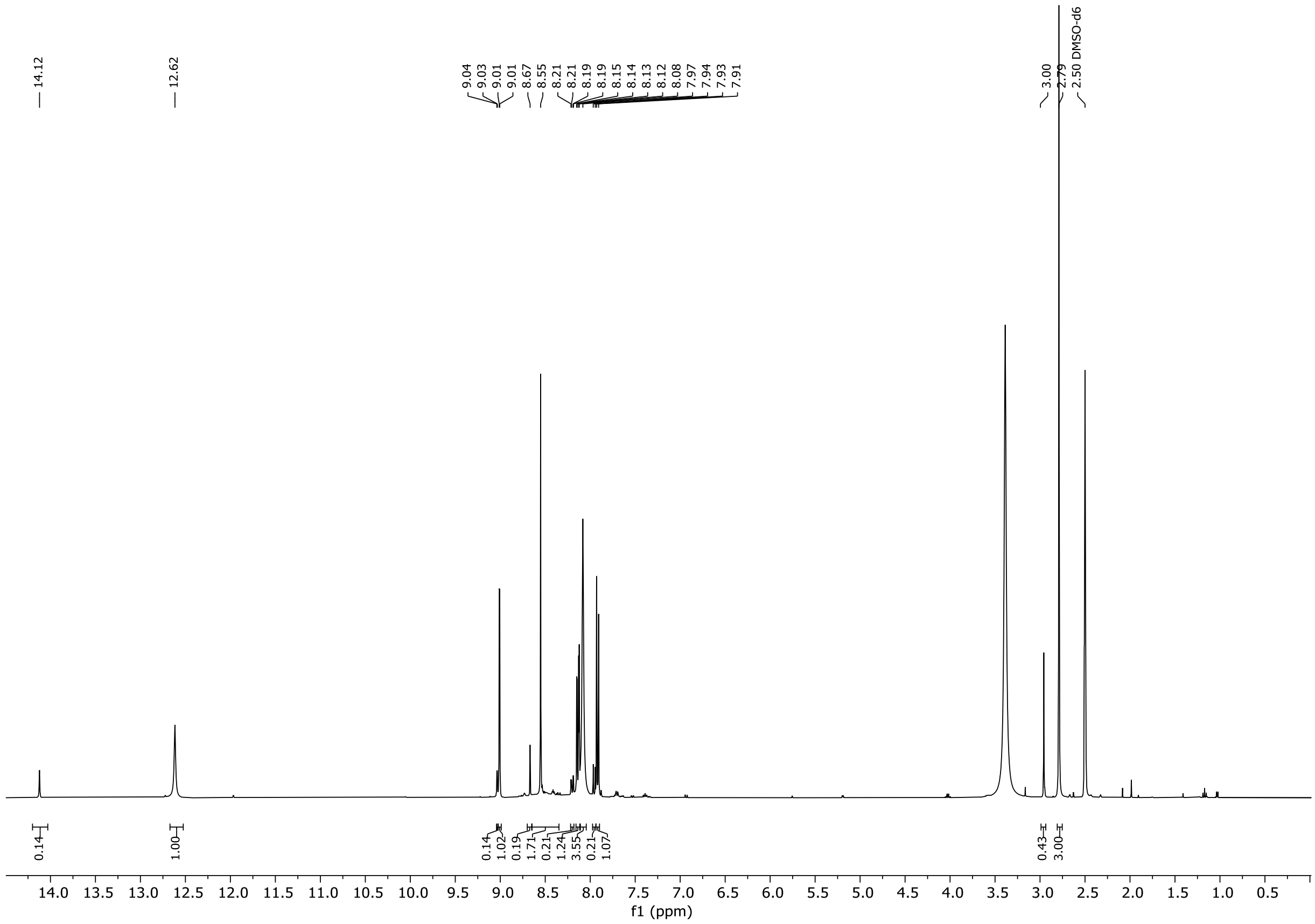

^{13}C NMR (101 MHz, DMSO-d_6) δ 163.6, 157.5, 155.5, 149.9, 146.5, 137.5, 130.0, 128.9, 121.1, 120.5, 25.8

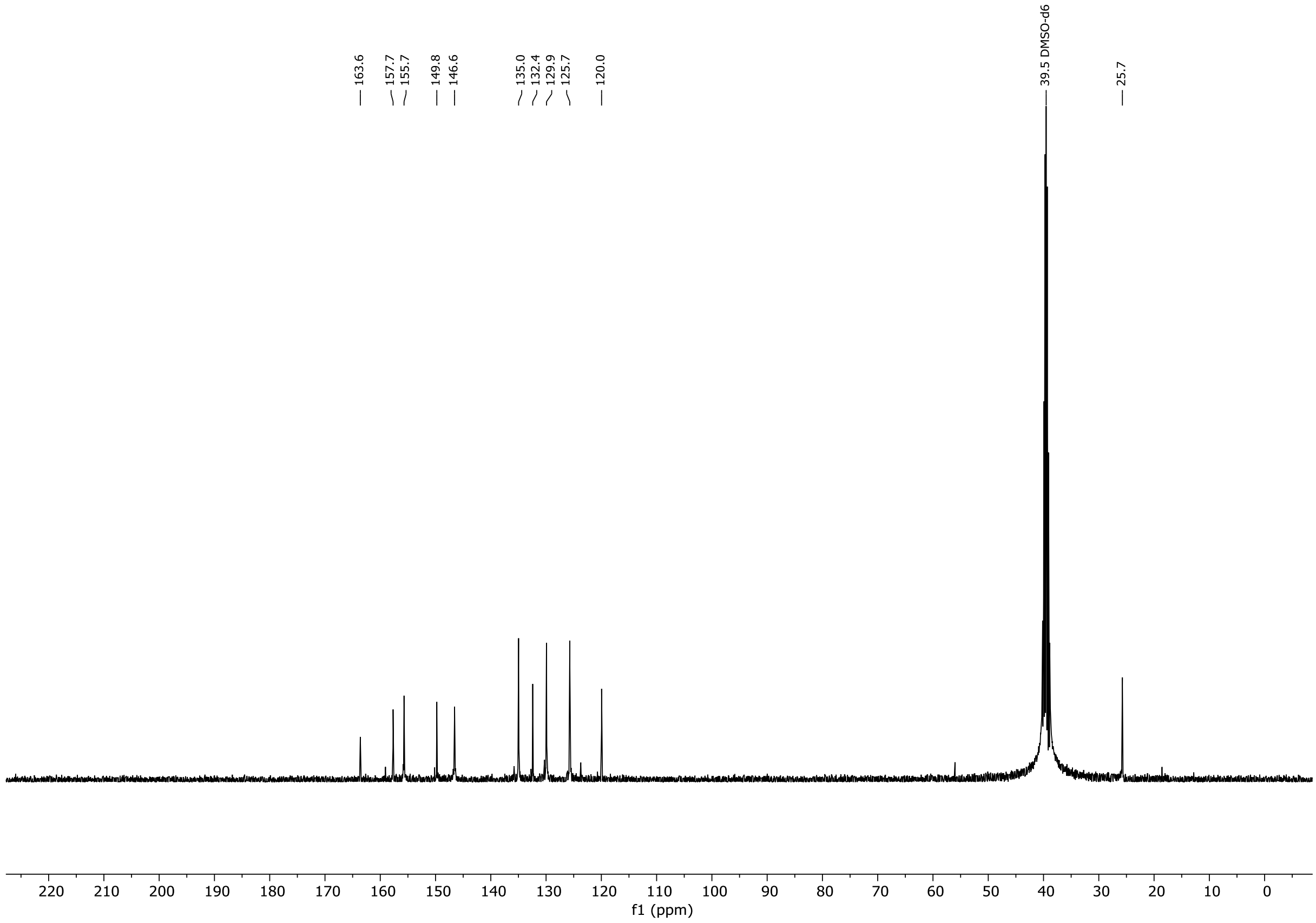

MS (ESI$^+$): m/z [M + H$^+$] 308.80. Calculated MS for $C_{11}H_{12}BrN_6$: 308.16.

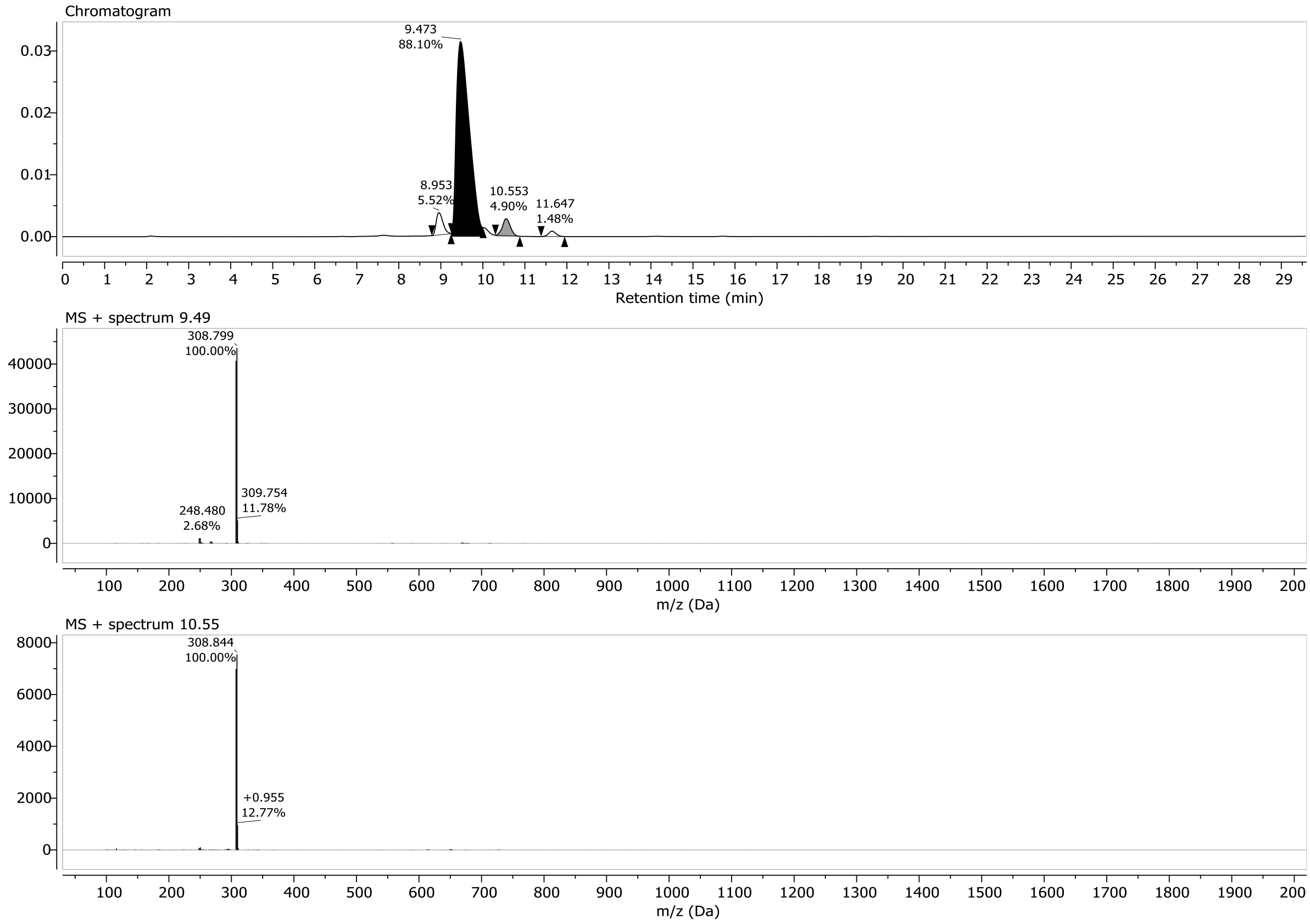

Synthesis of 2-((6-chloromethylquinazolinyl)methylene)hydrazine-1-carboximidamide hydrochloride 2c

Synthesis of 6-chloro-2,4-dimethylquinazoline 9c

The reaction was performed according to **general procedure D**, using NH_4OAc (351 mg, 4.56 mmol, 3.0 eq), 1-(2-amino-5-chlorophenyl)-ethan-1-one **8c** (300 mg, 1.52 mmol, 1.0 eq) and acetylacetone (183 mg, 1.82 mmol, 1.2 eq). Pure 6-chloro-2,4-dimethylquinazoline **9c** was obtained as a yellow solid (210 mg, 1.09 mmol, 72% yield).

Reaction time: 10 hours.

TLC monitoring: eluent mixture 2:8 DCM/EtOAc.

Direct phase flash column chromatography: eluent mixture from 6:4 to 1:1 *n*-Hex/EtOAc.

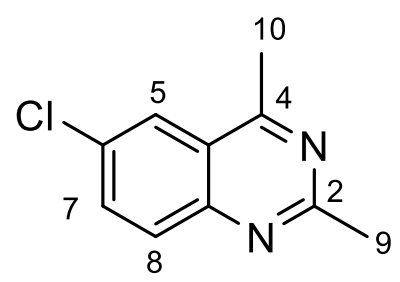

^{1}H-NMR (400 MHz, $CDCl_3$) δ 8.03 (d, *J* = 2.3 Hz, 1H, H5), 7.89 (d, *J* = 9.0 Hz, 1H, H8), 7.78 (dd, *J* = 9.0, 2.3 Hz, 1H, H7), 2.90 (s, 3H, H10), 2.84 (s, 3H, H9).

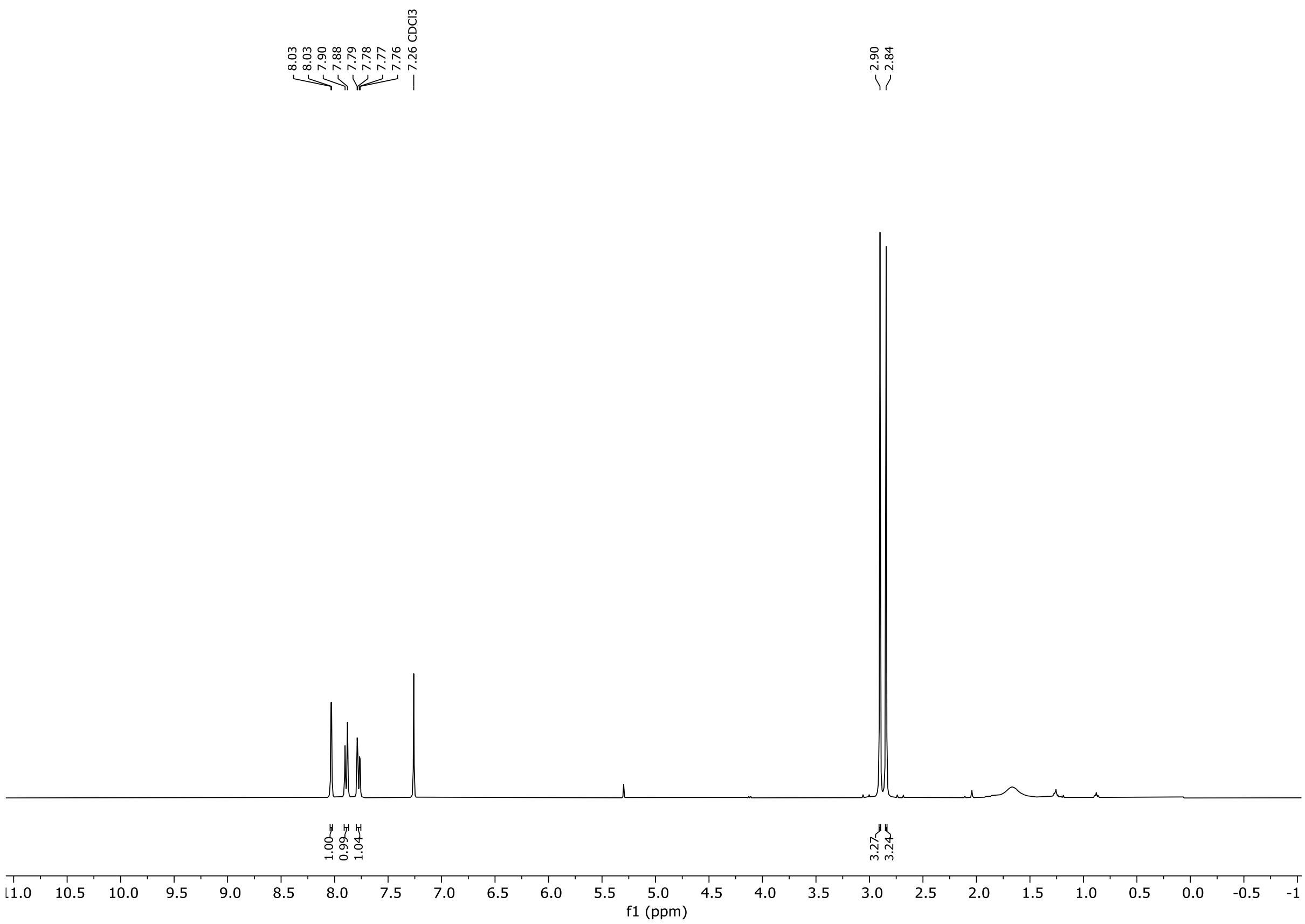

^{13}C-NMR (101 MHz, $CDCl_3$) δ 167.8, 164.4, 148.9, 134.9, 132.5, 130.5, 124.4, 123.2, 26.8, 22.1.

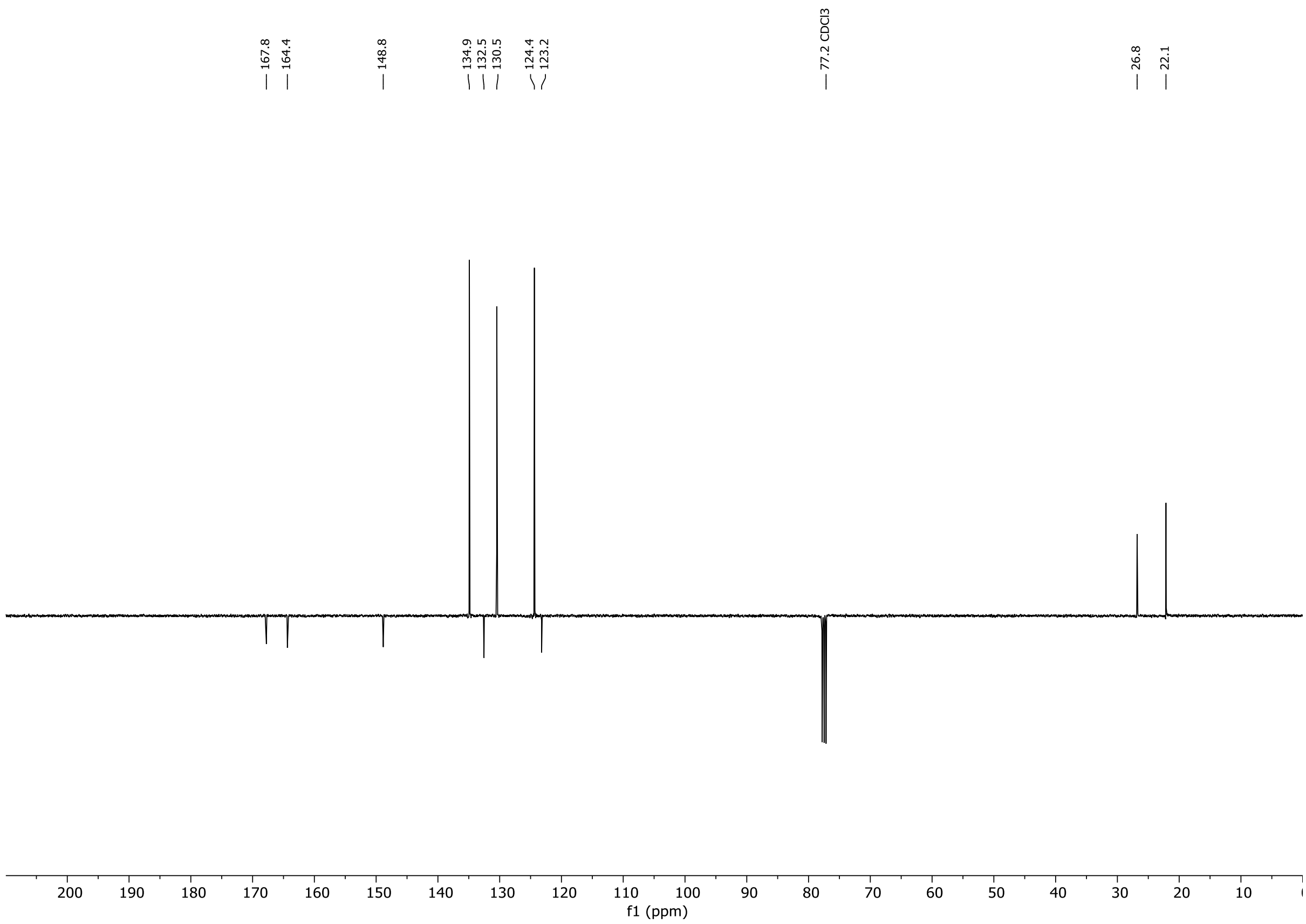

Synthesis of 6-chloro-methylquinazoline 4-carbaldehyde 10c

The reaction was performed according to **general procedure E**, using 6-chloro-2,4-dimethylquinazoline **9c** (95 mg, 0.49 mmol, 1.0 eq), SeO_2 (55 mg, 0.49 mmol, 1.0 eq.) and 1,4-dioxane (2.45 mL). Washing after Celite pad filtration was done using EtOAc (125 mL). After purification, pure 6-chloro-methylquinazoline 4-carbaldehyde **10c** was obtained as a yellow solid (33 mg, 0.16 mmol, 33% yield).

Reaction time: 30 min.

TLC monitoring: eluent mixture 1:1 DCM/EtOAc.

Direct phase flash column chromatography: eluent mixture from 85:15 to 6:4 *n*-Hex/EtOAc.

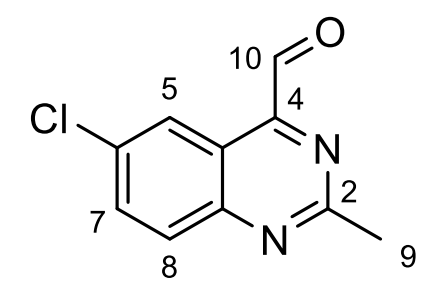

^{1}H-NMR (400 MHz, $CDCl_3$) δ 10.26 (s, 1H, H10), 9.08 (d, *J* = 2.3 Hz, 1H, H5), 7.99 (d, *J* = 9.0 Hz, 1H, H8), 7.87 (dd, *J* = 9.1, 2.3 Hz, 1H, H7), 3.00 (s, 3H, H9).

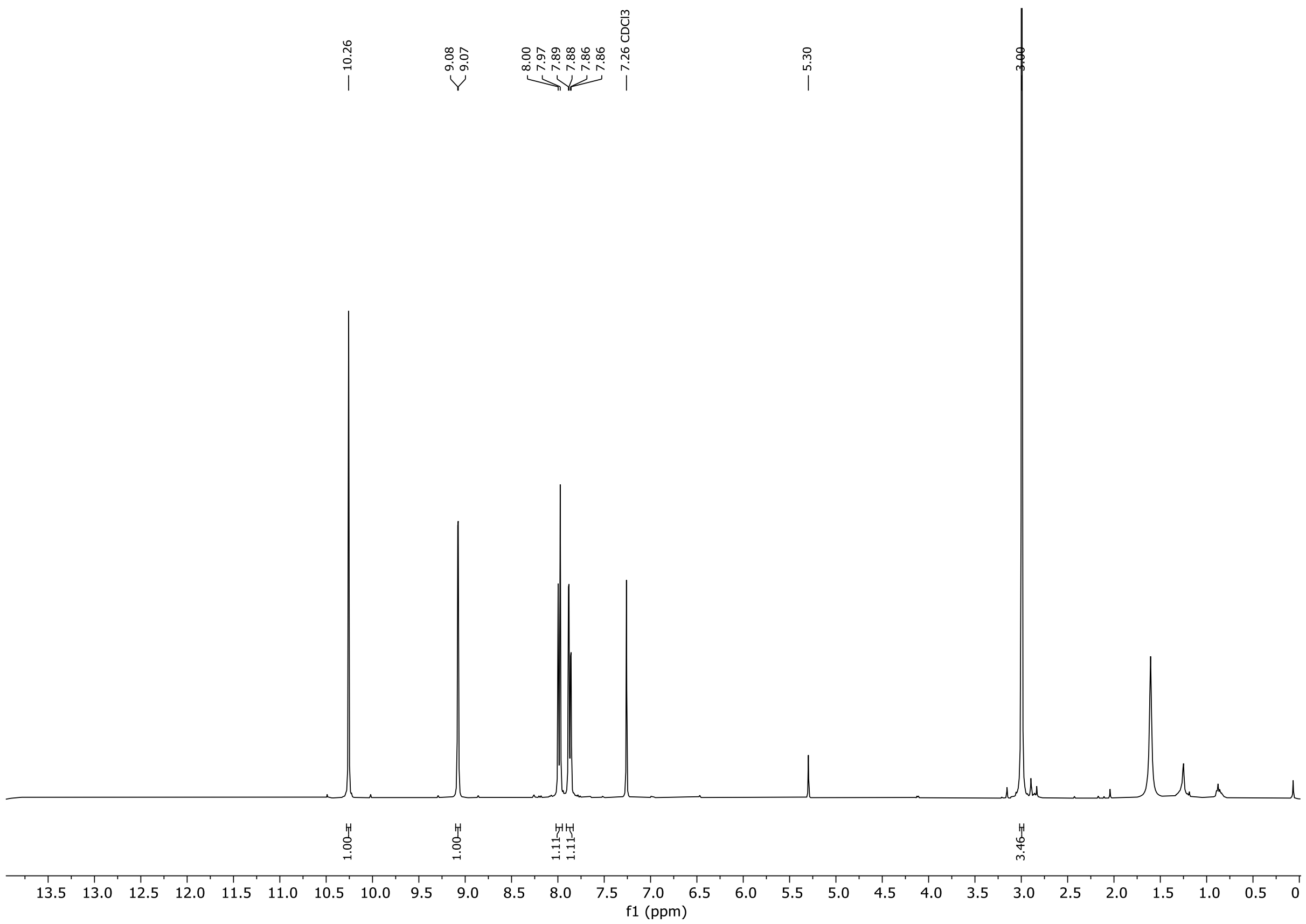

^{13}C-NMR (101 MHz, $CDCl_3$) δ 195.1, 164.8, 154., 151.4, 135.8, 135.6, 129.8, 124.5, 119.4, 26.2.

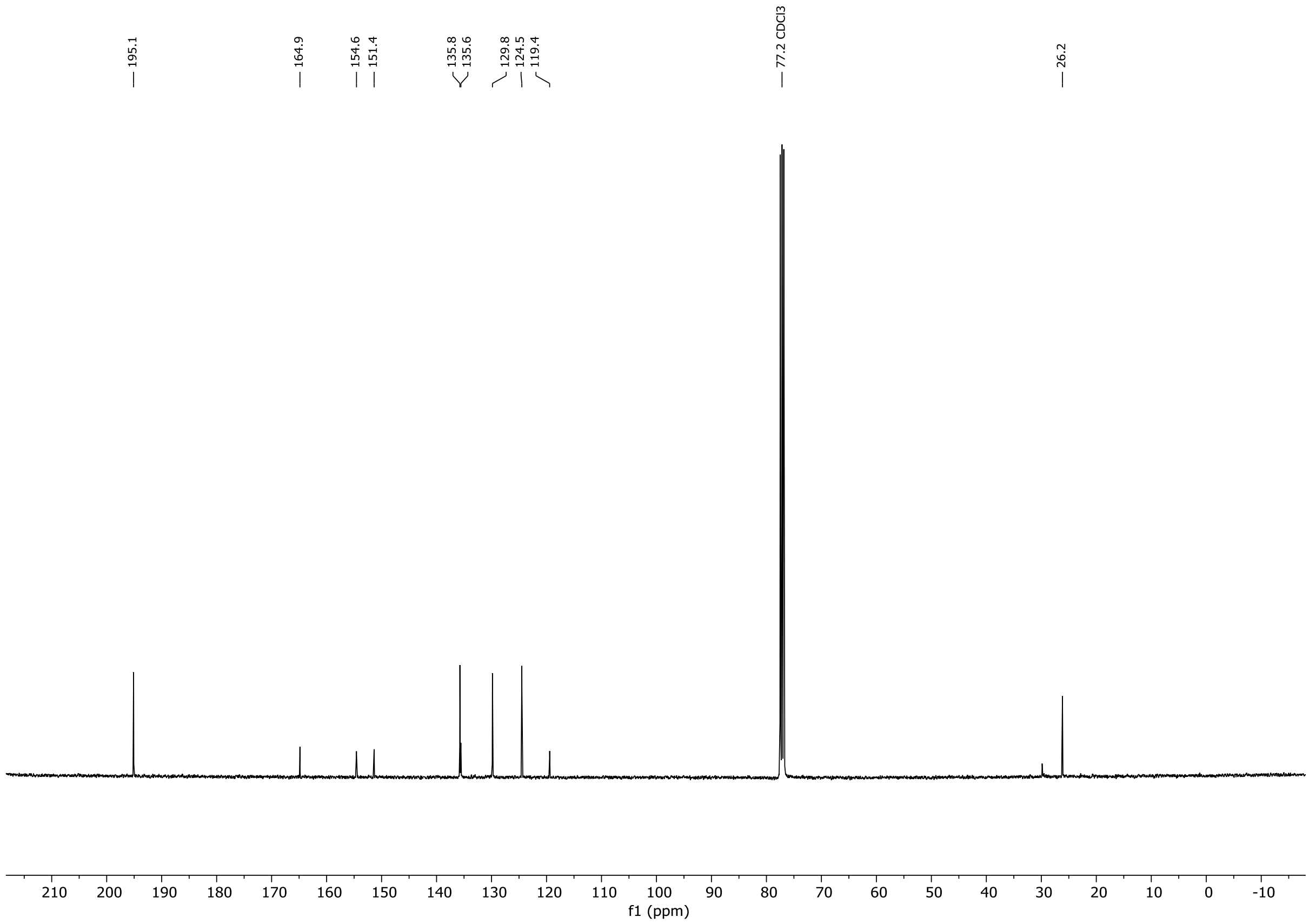

Synthesis of 4-((6-chloro-2-methylquinazolinyl)methylene)hydrazine-1-carboximidamide hydrochloride 2c

The reaction was performed according to **general procedure C**, using 6-chloro-methylquinazoline 4-carbaldehyde **10c** (31 mg, 0.15 mmol, 1 eq.), AG·HCl (15 mg, 0.14 mmol, 0.90 eq.) and EtOH (1.5 mL). Trituration was done in (i-Pr)$_2$O (3x 2.5 mL). After purification, pure 4-((6-chloro-2-methylquinazolinyl)methylene)hydrazine-1-carboximidamide hydrochloride **2c** was recovered as a yellow solid (12 mg, 0.09 mmol, 27% yield).

1:0.05 E/Z ratio (95%/5%).

Reaction time: 7 hours, then rt overnight.

TLC monitoring: eluent mixture 1:1 DCM/EtOAc.

BIOTAGE® Reverse phase flash column chromatography: eluent mixture from 0% to 100% CH_3CN in H_2O.

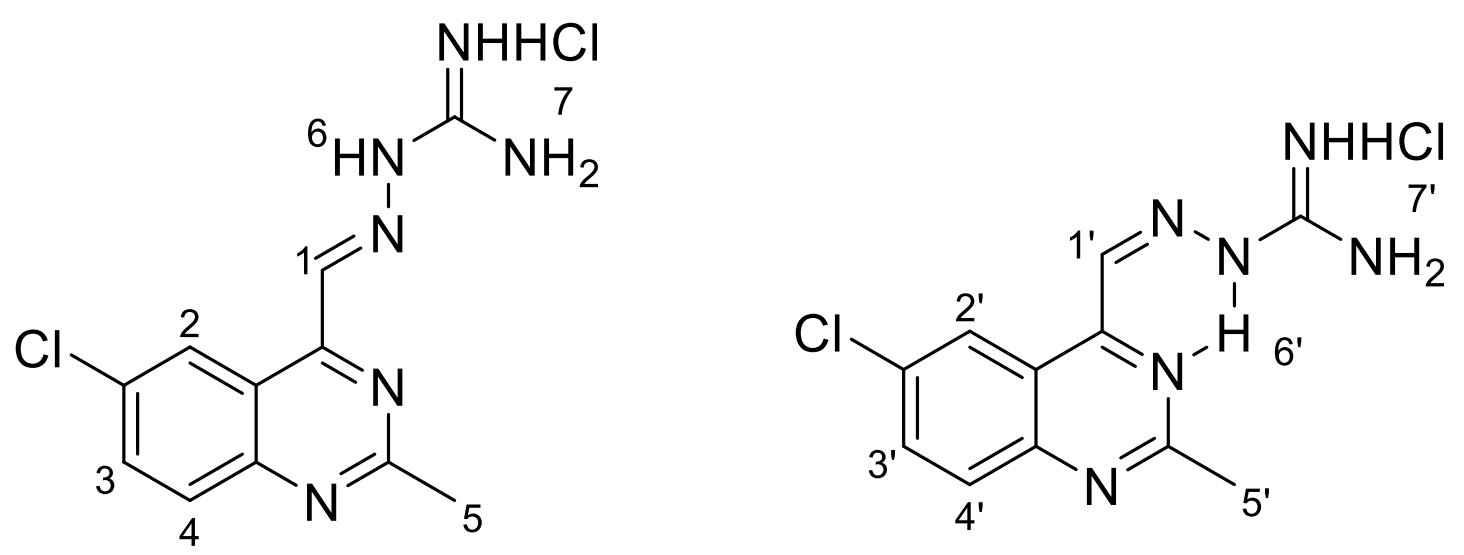

^{1}H NMR (400 MHz, DMSO-d_6) δ (ppm) 12.60 (s, 1H, H6), 8.92 (s, 1H, H2'), 8.88 (s, 1H, H2), 8.66 (s, 1H, H1'), 8.54 (s, 1H, H1), 8.57-8.50 (brs, 4H, H7'), 8.07 (brs, 4H, H7), 8.04 (dd, J = 9.0, 2.2 Hz, 2H, H3, H3'), 8.00 (d, J = 9.0 Hz, 2H, H4, H4'), 2.97 (s, 3H, H5'), 2.80 (s, 3H, H5).

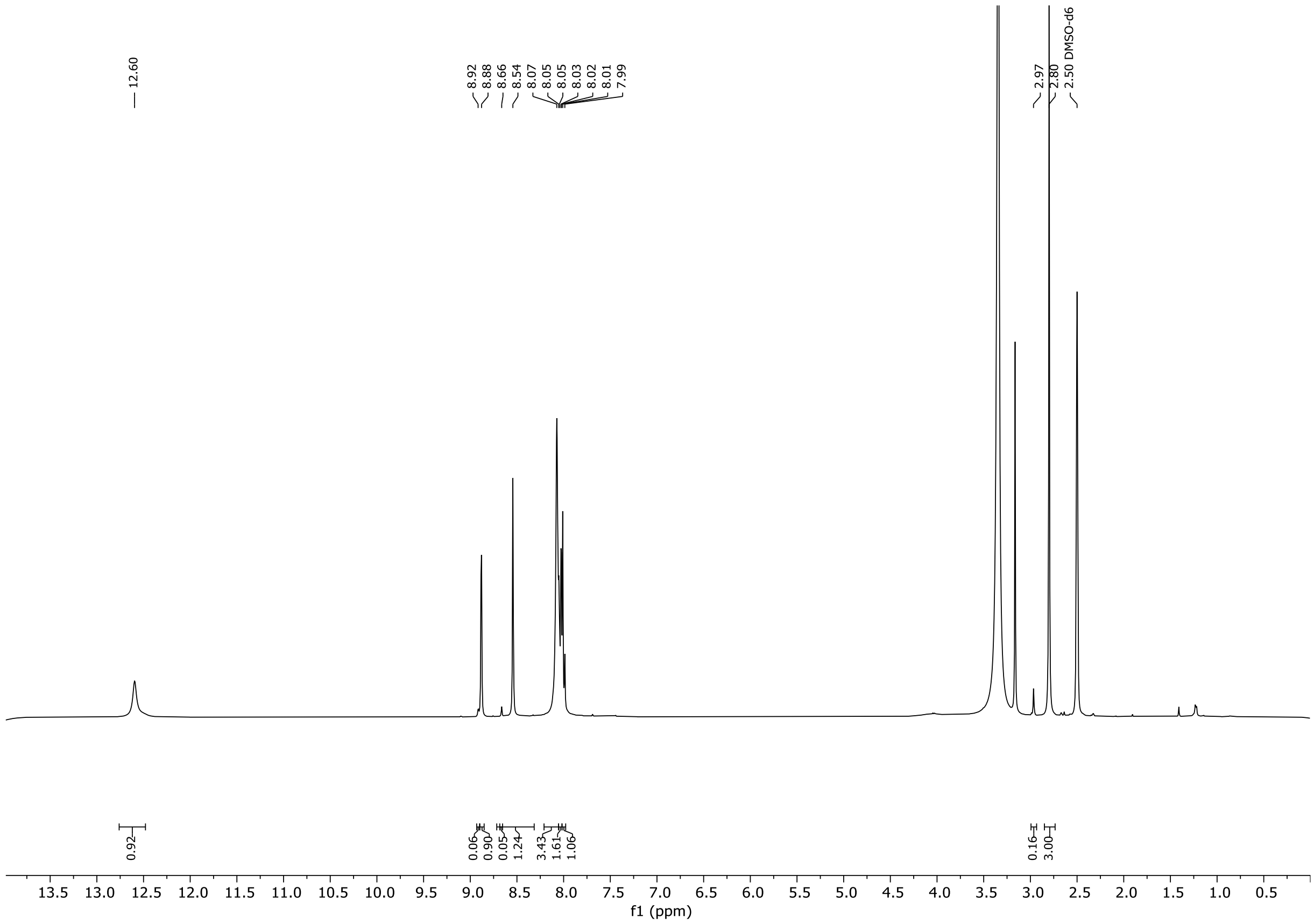

^{13}C NMR (101 MHz, DMSO-d_6) δ 163.6, 157.7, 155.7, 149.8, 146.6, 135.0, 132.4, 129.9, 125.7, 120.0, 25.7.

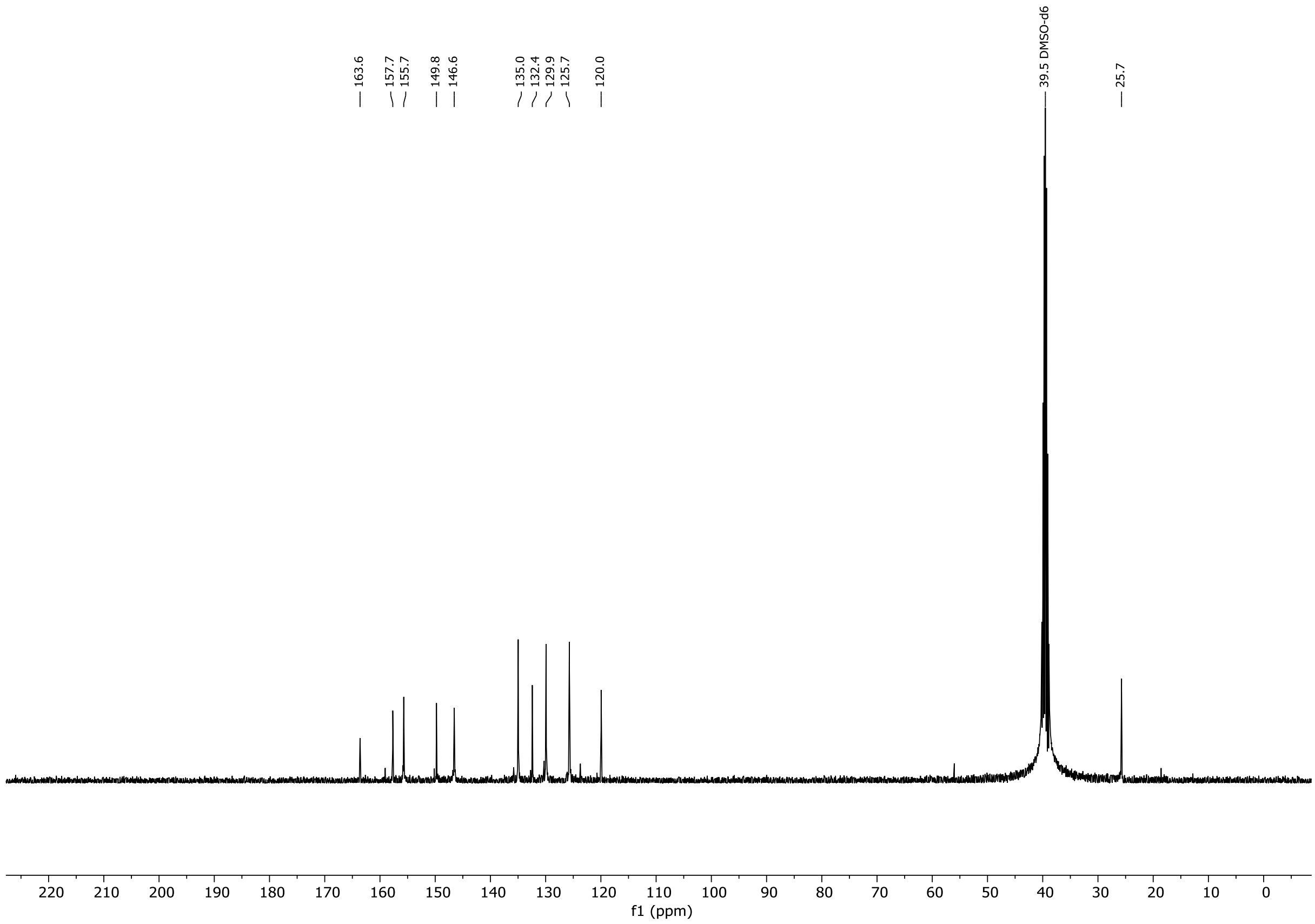

MS (ESI$^+$): m/z [M + H$^+$] 263.12. Calculated MS for $C_{11}H_{12}ClN_6$: 263.08.

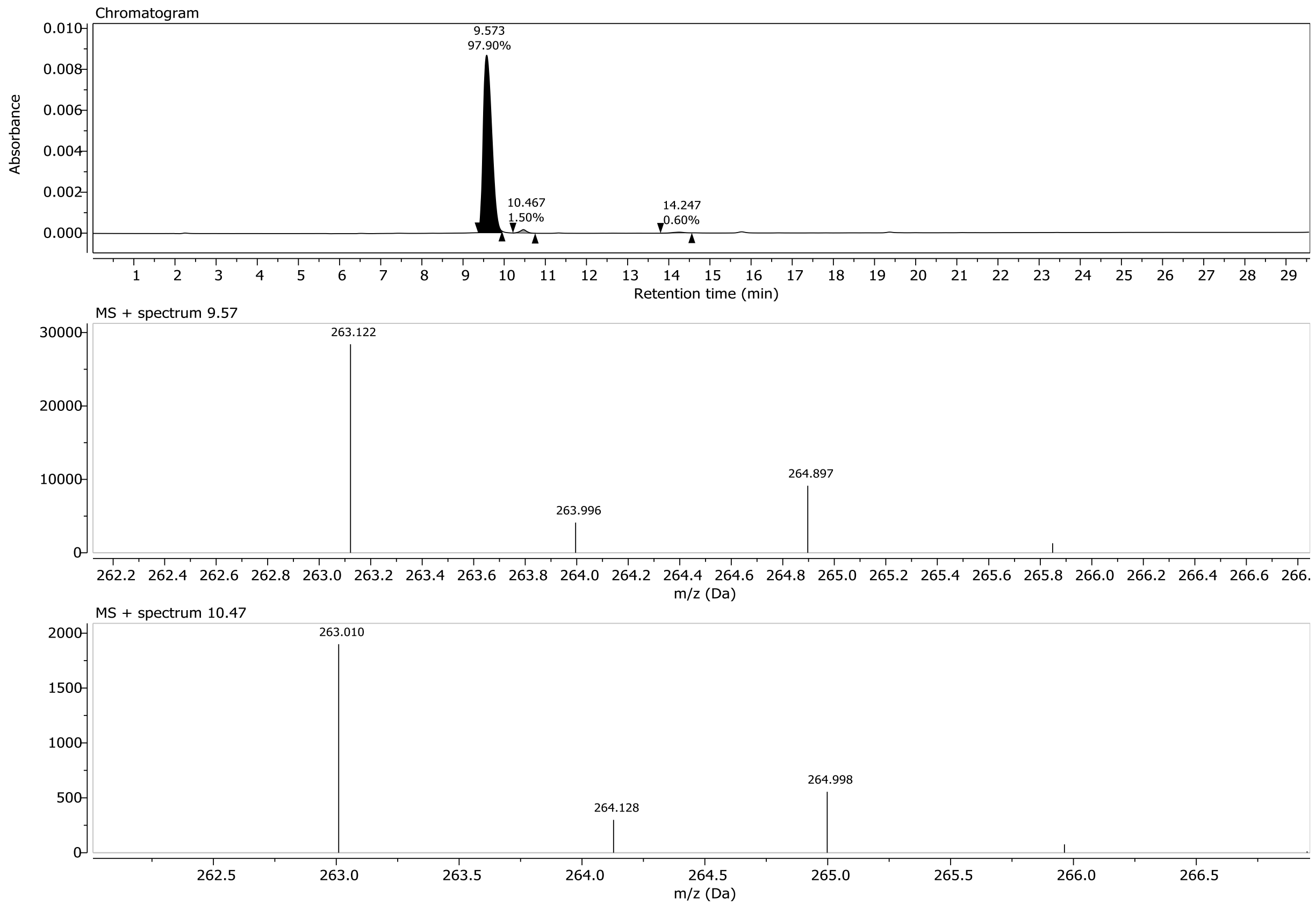

Synthesis of 4-((2-methyl-6-phenylquinazolinyl)methylene)hydrazine-1-carboximidamide hydrochloride 2d

Synthesis of 2,4-dimethyl-6-phenylquinazoline 9d

6-Bromo-2,4-dimethylquinazoline **9b** (200 mg, 0.84 mmol, 1.00 eq) was dissolved in toluene (1.68 mL) under nitrogen atmosphere. A solution of Na_2CO_3 (356 mg, 3.36 mmol, 4.00 eq) in H_2O (1.68 mL), a solution of phenylboronic acid (123 mg, 1.01 mmol, 1.20 eq) in EtOH (0.84 mL) and solid $Pd(PPh_3)_4$ (19 mg, 0.02 mmol 0.02 eq) were subsequently added. The resulting mixture was stirred at 110 °C for 6 hours, monitoring by TLC (eluent mixture: 1:1 DCM/EtOAc), then concentrated at reduced pressure and extracted with EtOAc (3 x 15 mL). The combined organic phases were washed with brine (15 mL), dried over Na_2SO_4 and the solvent was removed under reduced pressure. The crude was purified *via* direct phase flash column chromatography (eluent mixture: 7:3 DCM/EtOAc), obtaining pure 2,4-dimethyl-6-phenylquinazoline **9d** as a yellow solid (180 mg, 0.77 mmol, 91% yield).

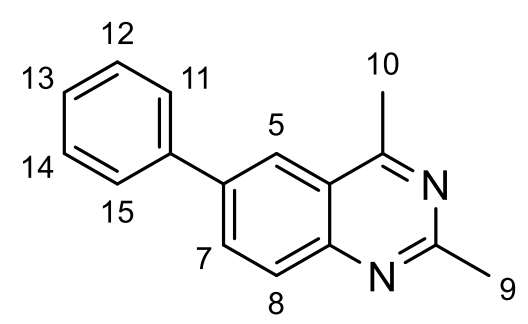

^{1}H-NMR (400 MHz, $CDCl_3$) δ 8.20 (d, *J* = 2.0 Hz, 1H, H5), 8.10 (dd, *J* = 8.7, 2.0 Hz, 1H, H7), 8.01 (d, *J* = 8.7 Hz, 1H, H8), 7.69 (d, *J* = 7.0 Hz, 2H, H11, H15), 7.51 (t, 2H, H12, H14), 7.42 (d, *J* = 7.4 Hz, 1H, H13), 2.98 (s, 3H, H10), 2.87 (s, 3H, H9).

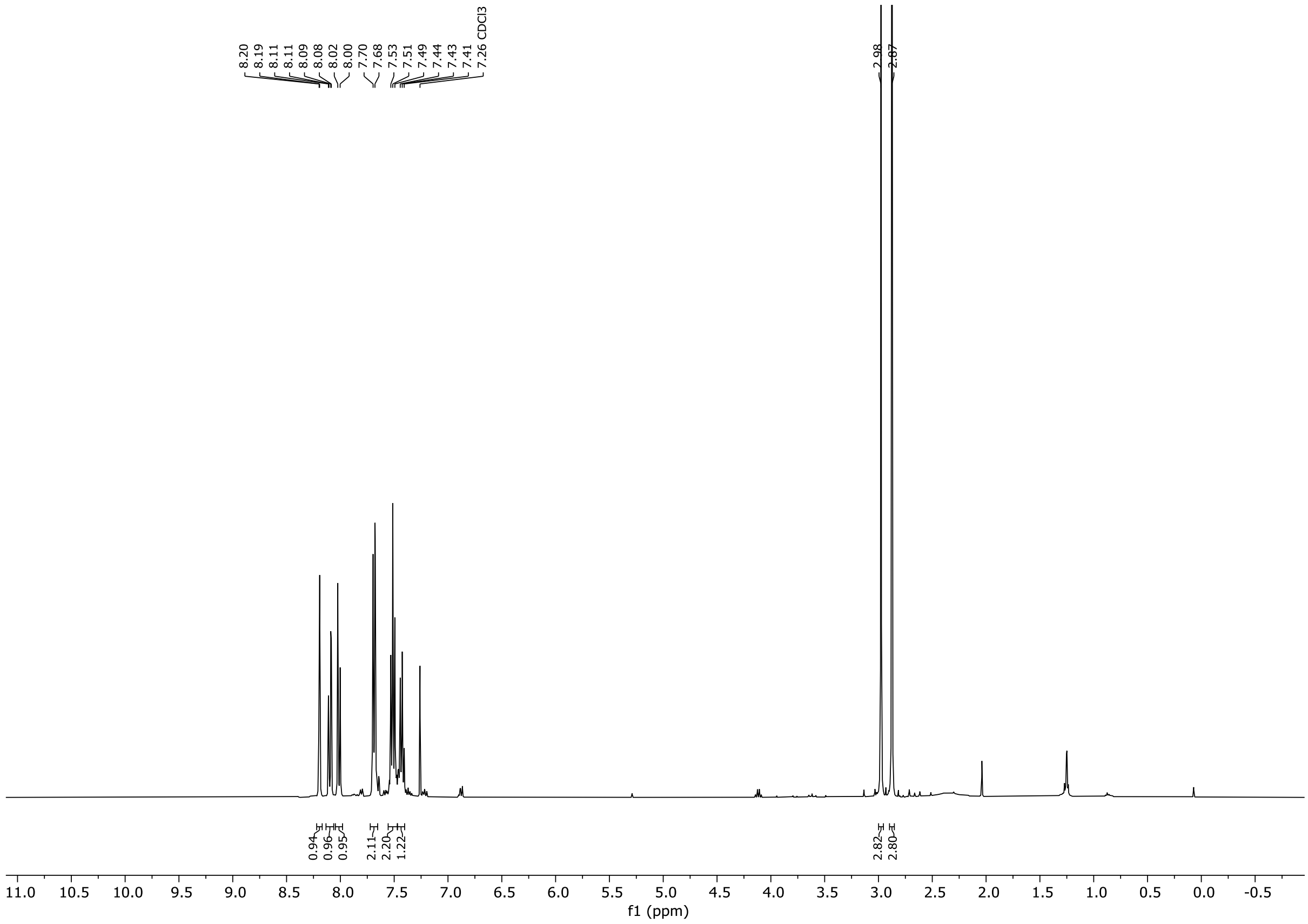

^{13}C-NMR (101 MHz, $CDCl_3$) δ 168.5, 163.5, 149.2, 140.1, 139.9, 133.6, 132.3, 132.2, 129.2, 128.7, 128.2, 127.6, 122.8, 122.5, 26.5, 21.9.

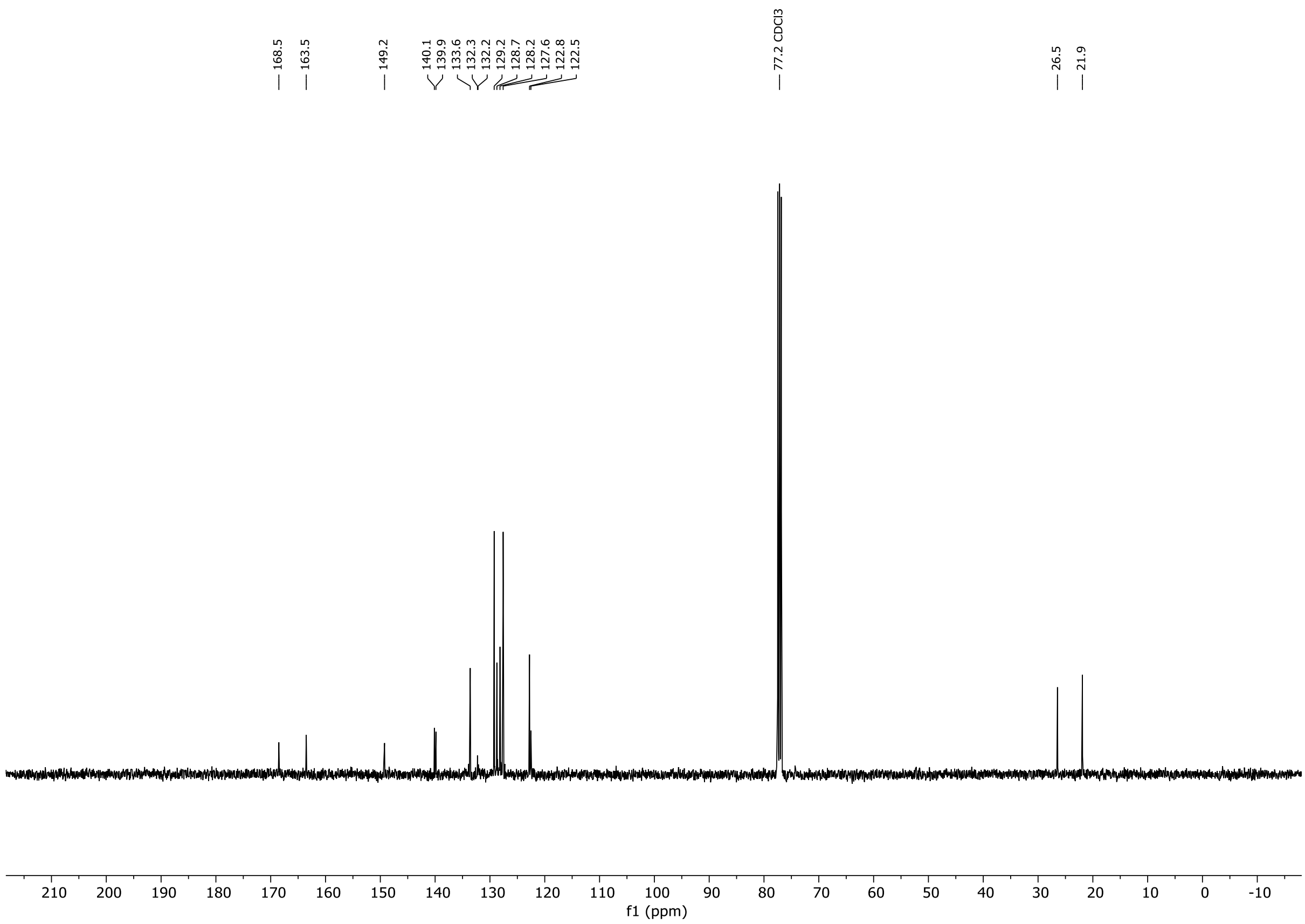

Synthesis of methyl-6-phenylquinazoline 4-carbaldehyde 10d

The reaction was performed according to **general procedure E**, using 2,4-dimethyl-6-phenylquinazoline **9d** (150 mg, 0.64 mmol, 1 eq.), SeO_2 (71 mg, 0.64 mmol, 1 eq), and 1,4-dioxane (3.2 mL). Washing after Celite pad filtration was done using EtOAc (125 mL). After purification, pure methyl-6-phenylquinazoline 4-carbaldehyde **10d** was obtained as a brown solid (70 mg, 0.28 mmol, 43% yield).

Reaction time: 30 min.

TLC monitoring: eluent mixture 1:1 DCM/EtOAc.

Direct phase flash column chromatography: eluent mixture from 7:3 to 1:1 *n*-Hex/EtOAc.

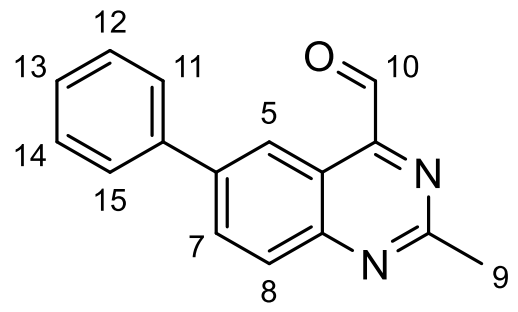

^{1}H-NMR (400 MHz, $CDCl_3$) δ 10.32 (s, 1H, H10), 9.26 (d, *J* = 2.1 Hz, 1H, H5), 8.21 (dd, *J* = 8.8, 2.1 Hz, 1H, H7), 8.10 (d, *J* = 8.8 Hz, 1H, H8), 7.74 (d, *J* = 7.3 Hz, 2H, H11, H15), 7.52 (t, *J* = 7.3 Hz, 2H, H12, H14), 7.44 (t, *J* = 7.3 Hz, 1H, H13), 3.02 (s, 3H, H9).

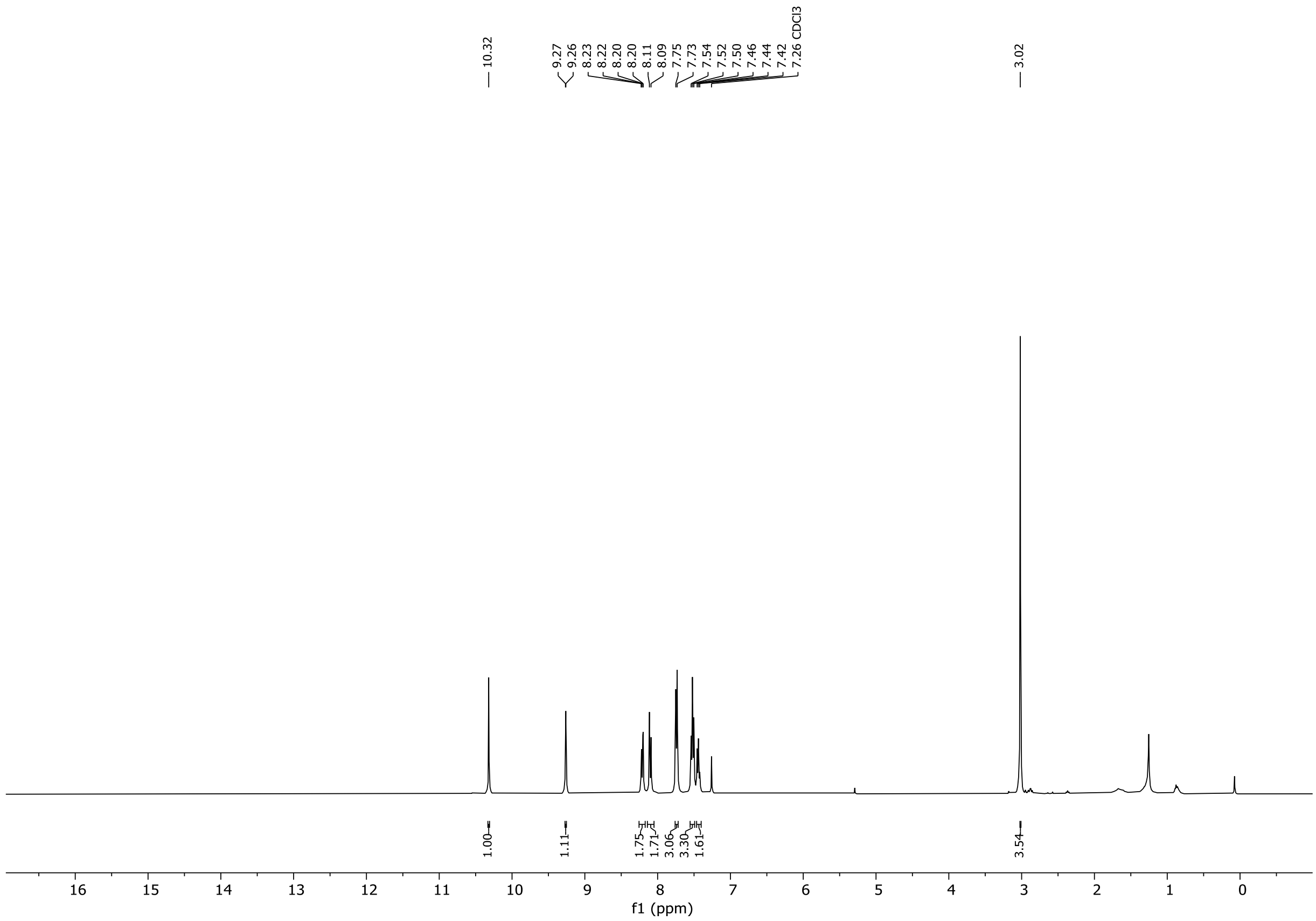

^{13}C-NMR (101 MHz, $CDCl_3$) δ 195.6, 164.3, 155.5, 152.3, 142.2, 139.6, 134.4, 129.3, 128.6, 128.5, 127.7, 122.9, 119.5, 26.2.

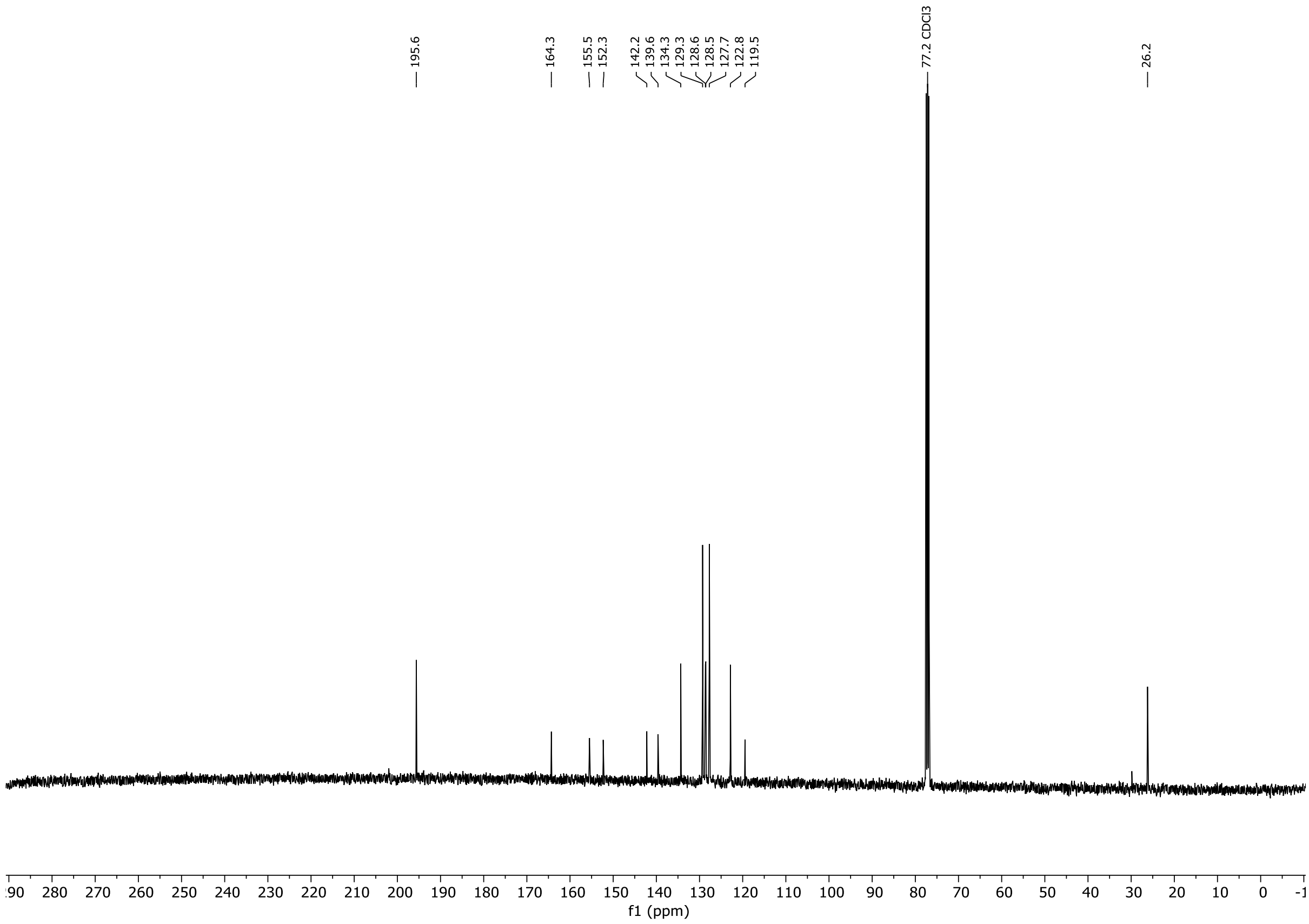

Synthesis of 4-((2-methyl-6-phenylquinazolinyl)methylene)hydrazine-1-carboximidamide hydrochloride 2d

The reaction was performed according to **general procedure C**, using methyl-6-phenylquinazoline 4-carbaldehyde **10d** (55 mg, 0.22 mmol, 1 eq.), AG·HCl (22 mg, 0.20 mmol, 0.90 eq.) and EtOH (2.2 mL). Trituration was done in (i-Pr)$_2$O (3 x 2.5 mL). After purification, pure 4-((2-methyl-6-phenylquinazolinyl)methylene)hydrazine-1-carboximidamide hydrochloride **2d** was recovered as a yellow solid (15 mg, 0.05 mmol, 23% yield).

1:0.09 E/Z ratio (92%/8%).

Reaction time: 7 hours, then rt overnight.

TLC monitoring: eluent mixture 1:1 DCM/EtOAc.

BIOTAGE® Reverse phase flash column chromatography: eluent mixture from 0% to 100% CH_3CN in H_2O.

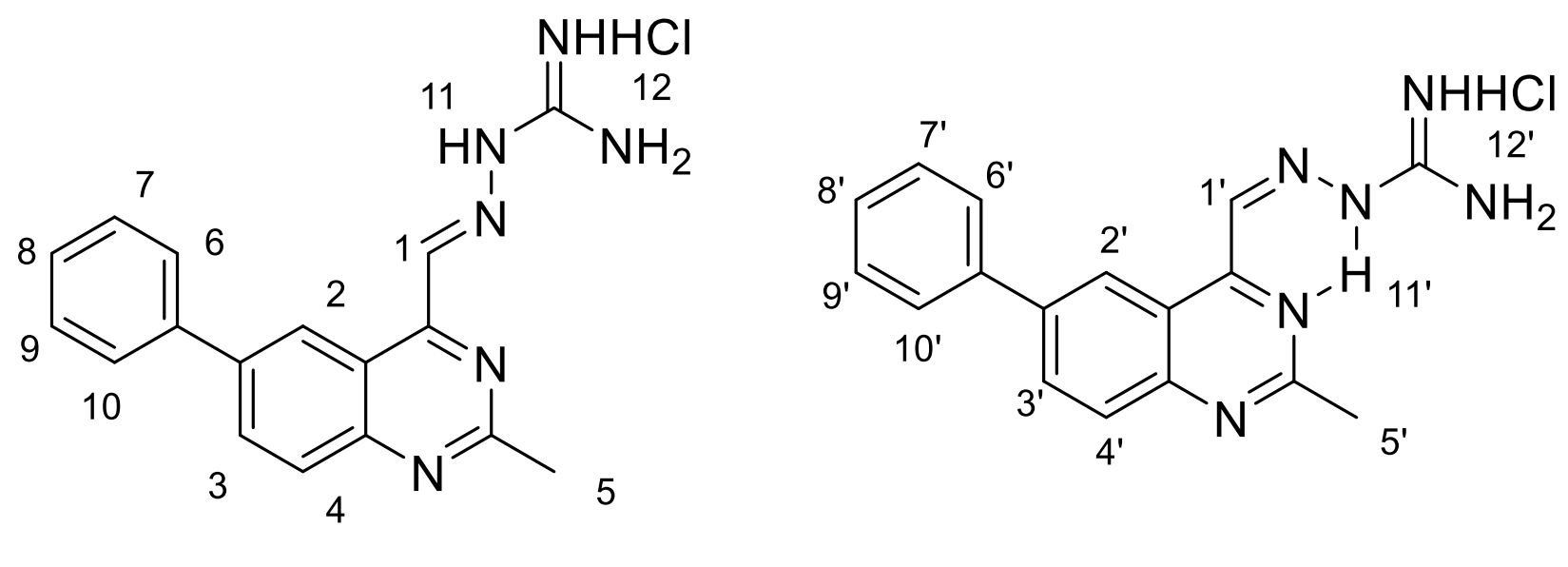

^{1}H NMR (400 MHz, DMSO-d_6) δ (ppm) 14.25 (s, 1H, H11'), 12.66 (s, 1H, H11), 9.04 (d, *J* = 2.1 Hz, 1H, H2), 8.92 (d, *J* = 2.0 Hz, 1H, H2'), 8.87 (s, 1H, H1'), 8.88-8.80 (brs, 4H, H12') 8.66 (s, 1H, H1), 8.42 (dd, *J* = 8.8, 2.0 Hz, 1H, H3'), 8.34 (dd, *J* = 8.8, 2.1 Hz, 1H, H3), 8.08 (brs, 4H, H12), 8.06 (d, *J* = 8.8 Hz, 2H, H4, H4'), 7.96 (d, *J* = 7.2 Hz, 2H, H6', H10'), 7.88 (d, *J* = 7.4 Hz, 2H, H6, H10), 7.54 (t, *J* = 7.5 Hz, 3H, H7, H9), 7.45 (t, *J* = 7.3 Hz, 1H, H8), 2.99 (s, 3H, H5'), 2.82 (s, 3H, H5).

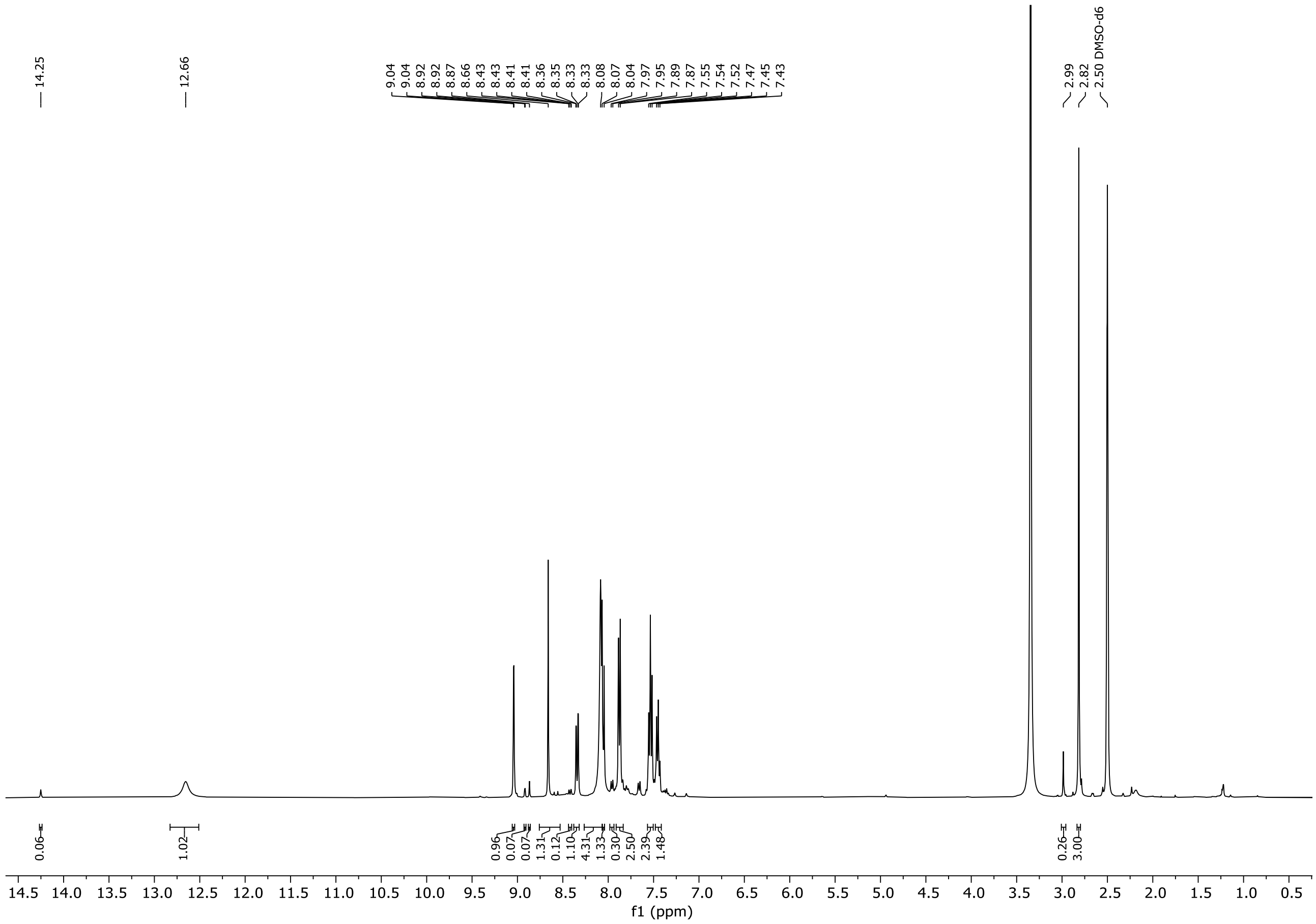

^{13}C NMR (101 MHz, DMSO-d_6) δ 163.1, 158.5, 155.7, 150.6, 146.4, 139.4, 139.1, 133.5, 129.1, 128.4, 128.2, 127.4, 123.9, 119.9, 25.8. Only the signals for the major form are reported.

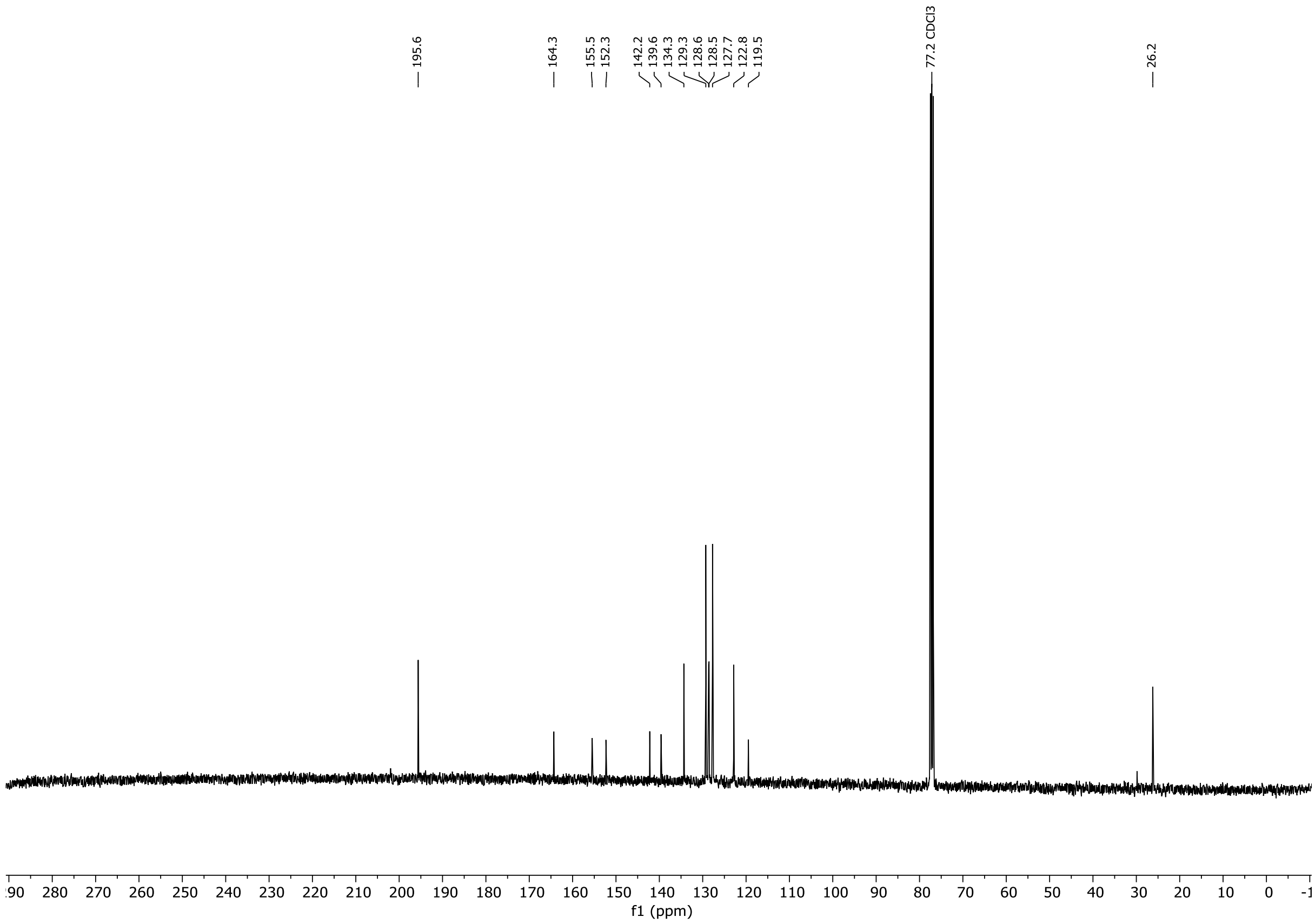

MS (ESI^+): m/z [M + H^+] 305.17. Calculated MS for $C_{17}H_{17}N_6$: 305.15.

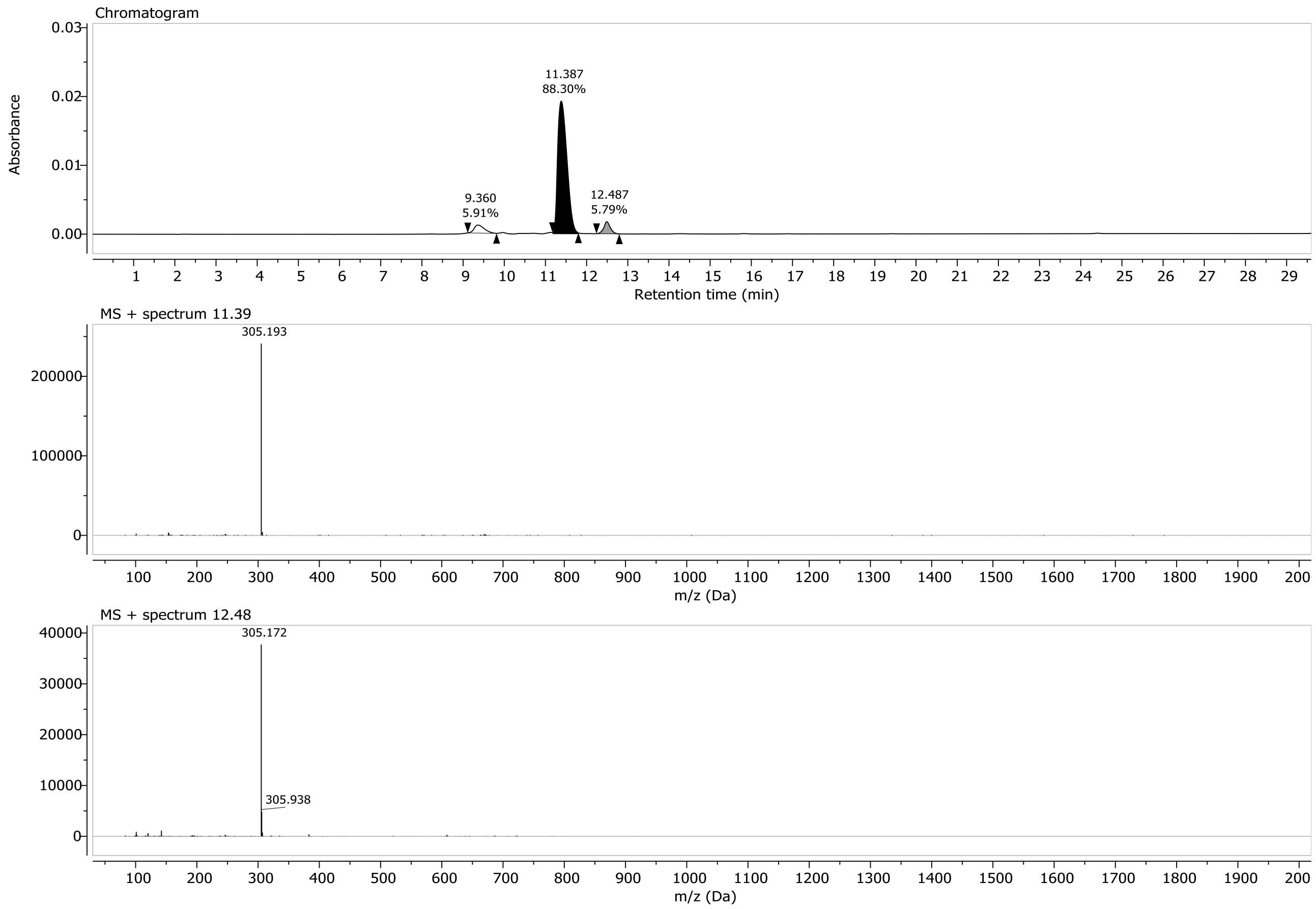

Synthesis of 4-((2-methyl-6-morpholinoquinazolinyl)methylene)hydrazine-1-carboximidamide hydrochloride 2e

The synthesis of 4-((2-methyl-6-morpholinoquinazolinyl)methylene)hydrazine-1-carboximidamide hydrochloride **2e** is described in the main text. The analytical characterization of intermediate (2,4-dimethylquinazolin-2-yl)methanol **9e**, methyl-6-morpholinoquinazoline-4-carbaldehyde **10e** and 4-((2-methyl-6-morpholinoquinazolinyl)methylene)hydrazine-1-carboximidamide hydrochloride **2e** is reported below.

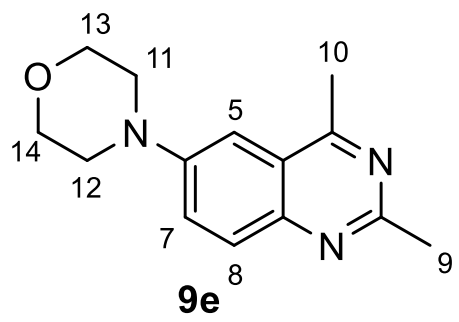

9e

^{1}H-NMR (400 MHz, $CDCl_3$) δ 7.82 (d, *J* = 9.2 Hz, 1H, H8), 7.58 (dd, *J* = 9.2, 2.7 Hz, 1H, H7), 7.14 (d, *J* = 2.7 Hz, 1H, H5), 3.94 – 3.87 (m, 4H, H11, H12), 3.30 – 3.23 (m, 4H, H13, H14), 2.84 (s, 3H, H10), 2.79 (s, 3H, H9).
^{13}C-NMR (101 MHz, $CDCl_3$) δ 166.3, 161.2, 149.5, 145.5, 129.1, 126.0, 123.1, 106.0, 66.9, 49.5, 26.2, 21.9.

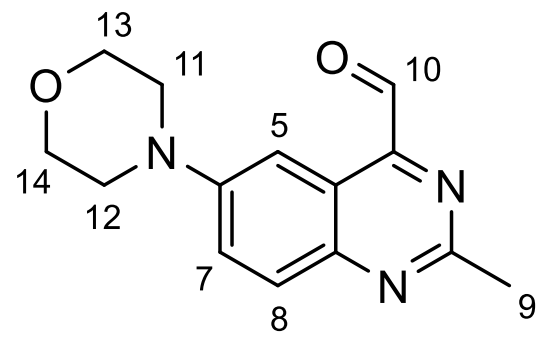

10e

^{1}H-NMR (400 MHz, $CDCl_3$) δ 10.28 (s, 1H, H10), 8.31 (d, *J* = 2.8 Hz, 1H, H5), 7.92 (d, *J* = 9.5 Hz, 1H, H8), 7.70 (dd, *J* = 9.5, 2.8 Hz, 1H, H7), 3.95 – 3.89 (m, 4H, H11, H12), 3.41 – 3.34 (m, 4H, H13, H14), 2.94 (s, 3H, H9).

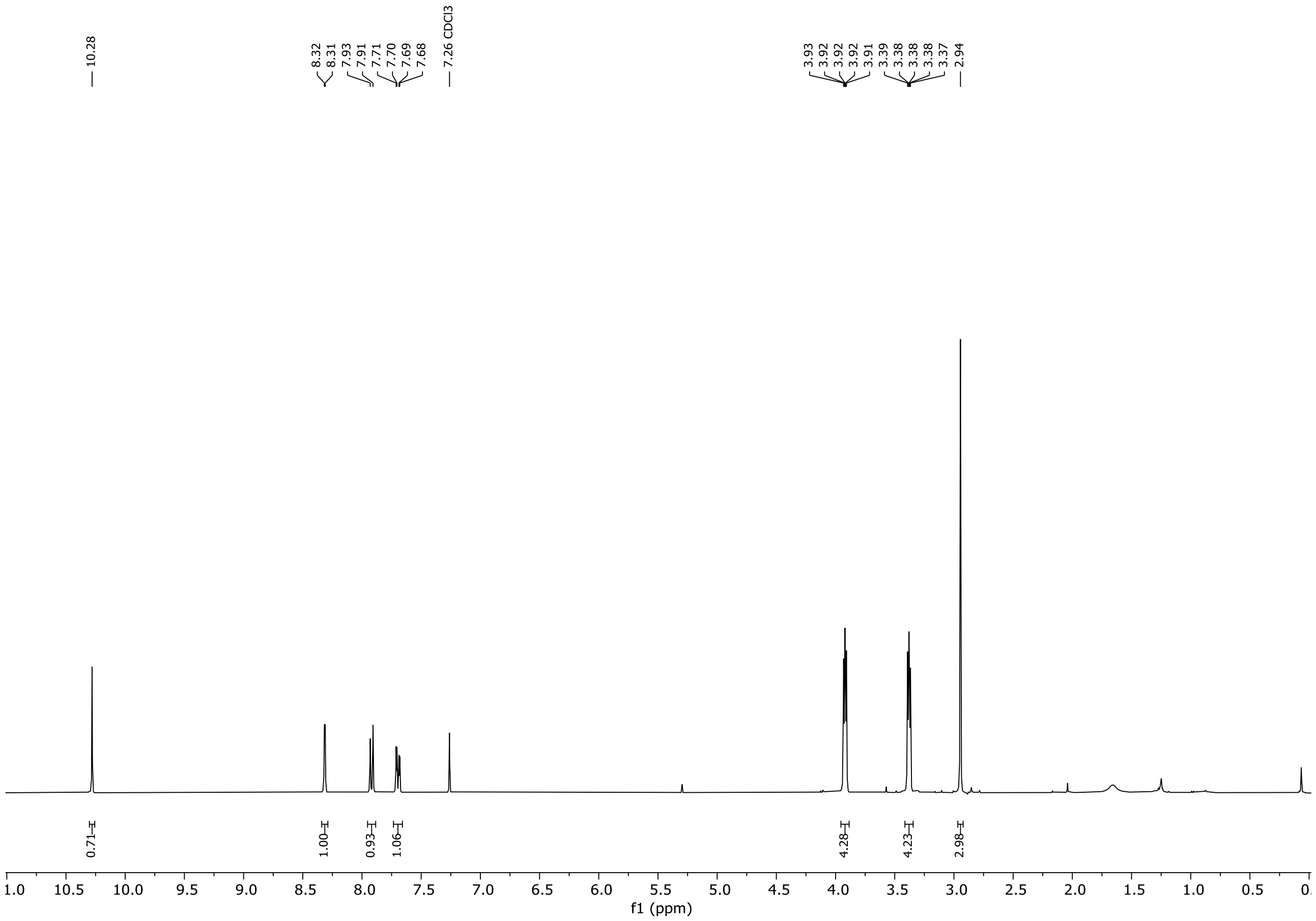

^{13}C-NMR (101 MHz, $CDCl_3$) δ 196.6, 161.8, 153.2, 151.7, 149.6, 129.3, 126.7, 121.1, 105.1, 67.2, 67.1, 49.4, 49.0, 26.2.

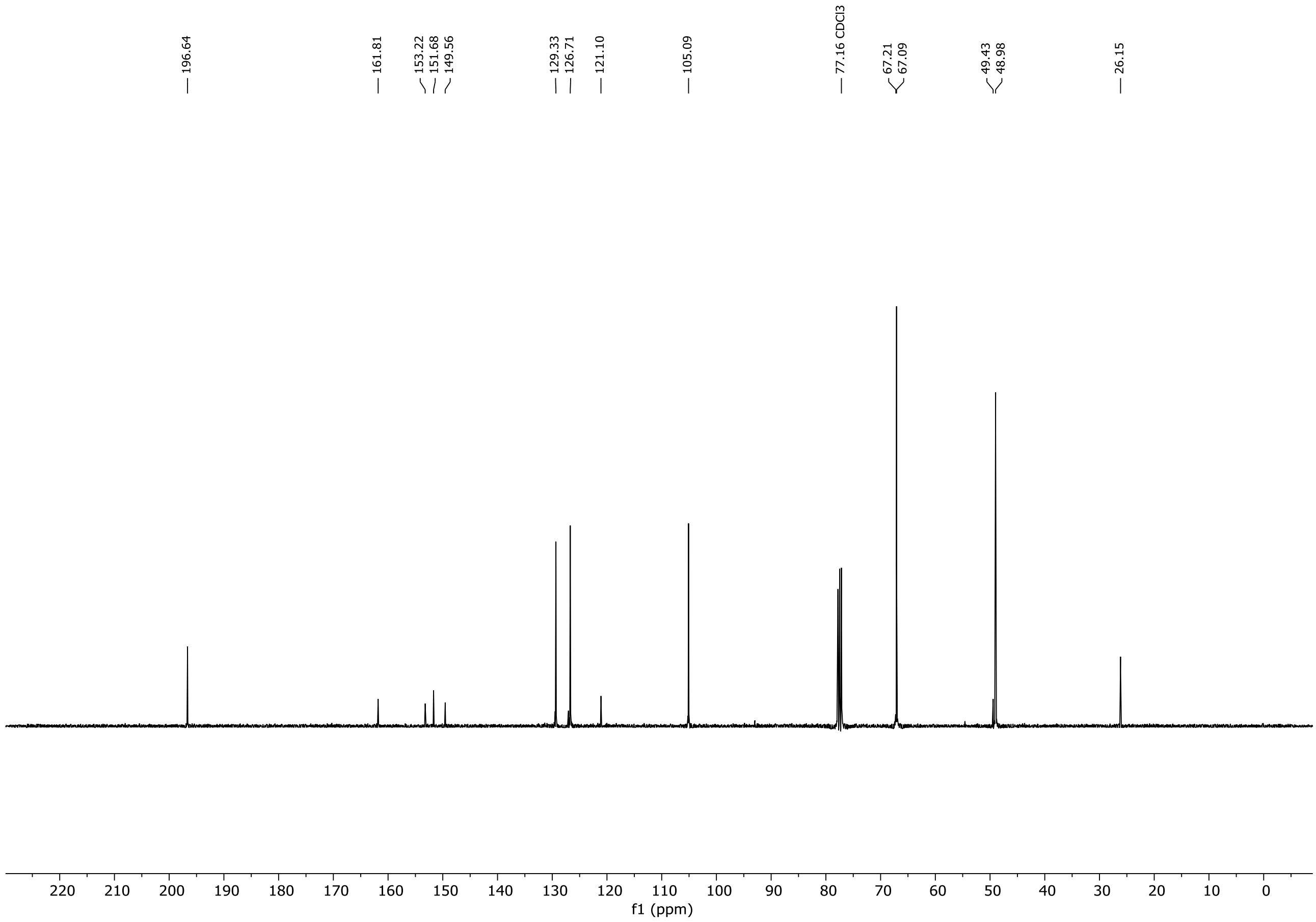

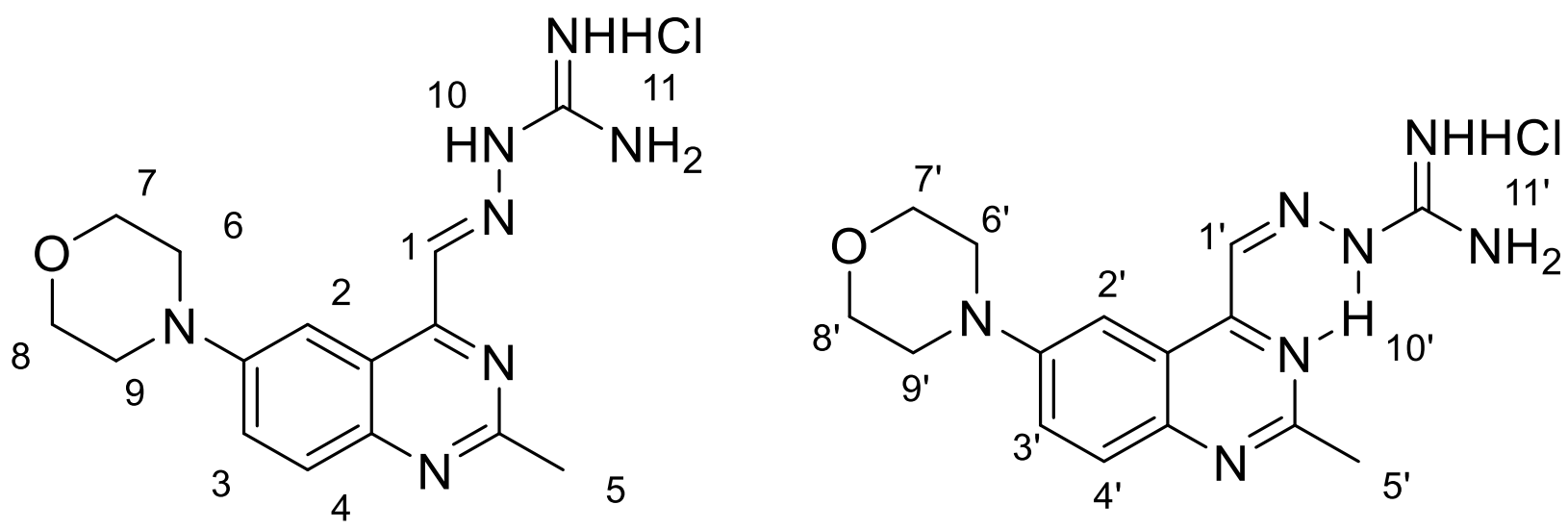

2eII **2eI**

^{1}H NMR (400 MHz, DMSO-d_6) δ (ppm) 12.32 (s, 1H, H10), 8.66 (s, 1H, H1'), 8.49 (s, 1H, H1), 8.33 (brs, 4H, H11'), 8.08 (d, *J* = 2.6 Hz, 1H, H2), 7.96 (dd, *J* = 9.4, 2.4 Hz, 1H, H2'), 7.88 (dd, *J* = 9.3, 2.6 Hz, 1H, H3), 7.81 (d, *J* = 9.3 Hz, 1H, H4), 7.75 (brs, 4H, H11), 4.07 (q, *J* = 4.9 Hz, 4H, H7', H8'), 3.41 (t, *J* = 4.9 Hz, 4H, H6', H9'), 3.77 (dd, *J* = 6.0, 3.4 Hz, 4H, H7, H8), 3.35 – 3.29 (m, 4H, H6, H9), 2.88 (s, 3H, H5'), 2.70 (s, 3H, H5).

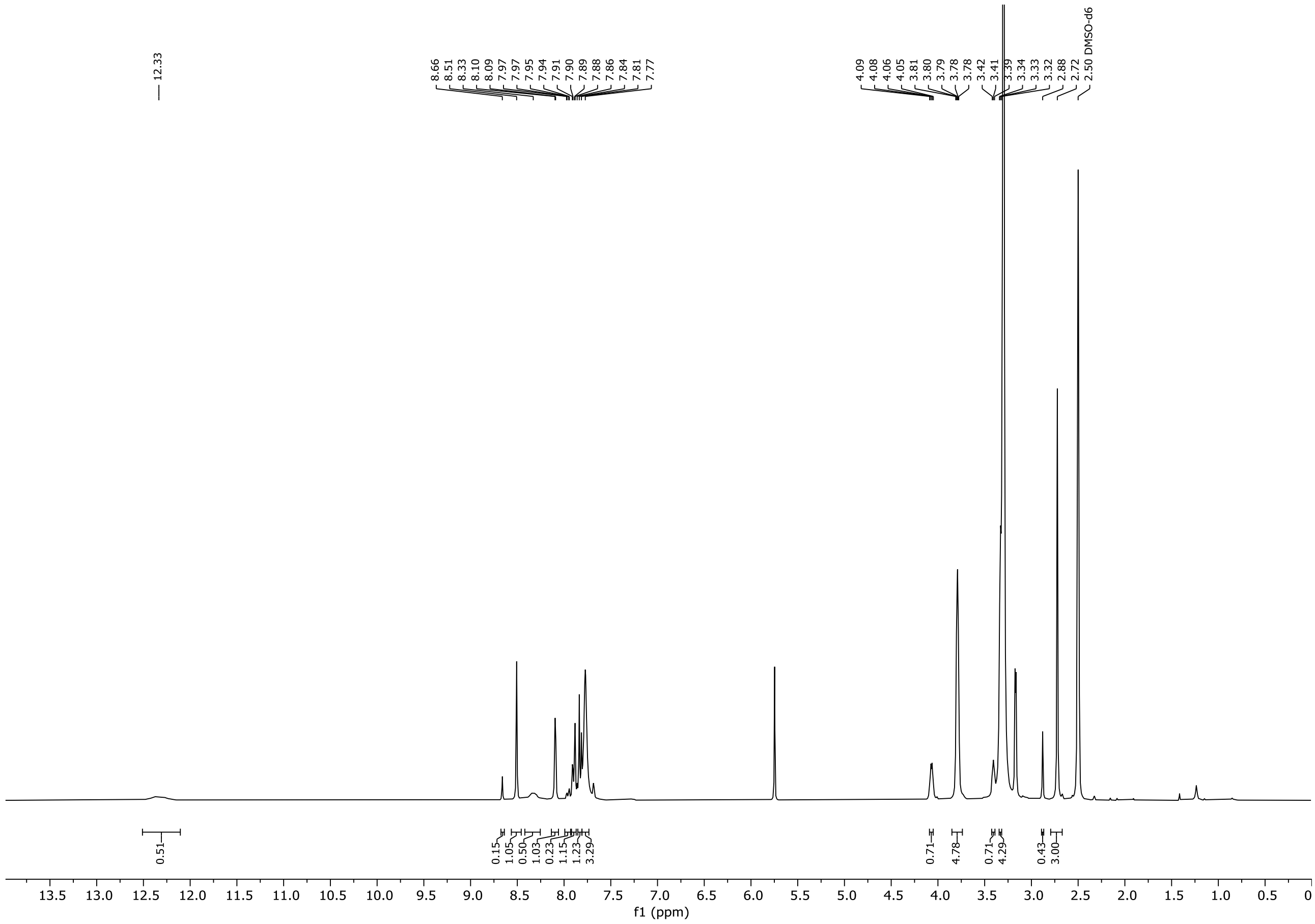

^{13}C NMR (101 MHz, DMSO-d_6) δ 160.0, 156.1, 155.7, 155.6, 150.0, 147.4, 147.3, 146.9, 128.4, 126.4, 120.7, 105.9, 66.0, 47.9, 25.4.

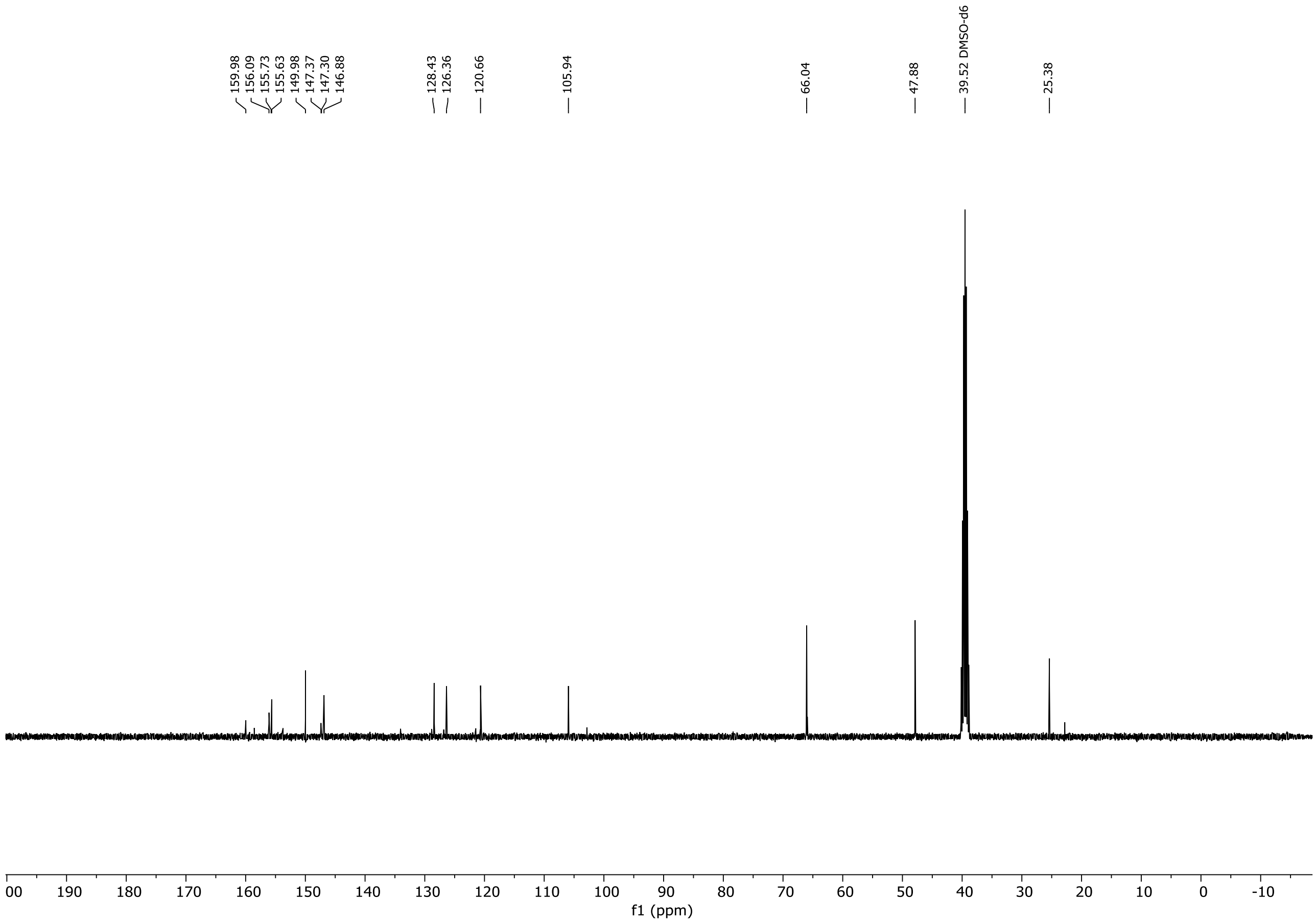

MS (ESI$^+$): m/z [M + H$^+$] 314.12. Calculated MS for $C_{15}H_{20}N_7O$: 314.17.

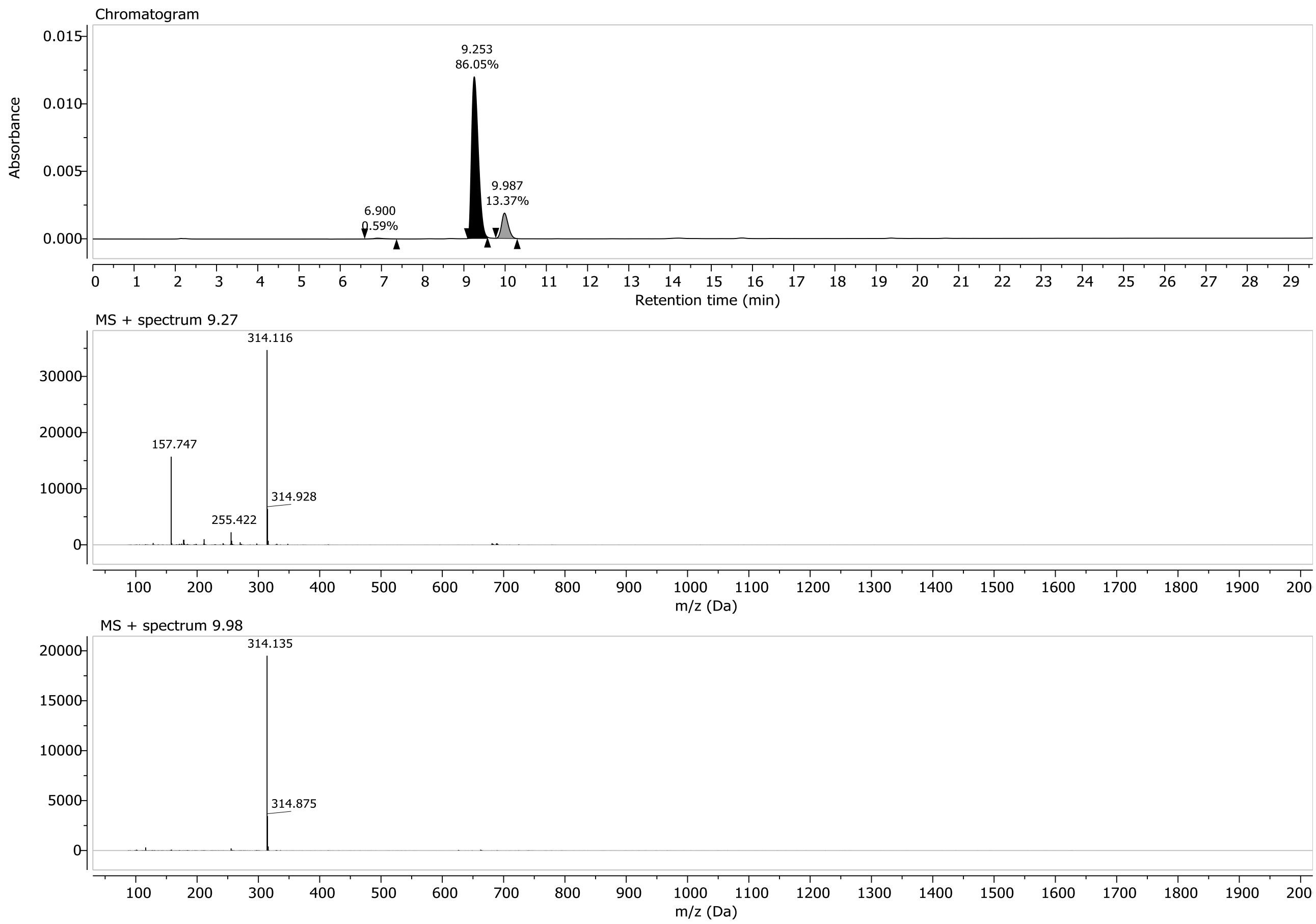

Synthesis of 4-((2-methyl-[1,3]dioxolo[4,5-g]quinazolinyl)methylene)hydrazine-1-carboximidamide hydrochloride 2f

Synthesis of 6,8-dimethyl-[1,3]dioxolo[4,5-g]quinazoline 9f

The reaction was performed according to **general procedure D**, using NH_4OAc (548 mg, 7.11 mmol, 3.0 eq), 1-(6-aminobenzo[d][1,3]dioxol-5-yl)ethan-1-one **8f** (150 mg, 0.84 mmol, 1.0 eq) and acetylacetone (101 mg, 1.00 mmol, 1.2 eq). Pure 6,8-dimethyl-[1,3]dioxolo[4,5-g]quinazoline **9f** was obtained as a brown solid (119 mg, 0.59 mmol, 70% yield).

Reaction time: 7 hours.

TLC monitoring: eluent mixture 3:7 *n*-Hex/EtOAc.

Direct phase flash column chromatography: eluent mixture 3:7 *n*-Hex/EtOAc.

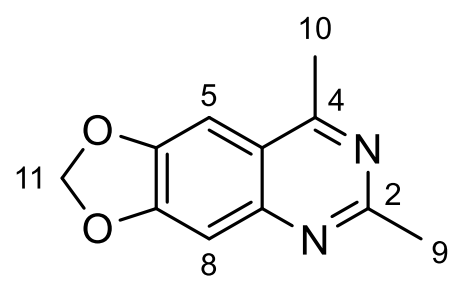

^{1}H-NMR (400 MHz, $CDCl_3$) δ 7.25 (s, 1H, H5), 7.21 (s, 1H, H8), 6.13 (s, 2H, H11), 2.80 (s, 3H, H9), 2.78 (s, 3H, H10). Spectroscopic data are consistent with the literature [53].

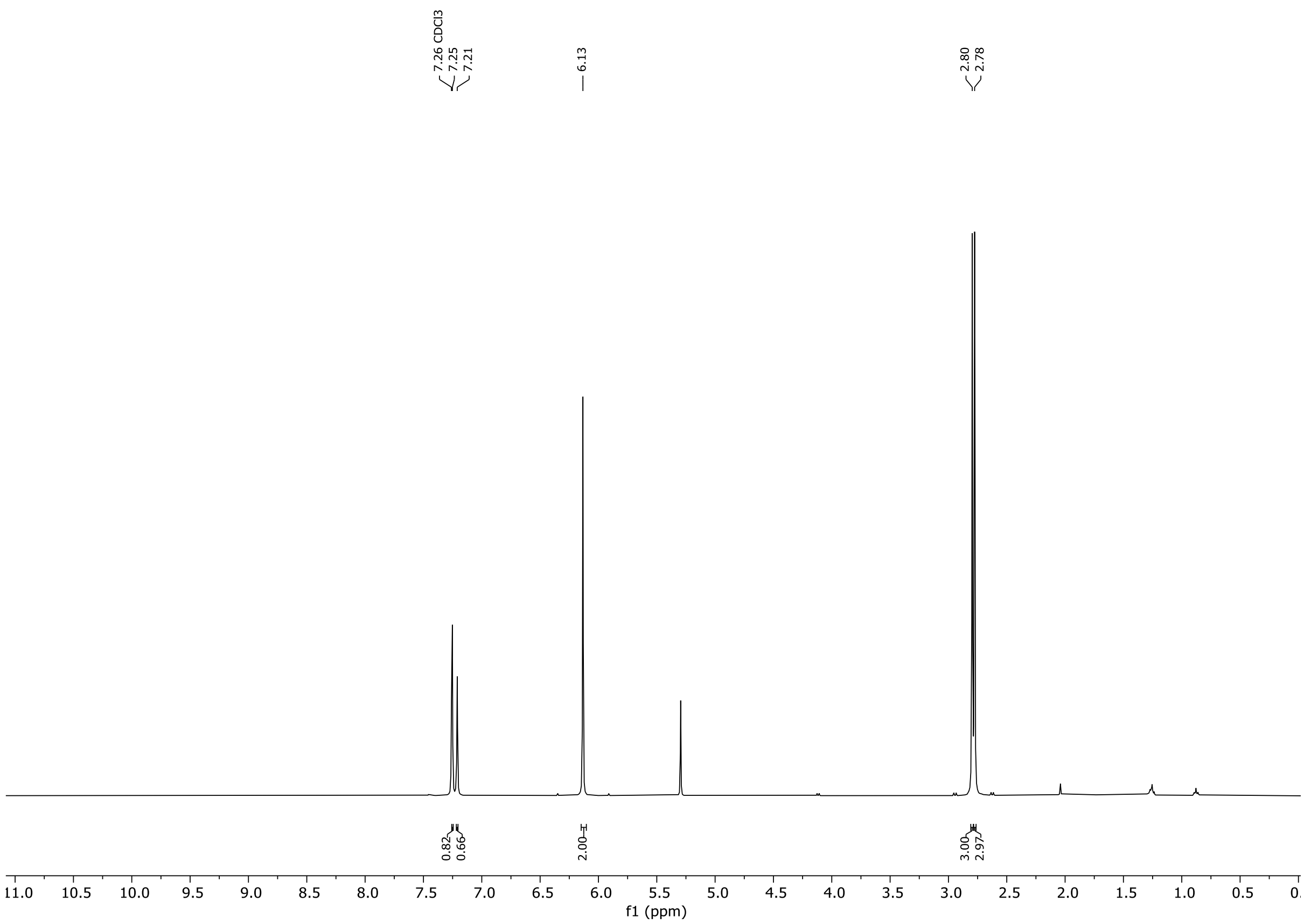

Synthesis of methyl-[1,3]dioxolo[4,5-g]quinazoline 4-carbaldehyde 10f

The reaction was performed according to **general procedure E**, using 6,8-dimethyl-[1,3]dioxolo[4,5-g]quinazoline **9f** (90 mg, 0.45 mmol, 1 eq), SeO_2 (50 mg, 0.45 mmol, 1.0 eq.) and 1,4-dioxane (2.25 mL). Washing after Celite pad filtration was done using EtOAc (125 mL). After purification, pure methyl-[1,3]dioxolo[4,5-g]quinazoline 4-carbaldehyde **10f** was obtained as a yellow solid (69 mg, 0.32 mmol, 71% yield).

Reaction time: 30 min.

TLC monitoring: eluent mixture 1:1 *n*-Hex/EtOAc.

Direct phase flash column chromatography: eluent mixture *n*-Hex/EtOAc from 1:1 to 3:7.

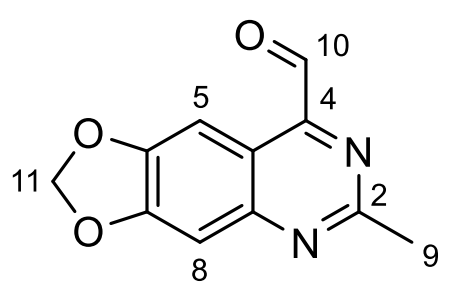

^{1}H-NMR (400 MHz, $CDCl_3$) δ 10.21 (s, 1H, H10), 8.36 (s, 1H, H5), 7.28 (s, 1H, H8), 6.19 (s, 2H, H11), 2.91 (s, 3H, H9).

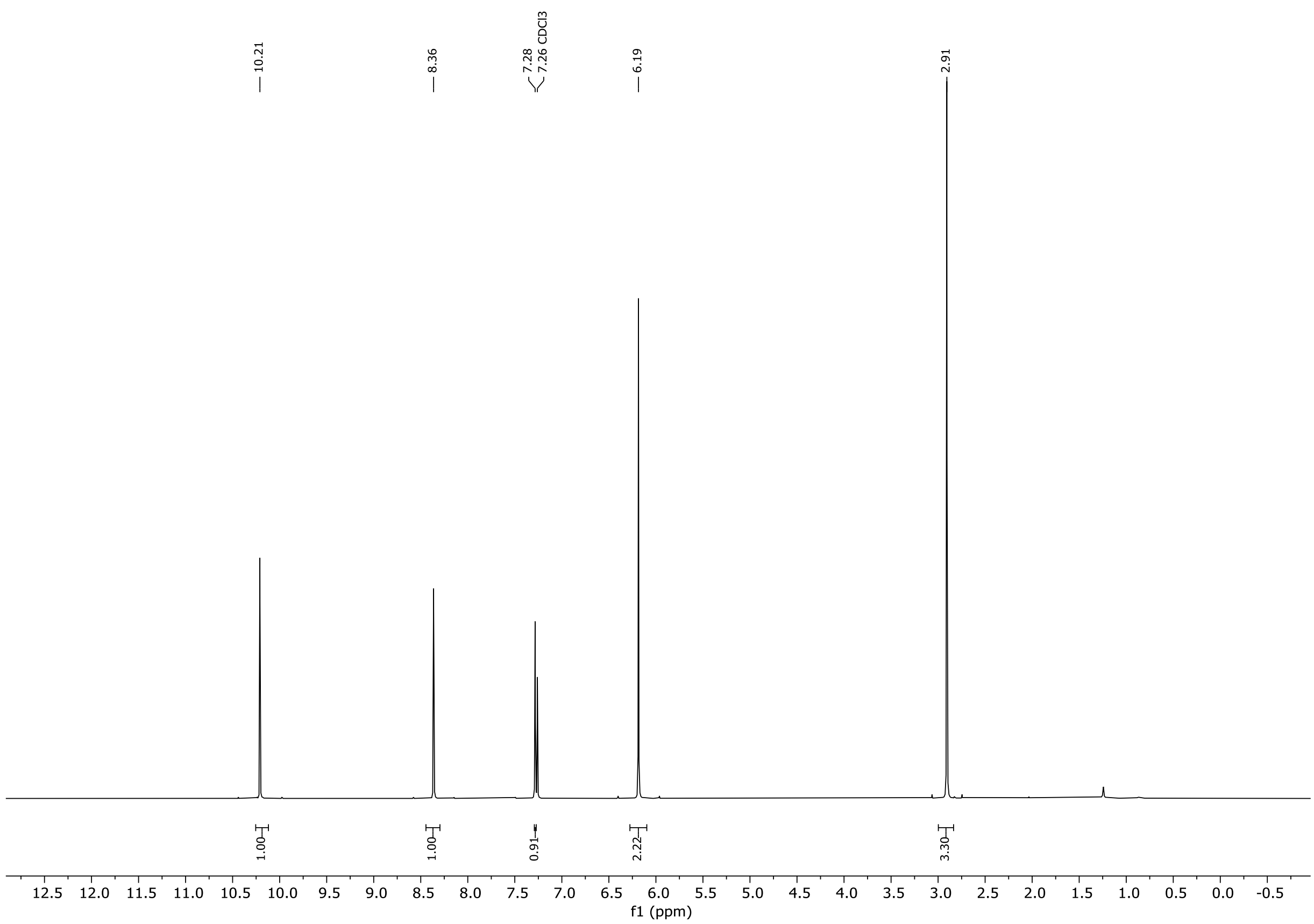

^{13}C-NMR (101 MHz, $CDCl_3$) δ 196.0, 163.0, 154.7, 152.8, 150.6, 116.8, 104., 102.7, 100.4, 25.8.

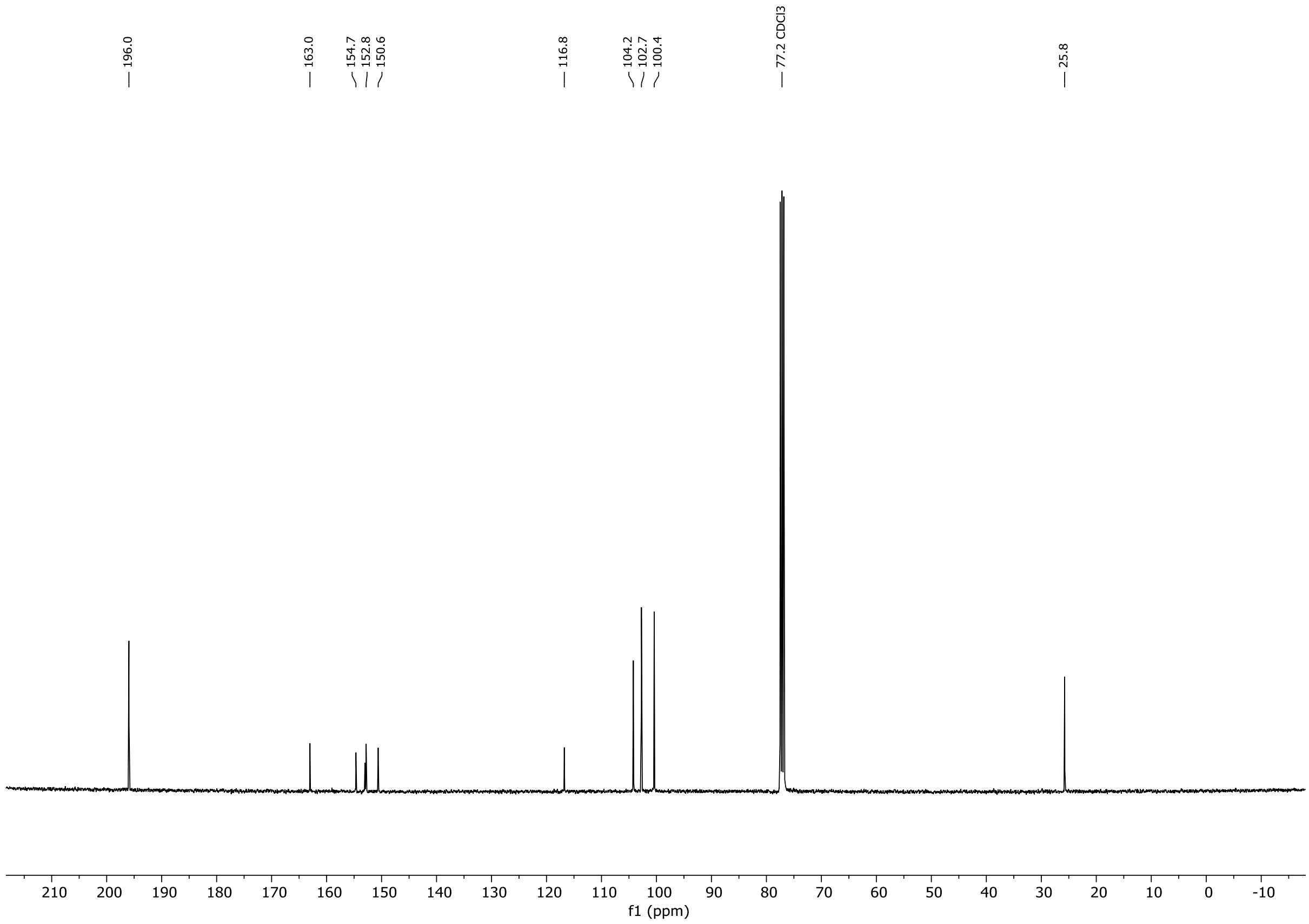

Synthesis of 4-((2-methyl-[1,3]dioxolo[4,5-g]quinazolinyl)methylene)hydrazine-1-carboximidamide hydrochloride 2f

The reaction was performed according to **general procedure C**, using methyl-[1,3]dioxolo[4,5-g]quinazoline 4-carbaldehyde **10f** (41 mg, 0.19 mmol, 1 eq.), AG·HCl (19 mg, 0.17 mmol, 0.90 eq.) and EtOH (1.9 mL). Trituration was done in Et_2O (2 x 2.5 mL). After purification, pure 4-((2-methyl-[1,3]dioxolo[4,5-g]quinazolinyl)methylene)hydrazine-1-carboximidamide hydrochloride **2f** was recovered as a yellow solid (22 mg, 0.08 mmol, quantitative yield).

1:0.15 E/Z ratio (87%/13%).

Reaction time: 7 hours, then rt overnight.

TLC monitoring: eluent mixture 1:1 DCM/EtOAc.

BIOTAGE® Reverse phase flash column chromatography: eluent mixture from 0% to 100% CH_3CN in H_2O.

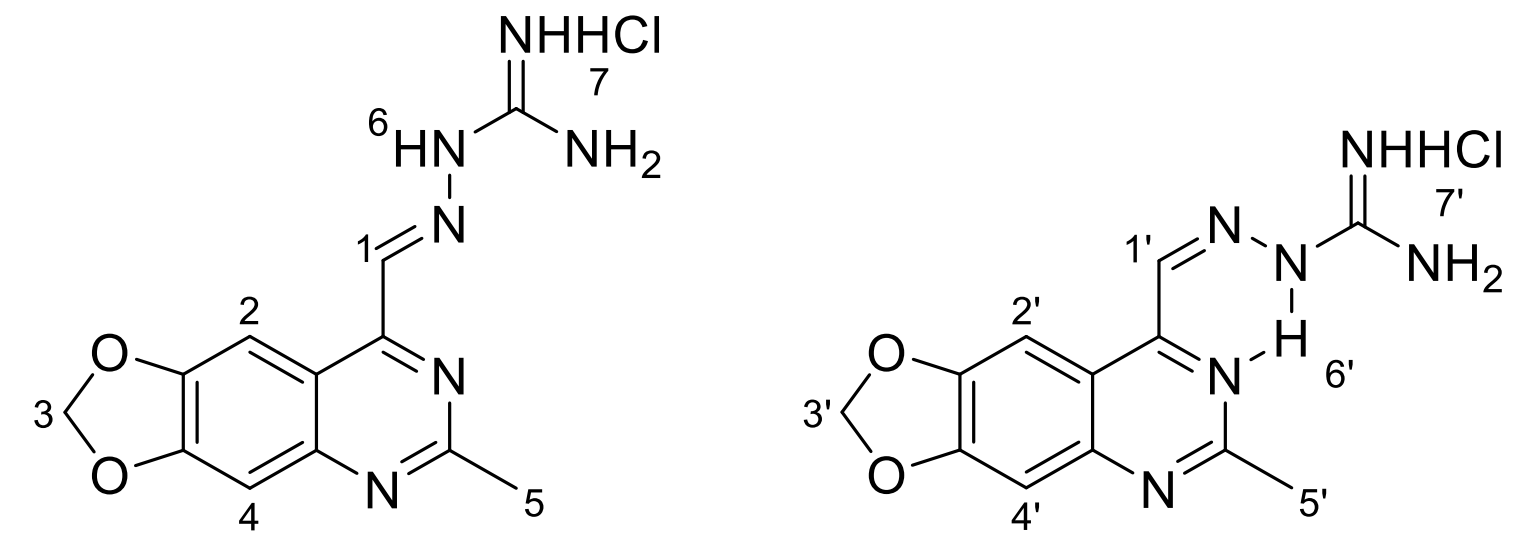

^{1}H NMR (400 MHz, DMSO-d_6) δ (ppm) 14.23 (s, 1H, H6'), 12.61 (s, 1H, H6), 8.52-8.38 (brs, 4H, H7'), 8.47 (s, 1H, H2), 8.44 (s, 1H, H2'), 8.22 (s, 1H, H1), 8.12 (s, 1H, H1'), 8.03 (brs, 4H, H7), 7.33 (s, 1H, H4'), 7.32 (s, 1H, H4), 6.34 (s, 2H, H3'), 6.31 (s, 2H, H3), 2.86 (s, 3H, H5'), 2.70 (s, 3H, H5).

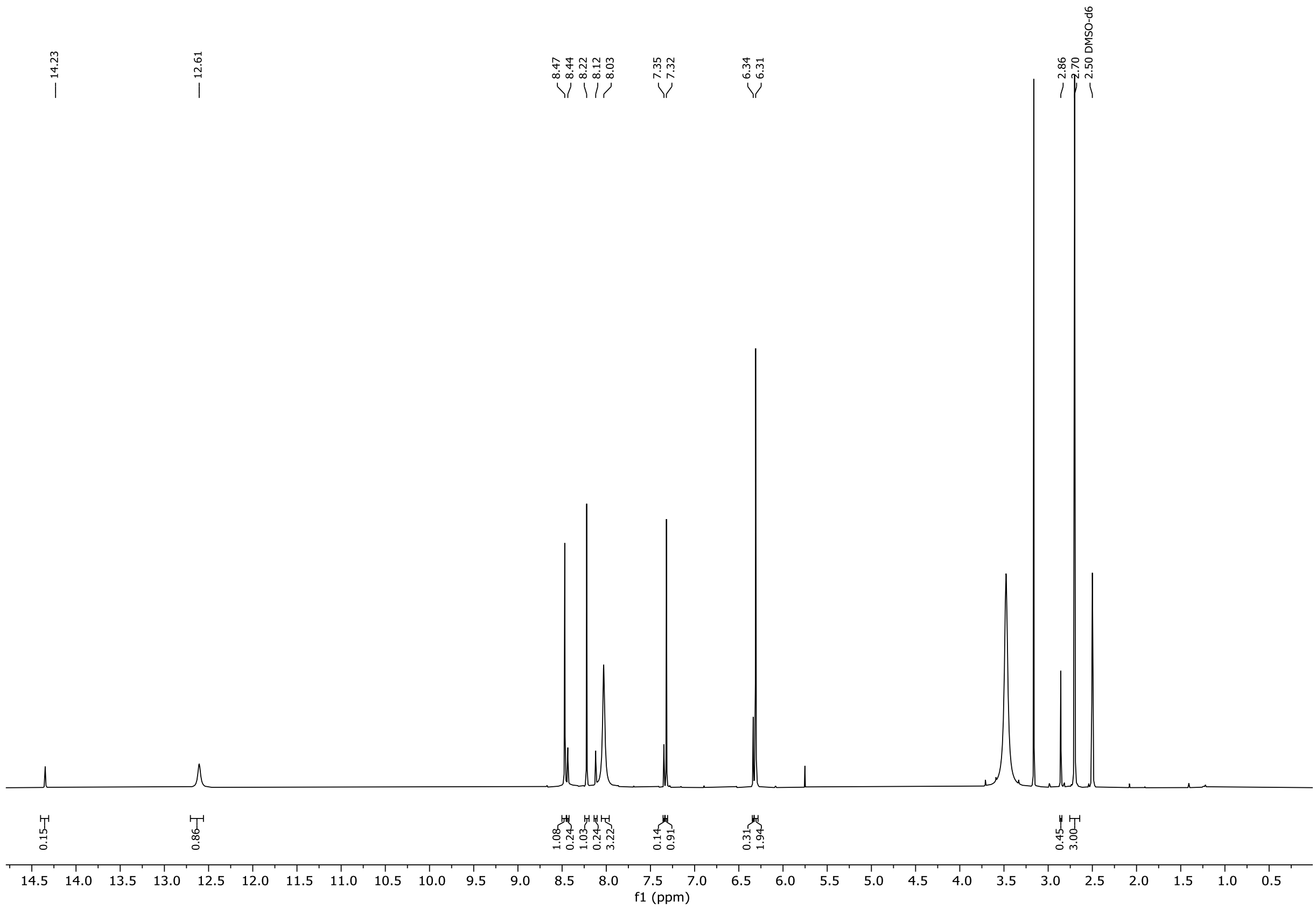

^{13}C NMR (101 MHz, DMSO-d_6) δ 161.4, 155.4, 154.8, 154.1, 151.3, 149.0, 148.0, 133.8, 117.4, 103.1, 99.2, 48.6, 25.2.

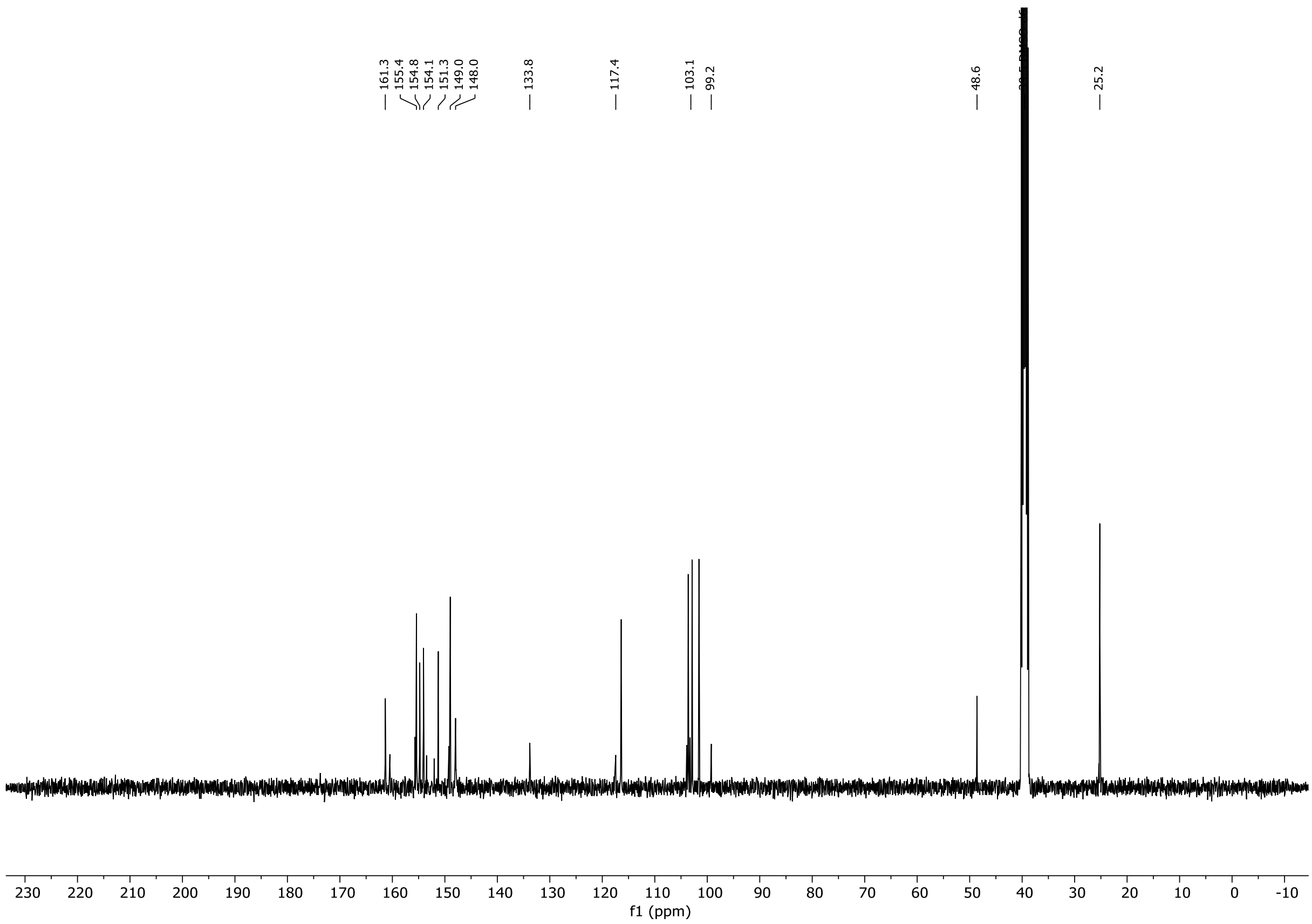

MS (ESI^+): m/z [M + H^+] 273.03. Calculated MS for $C_{12}H_{13}N_6O_2$: 273.11.

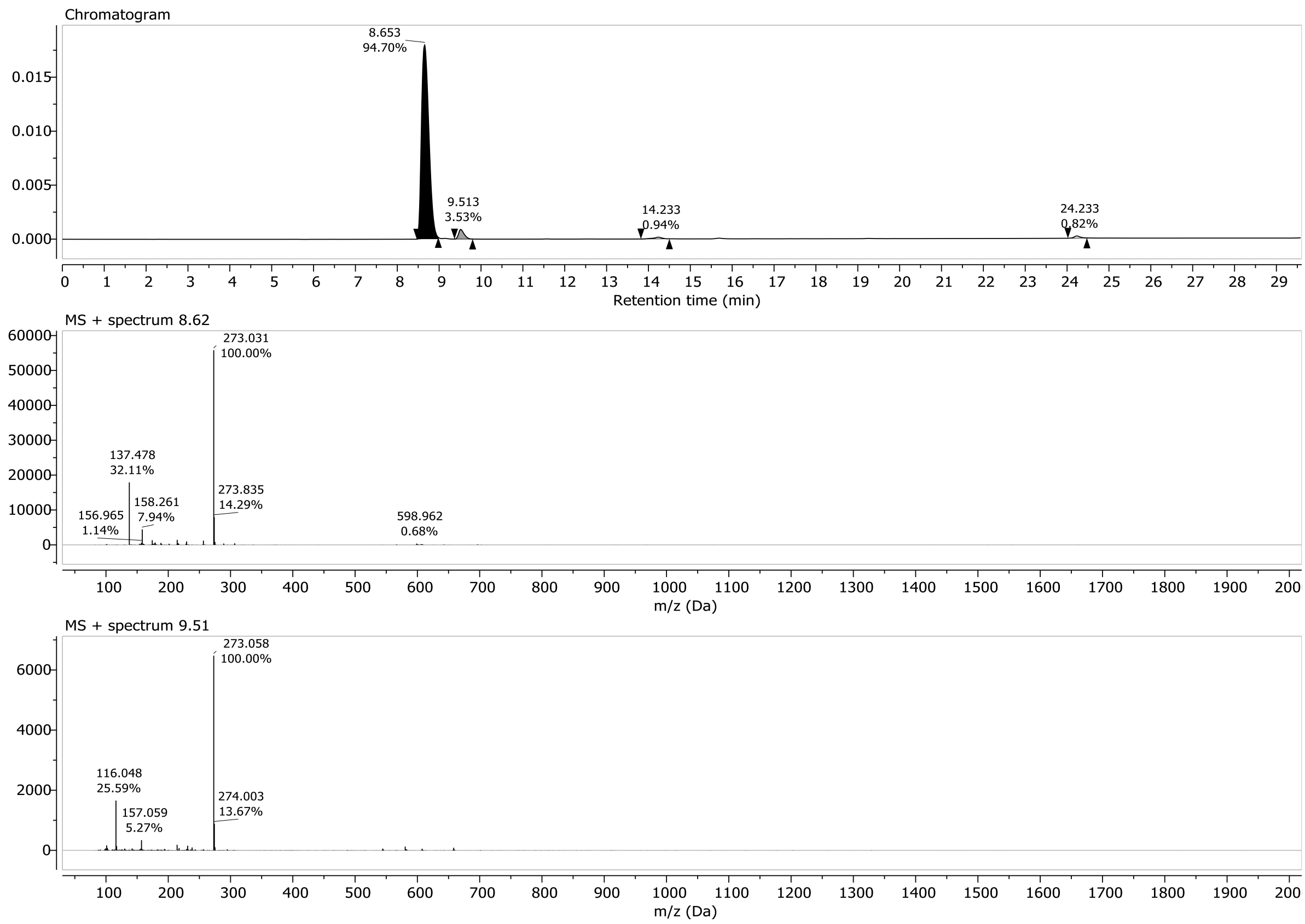
Chromatogram
8.653
94.70%
9.513
3.53%
14.233
0.94%
24.233
0.82%
Retention time (min)
MS + spectrum 8.62
273.031
100.00%
137.478
32.11%
158.261
7.94%
156.965
1.14%
273.835
14.29%
598.962
0.68%
m/z (Da)
MS + spectrum 9.51
273.058
100.00%
116.048
25.59%
157.059
5.27%
274.003
13.67%
m/z (Da)

Synthesis of GMQ-like heteroaryl GHs 3-5 – scaffold hopping

Synthesis of 2-((4-methylquinolin-2-yl)methylene)hydrazinecarboximidamide hydrochloride 3

Synthesis of (4-methylquinolin-2-yl)methanol 12

$Na_2S_2O_8$ (665.2 mg, 2.79 mmol, 2 eq.) and p-TsOH (312.7 mg, 1.82 mmol, 1.3 eq.) were added to a solution of lepidine **11** (0.185 mL, 1.40 mmol, 1 eq.) in 7:3 MeOH/H_2O (14.0 mL) at room temperature, under nitrogen atmosphere. The mixture was heated to 70°C for 4 hours and monitored by TLC (eluent mixture: 1:1 *n*-Hex/EtOAc). After cooling to room temperature, the solvent was removed and a saturated solution of $NaHCO_3$ was added. The mixture was extracted with DCM (3 x 20 mL) and the organic phase was washed with brine (20 mL). The organic phase was dried over anhydrous Na_2SO_4 and concentrated under vacuum. The crude was purified with flash chromatography (eluent mixture: 1:1 *n*-Hex/EtOAc) to obtain the pure (4-methylquinolin-2-yl)methanol **12** as a light yellow solid (213.0 mg, 1.23 mmol, 88 % yield).

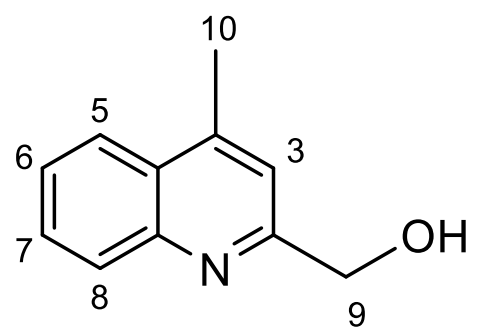

^{1}H-NMR (400 MHz, $CDCl_3$) δ 8.07 (d, 1H, H8), 7.98 (d, 1H, H5), 7.76 – 7.67 (m, 1H, H7), 7.60 – 7.52 (m, 1H, H6), 7.12 (s, 1H, H3), 4.87 (d, 2H, H9), 2.70 (d, 3H, H10).

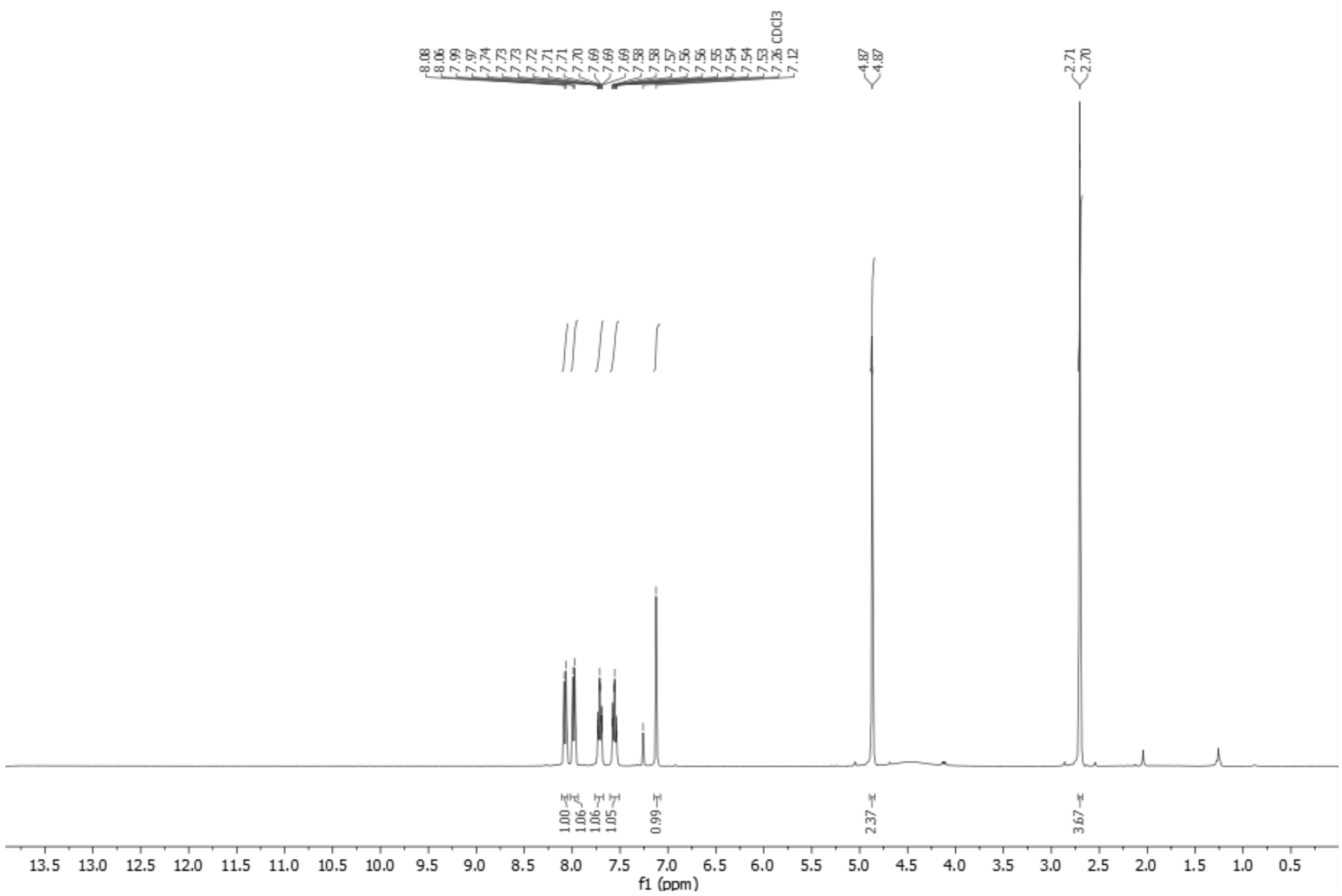

Synthesis of 4-methylquinoline-2-carbaldehyde 13

The reaction was performed according to **general procedure B**, using (4-methylquinolin-2-yl)methanol **12** (100.0 mg, 0.577 mmol, 1 eq.), DMP (489.9 mg, 1.155 mmol, 2 eq.) and dry DCM (2.90 mL). After purification, pure 4-methylquinoline-2-carbaldehyde **13** was obtained as a dark solid (22.4 mg, 0.13 mmol, 23% yield).

Reaction time: 7 hours.

TLC monitoring: eluent mixture 1:1 *n*-Hex/EtOAc.

Direct phase flash column chromatography: eluent mixture 9:1 *n*-Hex/EtOAc.

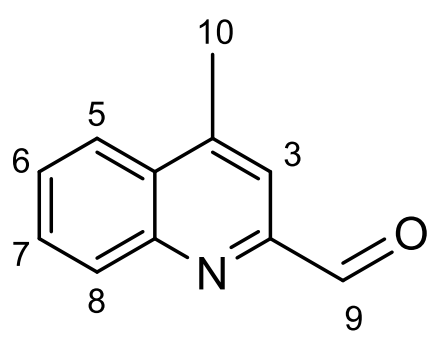

^{1}H-NMR (400 MHz, $CDCl_3$) δ 10.19 (s, 1H, H9), 8.24 (dd, 1H, H8), 8.05 (dd, 1H, H5), 7.86 (s, 1H, H3), 7.80 (ddd, 1H, H7), 7.70 (ddd, 1H, H6), 2.77 (s, 3H, H10).

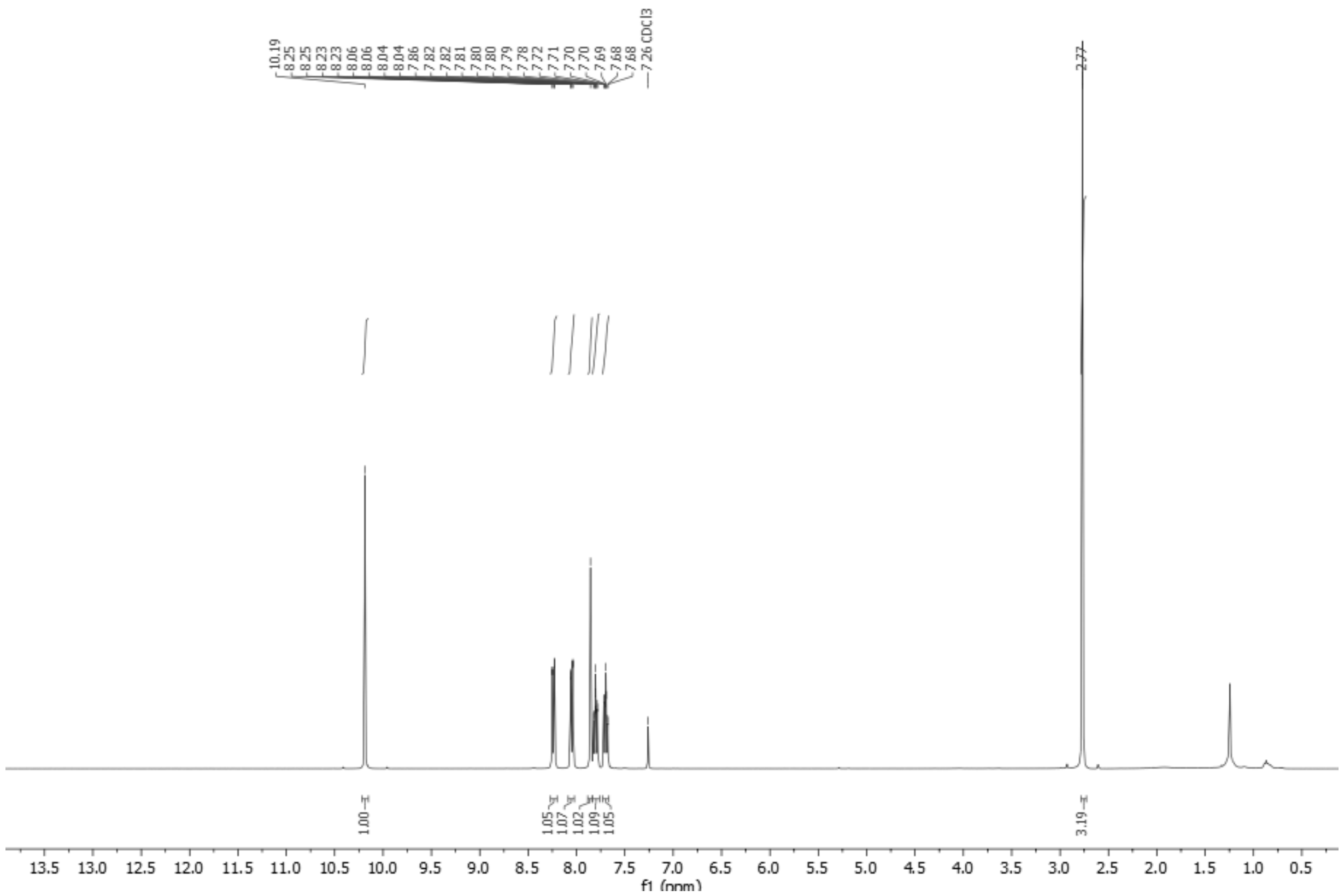

^{13}C-NMR (101 MHz, $CDCl_3$) δ 194.2, 152.3, 147.8, 146.2, 131.2, 130.2, 130.2, 129.1, 124.2, 118.0, 19.1.

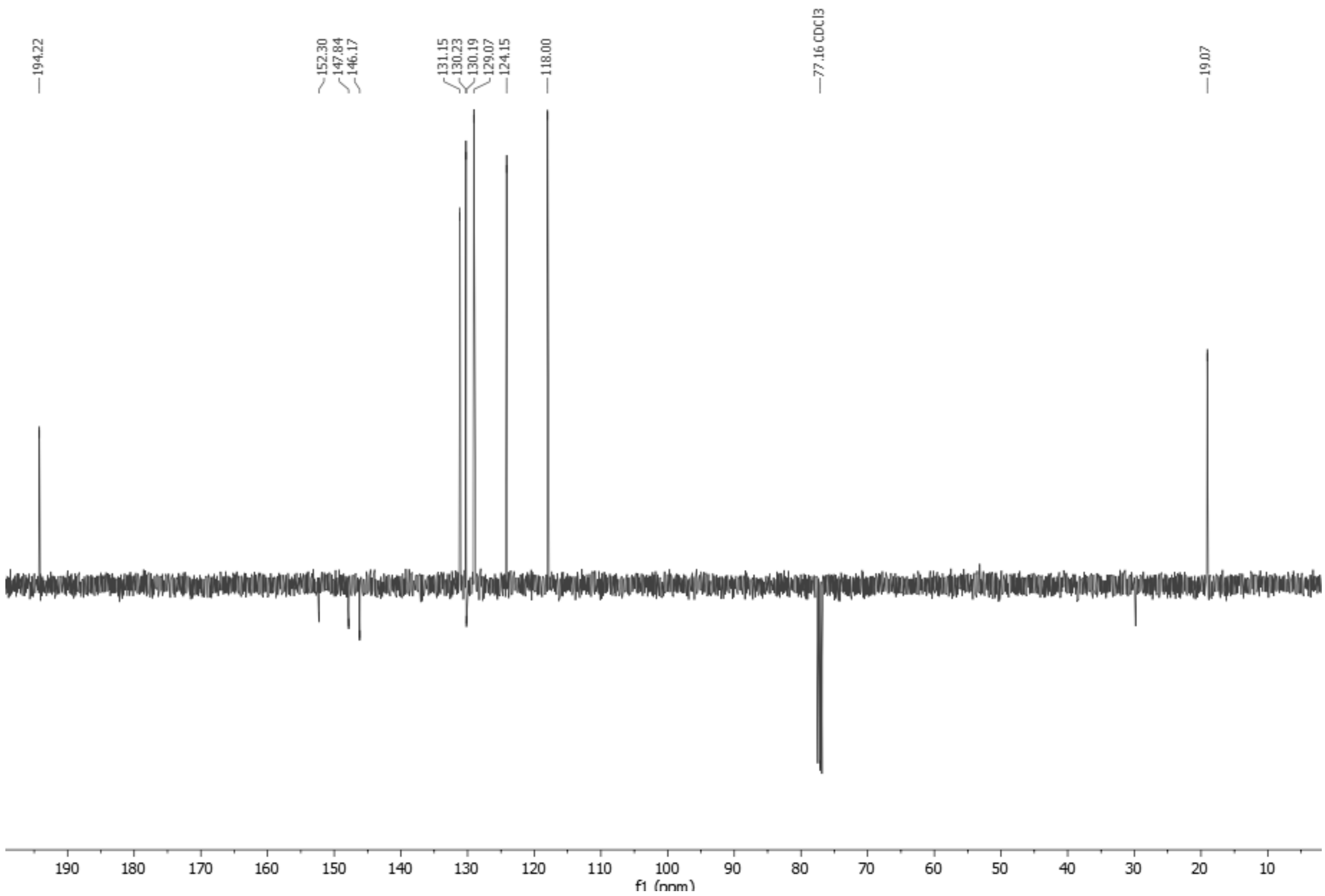

Synthesis of 2-((4-methylquinolin-2-yl)methylene)hydrazinecarboximidamide hydrochloride 3

The reaction was performed according to **general procedure C**, using 4-methylquinoline-2-carbaldehyde **13** (22.0 mg, 0.128 mmol, 1 eq.), AG.HCl (13.6 mg, 0.123 mmol, 0.96 eq.) and EtOH (1.28 mL). Five drops of HCl 1M were added to the mixture prior to heating. Trituration was done in Et_2O (2 x 2.5 mL). Pure target 2-((4-methylquinolin-2-yl)methylene)hydrazinecarboximidamide hydrochloride **3** was obtained as a yellow solid (31.2 mg, 0.118 mmol, 92% yield).

E/Z ratio: E-isomer only.

Reaction time: 5 hours, then rt overnight.

TLC monitoring: eluent mixture 9:1 DCM/EtOAc.

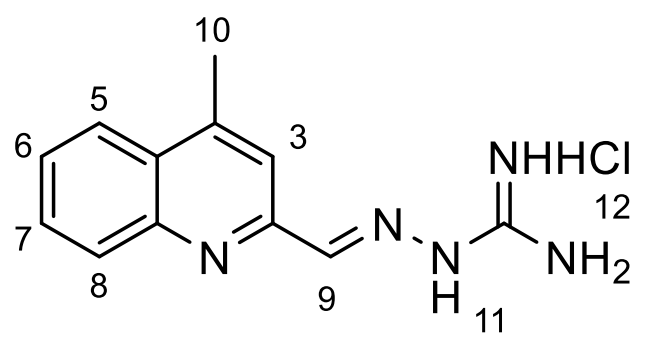

^{1}H NMR (400 MHz, DMSO-d_6) δ 12.14 (s, 1H, H11), 8.32 (s, 1H, H9), 8.26 (s, 1H, H3), 8.12 (dd, 1H, H8), 8.05 – 7.85 (m, 5H, H5, H12), 7.80 (ddd, 1H, H7), 7.68 (ddd, 1H, H6), 2.73 (d, 3H, H10).

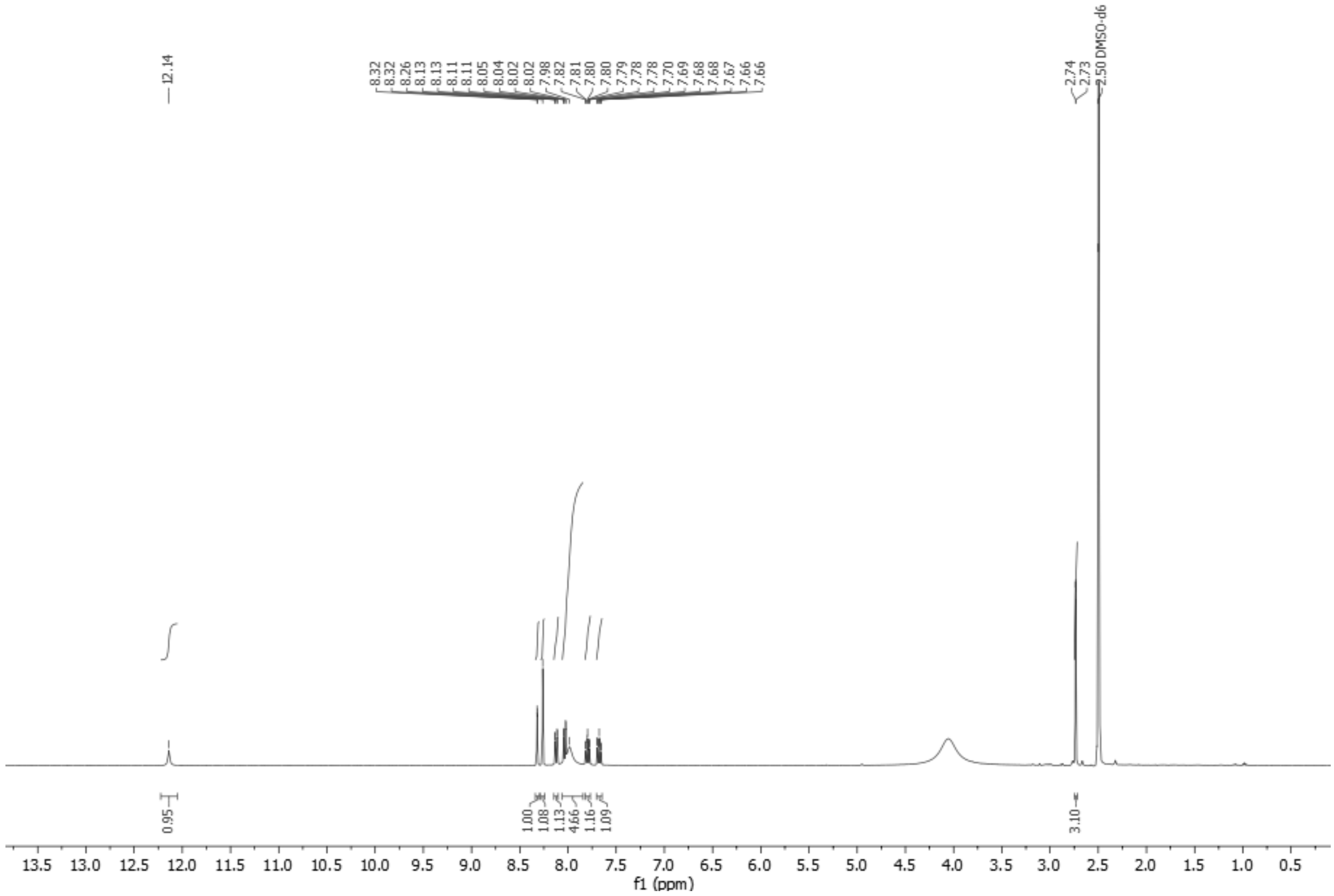

^{13}C NMR (101 MHz, DMSO-d_6) δ 155.5, 150.7, 150.0, 142.6, 142.6, 132.1, 128.5, 128.0, 125.7, 125.1, 119.5, 19.02

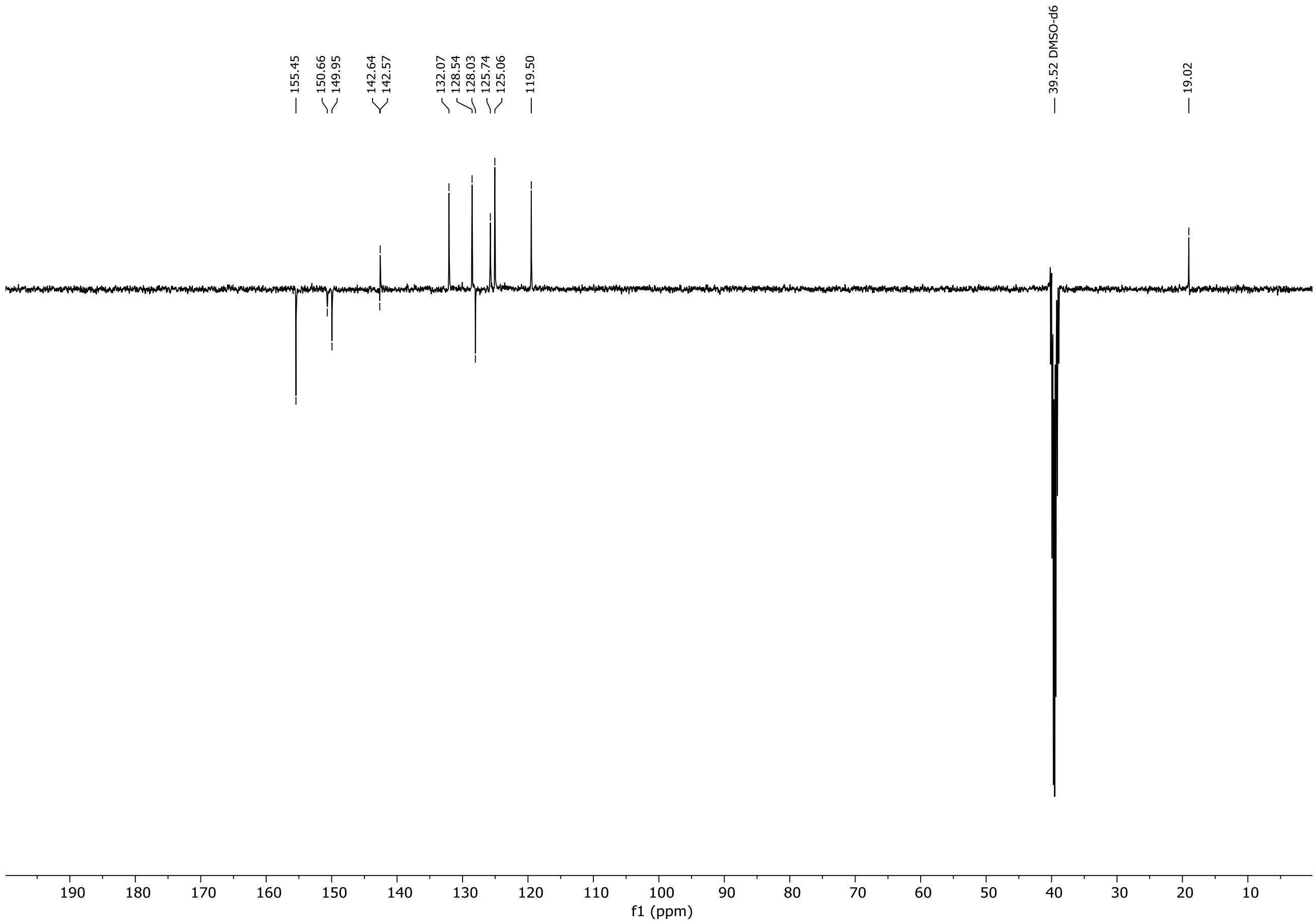

MS (ESI^+): m/z 228.17 [M + H^+]. Calculated MS for $C_{12}H_{13}N_5$: 227.12.

Synthesis of 2-(quinoxalinylmethylene)hydrazinecarboximidamide hydrochloride 4

The reaction was performed according to **general procedure C**, using quinoxaline-2-carboxaldehyde **14** (100 mg, 0.632 mmol, 1 eq.), AG.HCl (67.1 mg, 0.607 mmol, 0.96 eq.) and EtOH (6.32 mL). Five drops of HCl 1M were added to the mixture prior to heating. Trituration was done in Et_2O (2 x 2.5 mL). Pure target 2-(quinoxalinylmethylene)hydrazinecarboximidamide hydrochloride **4** was obtained as a brown solid (146.7 mg, 0.585 mmol, 93% yield).

E/Z ratio: E-isomer only.

Reaction time: 7 hours, then rt overnight.

TLC monitoring: eluent mixture 9:1 DCM/EtOAc.

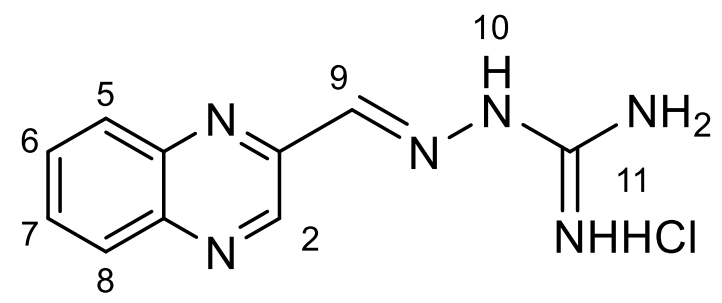

^{1}H-NMR (400 MHz, DMSO-d_6) δ 12.73 (s, 1H, H10), 9.82 (s, 1H, H2), 8.40 (s, 1H, H9), 8.15 – 8.07 (m, 6H, H5, H8, H11), 7.90 – 7.86 (m, 2H, H6, H7).

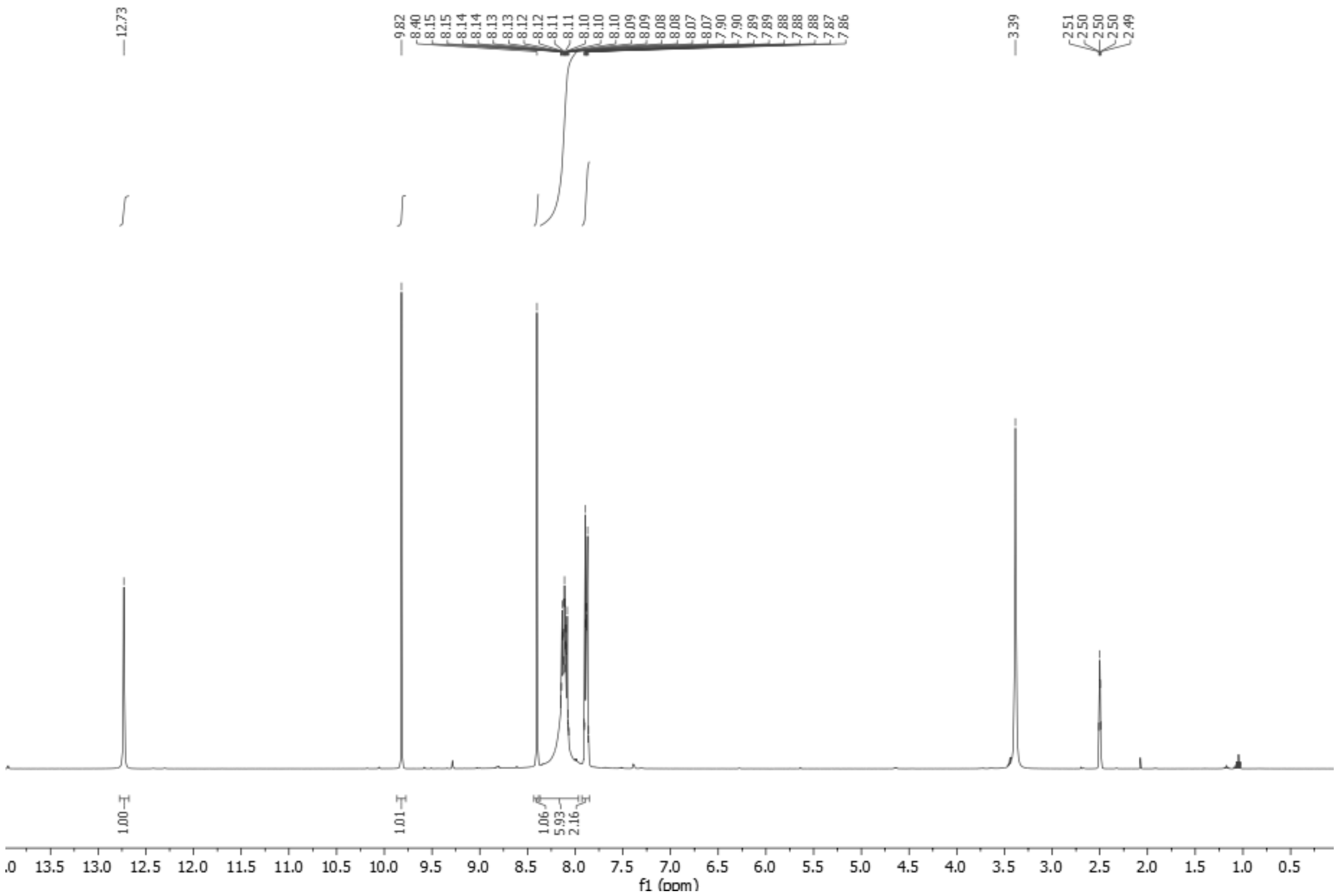

^{13}C-NMR (101 MHz, DMSO-d_6) δ 155.43, 147.73, 144.99, 143.84, 141.68, 141.11, 130.86, 130.76, 129.14, 129.01.

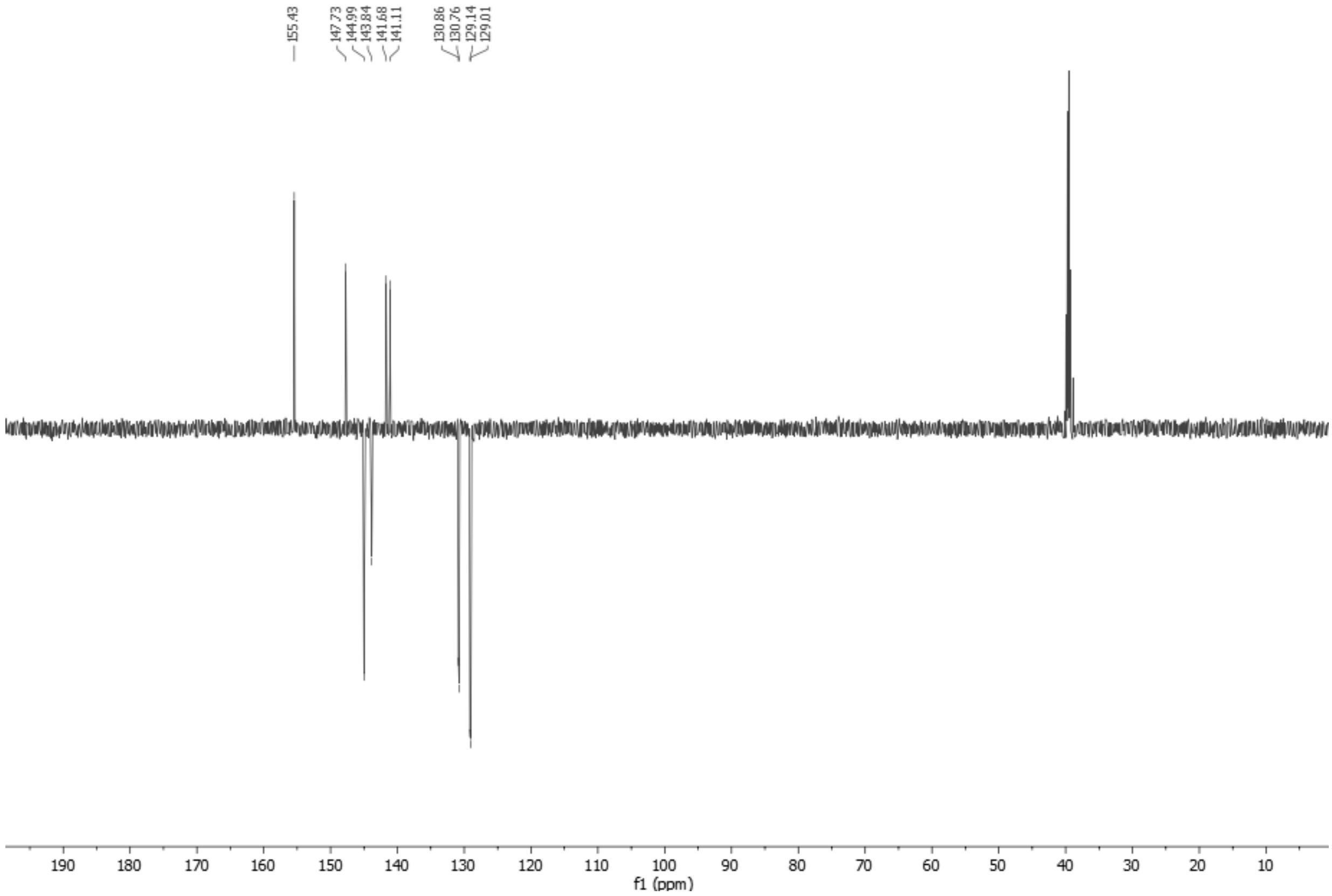

MS (ESI+): m/z [M + H+] 215.13. Calculated MS for $C_{11}H_{13}N_6$: 214.10.

Synthesis of 2-(benzo[h][1,6]naphthyridin-5-ylmethylene)hydrazine-1-carboximidamide hydrochloride 5

The synthesis of 2-(benzo[h][1,6]naphthyridin-5-ylmethylene)hydrazine-1-carboximidamide hydrochloride **5** is described in the main text; its analytical characterization is reported below.

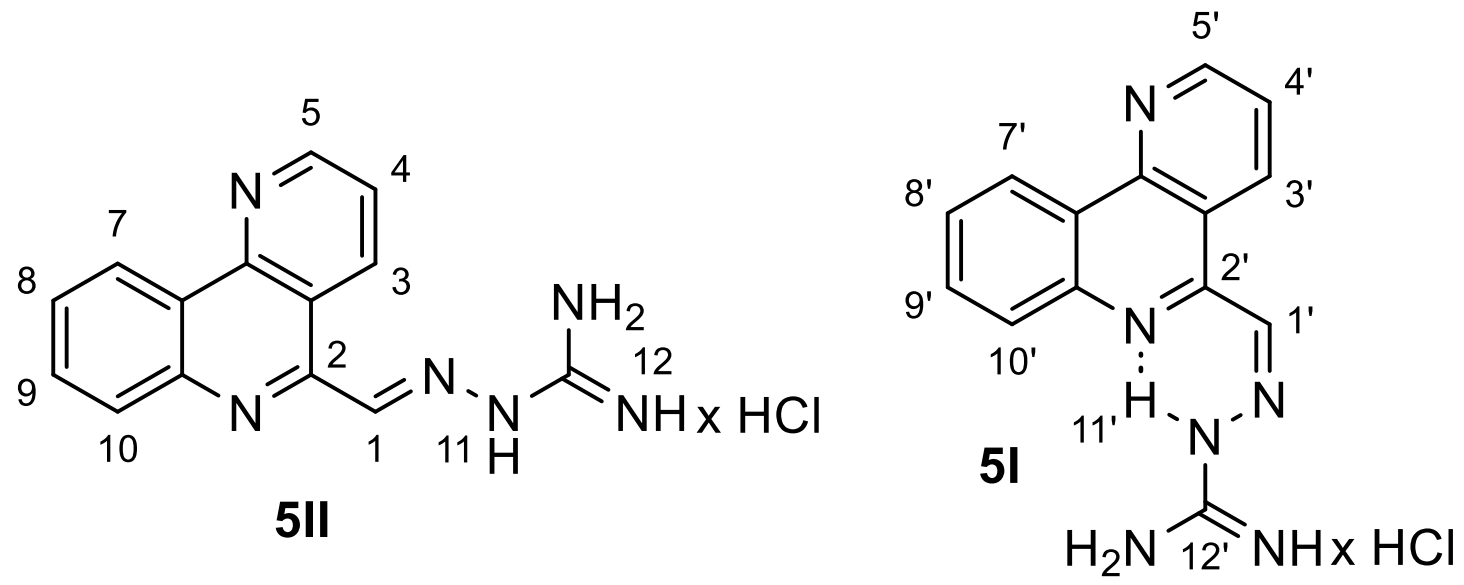

^{1}H NMR (600 MHz, DMSO-d_6) δ 14.86 (s, 1H, H11'), 12.86 (s, 1H, H11), 9.64 (dd, J = 8.4, 1.6 Hz, 1H, H3), 9.32 – 9.27 (m, 3H, H5, H3', H5'), 9.09 (dd, J = 8.2, 1.5 Hz, 2H, H7, H7'), 8.91 – 8.76 (bs, 4H, H12'), 8.80 (d, J = 8.1 Hz, 1H, H10'), 8.74 (s, 1H, H1), 8.67 (s, 1H, H1'), 8.29 (d, J = 8.1 Hz,

1H, H10), 8.11 (bs, 4H, H12), 7.99 (ddd, *J* = 8.3, 6.9, 1.5 Hz, 1H, H8'), 7.96 (ddd, *J* = 8.3, 6.9, 1.5 Hz, 1H, H8), 7.94 – 7.90 (m, 2H, H9', H4'), 7.90 – 7.86 (m, 2H, H9, H4).

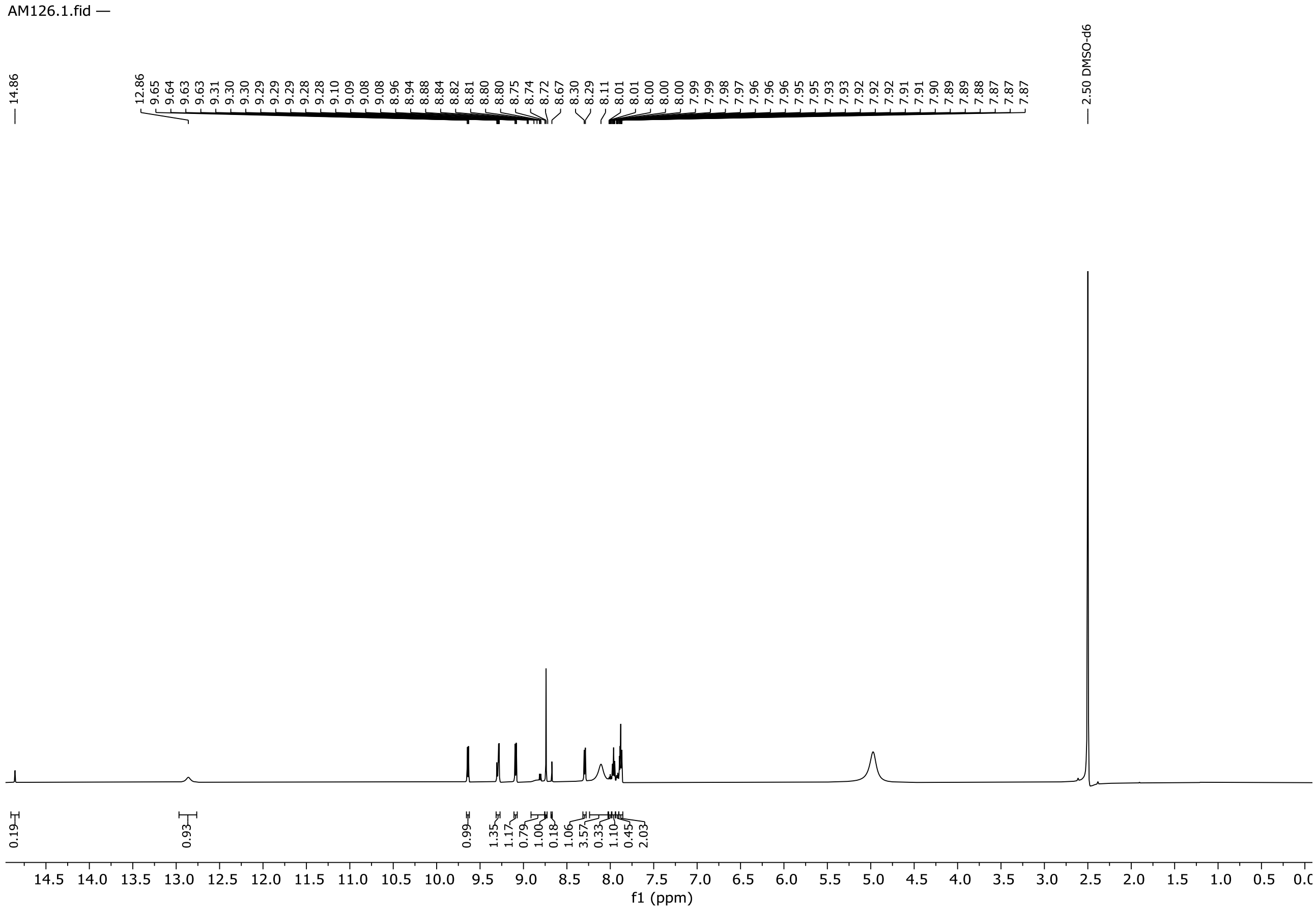

^{13}C-NMR (101 MHz, DMSO-d_6) δ 155.5, 153.9, 150.8, 148.3, 147.8, 144.1, 136.9, 131.1, 129.1, 128.7, 124.7, 124.1, 123.5, 118.7. Only the signals for the major form are reported.

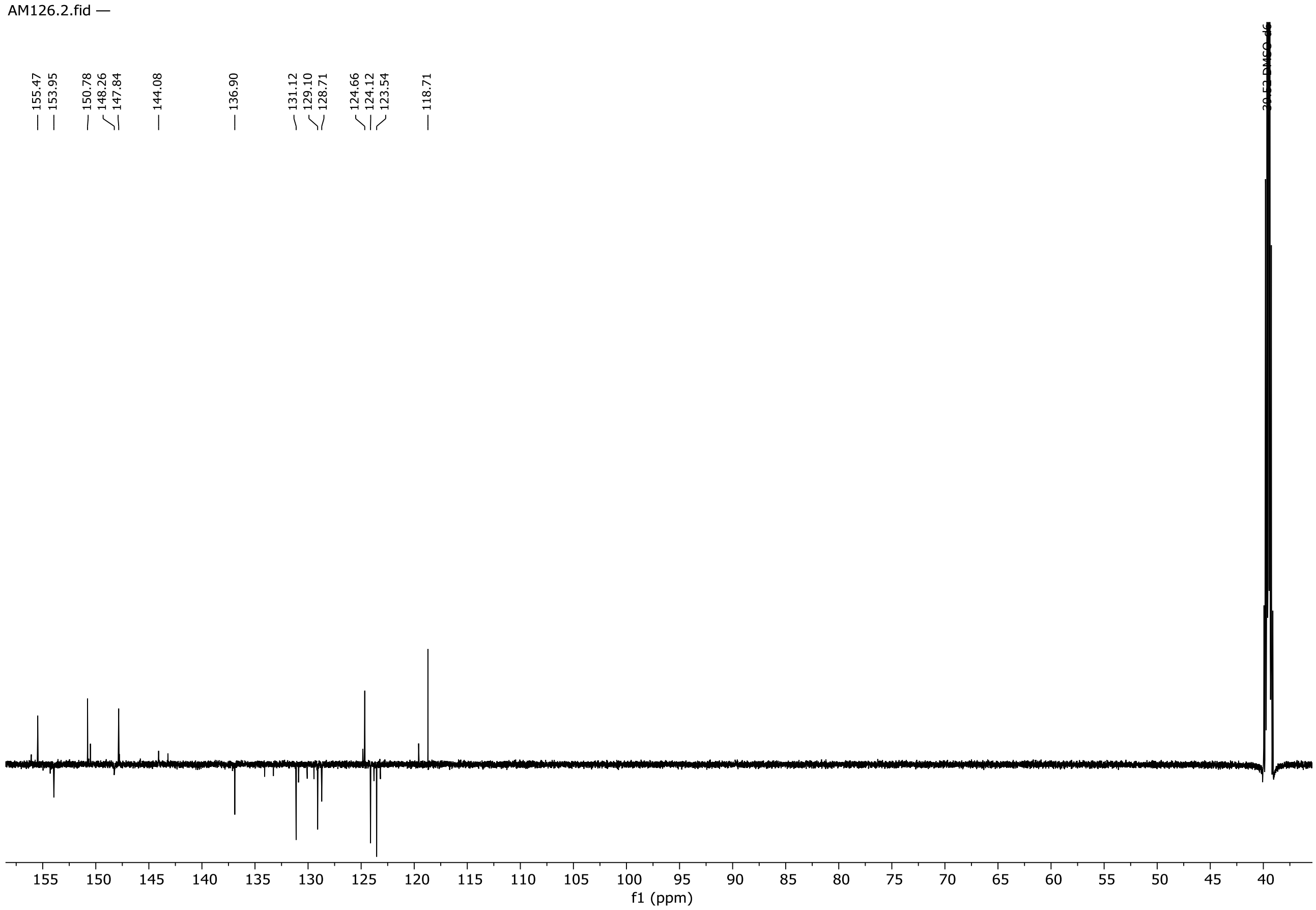
AM126.2.fid —
155.47
153.95
150.78
148.26
147.84
144.08
136.90
131.12
129.10
128.71
124.66
124.12
123.54
118.71
39.52 DMSO-d6
f1 (ppm)

BIBLIOGRAPHY

[30]. Arumugam, A.; Senadi, G.C. Visible-Light Photocatalyzed C-N Bond Activation of Tertiary Amines: A Three-Component Approach to Synthesize Quinazolines. *Org. Biomol. Chem.* **2024**, 22 (6), 1245–1253. https://doi.org/10.1039/d3ob02067c

[51]. Iguchi, D.; Erra-Balsells, R.; Bonesi, S.M. Photo-Fries Rearrangement of Aryl Acetamides: Regioselectivity Induced by the Aqueous Micellar Green Environment. *Photochem. Photobiol. Sci. Off. J. Eur. Photochem. Assoc. Eur. Soc. Photobiol.* **2016**, 15 (1), 105–116. https://doi.org/10.1039/c5pp00349k

[52]. Bichovski, P.; Haas, T., Kratzert, D.; Streuff, J. Synthesis of Bridged Benzazocines and Benzoxocines by a Titanium-Catalyzed Double-Reductive Umpolung Strategy. *Chem. Eur. J.* **2015**, 21 (6), 2339–2342. https://doi.org/10.1002/chem.201405852

[53]. Sherborne, G.J.; Diène, C.; Kemmitt, P.; Smith, A.D. Access to a Diverse Array of Bridged Benzo[1,5]Oxazocine and Benzo[1,4]Diazepine Structures. *Org. Lett.* **2023**, 25 (48), 8585–8589. https://doi.org/10.1021/acs.orglett.3c03392.

[54]. Zhang, Y.; Cao, N.; Gao, J.; Liang, J.; Liang, Y.; Xie, Y.; Zhou, S.; Tang, X. ASIC1a stimulates the resistance of human hepatocellular carcinoma by promoting EMT via the AKT/GSK3beta/ Snail pathway driven by TGFbeta/Smad signals. *J. Cell. Mol. Med.* **2022**, 26, 2777–2792. https://doi.org/10.1111/jcmm.17288

ANALYTICAL CHEMISTRY

E/Z isomeric GH forms - compound 1c

Both ^{1}H-NMR and LC-MS analyses revealed the presence of two E/Z stereoisomers for most prepared GMQ-like analogues. The Z stereoisomer is characterized by an intramolecular hydrogen bond between a proton of the GH group and the nitrogen atom in position 3 of the adjacent heterocycle. NOESY data, shown in Figure S1 for 2-((6-chloromethylquinazolinyl)methylene)hydrazine-1-carboximidamide hydrochloride **1c** as an example, do not show the presence of a hydrogen bond with the nitrogen in position 1, which could also be expected since this interaction would likewise form a six-membered ring. We hypothesize that this is due to the inductive effect of the methyl group in position 4 on the nitrogen in position 3, which would enhance its basicity. The percentage ratio observed for the two forms is reported in the manuscript, although this ratio depends on the amount of water present in the NMR sample or in the LC-MS analysis.

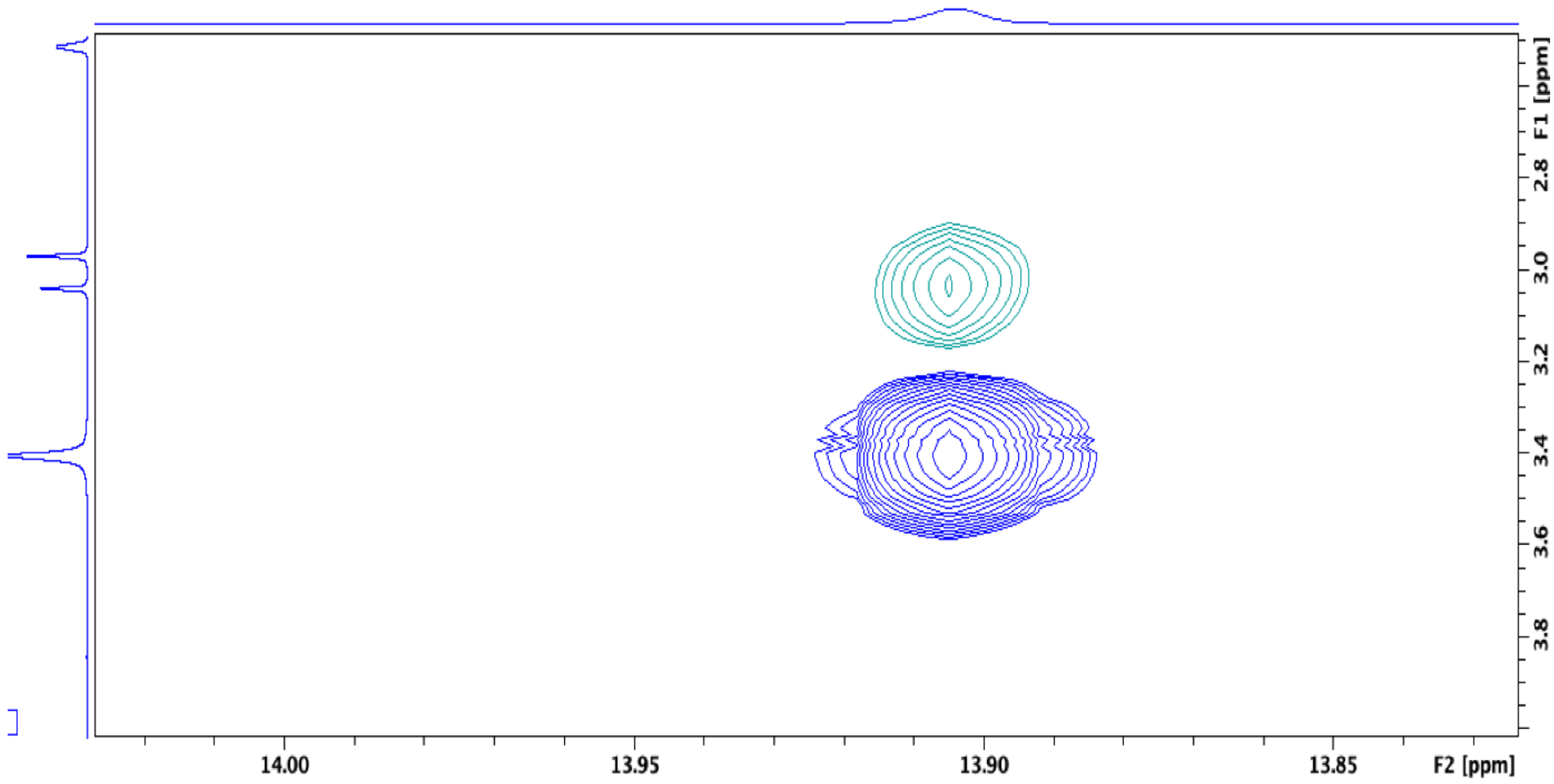

*Figure S1 –NOESY spectrum for GMQ-like analogue **1c**.*
